# Catalytic Amino Group Transfer Reactions Mediated by Photoinduced Nitrene Formation from Rhodium‐Hydroxamates

**DOI:** 10.1002/anie.202422461

**Published:** 2025-02-25

**Authors:** Hoimin Jung, Jeonguk Kweon, Jong‐Min Suh, Andrés Arribas, Dongwook Kim, Mi Hee Lim, Sukbok Chang

**Affiliations:** ^1^ Center for Catalytic Hydrocarbon Functionalizations Institute for Basic Science (IBS) Daejeon 34141 South Korea & Department of Chemistry Korea Advanced Institute of Science and Technology (KAIST) Daejeon 34141 South Korea; ^2^ Department of Chemistry Korea Advanced Institute of Science and Technology (KAIST) Daejeon 34141 South Korea; ^3^ Centro Singular de Investigación en Química Biolóxica e Materiais Moleculares (CiQUS) and Departamento de Química Orgánica Universidade de Santiago de Compostela 15782 Santiago de Compostela Spain

**Keywords:** Metal-nitrenoids, Photochemistry, Amination, Rhodium, Organometallics

## Abstract

Herein, we report a photocatalytic platform to access transient nitrenoids by designing photo‐responsive neutral rhodium‐hydroxamate complexes. Combined experimental and computational mechanistic studies, including electron paramagnetic resonance (EPR) and mass spectrometric analysis, suggest that electrophilic Fischer‐type Rh‐acylnitrenoid intermediates could be generated via photoactivation of corresponding Rh‐hydroxamates via N‐O bond homolysis and redox event. Moreover, catalytic acylnitrenoid transfer was explored toward the amidation of various hydrocarbons, amines, and alcohols to furnish new N−C, N−N, and N−O bonds.

## Graphical abstract

We investigated the photoinduced generation of Rh‐acylnitrenoids and their catalytic nitrene transfer reactivity using thermally stable, yet photoactive Rh‐hydroxamate. Combined EPR, mass, and DFT studies suggested that Fischer‐type Rh‐acylnitrenoid species could be furnished via the photoactivation of Rh‐hydroxamates. Further catalytic amidation reactivity of hydrocarbons, amines, and alcohols to form new C−N, N−N, and N−O bonds was explored.

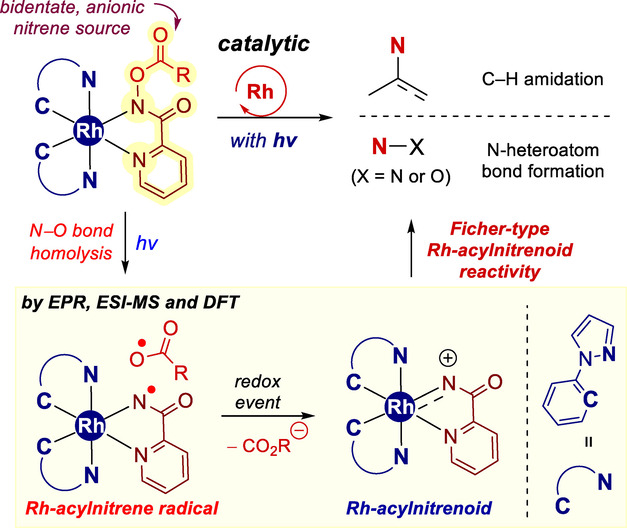

Transition‐metal nitrenoid species are pivotal intermediates for constructing synthetically and pharmaceutically valuable amine products via catalytic C−H amination of hydrocarbons (Scheme 1a).[^1^, ^2^, ^3^] In this context, a diverse array of amino precursors, including azides,[^4^, ^5^, ^6^] hydroxylamines,[^7^, ^8^, ^9^, ^10^] iminoiodinanes,[^11^, ^12^] and dioxazolones,[^13^, ^14^, ^15^] have been employed to generate highly reactive metal‐nitrenoids, primarily via thermal, two‐electron activation pathways. While these thermally generated intermediates have been reported to facilitate the catalytic formation of C−N, N−N, and N‐heteroatom bonds,[^16^, ^17^, ^18^, ^19^, ^20^, ^21^, ^22^, ^23^, ^24^, ^25^, ^26^] spontaneous but uncontrollable nitrenoid formation has lagged behind the examination of the key intermediates.

**Scheme 1:**
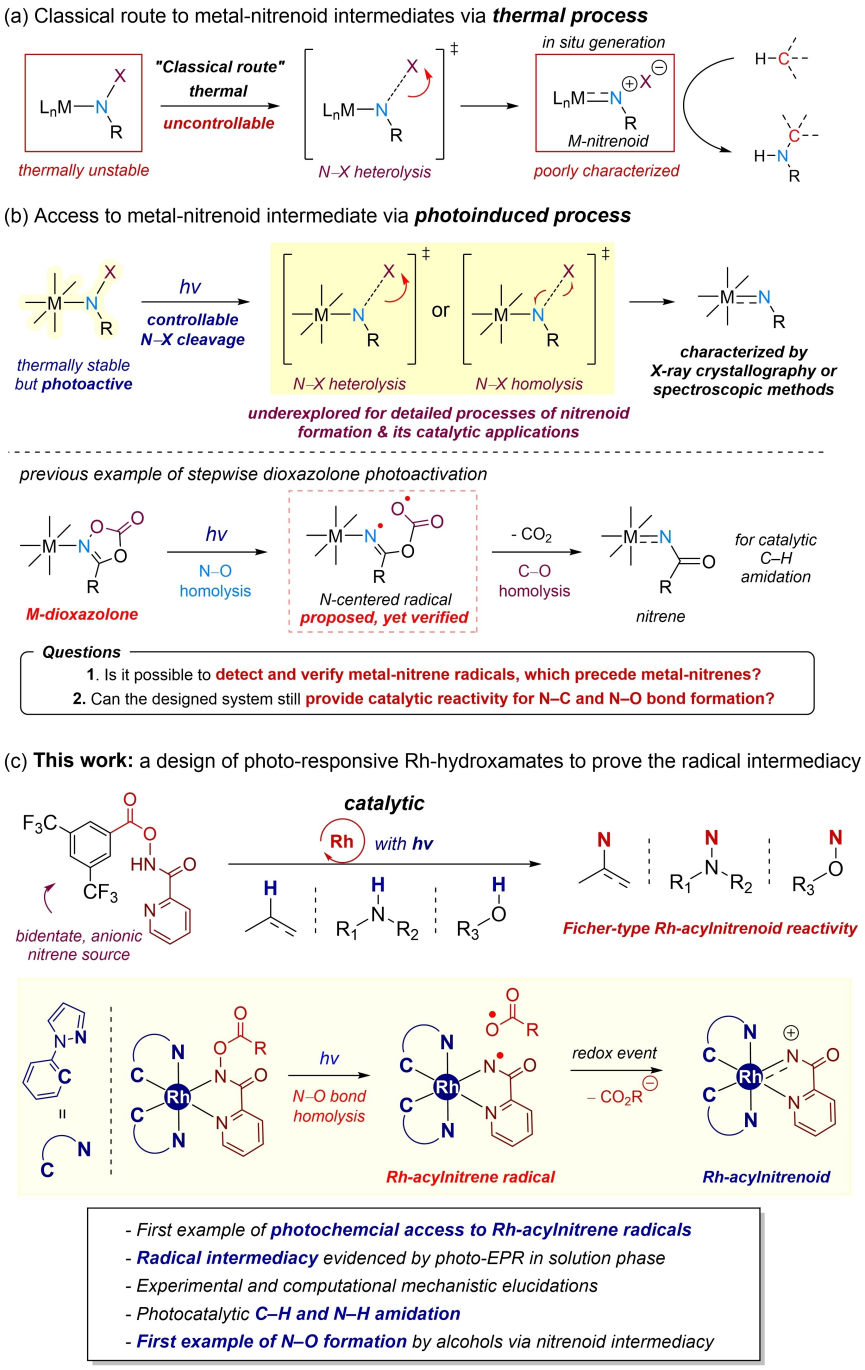
Nitrene Photocatalysis of Rh‐Hydroxamates.

In addition to the classical thermal two‐electron routes for generating metal‐nitrenoids, photochemical activation of metal‐nitrenoid precursor complexes^[27]^ has recently emerged as an alternative route to access reactive metal‐nitrenoid intermediates (Scheme 1b). This strategy is particularly useful in characterizing transient metal‐nitrenoid intermediacy via photocrystallographic analysis, as exemplified by Powers, Schneider, Betley, Albrecht, and our group.[^28^, ^29^, ^30^, ^31^, ^32^, ^33^, ^34^, ^35^, ^36^] While the photochemical strategy is highly promising in the activation of metal‐ligated nitrene precursors such as Rh‐ or Mn‐*N‐*haloamidos[^31^, ^37^] and Ir‐hydroxamates,^[38]^ only stoichiometric reactivity has been studied. While there are examples of photoinduced, catalytic metal‐nitrenoid transfer, it is still challenging to reveal the detailed mechanistic steps, which eventually generate reactive metal‐nitrenoid intermediates involved in the catalytic cycle.[^39^, ^40^, ^41^, ^42^, ^43^, ^44^, ^45^, ^46^] Although our group recently reported photocatalytic generation of Rh‐acylnitrenoid from Rh‐dioxazolone via stepwise, two‐step N−O and C−O bond cleavage mechanism, the preceding Rh‐nitrene radical species has only been proposed based on computational studies (Scheme 1b).^[36]^

Building on our recent study using Cp*Ir‐hydroxamate as a metal‐acylnitrenoid photoprecursor via metal‐to‐ligand charge transfer (MLCT) that mediates stoichiometric nitrene group transfer,^[38]^ we wondered whether our concept of photoinduced activation of metal‐hydroxamates could be generalized into catalytic processes. To this end, we demonstrated the photochemical metal‐nitrene radical formation using readily accessible, bench‐stable hydroxamates as acylnitrenoid precursors in conjunction with a light‐harvesting rhodium catalyst platform (Scheme 1c).

We herein demonstrate photoinduced Rh‐nitrenoid formation and its catalytic transfer reactions with C(sp^2^ or sp^3^)–H bonds, amines, and alcohols to form N−C or N–heteroatom bonds, by employing bidentate and anionic hydroxmate nitrene precursor. Our combined experimental and computational studies, including solution‐phase *in situ* photo EPR analysis and mass spectrometric studies, suggest that photochemical activation of Rh‐hydroxamate complexes can lead to the electrophilic Fischer‐type Rh‐acylnitrene intermediate. Further catalytic reactivity for the C−H and N−H amidation was also successfully demonstrated in addition to the N−O bond formation in reaction with alcohol nucleophiles.

Based on the literature precedents of utilizing light‐harvesting properties of octahedral rhodium complexes as photocatalysts,[^47^, ^48^, ^49^, ^50^] we designed a new platform for photochemical access to metal‐nitrene radicals, eventually enabling the formation of reactive metal‐nitrenoids. Since the previous use of the cationic [(PzPh)_2_Rh(dioxazolone)]^+^ complexes (PzPh=1‐phenyl pyrazole) was found to undergo fast decarboxylation events via sequential N−O and C−O bond cleavage upon photoirradiation,^[36]^ we pursued to search for suitable photoprecursors to enable access to the sought‐after metal‐nitrenoid intermediates. Considering the tunability and stability of hydroxamates,^[51]^ where one N−O bond‐breaking event is expected to occur to generate the desired intermediate (Scheme 2a), a neutral Rh‐hydroxamate complex **Rh1a** was prepared almost quantitatively by treating [(PzPh)_2_RhCl]_2_ (0.5 equiv)^[36]^ with *N‐*3,5‐bis(trifluoromethyl)benzoyl picolinamide (**1 a**, 1.0 equiv)[^52^, ^53^] and triethylamine base (1.0 equiv) in dichloromethane (Scheme 2b).

**Scheme 2:**
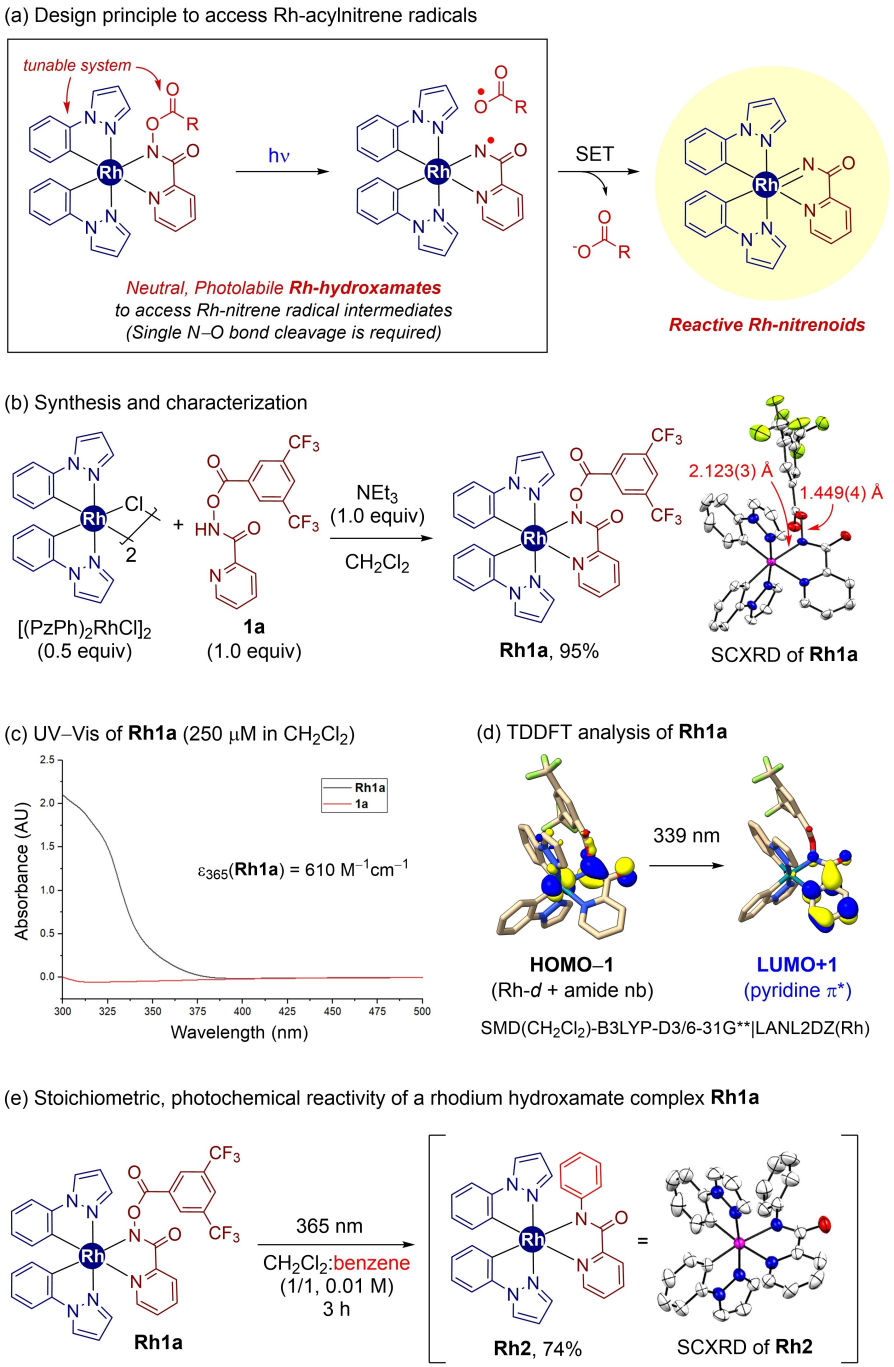
Design, Synthesis, and Photochemical Properties of a Rhodium‐Hydroxamate Complex **Rh1a**.

The photochemical properties of **Rh1a** were then investigated. While Rh‐hydroxamate complex **Rh1a** showed an absorption feature near 360 nm (ϵ_365_=610 cm^–1^M^–1^), *N*‐benzoyloxyamide **1 a** had only marginal absorption at this wavelength (Scheme 2c). Furthermore, time‐dependent density functional theory (TDDFT) simulation suggests that the absorption at ~360 nm could be assigned to be the HOMO–1 to LUMO+1 transition, where the HOMO–1 of **Rh1a** is comprised of mixed Rh‐*d* and amide nonbonding characters, and LUMO+1 is a π* orbital of the pyridine group (Scheme 2d).

Eventually, when **Rh1a** was irradiated with light in a 1 : 1 co‐solvent system of DCM and benzene, 74 % of the benzene amidated product was obtained, corresponding to the reactivity of the Rh‐acylnitrene intermediate (Scheme 2e).^[36]^ When separately synthesized **Rh2** was treated with **1 a** (1.0 equiv) and **Ar^F^COOH** (1.0 equiv) as the proton source, we observed the quantitative regeneration of **Rh1a** with the extrusion of amidated product within 10 min (see the Supporting Information for details), implying the potential catalytic application of the present rhodium complex.

Based on the promising stoichiometric photochemical C(sp^2^)–H amidation reactivity, we expanded the reaction system to a catalytic procedure (Table 1). In a model reaction, *N*‐bis(trifluoromethyl)benzoyloxypicolinamide **1 a** was examined as the amino group source to react with benzene (5 equiv). We hypothesized that in situ extruded **Ar^F^COOH** would serve as the proton source for catalytic turnovers. Pleasingly, employing 5 mol % of **Rh1a** as the catalyst gave rise to 84 % of C(sp^2^)–H amidation product **2** under 365 nm irradiation conditions (entry 1).^[54]^ Without the **Rh1a** catalyst or light source, the desired product was not detected while starting material **1 a** remained intact (entries 2 and 3), thus highlighting the importance of both photoirradiation and metal catalyst in the present reaction system. Interestingly, the choice of light wavelength was critical for the efficiency of the catalytic C−H amidation, as using 395 nm or 420 nm light source significantly decreased the yield of **2** to 63 % and 39 %, respectively (entries 4 and 5). This observation is in agreement with the UV–Vis analysis, as **Rh1a** showed marginal absorption features at 395 and 420 nm (Scheme 2c, see above).

**Table 1:**
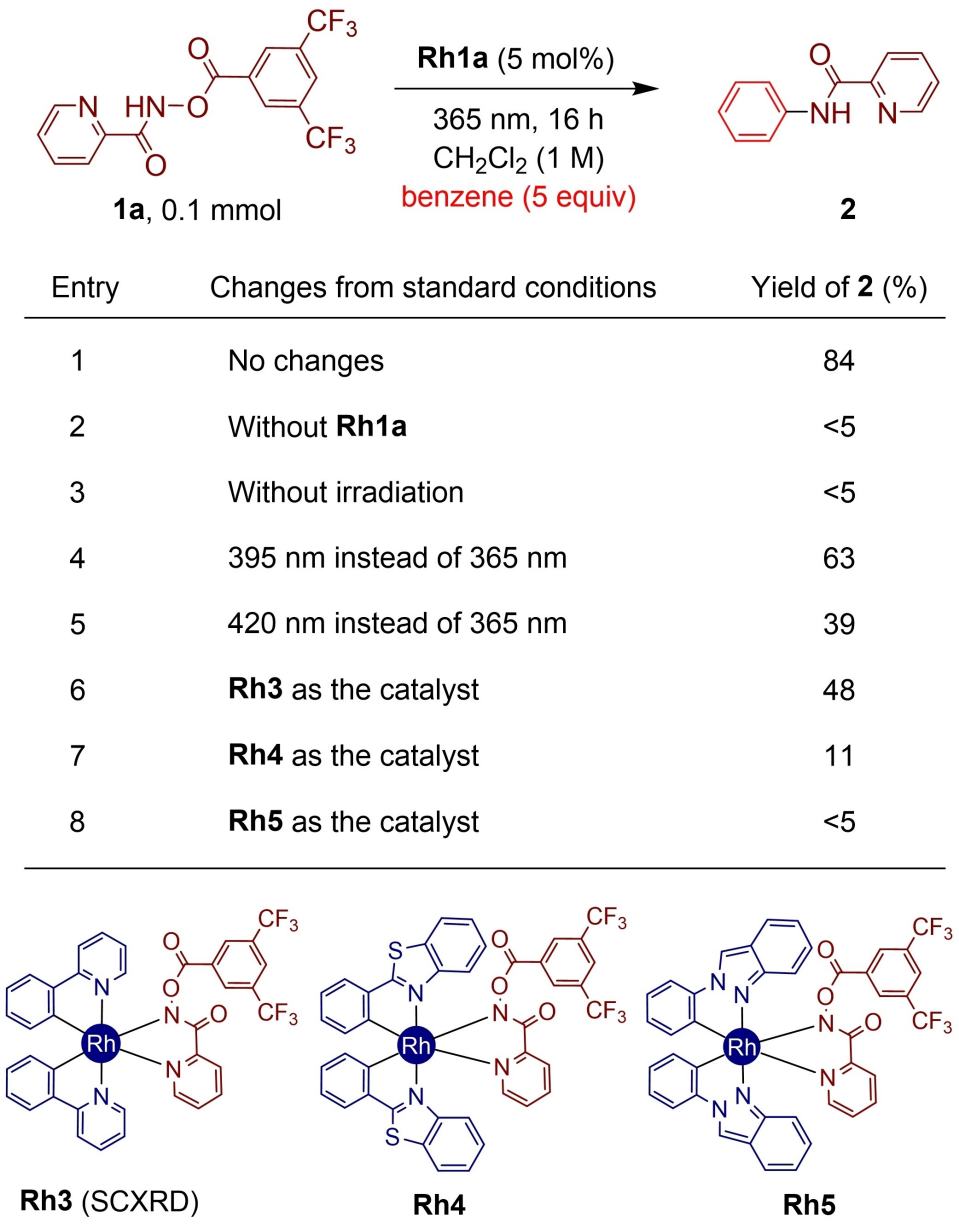
Photochemical Reactivity of Newly Designed Rh‐Hydroxamate Complexes.^[a]^

^[a]^ Yields are based on ^1^H NMR analysis of the crude reaction mixture in the presence of 1,1,2‐trichloroethane as an internal standard. Merck Penn PhD photoreactor M2 (LED intensity=100 %) was utilized. Yields are calculated based on the amount of employed **1 a**.

Additionally, the catalytic reactivity of a range of Rh‐hydroxamate complexes with different LX‐type ligands was investigated. Employing a Rh‐hydroxamate complex with 2‐phenylpyridine ligand (**Rh3**) showed the amido group transfer toward benzene, but with a decreased yield of **2** (48 %, entry 6). Of particular note, when using benzothiazole (**Rh4**) or indazole (**Rh5**)‐ligated Rh‐hydroxamate complexes (entries 7 and 8), which were proved to mediate photocatalytic radical transformations by Meggers and co‐workers,[^48^, ^55^] gave only poor efficiency in the present reaction system. This reactivity difference would be ascribed to the π‐abundant nature of the cyclometalated LX‐ligands in **Rh4** or **Rh5** when compared to the PzPh ligand (**Rh1a**). In fact, in the **Rh4** and **Rh5** complexes, the postulated Rh‐to‐LX‐ligand charge transfer may dominate over the desired hydroxamate activation pathway.^[50]^ Indeed, further UV–Vis and TDDFT analysis of **Rh3–Rh5** revealed that Rh‐to‐LX‐ligand charge transfer is much preferred over hydroxamate photoactivation (see the Supporting Information for details).

Having identified that cyclometalated rhodium complex **Rh1a** and its derivatives can induce catalytic C−H amination under photoirradiation conditions, we next tried to probe the key intermediates of the current reaction. Based on our initial working hypothesis, the putative reaction mechanism is illustrated in Figure 1a, including i) photoinduced N−O bond homolysis of **Rh1a** to form nitrene radical ^**2**^ **RhA** and **Ar^F^COO•** ii) single electron transfer (SET) to generate the Rh‐acylnitrenoid ^**1**^ **RhA**, iii) C−H amidation of benzene to produce product complex **Rh2**, and iv) ligand substitution with **2 a** to regenerate **Rh1a**.

**Figure 1:**
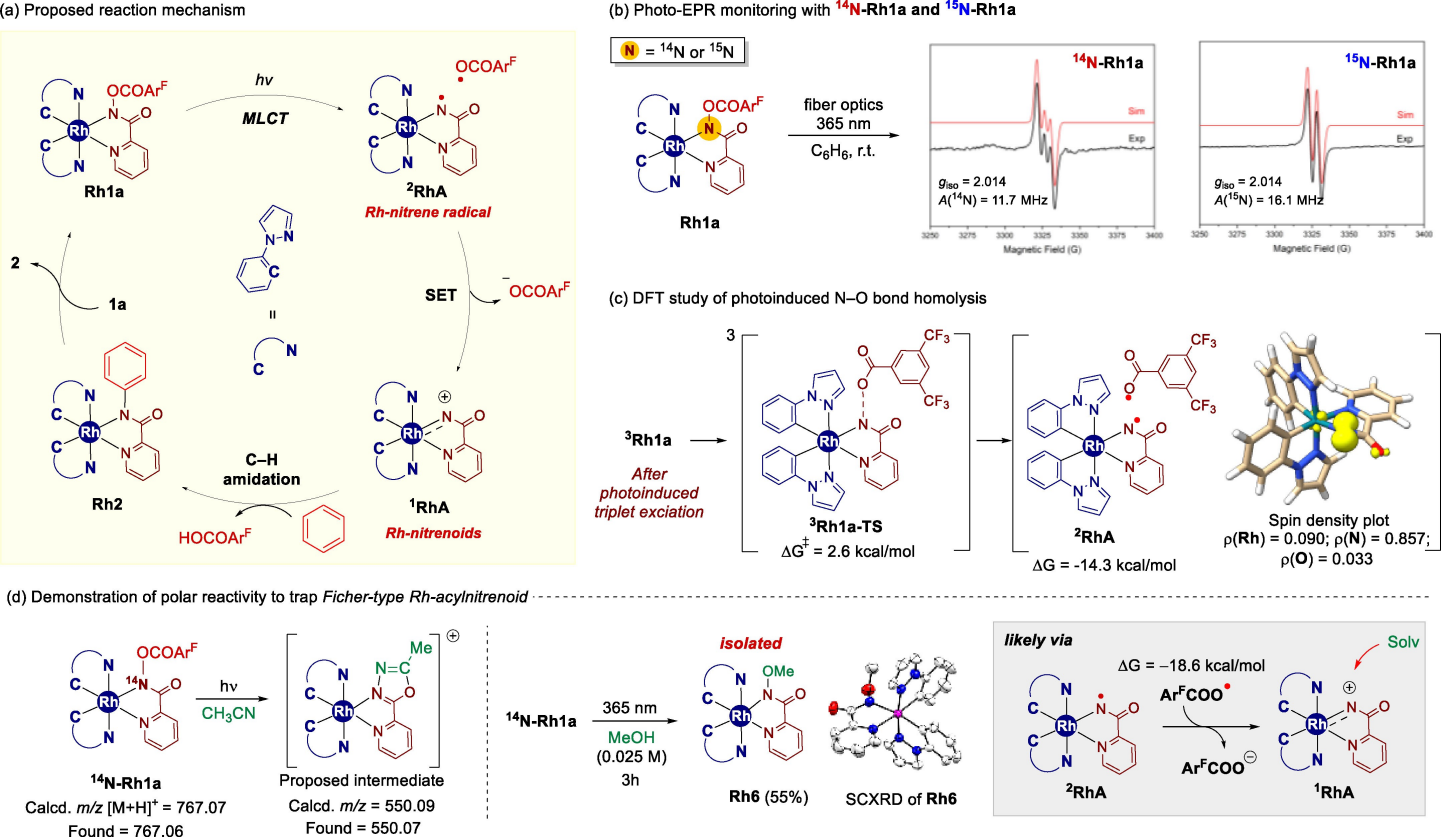
Mechanistic Investigations of the formation of Rh‐nitrene radical ^**2**^ **RhA** and Rh‐acylnitrene ^**1**^ **RhA**

First, we monitored X‐band electron paramagnetic resonance (EPR) coupled with a 365 nm fiber optics setup to verify whether open‐shell intermediates would be involved in this transformation (see Figure 1b and Supporting Information for the detailed setup). For this purpose, ^**14**^ **N‐Rh1a** was dissolved in benzene (10 mM) and irradiated at room temperature during EPR measurement. To our surprise, an isotropic, triplet signal was indeed obtained, indicative of an S=1/2 system (where S is the spin quantum number, Figure 1b). An isotropic hyperfine interaction (HFI), presumably caused by a ^14^N atom, was observed (*A* ^14N^_*iso*_ = 11.7 MHz) at *g*_iso_=2.014, which is consistent with those of organic N‐centered radical species.[^56^, ^57^, ^58^]

When conducting EPR monitoring under light irradiation using isotopically labeled ^**15**^ **N‐Rh1a**, a clear doublet signal with *g*_iso_=2.014 and *A* ^15N^_*iso*_=16.1 MHz appeared (Figure 1b), thus indicating that the observed EPR signal is assignable to the proposed N‐centered radical species ^**2**^ **RhA**. Of note, similar transition metal‐nitrene radical species such as Co‐sulfonylnitrene radicals,[^58^, ^59^, ^60^] Fe(III)‐nitrene radical,^[61]^ Pd‐arylnitrene radical,^[62]^ and Rh‐tosylnitrene radical,^[63]^ yet, to our best knowledge, this is the first example to access Rh‐acylnitrene radical species. Both ^14^N and ^15^N‐derived signals decayed rapidly over time under light irradiation or when the light source was turned off, thus suggesting that the generated open‐shell intermediate is transient and the signal growth is photo‐responsive (see the Supporting Information for details). Of note, the corresponding acyloxy radical was not detected, likely due to rapid decomposition including Kolbe‐type decarboxylation pathway.[^64^, ^65^] The computationally assessed barrier of this N−O cleavage of triplet‐excited ^**3**^ **Rh1a** is only 2.6 kcal/mol to furnish a radical pair of ^**2**^ **RhA** and acyloxy radical **Ar^F^COO•** (see the Supporting Information for the detailed computational results). While we could not observe the hyperfine interaction with the rhodium atom, it was attributed to the highly N‐atom‐localized spin density of ^**2**^ **RhA** intermediates (Figure 1c).

Computational simulations of the EPR parameters of ^**2**^ **RhA** also showed very low hyperfine coupling constants (<0.1 MHz) for the rhodium center, further explaining the lack of resolved HFI in the above EPR experiment at room temperature (see the Supporting Information for more details on the EPR simulation study). When conducting additional EPR experiments with a spin‐trapping agent DMPO (5,5‐dimethyl‐1‐pyrroline‐*N*‐oxide), multiple species with an S=1/2 system were observed, suggesting that both *N*‐and *O*‐centered radicals may be trapped under these conditions (see the Supporting Information for details).

Next, we sought to detect the relevant transient species through mass spectrometric methods.[^66^, ^67^, ^68^, ^69^] Hence, we conducted electrospray ionization mass spectrometry (ESI–MS) experiments to probe the existence of photoinduced fleeting intermediates (Figure 1d). Upon photoirradiation of ^**14**^ **N‐Rh1a** in acetonitrile, a new peak at *m/z*=550.07, corresponding to the adduct of Rh‐acylnitrene **RhA** and MeCN, was observed while the original mass value of ^**14**^ **N‐Rh1a** (*m/z*=767.06 as [M+H]^+^ form) completely vanished (Figure 1d, left). It is worth noting that analogous ESI‐MS experiments using ^**15**^ **N‐Rh1a** and/or **CD_3_CN** also strongly support the same conclusion (see the Supporting Information for details). While attempts to isolate the proposed acetonitrile adduct were unsuccessful, we propose that the observed Ritter‐type solvolysis outcome[^70^, ^71^] might be attributed to the reaction of the electrophilic metal‐nitrenoid intermediate with acetonitrile.

Interestingly, while we detected a new mass value of *m/z*=563.06 upon photoirradiation of **Rh1a** in MeOH solvent (see the Supporting Information for ESI‐MS experiments), a preparatory scale photolysis successfully provided **Rh6** (calc. *m/z*=563.07, 55 %), where the original N−O bond of the hydroxamate is cleaved with a concomitant new N–OMe bond formation. The structure of **Rh6** was confirmed by SCXRD.^[88]^ This stoichiometric, polar solvolysis‐type reactivity with alcohol is again indicative of the potential involvement of cationic Rh‐acylnitrenoid ^**1**^ **RhA** intermediate, considering the capability of electrophilic Fischer‐type metal‐nitrenoids to undergo N−P,[^41^, ^72^, ^73^, ^74^, ^75^] N−N,[^76^, ^77^, ^78^, ^79^] and N–S[^39^, ^80^, ^81^, ^82^, ^83^] bond formation with external nucleophiles. Based on our hypothesis that a metal‐nitrenoid is likely involved, we propose that the single‐electron oxidation of the spectroscopically observed ^**2**^ **RhA** leads to the formation of the corresponding nitrene species ^**1**^ **RhA** (Figure 1d, right). Indeed, computational estimation suggests that ^**2**^ **RhA** could be easily oxidized with acyloxy radical (**Ar^F^COO•**) to furnish its oxidized Rh‐acylnitrene (^**1**^ **RhA**) intermediate along with benzoate (**Ar^F^COO** ^–^) by 18.6 kcal/mol.

To understand the mechanism of this solvolysis‐type reactivity, we further conducted DFT computations on the N−O bond formation pathways from two potential intermediates, ^**2**^ **RhA** and ^**1**^ **RhA** (Figure 2). The direct new N−O bond formation pathway from the nitrene radical intermediate ^**2**^ **RhA** requires a high barrier of 34.2 kcal/mol, and the overall path is highly endergonic to furnish ^**2**^ **RhB**. On the other hand, an O−H abstraction of ^**2**^ **RhA** from methanol^[84]^ may take place with a much lower barrier of 12.9 kcal/mol via ^**2**^ **RhA‐TS’** to form a Rh‐picolinamido complex ^**1**^ **RhC**, which is catalytically incompetent. In contrast, the formation of ^**1**^ **RhB** on its singlet energy surface via nucleophilic attack of methanol to ^**1**^ **RhA** requires only an 8.9 kcal/mol barrier traversing ^**1**^ **RhA‐TS**. Hence, the nucleophilic addition of solvent molecule toward electrophilic N‐atom of Rh‐acylnitrenoid ^**1**^ **RhA** agrees well with our mass spectrometric experiments.

**Figure 2:**
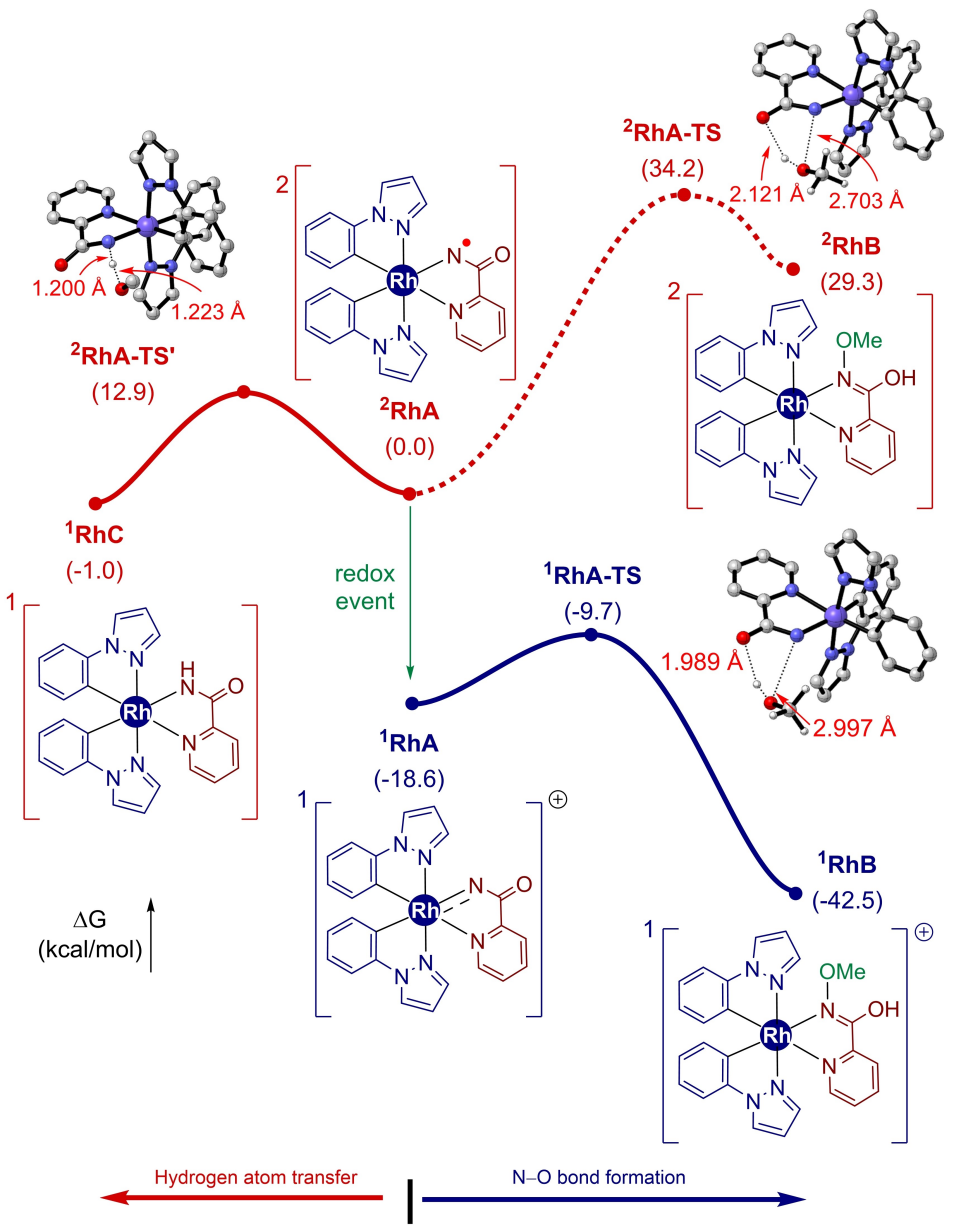
Gibbs energy profile of **Rh6** formation pathways from ^**2**^ **RhA** and ^**1**^ **RhA**.SMD(MeOH)‐B3LYP−D3/6–311+G**|SDD(Rh) // B3LYP−D3/6‐31G** | LANL2DZ(Rh).

The successful demonstration of photocatalytic C−H amidation reactivity of the newly designed Rh‐hydroxamate system along with EPR experiments suggests that Rh‐nitrene radical ^**2**^ **RhA** may be initially formed upon photoirradiation of the **Rh1a** complex. Eventually, the solvolysis‐type photoreactivity and corresponding experimental outcomes indicate that Rh‐acylnitrenoid ^**1**^ **RhA** likely leads to the amido group transfer reaction. With the mechanistic insight of the present reaction in hand, we subsequently investigated the substrate scope of the current nitrene transfer protocol (Table 2). Of note, the resulting *N*‐alkyl(aryl)picolinamide is well known to readily convert into deprotected alkyl(aryl)amines under reductive conditions.[^85^, ^86^] Monosubstituted arenes such as toluene (**5**, 58 %), anisole (**6**, 56 %), chlorobenzene (**7**, 58 %), and phenol (**8**, 48 %) successfully delivered the desired amidation products as regioisomeric mixtures, where *ortho*‐ and *para‐*positions to the preexisting substituents were selectively amidated over its *meta‐*position.

**Table 2:**
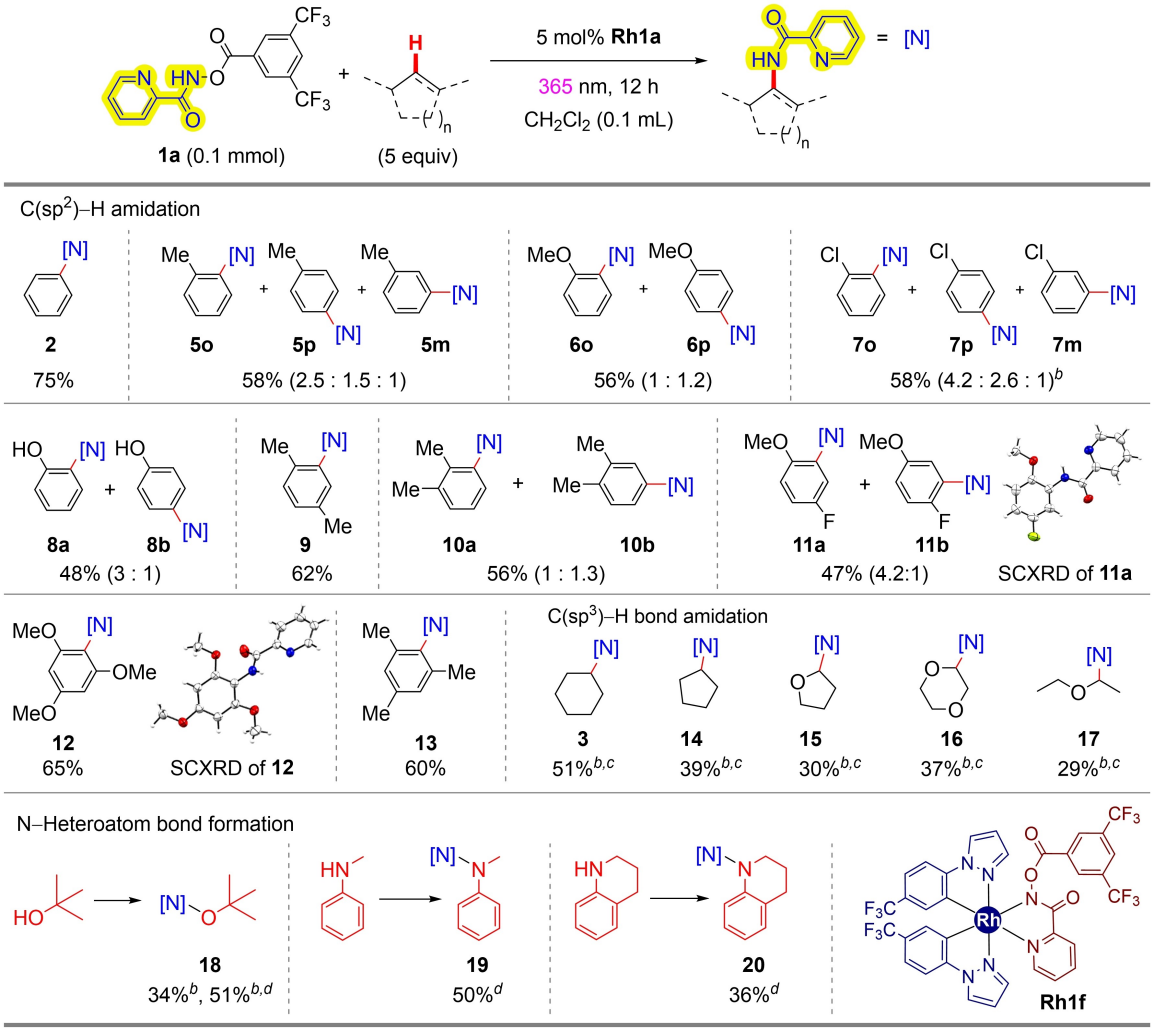
Substrate scope.^[a]^

^[a]^ Conditions: **1 a** (0.1 mmol), substrate (5 equiv), **Rh1a** (5 mol %) in CH_2_Cl_2_ (1.0 M) was irradiated with 365 nm LED (Merck Penn PhD photoreactor M2, LED intensity=100 %) for 12 h under Ar atmosphere at room temperature. ^[b]^ 0.1 mL substrate was loaded. ^[c]^ 20 mol % **Rh1a** catalyst was loaded. ^[d]^ 5 mol % **Rh1 f** was used instead of **Rh1a**; Isolated yield; Yields are calculated based on the amount of employed **1 a**.

Disubstituted arenes such as *p*‐xylene and *o‐*xylene were also amidated to give corresponding aniline products **9** (62 %) and **10** (56 %), respectively. When 1,4‐disubstituted arene carrying both electron‐rich and deficient groups was employed, a C(sp^2^)–H bond adjacent to the electron‐donating group was preferentially amidated (**11**, 47 %), and the structure of the major regioisomer **11 a** was characterized by SCXRD analysis.^[88]^ Interestingly, the current protocol was amenable to amidate sterically hindered C(sp^2^)–H bonds of trisubstituted arenes such as 1,3,5‐trimethoxybenzene (**12**, 65 %) or mesitylene (**13**, 60 %), yet no sp^3^ C−H amidation was observed.

On the other hand, for substrates carrying only C(sp^3^)–H bonds, amide functionality was successfully introduced to cyclohexane (**3**, 51 %) or cyclopentane (**14**, 39 %), yet required increased catalyst and substrate loadings. Interestingly, our current photochemical amidation protocol could be fruitfully applied to selective α‐C−H amidation^[87]^ towards tetrahydrofuran (**15**, 30 %), 1,4‐dioxane (**16**, 37 %), and diethyl ether (**17**, 29 %). Notably, the observed moderate kinetic isotope effect (KIE) value (*k*_H_/*k*_D_ = 2.1), obtained from experiments using cyclohexane and its deuterated counterpart, along with the stereoretentive amidation of (*S*)‐1‐chloro‐2‐methylbutane to form **(*R*)‐4**, suggested that the singlet Rh‐acylnitrenoid ^**1**^ **RhA** is involved in a concerted C−H insertion pathway (see the Supporting Information for experimental details).

Inspired by the above‐observed solvolysis type N−O bond formation reactivity (Figure 1d), we briefly examined the catalytic reactivity with several heteroatom nucleophiles. When employing *tert*‐butanol, we obtained the corresponding hydroxylamine product **18** in 34 % yield with **Rh1a**, where it could be improved to 51 % yield by using **Rh1 f** catalyst that bears electron‐withdrawing CF_3_ substituents. We hypothesized that the CF_3_ group might enhance the electrophilicity of the proposed Rh‐acylnitrene intermediate, leading to improved reactivity toward external nucleophiles. It needs to be mentioned that this type of solvolysis of the putative rhodium acylnitrenoid represents the first example of catalytic N−O bond formation with external alcohol nucleophiles.^[36]^ Significantly, the reaction was also amenable to constructing hydrazides^[76]^ using secondary anilines as the substrates (**19** and **20**).

In conclusion, this study presents the first demonstration of catalytic amido group transfer reactivity using photo‐responsive rhodium‐hydroxamate complexes. Using *in situ* EPR analysis in solution, N‐centered radical species was observed, which could be assigned as the Rh‐acylnitrene radical intermediate, generated by photochemical N−O bond activation of Rh‐hydroxamates. Furthermore, mass spectrometry, solvolysis‐type reactivity, and mechanistic probe experiments all corroborated that the electrophilic, Fischer‐type Rh‐acylnitrene character of the current system would be responsible for the key C−N, N−N, and O−N bond‐forming reactivity. We believe that our findings will not only enhance the fundamental mechanistic understandings of photochemical Rh‐acylnitrenoid transfer, but also will generalize the concept of *“nitrene photocatalysis”*, which can be broadly utilized in synthetic chemistry.

## Conflict of Interests

The authors declare no conflict of interest.

## Supporting information

As a service to our authors and readers, this journal provides supporting information supplied by the authors. Such materials are peer reviewed and may be re‐organized for online delivery, but are not copy‐edited or typeset. Technical support issues arising from supporting information (other than missing files) should be addressed to the authors.

Supporting Information

## Data Availability

The data that support the findings of this study are available in the supplementary material of this article.
