# Supplementary material for: Catalytic Amino Group Transfer Reactions Mediated by Photoinduced Nitrene Formation from Rhodium‐Hydroxamates
Source: Angew Chem Int Ed Engl. 2025 Feb 25;64(15):e202422461. doi: 10.1002/anie.202422461 (PMC11976214; doi:10.1002/anie.202422461)
Supplement: Supplementary file 1 — Supporting Information [file ANIE-64-e202422461-s001.pdf]

Angewandte Chemie
Eine Zeitschrift der Gesellschaft Deutscher Chemiker

Supporting Information

Catalytic Amino Group Transfer Reactions Mediated by Photoinduced Nitrene Formation from Rhodium-Hydroxamates

H. Jung, J. Kweon, J.-M. Suh, A. Arribas, D. Kim, M. H. Lim, S. Chang**

Table of Contents

1. General considerations..2

2. Procedures for the preparation of starting materials 3

3. Procedures for the synthesis of rhodium complexes 5

3. Procedures for the photocatalyzed C–H amidation using Rh-hydroxamate catalysts..............11

4. Mechanistic experiments 22

5. Computational study..42

6. Crystallographic data...65

7. ^{1}H, ^{13}C, ^{19}F, and ^{31}P NMR spectra..74

8. References 125

1. General considerations

Reagent preparation. Unless otherwise stated, all commercial reagents and solvents were used without additional purification. Analytical thin layer chromatography (TLC) was performed on Merck pre-coated silica gel 60 F254 plates. Visualization on TLC was achieved by UV light (254 nm). Silica-gel chromatography was performed using a CombiFlash® Rf + system with RediSep® Rf Silica columns using a proper eluent. Air-sensitive liquid and solutions were transferred via syringe by using degassed solvents. Concentration of solution was carried out by using a rotary evaporator and generally followed by removal of residual solvents on a vacuum line held at 0.1–1 torr.

Product characterization: ^{1}H NMR was recorded on Bruker AVANCE III HD (400 MHz), AVANCE NEO Nanobay (400 MHz), AVANCE NEO (500 MHz), or Agilent Technologies DD2 (600 MHz). Chemical shifts were quoted in parts per million (ppm) referenced to the residual solvent peak ($CHCl_3$ in $CDCl_3$: 7.26 ppm; $CHDCl_2$ in CD_2Cl_2: 5.32 ppm; $(CD_3)SO(CD_2H)$ in DMSO-d_6). The following abbreviations were used to describe peak splitting patterns when appropriate: s = singlet, d = doublet, t = triplet, q = quartet, dd = doublet of doublet, dt = doublet of triplet, td = triplet of doublet, m = multiplet br = broad. Coupling constants, *J*, were reported in hertz (Hz). ^{13}C NMR was obtained on Bruker AVANCE III HD (100 MHz), AVANCE NEO (125 MHz), or Agilent Technologies DD2 (150 MHz) and was fully decoupled by broad band proton decoupling. Chemical shifts were reported in ppm referenced to the residual solvent peak. ^{19}F NMR was recorded on Bruker AVANCE III HD (376 MHz) or AVANCE NEO (471 MHz). Infrared (IR) spectra were acquired on Bruker Alpha ATR FT-IR Spectrometer. Frequencies are given in wave numbers (cm^{-1}), and only selected peaks were reported. The melting point was measured with Buchi Melting Point M-565. High-resolution mass spectra (HRMS) were obtained from KAIST Analysis Center for Research Advancement (KARA) via the electrospray ionization (ESI) method.

2. Procedures for the preparation of starting materials

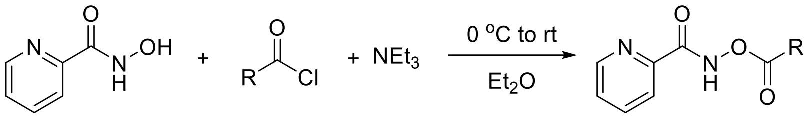

Preparation of N-(acyloxy)picolimamides. *N*-Hydroxypicolinamide was prepared by following the previously reported procedure.[1] A solution of *N*-hydroxypicolinamide in Et_2O (0.1 M) was cooled down to 0 °C. To the reaction mixture were added acyl chloride (1 equiv) followed by NEt_3 (1 equiv). The reaction mixture was warmed to room temperature and stirred for 12 h. The reaction mixture was quenched by adding water and extracted with Et_2O for three times. The organic layer was dried over $MgSO_4$ and concentrated under reduced pressure. The desired amide product was obtained via recrystallization in CH_2Cl_2/*n*-pentane or via silica column chromatography (*n*-Hexanes to *n*-Hexanes/EtOAc = 1:1).

***N*-[{3,5-Bis(trifluoromethyl)benzoyloxy}picolinamide] (1a)**

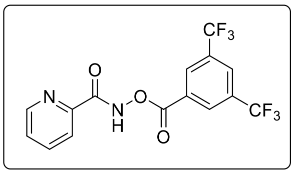

Colorless solid (10 mmol scale, 2.86 g, 76%); **m.p.** 135–137 °C; **^{1}H NMR** (500 MHz, CD_2Cl_2) δ 11.20 (br s, 1H), 8.65 – 8.59 (m, 3H), 8.20 (s, 1H), 8.15 (d, *J* = 7.8 Hz, 1H), 7.94 (td, *J* = 7.8, 1.7 Hz, 1H), 7.56 (dd, *J* = 7.1, 5.4 Hz, 1H); **^{13}C NMR** (150 MHz, CD_2Cl_2) δ 162.8, 162.7, 149.1, 148.5, 138.2, 132.8 (q, *J* = 34.4 Hz), 130.9 – 130.5 (m), 129.7, 128.1 – 128.0 (m), 128.0, 123.2 (q, *J* = 272.8 Hz), 123.2; **^{19}F NMR** (471 MHz, CD_2Cl_2) δ -63.4; **IR** (cm^{-1}) 3326; 1754; 1706; 1618; 1421; 1271; 1173; 997; 840; 697; **HRMS** (ESI) *m/z* calcd. for $C_{15}H_8F_6N_2NaO_3^+$ [M+Na]$^+$: 401.0337, found: 401.0353.

^{15}*N*-[{3,5-Bis(trifluoromethyl)benzoyloxy}picolinamide] (^{15}N-1a)

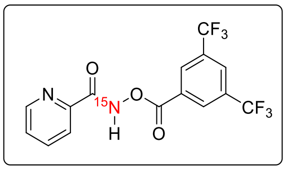

Colorless solid (1.0 mmol scale, 254 mg, 70%); **m.p.** 132–134 °C; **^{1}H NMR** (600 MHz, CD_2Cl_2) δ 11.11 (d, *J* = 45.7 Hz, 1H), 8.63 (d, *J* = 3.8 Hz, 3H), 8.20 (s, 1H), 8.16 (d, *J* = 7.8 Hz, 1H), 7.94 (td, *J* = 7.8, 1.8 Hz, 1H), 7.57 (ddd, *J* = 7.8, 4.8, 1.3 Hz, 1H); **^{13}C NMR** (150 MHz, CD_2Cl_2) δ 162.8, 162.6 (d, *J* = 11.0 Hz), 149.1, 148.4 (d, *J* = 10.4 Hz), 138.2, 132.8 (q, *J* = 34.4 Hz), 130.7 (q, *J* = 4.0 Hz) 129.7, 128.1 – 128.0 (m), 128.0, 123.2 (q, *J* = 272.8 Hz), 123.2; **^{19}F NMR** (564 MHz, CD_2Cl_2) δ -63.4; **IR** (cm^{-1}) 3320; 1754; 1707; 1619; 1414; 1379; 1272; 1173; 1127; 997; 840; 693; **HRMS** (ESI) *m/z* calcd. for $C_{15}H_8F_6{}^{15}NNNaO_3^+$ [M+Na]$^+$: 380.0482, found: 380.0500.

***N*-(Benzoyloxy)picolinamide (1b)**

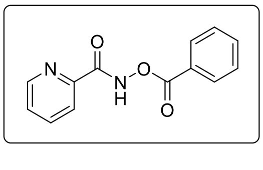

Colorless solid (2.0 mmol scale, 397 mg, 82%); **m.p.** 154–156 °C; **^{1}H NMR** (600 MHz, $CDCl_3$) δ 11.15 (s, 1H), 8.61 (d, *J* = 4.6 Hz, 1H), 8.22 – 8.14 (m, 3H), 7.90 (t, *J* = 7.7 Hz, 1H), 7.65 (t, *J* = 7.6 Hz, 1H), 7.56 – 7.45 (m, 3H); **^{13}C**

NMR (150 MHz, $CDCl_3$) δ 164.7, 161.0, 148.7, 148.6, 137.7, 134.4, 130.3, 128.9, 127.3, 126.9, 122.9; **IR** (cm^{-1}) 3162; 1768; 1668; 1462; 1314; 1234; 1140; 994; 848; 700; **HRMS** (ESI) *m/z* calcd. for $C_{13}H_{11}N_2O_3^+$ $[M+H]^+$: 243.0764, found: 243.0771.

***N*-Acetoxypicolinamide (1c)**

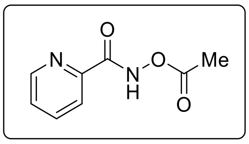

Colorless solid (5.0 mmol scale, 500 mg, 55%); **m.p.** 95–97 °C; **^{1}H NMR** (600 MHz, $CDCl_3$) δ 10.92 (s, 1H), 8.56 (ddd, *J* = 4.7, 1.7, 0.9 Hz, 1H), 8.15 (dt, *J* = 7.7, 1.1 Hz, 1H), 7.88 (td, *J* = 7.7, 1.7 Hz, 1H), 7.51 – 7.43 (m, 1H), 2.30 (s, 3H); **^{13}C NMR** (150 MHz, $CDCl_3$) δ 168.5, 161.7, 148.6, 148.4, 137.6, 127.3, 122.9, 18.5; **IR** (cm^{-1}) 3098; 2902; 1783; 1690; 1493; 1435; 1305; 1244; 1091; 1019; 1003; 854; 749; **HRMS** (ESI) *m/z* calcd. for $C_8H_9N_2O_3^+$ $[M+H]^+$: 181.0608, found: 181.0610.

***N*-(Pivaloyloxy)picolinamide (1d)**

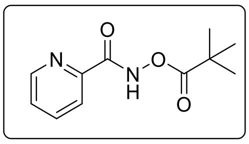

Colorless solid (2.0 mmol scale, 144 mg, 65%); **m.p.** 99–101 °C; **^{1}H NMR** (400 MHz, $CDCl_3$) δ 10.85 (br s, 1H), 8.62 – 8.50 (m, 1H), 8.13 (dd, *J* = 7.9, 1.3 Hz, 1H), 7.92 – 7.78 (m, 1H), 7.46 (ddd, *J* = 7.8, 4.8, 1.5 Hz, 1H), 1.36 (d, *J* = 2.1 Hz, 9H); **^{13}C NMR** (150 MHz, $CDCl_3$) δ 176.3, 162.0, 148.6, 148.6, 137.6, 127.2, 122.8, 38.6, 27.2; **IR** (cm^{-1}) 3083; 2973; 1722; 1687; 1590; 1565; 1502; 1468; 1363; 1286; 1078; 876; 753; 700; 625; **HRMS** (ESI) *m/z* calcd. for $C_{11}H_{15}N_2O_3^+$ $[M+H]^+$: 223.1077, found: 223.1081.

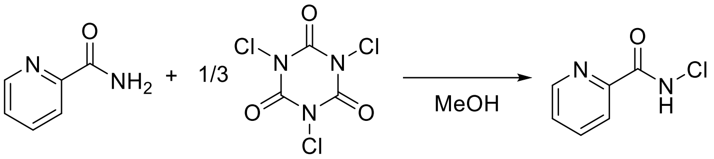

Preparation of N-chloropicolimamide. To a solution of picolinamide (6.00 mmol, 733 mg) in methanol (5 mL) was added trichloroisocyanuric acid (2.00 mmol, 465 mg). The reaction mixture was stirred for 1 h, and undissolved solid was removed by filtration, and the solvent was removed under reduced pressure. *N*-Chloropicolimamide was obtained by silica column chromatography (*n*-Hexane to *n*-Hexane/EtOAc/MeOH = 10:10:1).

***N*-Chloropicolinamide (1e)**

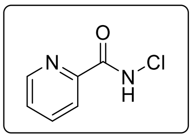

Colorless solid (782 mg, 83%); **m.p.** 66–68 °C; **^{1}H NMR** (600 MHz, $CDCl_3$) δ 8.77 (br s, 1H), 8.53 (d, *J* = 5.0 Hz, 1H), 8.17 (d, *J* = 7.9 Hz, 1H), 7.87 (td, *J* = 7.8, 1.5 Hz, 1H), 7.48 (ddd, *J* = 7.3, 5.4, 1.1 Hz, 1H); **^{13}C NMR** (150 MHz, $CDCl_3$) δ 163.3, 148.9, 148.5, 137.8, 127.1, 122.8; **IR** (cm^{-1}) 3100; 3065; 1668; 1583; 1445; 1395; 1230; 1092; 894; 681; **HRMS** (ESI) *m/z* calcd. for $C_6H_6ClN_2O^+$ $[M+H]^+$: 157.0163, found: 157.0168.

3. Procedures for the synthesis of rhodium complexes

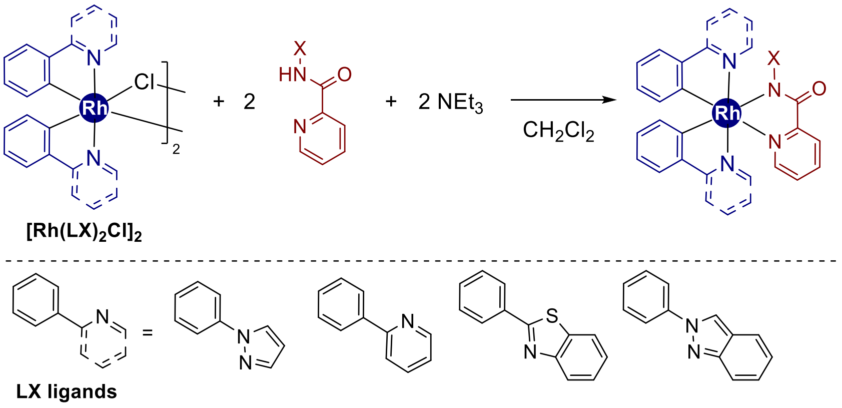

Preparation of rhodium-hydroxamate and chloroamido complexes. **$[Rh(LX)_2Cl]_2$** was synthesized based on the reported procedure using $RhCl_3{\cdot}xH_2O$ and corresponding LX type ligands.[1] Listed LX-type ligands were purchased from TCI or Merck chemical companies and used without further purifications: 1-phenyl-*1H*-pyrazole (PzPh), 2-phenylpyridine (PhPy), 2-phenylbenzo[*d*]thiazole (PhTz). 2-Phenyl-*2H*-indazole (PhInd) was prepared based on the reported procedure.[2] Then, to a solution of *N*-acyloxyamide or *N*-chloropicolinamide (0.1 mmol scale, 2 equiv) and **$[Rh(LX)_2Cl]_2$** (0.05 mmol, 1 equiv) in CH_2Cl_2 (0.01 M) was added NEt_3 (1.0 equiv). The reaction mixture was stirred at 25 °C for 12 h in the dark, and the reaction mixture was washed with $NaHCO_3$ (*aq*). The reaction mixture was concentrated under reduced pressure and the residue was purified by recrystallization in CH_2Cl_2/*n*-pentane or by silica chromatography (CH_2Cl_2 to CH_2Cl_2/MeOH = 10:1) to obtain corresponding rhodium complexes.

$[(PzPh)_2Rh(1a)]$, Rh1a

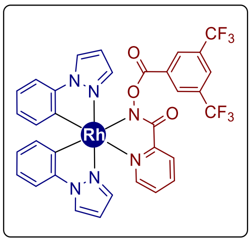

Synthesized from **$[Rh(PzPh)_2Cl]_2$** (0.1 mmol) and **1a** (0.2 mmol). Colorless solid (146 mg, 95%); **m.p.** 254–256 °C (decomp.); **^{1}H NMR** (400 MHz, CD_2Cl_2) δ 8.41 (d, *J* = 2.2 Hz, 1H), 8.29 (d, *J* = 9.0 Hz, 1H), 8.19 (d, *J* = 2.8 Hz, 1H), 8.14 (d, *J* = 2.8 Hz, 1H), 8.02 – 7.93 (m, 2H), 7.92 (s, 2H), 7.88 (d, *J* = 6.1 Hz, 1H), 7.37 – 7.31 (m, 1H), 7.30 (dd, *J* = 7.6, 1.3 Hz, 1H), 7.12 (d, *J* = 7.9 Hz, 1H), 7.08 (td, *J* = 7.6, 1.3 Hz, 1H), 7.03 (d, *J* = 2.2 Hz, 1H), 6.88 (td, *J* = 7.4, 1.2 Hz, 1H), 6.68 (td, *J* = 7.6, 1.3 Hz, 1H), 6.60 (t, *J* = 2.5 Hz, 1H), 6.57 (d, *J* = 2.6 Hz, 1H), 6.45 (t, *J* = 7.7 Hz, 2H), 6.18 (dt, *J* = 7.5, 1.3 Hz, 1H); **^{13}C NMR** (150 MHz, CD_2Cl_2) δ 166.8, 164.1, 155.4, 150.4 (d, *J* = 31.2 Hz), 149.5, 148.6 (d, *J* = 31.9 Hz), 143.5, 142.9, 141.1, 139.1, 138.5, 135.2, 134.9, 132.4, 131.6 (q, *J* = 33.6 Hz) 129.7 (q, *J* = 4.0 Hz), 126.7, 126.3 (d, *J* = 1.5 Hz), 126.2, 126.12 – 125.93 (m), 126.0, 125.6 (d, *J* = 1.7 Hz), 125.5, 123.9, 123.4 (q, *J* = 272.8 Hz), 123.4, 112.0, 111.5, 108.6, 108.3; **^{19}F NMR** (471 MHz, CD_2Cl_2) δ -63.1; **IR** (cm^{-1}) 3099; 1748; 1635; 1600; 1479; 1383; 1280; 1175; 1026; 906; 781; 741; 695; **HRMS** (ESI) *m/z* calcd. $C_{33}H_{21}F_6N_6NaO_3Rh^+$ $[M+Na]^+$: 789.0527, found: 789.0540.

[(PzPh)$_2$Rh(^{15}N-1a)], ^{15}N-Rh1a

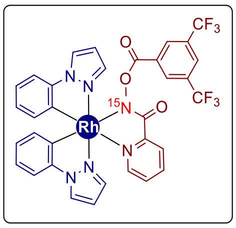

Synthesized from **[Rh(PzPh)$_2$Cl]$_2$** (0.1 mmol) and **^{15}N-1a** (0.2 mmol). Colorless solid (146 mg, 95%); **m.p.** 251–253 °C (decomp.); **^{1}H NMR** (400 MHz, CD_2Cl_2) δ 8.43 (s, 1H), 8.31 (d, *J* = 7.8 Hz, 1H), 8.21 (d, *J* = 2.9 Hz, 1H), 8.16 (s, 1H), 8.02 – 7.92 (m, 4H), 7.90 (d, *J* = 5.1 Hz, 1H), 7.38 – 7.25 (m, 2H), 7.14 (d, *J* = 7.9 Hz, 1H), 7.08 (t, *J* = 7.2 Hz, 1H), 7.04 (d, *J* = 2.2 Hz, 1H), 6.89 (t, *J* = 7.4 Hz, 1H), 6.69 (t, *J* = 7.6 Hz, 1H), 6.61 (d, *J* = 2.6 Hz, 1H), 6.57 (t, *J* = 2.6 Hz, 1H), 6.46 (t, *J* = 7.5 Hz, 2H), 6.20 (d, *J* = 7.5 Hz, 1H); **^{13}C NMR** (100 MHz, CD_2Cl_2) δ 166.7 (d, *J* = 3.6 Hz), 164.1, 155.4 (d, *J* = 15.6 Hz), 150.4 (dd, *J* = 31.2, 16.7 Hz), 149.5, 148.6 (d, *J* = 31.6 Hz), 143.4, 142.9, 141.1, 139.0, 138.5, 135.2, 134.9, 132.3, 131.6 (q, *J* = 33.8 Hz), 129.7 (q, *J* = 3.3 Hz), 126.7, 126.3, 126.2, 126.1 – 126.0 (m), 126.0, 125.6, 125.6, 125.5, 125.4, 123.9, 123.5 (q, *J* = 273.7 Hz), 123.4, 119.4, 111.4, 108.5, 108.3; **^{19}F NMR** (377 MHz, CD_2Cl_2) δ -63.2; **IR** (cm^{-1}) 3096; 1743; 1632; 1596; 1409; 1337; 1174; 1129; 907; 744; **HRMS** (ESI) *m/z* calcd. for $C_{33}H_{21}F_6N_5{}^{15}NNaO_3Rh^+$ [M+Na]$^+$: 790.0497, found: 790.0506.

[(PzPh)$_2$Rh(1b)], Rh1b

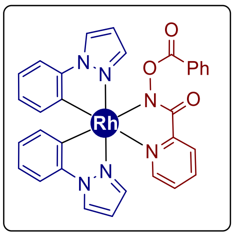

Synthesized from **[Rh(PzPh)$_2$Cl]$_2$** and **1c** (0.1 mmol). Colorless solid (50.0 mg, 79%); **m.p.** 257–259 °C (decomp.); **^{1}H NMR** (500MHz, CD_2Cl_2) δ 8.48 (s, 1H), 8.30 (d, *J* = 7.8 Hz, 1H), 8.20 (d, *J* = 2.8 Hz, 1H), 8.13 (d, *J* = 2.8 Hz, 1H), 7.95 (td, *J* = 7.7, 1.7 Hz, 1H), 7.87 (d, *J* = 4.4 Hz, 1H), 7.48 – 7.40 (m, 3H), 7.33 – 7.28 (m, 2H), 7.28 – 7.23 (m, 2H), 7.19 (dd, *J* = 7.9, 1.3 Hz, 1H), 7.07 (td, *J* = 7.6, 1.3 Hz, 1H), 7.00 (d, *J* = 2.2 Hz, 1H), 6.88 (td, *J* = 7.4, 1.2 Hz, 1H), 6.80 (dd, *J* = 7.6, 1.3 Hz, 1H), 6.58 (t, *J* = 2.5 Hz, 1H), 6.56 (t, *J* = 2.5 Hz, 1H), 6.53 (td, *J* = 7.5, 1.2 Hz, 1H), 6.42 (d, *J* = 7.5 Hz, 1H), 6.20 (d, *J* = 7.6 Hz, 1H); **^{13}C NMR** (125 MHz, CD_2Cl_2) δ 166.6, 166.4, 155.8, 151.0 (d, *J* = 30.9 Hz), 149.4, 148.9 (d, *J* = 31.7 Hz), 143.5, 142.9, 141.3, 138.9, 138.3, 135.2, 134.8, 132.5, 130.3, 129.4, 128.2, 126.4, 126.2(1), 126.2(0), 126.0, 125.9, 125.6, 125.4, 123.8, 123.2, 111.8, 111.4, 108.3 (2C); **IR** (cm^{-1}) 3086; 1730; 1627; 1564; 1478; 1408; 1281; 1175; 1023; 747; 702; **HRMS** (ESI) *m/z* calcd. for $C_{31}H_{24}N_6O_3Rh^+$ [M+H]$^+$: 631.0959, found: 631.0967.

[(PzPh)$_2$Rh(1c)], Rh1c

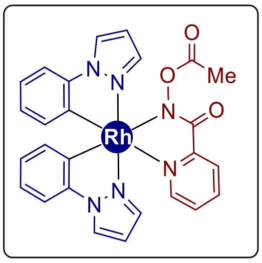

Synthesized from **[Rh(PzPh)$_2$Cl]$_2$** and **1c** (0.1 mmol). Colorless solid (31.5 mg, 55%); **m.p.** 265–267 °C (decomp.); **^{1}H NMR** (500 MHz, CD_2Cl_2) δ 8.33 (s, 1H), 8.25 (d, *J* = 7.8 Hz, 1H), 8.18 (d, *J* = 2.8 Hz, 1H), 8.13 (d, *J* = 2.8 Hz, 1H), 7.92 (td, *J* = 7.8, 1.7 Hz, 1H), 7.84 (d, *J* = 4.5 Hz, 1H), 7.29 (d, *J* = 9.1 Hz, 1H), 7.28 – 7.25 (m, 1H), 7.24 (dd, *J* = 7.9, 1.3 Hz, 1H), 7.06 (td, *J* = 7.6, 1.3 Hz, 1H), 6.99 (td, *J* = 7.6, 1.3 Hz, 1H), 6.95 (d, *J* = 2.2 Hz, 1H), 6.87 (td, *J* = 7.4, 1.2 Hz, 1H), 6.77 (td, *J* = 7.5, 1.2 Hz, 1H), 6.53 (dt, *J* = 6.8, 2.5 Hz, 2H), 6.41 (d, *J* = 7.5 Hz, 1H), 6.27 (d, *J* =

7.5 Hz, 1H), 1.54 (s, 3H); **^{13}C NMR** (125 MHz, CD_2Cl_2) δ 169.8, 166.3, 155.8, 151.0 (d, *J* = 30.8 Hz), 149.3, 149.1 (d, *J* = 31.7 Hz), 143.4, 143.0, 141.2, 138.8, 138.2, 135.2, 134.9, 126.3, 126.2, 126.0, 125.9, 125.6, 125.3, 123.8, 123.2, 111.8, 111.5, 108.2, 108.2, 18.8; **IR** (cm^{-1}) 3120; 1754; 1625; 1596; 1477; ;1339; 1201; 1074; 933; 748; **HRMS** (ESI) *m/z* calcd. for $C_{26}H_{22}N_2O_3Rh^+$ [M+H]$^+$: 569.0803, found: 569.0809.

[(PzPh)$_2$Rh(1d)], Rh1d

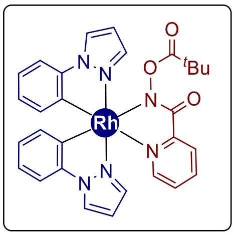

Synthesized from **[Rh(PzPh)$_2$Cl]$_2$** and **1d** (0.2 mmol). Colorless solid (104 mg, 81%); **m.p.** 253–255 °C (decomp.); **^{1}H NMR** (400 MHz, CD_2Cl_2) δ 8.44 (s, 1H), 8.24 (d, *J* = 10.3 Hz, 1H), 8.18 (d, *J* = 2.9 Hz, 1H), 8.12 (d, *J* = 2.8 Hz, 1H), 7.91 (td, *J* = 7.7, 1.7 Hz, 1H), 7.81 (d, *J* = 5.4 Hz, 1H), 7.35 – 7.17 (m, 3H), 7.06 (td, *J* = 7.6, 1.4 Hz, 1H), 7.01 – 6.91 (m, 2H), 6.86 (td, *J* = 7.3, 1.2 Hz, 1H), 6.73 (td, *J* = 7.5, 1.3 Hz, 1H), 6.54 (dt, *J* = 7.7, 2.6 Hz, 2H), 6.39 (d, *J* = 7.5 Hz, 1H), 6.24 (d, *J* = 7.6 Hz, 1H), 0.84 (s, 9H); **^{13}C NMR** (100 MHz, CD_2Cl_2) δ 177.2, 166.1, 155.9, 151.3 (d, *J* = 30.8 Hz), 149.2, 149.1 (d, *J* = 30.1 Hz), 143.4, 142.9, 141.3, 138.8, 138.3, 135.2, 134.9, 126.2, 126.2, 125.9, 125.8 (2C), 125.2, 123.7, 123.2, 111.8, 111.5, 108.2 (2C), 38.3, 27.2; **IR** (cm^{-1}) 3120; 1739; 1632; 1597; 1478; 1410; 1339; 1117; 1026; 746; **HRMS** (ESI) *m/z* calcd. for $C_{31}H_{24}N_6O_3Rh^+$ [M+H]$^+$: 631.0959, found: 631.0967.

[(PzPh)$_2$Rh(1e)], Rh1e

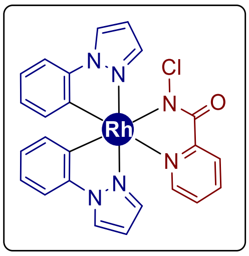

Synthesized from **[Rh(PzPh)$_2$Cl]$_2$** and **1d** (0.1 mmol). Colorless solid (41.0 mg, 75%); **m.p.** 309–311 °C (decomp.); **^{1}H NMR** (600 MHz, CD_2Cl_2) δ 8.26 (d, *J* = 7.8 Hz, 1H), 8.19 (s, 1H), 8.14 (s, 1H), 7.93 (t, *J* = 7.8 Hz, 1H), 7.88 (d, *J* = 5.2 Hz, 1H), 7.75 (s, 1H), 7.28 (d, *J* = 7.4 Hz, 3H), 7.07 (t, *J* = 7.6 Hz, 1H), 7.03 (t, *J* = 7.6 Hz, 1H), 6.88 (t, *J* = 7.5 Hz, 1H), 6.83 (t, *J* = 5.3 Hz, 2H), 6.57 (s, 1H), 6.52 (s, 1H), 6.37 (d, *J* = 7.5 Hz, 1H), 6.24 (d, *J* = 7.5 Hz, 1H); **^{13}C NMR** (125 MHz, CD_2Cl_2) δ 169.3, 154.9, 150.1 (d, *J* = 31.3 Hz), 149.3, 143.3, 142.6, 139.6, 139.0, 138.3, 135.1, 134.8, 126.5, 126.3(4), 126.2(7), 126.2, 126.1, 125.4, 123.8, 123.4, 112.0, 111.7, 108.5, 108.3; **IR** (cm^{-1}) 3076; 1618; 1590; 1477; 1326; 1281; 1072; 748; **HRMS** (ESI) *m/z* calcd. for $C_{24}H_{19}ClN_6ORh^+$ [M+H]$^+$: 545.0358, found: 545.0367.

[(CF$_3$PzPh)$_2$Rh(1a)], Rh1f

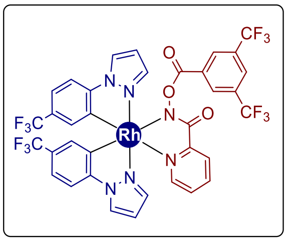

Synthesized from **[Rh(CF$_3$PzPh)$_2$Cl]$_2$** (0.05 mmol) and **1a** (0.1 mmol). Colorless solid (56.3 mg, 62%); **m.p.** 248–250 °C (decomp.); **^{1}H NMR** (400 MHz, CD_2Cl_2) δ 8.55 (s, 1H), 8.39 – 8.33 (m, 2H), 8.30 (d, *J* = 2.9 Hz, 1H), 8.01 (td, *J* = 7.7, 1.6 Hz, 1H), 7.98 (s, 1H), 7.96 (s, 2H), 7.84 (d, *J* = 5.3 Hz, 1H), 7.46 (d, *J* = 8.3 Hz, 1H), 7.40 – 7.32 (m, 2H), 7.28 (d, *J* = 8.3 Hz, 1H), 7.12 (d, *J* = 2.3 Hz, 1H), 6.94 (d, *J* = 8.3 Hz, 1H), 6.67 (t, *J* = 2.6 Hz, 1H), 6.63 (dd, *J* = 6.1, 3.5 Hz,

2H), 6.45 (s, 1H); **^{13}C NMR** (150 MHz, CD_2Cl_2) δ 166.7, 163.8, 155.1, 150.7 (d, *J* = 31.8 Hz), 150.5, 149.1 (d, *J* = 32.4 Hz), 149.0, 146.0, 145.5, 142.4, 139.8, 139.6, 131.9 (q, *J* = 33.8 Hz), 131.4 (q, *J* = 3.5 Hz), 131.2 (q, *J* = 4.0 Hz), 129.6 (q, *J* = 4.0 Hz), 127.8 (q, *J* = 31.3 Hz), 127.6 – 127.5 (m), 127.5 – 127.3 (m), 127.1, 127.1 (q, *J* = 31.8 Hz), 126.5 – 126.2 (m), 125.9, 124.6 (q, *J* = 272.8 Hz), 124.1 (q, *J* = 272.7 Hz), 123.4 (q, *J* = 272.5 Hz), 122.0 (q, *J* = 3.8 Hz), 121.2 (q, *J* = 4.0 Hz), 112.0, 111.5, 109.7, 109.4; **^{19}F NMR** (377 MHz, CD_2Cl_2) δ -62.3, -63.0, -63.3; **IR** (cm^{-1}) 3090; 1745; 1642; 1599; 1343; 1276; 1113; 1053; **HRMS** (ESI) *m/z* calcd. for $C_{35}H_{20}F_{12}N_6O_3Rh^+$ $[M+H]^+$: 903.0455, found: 903.0466.

[(PhPy)$_2$Rh(1a)], Rh3

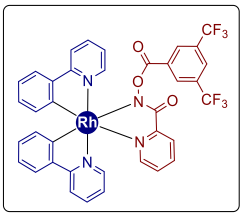

Synthesized from **[Rh(PhPy)$_2$Cl]$_2$** and **1a** (0.1 mmol). Pale yellow solid (52.6 mg, 67%); **m.p.** 246–248 °C (decomp.); **^{1}H NMR** (600 MHz, CD_2Cl_2) δ 9.49 (d, *J* = 6.0 Hz, 1H), 8.31 (d, *J* = 7.9 Hz, 1H), 7.97 – 7.91 (m, 4H), 7.89 (d, *J* = 4.3 Hz, 3H), 7.83 (t, *J* = 7.7 Hz, 1H), 7.79 – 7.70 (m, 3H), 7.44 (d, *J* = 7.5 Hz, 1H), 7.31 – 7.26 (m, 1H), 7.24 (t, *J* = 6.6 Hz, 1H), 7.13 (td, *J* = 5.9, 2.7 Hz, 1H), 7.06 (t, *J* = 7.5 Hz, 1H), 6.92 (t, *J* = 7.4 Hz, 1H), 6.59 (dq, *J* = 14.4, 7.3 Hz, 2H), 6.45 (d, *J* = 7.6 Hz, 1H), 6.24 (d, *J* = 7.5 Hz, 1H); **^{13}C NMR** (150 MHz, CD_2Cl_2) δ 169.8 (d, *J* = 32.4 Hz), 167.9 (d, *J* = 32.9 Hz),167.2, 165.7, 165.1, 164.1, 155.1, 151.8, 149.5, 148.9, 145.0, 144.2, 138.9, 138.0, 137.8, 133.9, 133.7, 132.2, 131.5 (q, *J* = 33.8 Hz), 129.8 (q, *J* = 3.5 Hz), 129.7, 129.0 (d, *J* = 1.7 Hz), 127.0, 126.1 – 125.9 (m),125.6, 124.4, 123.7, 123.4 (q, *J* = 272.9 Hz), 123.2, 123.2, 123.1, 122.7, 119.2 (d, *J* = 1.7 Hz), 119.2 (d, *J* = 1.7 Hz); **^{19}F NMR** (377 MHz, CD_2Cl_2) δ -63.1; **IR** (cm^{-1}) 3038; 1738; 1642; 1600; 1579; 1382; 1179; 1024; 921; 734; **HRMS** (ESI) *m/z* calcd. for $C_{37}H_{24}F_6N_4O_3Rh^+$ $[M+H]^+$: 789.0802, found: 789.0808.

[(PhTh)$_2$Rh(1a)], Rh4

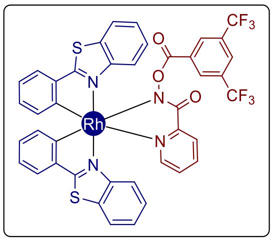

Synthesized from **[Rh(PhTh)$_2$Cl]$_2$** and **1a** (0.1 mmol). Yellow solid (74.3 mg, 82%); **m.p.** 215–217 °C (decomp.); **^{1}H NMR** (600 MHz, CD_2Cl_2) δ 8.87 (d, *J* = 8.7 Hz, 1H), 8.22 (d, *J* = 7.8 Hz, 1H), 8.01 – 7.97 (m, 5H), 7.93 (ddd, *J* = 15.5, 7.8, 1.4 Hz, 2H), 7.86 (dd, *J* = 7.7, 1.3 Hz, 1H), 7.58 (ddd, *J* = 8.5, 7.2, 1.3 Hz, 1H), 7.45 (ddd, *J* = 8.3, 7.2, 1.1 Hz, 1H), 7.42 – 7.38 (m, 2H), 7.38 – 7.34 (m, 1H), 7.16 – 7.09 (m, 2H), 6.94 (td, *J* = 7.5, 1.4 Hz, 1H), 6.77 – 6.69 (m, 2H), 6.62 (d, *J* = 7.7 Hz, 1H), 6.48 – 6.44 (m, 1H), 6.38 (d, *J* = 8.4 Hz, 1H); **^{13}C NMR** (150 MHz, CD_2Cl_2) δ 176.8 (d, *J* = 3.3 Hz), 176.5 (d, *J* = 3.4 Hz), 169.3 (d, *J* = 30.1 Hz), 167.5 (d, *J* = 30.6 Hz),167.2, 163.1, 155.8, 150.1, 150.0, 149.2, 141.2, 140.5, 139.3, 135.1, 134.5, 132.5, 132.4, 132.0, 131.7 (q, *J* = 33.5 Hz), 130.8, 130.3, 129.8 – 129.6 (m), 128.3, 127.7, 127.2, 126.5, 126.3, 126.2 – 126.0 (m), 125.8, 125.7, 125.5, 123.9, 123.6, 123.5 (q, *J* = 272.6 Hz), 123.4, 122.8, 122.2, 119.1; **^{19}F NMR** (377 MHz, CD_2Cl_2) δ -63.1; **IR** (cm^{-1}) 3055; 1745; 1642; 1597; 1475; 1413; 1382; 1321; 1223; 1130; 1021; 908; 751; **HRMS** (ESI) *m/z* calcd. for $C_{41}H_{24}F_6N_4O_3RhS_2^+$ $[M+H]^+$: 901.0244, found: 901.0256.

[(PhInd)$_2$Rh(1a)], Rh5

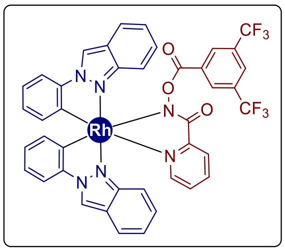

Synthesized from **[Rh(PhInd)$_2$Cl]$_2$** and **1a** (0.1 mmol). Brown solid (82.7 mg, 95%); **m.p.** 257–259 °C (decomp.); **^{1}H NMR** (600 MHz, CD_2Cl_2) δ 8.77 (s, 1H), 8.69 (s, 1H), 8.29 (dd, J = 8.4, 3.7 Hz, 2H), 8.03 – 8.00 (m, 1H), 8.00 – 7.96 (m, 1H), 7.94 (s, 3H), 7.85 (d, J = 8.5 Hz, 1H), 7.79 (d, J = 8.5 Hz, 1H), 7.63 (d, J = 7.0 Hz, 1H), 7.49 (dd, J = 8.8, 6.6 Hz, 1H), 7.36 (ddd, J = 7.2, 5.3, 1.4 Hz, 1H), 7.28 – 7.21 (m, 2H), 7.20 – 7.11 (m, 2H), 7.11 – 7.04 (m, 1H), 6.86 (t, J = 8.0 Hz, 1H), 6.71 – 6.64 (m, 1H), 6.53 (t, J = 8.0 Hz, 1H), 6.35 (d, J = 7.6 Hz, 1H), 6.09 (d, J = 7.6 Hz, 1H), 5.91 (d, J = 8.8 Hz, 1H); **^{13}C NMR** (125 MHz, CD_2Cl_2) δ 167.1, 163.1, 155.8, 152.8 (d, J = 31.3 Hz), 151.0 (d, J = 30.8 Hz), 149.9, 147.8, 147.4, 143.2, 142.7, 139.4, 135.9, 135.3, 132.3, 131.6 (q, J = 33.8 Hz), 129.8, 129.7 – 129.5 (m), 129.3, 127.7, 126.9(3), 126.8(9), 126.1 – 125.9 (m), 125.8, 124.2, 123.8, 123.6, 123.4 (q, J = 272.3 Hz), 123.2, 123.0, 122.9, 122.1, 121.4, 120.3, 119.5, 116.2, 113.9, 113.7, 113.2; **^{19}F NMR** (377 MHz, CD_2Cl_2) δ -63.2; **IR** (cm^{-1}) 3062; 1735; 1639; 1597; 1453; 1184; 1075; 1024; 978; 751; **HRMS** (ESI) *m/z* calcd. for $C_{41}H_{26}F_6N_6O_3Rh^+$ $[M+H]^+$: 867.1020, found: 867.1028.

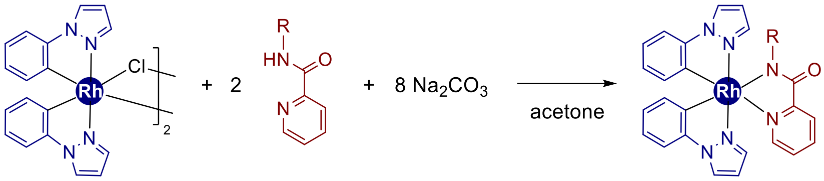

Preparation of product complexes. To a solution of picolinamide derivatives (0.1 mmol, 2 equiv) and **[Rh(PzPh)$_2$Cl]$_2$** (0.05 mmol, 1 equiv, 42.4 mg) in acetone (0.01 M) was added Na_2CO_3 (0.4 mmol, 8.0 equiv, 42.4 mg). The reaction mixture was stirred at 25 °C for 12 h in the dark, and the reaction mixture was filtered through a pad of celite and washed with dichloromethane. The crude reaction mixture was concentrated under reduced pressure and the residue was purified by recrystallization in CH_2Cl_2/*n*-pentane or by silica chromatography (CH_2Cl_2 to CH_2Cl_2/MeOH = 10:1) to obtain corresponding rhodium complexes.

[(PzPh)$_2$Rh(2)], Rh2

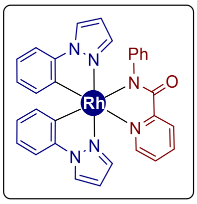

Synthesized from **[Rh(PzPh)$_2$Cl]$_2$** and *N*-phenylpicolinamide[3] (**2**, 0.1 mmol). Pale yellow solid (46.3 mg, 79%); **m.p.** 298–300 °C (decomp.); **^{1}H NMR** (500 MHz, CD_2Cl_2) δ 8.35 (d, *J* = 7.6 Hz, 1H), 8.13 (d, *J* = 2.8 Hz, 1H), 8.11 (d, *J* = 3.6 Hz, 1H), 7.95 – 7.89 (m, 3H), 7.30 – 7.25 (m, 2H), 7.07 – 7.01 (m, 2H), 7.00 (d, *J* = 2.3 Hz, 1H), 6.89 – 6.81 (m, 3H), 6.81 – 6.76 (m, 1H), 6.69 (t, *J* = 7.3 Hz, 1H), 6.58 – 6.51 (m, 5H), 6.41 (d, *J* = 7.4 Hz, 1H), 5.93 (d, *J* = 7.5 Hz, 1H); **^{13}C NMR** (150 MHz, CD_3OD) δ 169.3, 158.3, 152.3 (d, *J* = 30.6 Hz), 151.8 (d, *J* = 31.2 Hz), 150.3, 148.9, 144.3, 143.4, 140.2, 139.8, 139.0, 135.7, 135.0, 128.6, 127.8, 127.7, 127.3, 127.1, 126.5, 126.2, 126.0, 124.4, 123.7, 123.3, 112.9, 112.2, 109.2, 109.2; **IR** (cm^{-1}) 3058; 1607; 1575; 1556; 1476; 743; 690; **HRMS** (ESI) *m/z* calcd. for $C_{30}H_{24}N_6ORh^+$ $[M+H]^+$: 587.1061, found: 587.1068.

[(PzPh)$_2$Rh(1f)], Rh6

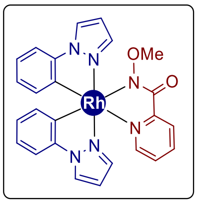

Synthesized from **[Rh(PzPh)$_2$Cl]$_2$** and *N*-methoxypicolinamide[4] (**1f**, 0.1 mmol). Colorless solid (27.2 mg, 50%); **m.p.** 315–317 °C (decomp.); **^{1}H NMR** (600 MHz, CD_2Cl_2) δ 8.25 (d, *J* = 7.9 Hz, 1H), 8.21 (d, *J* = 2.8 Hz, 1H), 8.15 (d, *J* = 2.8 Hz, 1H), 7.89 (t, *J* = 7.8 Hz, 1H), 7.86 (s, 1H), 7.81 (d, *J* = 5.2 Hz, 1H), 7.28 (t, *J* = 7.0 Hz, 2H), 7.22 (t, *J* = 7.1 Hz, 1H), 7.08 – 7.02 (m, 1H), 7.02 – 6.96 (m, 1H), 6.92 – 6.84 (m, 2H), 6.83 – 6.77 (m, 1H), 6.58 – 6.47 (m, 2H), 6.35 (d, *J* = 7.5 Hz, 1H), 6.30 (d, *J* = 7.6 Hz, 1H), 3.09 (s, 3H); **^{13}C NMR** (150 MHz, CD_2Cl_2) δ 167.1, 157.2, 152.3 (d, *J* = 30.6 Hz), 149.7 (d, *J* = 31.2 Hz), 149.0, 143.3, 142.7, 140.3, 138.7, 138.3, 134.8(4), 134.7(8), 126.5, 126.2, 126.0 (2C), 125.8, 125.0, 123.7, 123.1, 111.9, 111.6, 108.4, 108.3, 61.0; **IR** (cm^{-1}) 3077; 1613; 1587; 1561; 1476; 1407; 1337; 1027; 747; 693; **HRMS** (ESI) *m/z* calcd. for $C_{25}H_{22}N_6O_2Rh^+$ $[M+H]^+$: 541.0854, found: 541.0861.

3. Procedures for the photocatalyzed C–H amidation using Rh-hydroxamate catalysts

3-1. Stoichiometric reaction with Rh1a

To a teflon-lined 4 mL vial equipped with a stir bar were added **Rh1a** complex (0.025 mmol, 19.1 mg), anhydrous CH_2Cl_2 (0.5 mL) and benzene (0.5 mL) under N_2 atmosphere. The vial was sealed with a cap, and equipped in a Merck Penn PhD photoreactor M2.[5] The reaction mixture was irradiated for 3 h (365 nm, 100% intensity) and maintained at room temperature. Then, the solvent was evaporated, and the product yield of **Rh2** and **2** was determined using ^1H NMR analysis in the presence of 1,1,2-trichloroethane as an internal standard in CD_2Cl_2. Detailed results are summarized in Scheme S1.

Scheme S1. Stoichiometric C–H Amidation of Benzene with **Rh1a**.

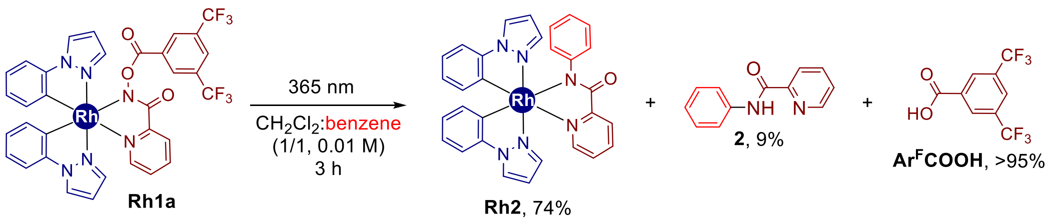

3-2. Stoichiometric regeneration of reactant complex Rh1a from Rh2

To a J-Young NMR tube were added the product complex **Rh2** (0.01 mmol, 5.9 mg), **1a** (0.01 mmol, 3.8 mg), and anhydrous CD_2Cl_2 (0.4 mL). The stock solution of 1,1,2-trichloroethane internal standard solution in CD_2Cl_2 (0.1 M, 0.1 mL) was added to the NMR tube, and the formation of **Rh1a** was monitored by ^1H NMR analysis (exp 3-2A). To another J-Young tube, all components from the exp 3-2A were added, followed by **ArFCOOH** (0.1 mmol. 2.6 mg). Again, the formation of **Rh1a** was monitored by ^1H NMR analysis (exp 3-2B). Detailed results for the reaction monitoring results are summarized in Scheme S2.

Scheme S2. Stoichiometric Regeneration of **Rh1a** from **Rh2**.

Exp 3-2A

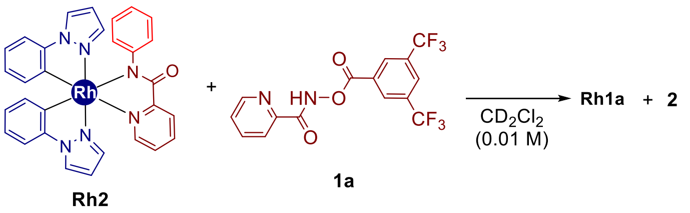

Time	Yield of **Rh1a**	Yield of **Rh2**
10 min	4%	89%
20 min	7%	86%
12 h	78%	11%

Exp 3-2B

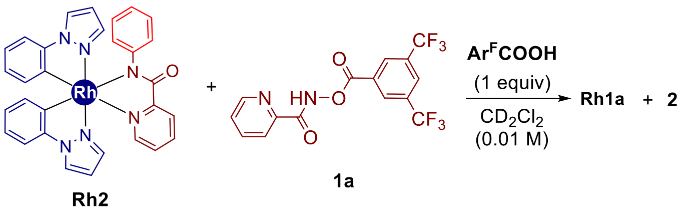

Time	Yield of **Rh1a**	Yield of **Rh2**
10 min	>95%	<5%

3-3. Stoichiometric C–H amidation reactivity with various Rh complexes.

To a teflon-lined 4 mL vial equipped with a stir bar were added Rh-hydroxamate complex (0.025 mmol), anhydrous CH_2Cl_2 (0.5 mL) and benzene (0.5 mL) under N_2 atmosphere. The vial was sealed with a cap, and equipped in a Merck Penn PhD photoreactor M2.[5] The reaction mixture was irradiated for 3 h (365 nm, 100% intensity) and maintained at room temperature. Upon completion, **1a** (0.025 mmol, 9.4 mg) was added as a proton source. Then, the solvent was evaporated, and the product yield of **2** was determined using ^{1}H NMR analysis in the presence of 1,1,2-trichloroethane as an internal standard in CD_2Cl_2. Detailed results are summarized in Scheme S3.

Scheme S3. Stoichiometric C–H Amidation of Various Rh Complexes.

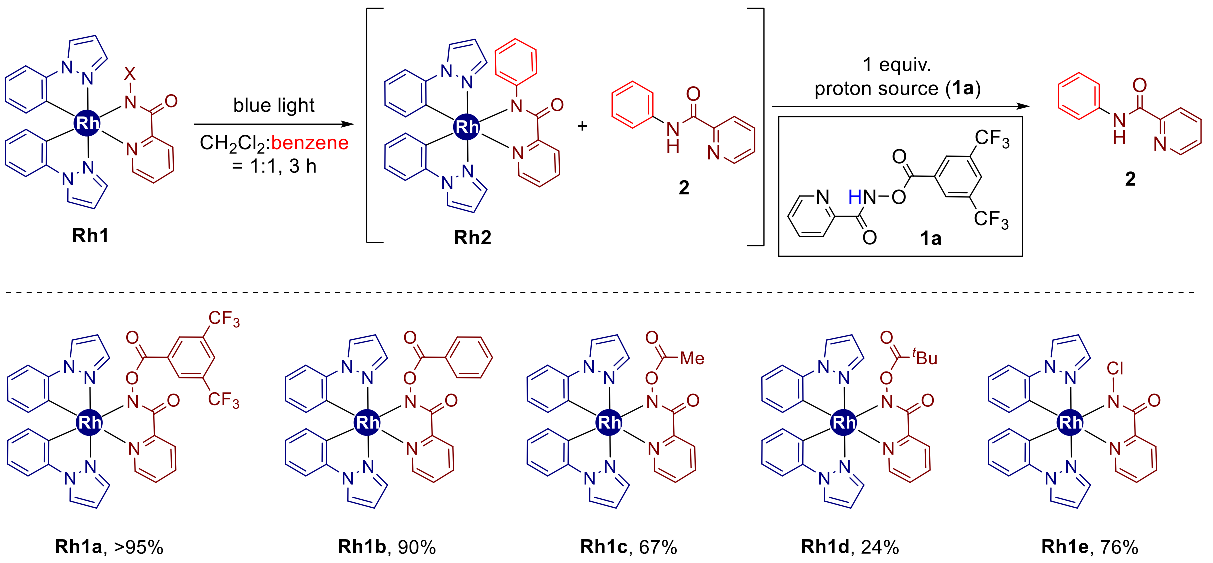

3-2. Optimization of catalytic reaction conditions

To a pre-dried 4 mL teflon-lined vial equipped with a stir bar were added **1a** (0.1 mmol), Rh-hydroxamate complex (0.005 mmol) and anhydrous solvent (1 M, 0.1 mL) under N_2 atmosphere. The vial was sealed with a cap, and equipped in a Merck Penn PhD photoreactor M2.[5] The reaction mixture was irradiated for 12 h (365 nm, 100% intensity) and maintained at room temperature. After completion, the solvent was removed under reduced pressure, and the product yield was determined using ^{1}H NMR analysis in the presence of 1,1,2-trichloroethane as an internal standard. Detailed reaction optimization conditions are shown in Table S1.

Table S1. Optimization of reaction parameters

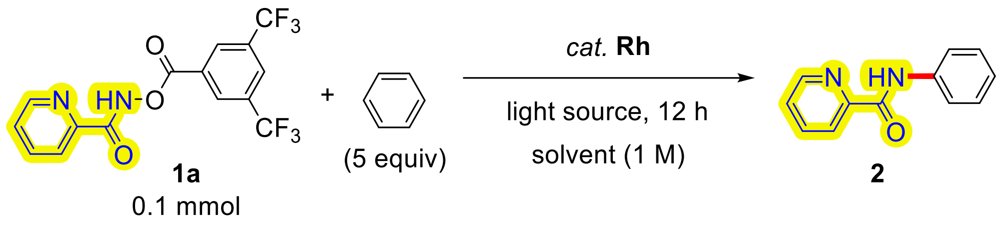

Entry	Catalyst	Light source*	Solvent	2 (%)	Remaining 1a (%)
1	5 mol% **Rh1a**	365 nm	CH_2Cl_2	84%	<5%
2	1 mol% **Rh1a**	365 nm	CH_2Cl_2	19%	68%
3	5 mol% **Rh1a**	365 nm	$CHCl_3$	79%	<5%
4	5 mol% **Rh1a**	365 nm	1,2-DCE	74%	<5%
5	5 mol% **Rh1a**	365 nm	CH_3CN	78%	<5%
6	5 mol% **Rh1a**	365 nm	EtOAc	73%	<5%
7	5 mol% **Rh1a**	395 nm	CH_2Cl_2	63%	<5%
8	5 mol% **Rh1a**	420 nm	CH_2Cl_2	39%	<5%
9	5 mol% **Rh3**	365 nm	CH_2Cl_2	48%	45%
10	5 mol% **Rh4**	365 nm	CH_2Cl_2	11%	77%
11	5 mol% **Rh5**	365 nm	CH_2Cl_2	<5%	92%
12	No catalyst	365 nm	CH_2Cl_2	<5%	>95%
13	5 mol% **Rh1a**	No light	CH_2Cl_2	<5%	>95%
14	5 mol% **Rh3**	420 nm	CH_2Cl_2	16%	49%
15	5 mol% **Rh4**	420 nm	CH_2Cl_2	<5%	68%
16	5 mol% **Rh5**	420 nm	CH_2Cl_2	<5%	95%
17	5 mol% **Rh1f**	420 nm	CH_2Cl_2	20%	64%

*If otherwise noted, Merck Penn PhD Photoreactor M2 (100% intensity) was used as the light source.

3-3. Procedure for the photocatalytic C–H amidations

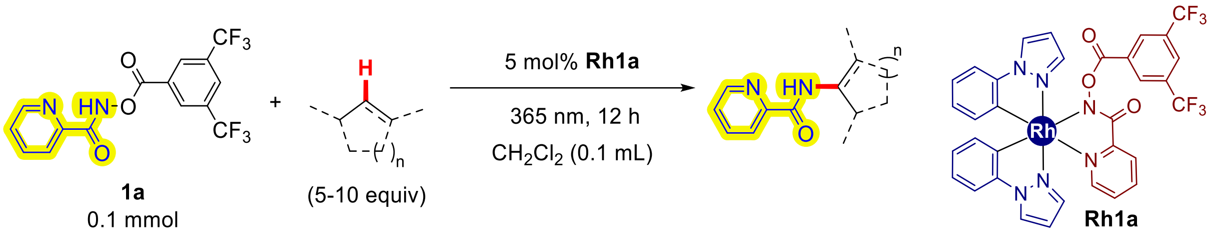

To a pre-dried 4 mL teflon-lined vial equipped with a stir bar were added **Rh1a** (0.005 mmol, 3.8 mg), **1a** (0.1 mmol, 37.8 mg), and anhydrous CH_2Cl_2 (1 M, 0.1 mL) under N_2 atmosphere. Substrate was added, the vial was sealed with a cap, and placed on a stirrer plate. The vial was sealed with a cap, and equipped in a Merck Penn PhD photoreactor M2.[5] The reaction mixture was irradiated for 12 min (365 nm, 100% intensity) and maintained at room temperature. After completion, the solvent was removed under reduced pressure, and the C–H amidation product was isolated by silica chromatography (*n*-hexane to *n*-hexane/EtOAc/MeOH = 20:10:1).

N-Phenylpicolinamide (2)

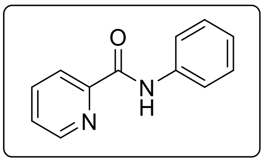

Colorless solid (14.8 mg, 75%); **^{1}H NMR** (600 MHz, $CDCl_3$) δ 10.03 (s, 1H), 8.62 (d, *J* = 4.7 Hz, 1H), 8.31 (d, *J* = 7.8 Hz, 1H), 7.91 (td, *J* = 7.7, 1.7 Hz, 1H), 7.79 (d, *J* = 7.3 Hz, 2H), 7.48 (dd, *J* = 7.0, 4.2 Hz, 1H), 7.40 (d, *J* = 8.6 Hz, 2H), 7.16 (d, *J* = 7.4 Hz, 1H); **^{13}C NMR** (150 MHz, $CDCl_3$) δ 162.1, 150.0, 148.1, 137.9, 137.8, 129.2, 126.6, 124.5, 122.6, 119.8.

The spectroscopic data are consistent with those reported in the literature.[1]

N-Cyclohexylpicolinamide (3)

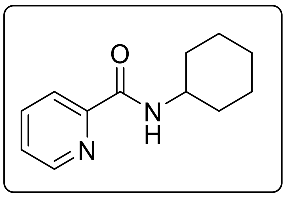

Conducted with 20 mol% of **Rh1a** and 0.1 mL substrate loading. Colorless solid (10.4 mg, 51%); **^{1}H NMR** (500 MHz, $CDCl_3$) δ 8.53 (d, *J* = 4.6 Hz, 1H), 8.20 (d, *J* = 7.9 Hz, 1H), 7.94 (s, 1H), 7.83 (t, *J* = 7.8 Hz, 1H), 7.43 – 7.36 (m, 1H), 4.02 – 3.91 (m, 1H), 2.06 – 1.97 (m, 2H), 1.84 – 1.72 (m, 2H), 1.65 (dd, *J* = 8.9, 4.3 Hz, 1H), 1.49 – 1.39 (m, 2H), 1.35 – 1.22 (m, 3H); **^{13}C NMR** (125 MHz, $CDCl_3$) δ 163.4, 150.4, 148.1, 137.4, 126.1, 122.4, 48.3, 33.2, 25.8, 25.0.

The spectroscopic data are consistent with those reported in the literature.[14]

Mixture of *N*-(*o*-tolyl)picolinamide (5o), *N*-(*m*-tolyl)picolinamide (5m) and *N*-(*p*-tolyl)picolinamide (5p)

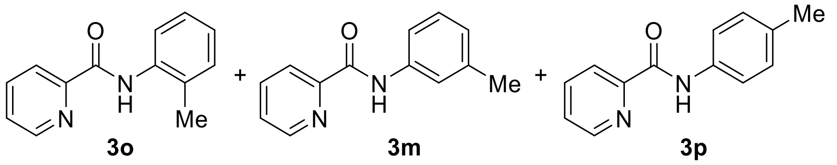

Colorless solid (12.3 mg, 58%).

***N*-(*o*-Tolyl)picolinamide (5o)**, **^{1}H NMR** (500 MHz, $CDCl_3$) δ 10.09 (br s, 1H), 8.66 – 8.58 (m, 1H), 8.34 – 8.30 (m, 1H), 8.30 – 8.28 (m, 1H), 7.94 – 7.87 (m, 1H), 7.50 – 7.44 (m, 1H), 7.31 – 7.26 (m, 1H), 7.23 (d, *J* = 7.5 Hz, 1H), 7.09 (t, *J* = 7.5 Hz, 1H), 2.43 (s, 3H); **^{13}C NMR** (125 MHz, $CDCl_3$) δ 162.0, 150.3, 148.2, 137.8, 136.1, 130.5, 128.1, 127.0, 126.5, 124.7, 122.5, 121.4, 17.9.

The spectroscopic data are consistent with those reported in the literature.[6]

***N*-(*m*-Tolyl)picolinamide (5m)**, **^{1}H NMR** (500 MHz, $CDCl_3$) δ 9.97 (br s, 1H), 8.66 – 8.58 (m, 1H), 8.34 – 8.30 (m, 1H), 7.94 – 7.87 (m, 1H), 7.70 – 7.63 (m, 1H), 7.57 (d, *J* = 6.0 Hz, 1H), 7.50 – 7.44 (m, 1H), 7.31 – 7.26 (m, 1H), 6.97 (d, *J* = 7.6 Hz, 1H), 2.39 (s, 3H); **^{13}C NMR** (125 MHz, $CDCl_3$) δ 162.0, 150.0, 148.1, 139.1, 137.8, 137.8, 129.0, 126.5, 125.3, 122.5, 120.4, 116.9, 21.7.

The spectroscopic data are consistent with those reported in the literature.[7]

***N*-(*p*-Tolyl)picolinamide (5p)**, **^{1}H NMR** (500 MHz, $CDCl_3$) δ 9.97 (br s, 1H), 8.66 – 8.58 (m, 1H), 8.34 – 8.30 (m, 1H), 7.94 – 7.87 (m, 1H), 7.70 – 7.63 (m, 2H), 7.50 – 7.44 (m, 1H), 7.19 (d, *J* = 8.2 Hz, 2H), 2.35 (s, 3H); **^{13}C NMR** (125 MHz, $CDCl_3$) δ 162.1, 150.1, 148.1, 137.8, 135.4, 134.0, 129.7, 126.5, 122.5, 119.8, 21.1.

The spectroscopic data are consistent with those reported in the literature.[7]

***N*-(2-Methoxyphenyl)picolinamide (6o)**

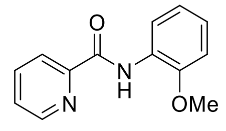

Colorless solid (5.7 mg, 25%); **^{1}H NMR** (600 MHz, $CDCl_3$) δ 10.58 (s, 1H), 8.66 (d, *J* = 4.3 Hz, 1H), 8.62 (d, *J* = 8.1 Hz, 1H), 8.30 (dd, *J* = 7.7, 1.3 Hz, 1H), 7.90 (t, *J* = 7.7 Hz, 1H), 7.50 – 7.43 (m, 1H), 7.10 (t, *J* = 7.8 Hz, 1H), 7.03 (t, *J* = 7.7 Hz, 1H), 6.94 (d, *J* = 8.0 Hz, 1H), 3.98 (d, *J* = 1.2 Hz, 3H); **^{13}C NMR** (150 MHz, $CDCl_3$) δ 162.2, 150.5, 148.9, 148.3, 137.6, 127.7, 126.4, 124.1, 122.5, 121.2, 119.9, 110.2, 56.0.

The spectroscopic data are consistent with those reported in the literature.[6]

***N*-(4-Methoxyphenyl)picolinamide (6p)**

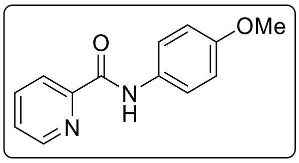

Colorless solid (7.1 mg, 31%); **^{1}H NMR** (600 MHz, $CDCl_3$) δ 9.92 (s, 1H), 8.61 (d, *J* = 3.9 Hz, 1H), 8.29 (d, *J* = 7.8 Hz, 1H), 7.90 (td, *J* = 7.7, 1.7 Hz, 1H), 7.73 – 7.67 (m, 2H), 7.47 (dd, *J* = 7.6, 4.7 Hz, 1H), 6.95 – 6.90 (m, 2H), 3.82 (s, 3H); **^{13}C NMR** (150 MHz, $CDCl_3$) δ 161.9, 156.6, 150.2, 148.1, 137.8, 131.2, 126.4, 122.5, 121.4, 114.4, 55.7.

The spectroscopic data are consistent with those reported in the literature.[3]

***N*-(2-Chloro)picolinamide (7o)**

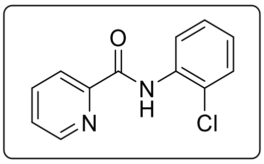

Conducted with 0.1 mL chlorobenzene. Colorless solid (8.4 mg, 33%); **^{1}H NMR** (600 MHz, $CDCl_3$) δ 10.72 (s, 1H), 8.71 – 8.61 (m, 2H), 8.30 (d, *J* = 7.8 Hz, 1H), 7.92 (t, *J* = 7.7 Hz, 1H), 7.55 – 7.48 (m, 1H), 7.43 (d, *J* = 9.5 Hz, 1H), 7.37 – 7.30 (m, 1H), 7.08 (t, *J* = 8.5 Hz, 1H); **^{13}C NMR** (125 MHz, $CDCl_3$) δ 162.3, 149.9, 148.5, 137.8, 134.9, 129.4, 127.9, 126.8, 124.8, 123.6, 122.6, 121.2.

The spectroscopic data are consistent with those reported in the literature.[8]

Mixture of *N*-(3-chloro)picolinamide (7m) and *N*-(4-chloro)picolinamide (7p)

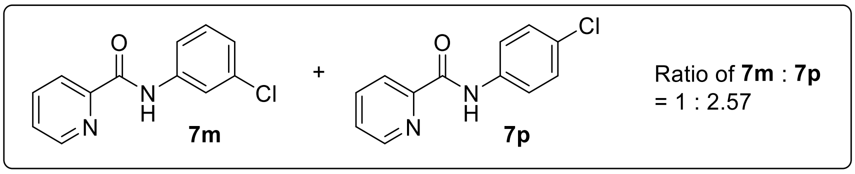

Conducted with 0.1 mL chlorobenzene. Colorless solid (7.1 mg, 25%).

***N*-(3-Chloro)picolinamide (7m)**, **^{1}H NMR** (600 MHz, $CDCl_3$) δ 10.04 (br s, 1H), 8.62 (d, *J* = 4.8 Hz, 1H), 8.29 (d, *J* = 7.8 Hz, 1H), 7.96 – 7.89 (m, 2H), 7.62 (d, *J* = 6.1 Hz, 1H), 7.50 (dd, *J* = 7.0, 5.2 Hz, 1H), 7.31 (t, *J* = 8.1 Hz, 1H), 7.13 (d, *J* = 10.1 Hz, 1H); **^{13}C NMR** (125 MHz, $CDCl_3$) δ 162.2, 149.6, 148.2, 139.0, 137.9, 134.9, 130.2, 126.8, 124.5, 122.6, 119.9, 117.8.

The spectroscopic data are consistent with those reported in the literature.[9]

***N*-(4-Chloro)picolinamide (7p)**, **^{1}H NMR** (600 MHz, $CDCl_3$) δ 10.04 (br s, 1H), 8.62 (d, *J* = 4.8 Hz, 1H), 8.29 (d, *J* = 7.8 Hz, 1H), 7.96 – 7.89 (m, 1H), 7.75 (d, *J* = 8.8 Hz, 2H), 7.50 (dd, *J* = 7.0, 5.2 Hz, 1H), 7.35 (d, *J* = 8.8 Hz, 2H); **^{13}C NMR** (125 MHz, $CDCl_3$) δ 162.1, 149.7, 148.1, 137.9, 136.5, 129.4, 129.3, 126.8, 122.6, 121.0.

The spectroscopic data are consistent with those reported in the literature.[10]

***N*-(2-Hydroxyphenyl)picolinamide (8o)**

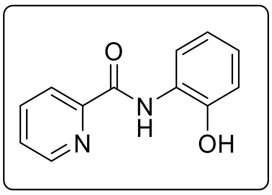

Colorless solid (7.7 mg, 36%); **^{1}H NMR** (500 MHz, $CDCl_3$) δ 10.24 (br s, 1H), 9.49 (s, 1H), 8.65 (d, *J* = 4.7 Hz, 1H), 8.30 (d, *J* = 7.8 Hz, 1H), 7.94 (t, *J* = 6.9 Hz, 1H), 7.57 – 7.49 (m, 1H), 7.18 (t, *J* = 8.1 Hz, 2H), 7.08 (dd, *J* = 7.9, 1.5 Hz, 1H), 6.92 (td, *J* = 7.6, 1.6 Hz, 1H); **^{13}C NMR** (125 MHz, $CDCl_3$) δ 163.4, 149.3, 148.5, 148.4, 138.1, 127.6, 127.2, 125.2, 122.9, 122.7, 120.5, 120.2.

The spectroscopic data are consistent with those reported in the literature.[1]

***N*-(4-Hydroxyphenyl)picolinamide (8p)**

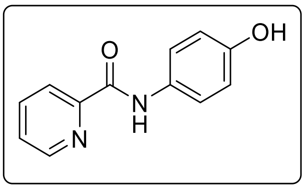

Colorless solid (2.6 mg, 12%); **^{1}H NMR** (600 MHz, DMSO-d_6) δ 10.39 (s, 1H), 9.28 (s, 1H), 8.71 (dd, *J* = 4.6, 1.2 Hz, 1H), 8.13 (dt, *J* = 7.8, 1.2 Hz, 1H), 8.09 – 8.01 (m, 1H), 7.71 – 7.60 (m, 3H), 6.75 (d, *J* = 8.8 Hz, 2H); **^{13}C NMR** (150 MHz, DMSO-d_6) δ 161.8, 153.9, 150.2, 148.3, 138.0, 130.0, 126.6, 122.1, 121.9, 115.0.

The spectroscopic data are consistent with those reported in the literature.[11]

***N*-(2,5-Dimethylphenyl)picolinamide (9)**

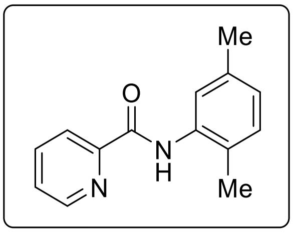

Colorless solid (14.1 mg, 62%); **^{1}H NMR** (600 MHz, $CDCl_3$) δ 10.05 (s, 1H), 8.63 (d, *J* = 4.7 Hz, 1H), 8.31 (d, *J* = 7.9 Hz, 1H), 8.18 – 8.08 (m, 1H), 7.91 (td, *J* = 7.7, 1.7 Hz, 1H), 7.48 (ddd, *J* = 7.6, 4.8, 1.3 Hz, 1H), 7.11 (d, *J* = 7.6 Hz, 1H), 6.91 (d, *J* = 7.8 Hz, 1H), 2.38 (s, 3H), 2.37 (s, 3H); **^{13}C NMR** (150 MHz, $CDCl_3$) δ 162.0, 150.4, 148.2, 137.8, 136.8, 135.8, 130.3, 126.5, 125.5, 125.1, 122.5, 122.1, 21.4, 17.4.

The spectroscopic data are consistent with those reported in the literature.[1]

Mixture of *N*-(2,3-dimethylphenyl)picolinamide (10a) and *N*-(3,4-dimethylphenyl)picolinamide (10b)

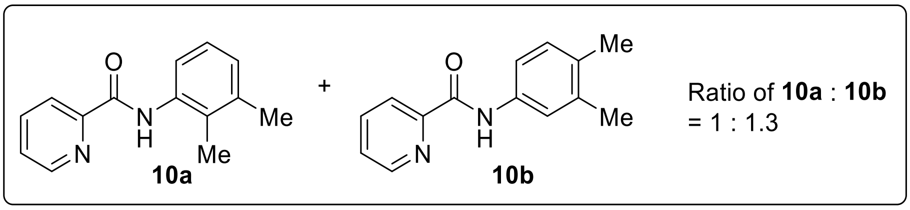

Colorless solid (12.7 mg, 56%); **m.p.** 65–67 °C; **IR** (cm^{-1}) 3346; 1680; 1586; 1523; 1460; 1307; 1139; 1040; 814; 663; **HRMS** (ESI) *m/z* calcd. for $C_{14}H_{15}N_2O^+$ $[M+H]^+$: 227.1179, found: 227.1183.

***N*-(2,3-Dimethylphenyl)picolinamide (10a),** **^{1}H NMR** (500 MHz, $CDCl_3$) δ10.05 (s, 1H), 8.63 (d, *J* = 4.7 Hz, 1H), 8.34 – 8.28 (m, 1H), 7.99 (d, *J* = 7.5 Hz, 1H), 7.94 – 7.87 (m, 1H), 7.50 – 7.45 (m, 1H), 7.17 (t, *J* = 7.8 Hz, 1H), 7.02 (d, *J* = 7.3 Hz, 1H), 2.34 (s, 3H), 2.29 (s, 4H); **^{13}C NMR** (150 MHz, $CDCl_3$) δ 161.9, 150.4, 148.2, 137.8, 137.4, 135.6, 127.9, 126.8, 126.5, 126.1, 122.5, 120.4, 20.8, 13.7.
The spectroscopic data are consistent with those reported in the literature.[12]

***N*-(3,4-Dimethylphenyl)picolinamide (10b),** **^{1}H NMR** (500 MHz, $CDCl_3$) δ 9.93 (s, 1H), 8.61 (d, *J* = 4.8 Hz, 1H), 8.34 – 8.28 (m, 1H), 7.94 – 7.87 (m, 1H), 7.59 (d, *J* = 2.3 Hz, 1H), 7.51 (dd, *J* = 8.1, 2.4 Hz, 1H), 7.50 – 7.45 (m, 1H), 7.14 (d, *J* = 8.1 Hz, 1H), 2.30 (s, 3H), 2.25 (s, 4H); **^{13}C NMR** (125 MHz, $CDCl_3$) δ 162.2, 150.2, 148.1, 137.8, 137.4, 135.7, 132.8, 130.2, 126.4, 122.6, 121.1, 117.3, 20.1, 19.4.

***N*-(5-Fluoro-2-methoxyphenyl)picolinamide (11a)**

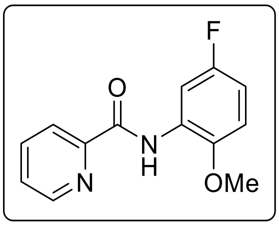

Colorless solid (9.3 mg, 38%); **m.p.** 108–110 °C; **^{1}H NMR** (500 MHz, $CDCl_3$) δ 10.61 (br s, 1H), 8.65 (d, *J* = 4.9 Hz, 1H), 8.46 (dd, *J* = 10.6, 3.1 Hz, 1H), 8.28 (d, *J* = 7.9 Hz, 1H), 7.90 (td, *J* = 7.8, 1.8 Hz, 1H), 7.48 (ddd, *J* = 7.6, 4.7, 1.2 Hz, 1H), 6.84 (dd, *J* = 9.0, 4.9 Hz, 1H), 6.80 – 6.73 (m, 1H), 3.95 (s, 3H); **^{13}C NMR** (125 MHz, $CDCl_3$) δ 162.3, 157.2 (d, *J* = 236.7 Hz), 150.1, 148.4, 145.1, 137.7, 128.4 (d, *J* = 12.0 Hz), 126.6, 122.6, 110.5 (d, *J* = 9.7 Hz), 109.4 (d, *J* = 23.4 Hz), 107.6 (d, *J* = 29.9 Hz), 56.5; **^{19}F NMR** (471 MHz, $CDCl_3$) δ -121.0; **IR** (cm^{-1}) 3348; 1690; 1614; 1532; 1483; 1431; 1253; 1180; 1024; 898; 677; **HRMS** (ESI) *m/z* calcd. for $C_{13}H_{12}FN_2O_2^+$ $[M+H]^+$: 247.0877, found: 247.0884.

***N*-(2-Fluoro-5-methoxyphenyl)picolinamide (11b)**

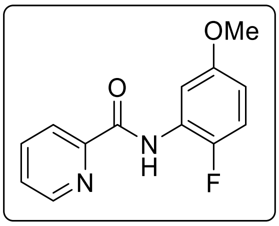

Colorless solid (2.2 mg, 9%); **m.p.** 116–118 °C; **^{1}H NMR** (500 MHz, $CDCl_3$) δ 10.35 (s, 1H), 8.65 (d, *J* = 4.7 Hz, 1H), 8.36 – 8.22 (m, 2H), 7.92 (td, *J* = 7.8, 1.8 Hz, 1H), 7.50 (ddd, *J* = 7.6, 4.9, 1.4 Hz, 1H), 7.05 (dd, *J* = 10.5, 9.0 Hz, 1H), 6.61 (dt, *J* = 9.0, 3.8 Hz, 1H), 3.84 (s, 3H); **^{13}C NMR** (100 MHz, $CDCl_3$) δ 162.3, 156.1, 149.7, 148.4, 147.5 (d, *J* = 237.0 Hz), 137.8, 126.9 (d, *J* = 13.2 Hz), 126.8, 122.5, 115.3 (d, *J* = 20.5 Hz), 109.9 (d, *J* = 7.3 Hz), 106.1, 56.0; **^{19}F NMR** (376 MHz, $CDCl_3$) δ -141.1; **IR** (cm^{-1}) 3352; 1686; 1621; 1537; 1481; 1434; 1234; 1033; 715; **HRMS** (ESI) *m/z* calcd. for $C_{13}H_{12}FN_2O_2^+$ $[M+H]^+$: 247.0877, found: 247.0880.

N-(2,4,6-Trimethoxyphenyl)picolinamide (12)

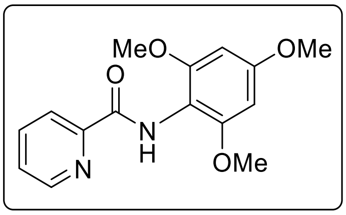

Colorless solid (18.8 mg, 65%); **m.p.** 150–152 °C; **^{1}H NMR** (500 MHz, $CDCl_3$) δ 9.12 (br s, 1H), 8.62 (d, *J* = 4.7 Hz, 1H), 8.27 (d, *J* = 7.8 Hz, 1H), 7.85 (t, *J* = 8.5 Hz, 1H), 7.47 – 7.40 (m, 1H), 6.20 (s, 2H), 3.83 (s, 3H), 3.81 (s, 6H); **^{13}C NMR** (125 MHz, $CDCl_3$) δ 163.1, 160.1, 156.6, 150.4, 148.2, 137.3, 126.2, 122.7, 107.3, 91.2, 56.2, 55.6; **IR** (cm^{-1}) 3294; 1679; 1591; 1515; 1229; 1159; **HRMS** (ESI) *m/z* calcd. for $C_{15}H_{17}N_2O_4^+$ $[M+H]^+$: 289.1183, found: 289.1190.

N-Mesitylpicolinamide (13)

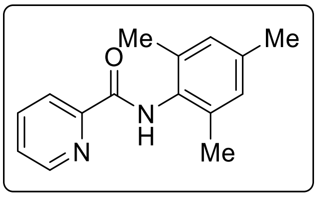

Colorless solid (14.5 mg, 60%); **^{1}H NMR** (500 MHz, $CDCl_3$) δ 9.39 (br s, 1H), 8.63 (d, *J* = 4.7 Hz, 1H), 8.30 (dt, *J* = 7.9, 1.1 Hz, 1H), 7.94 – 7.84 (m, 1H), 7.55 – 7.43 (m, 1H), 6.94 (s, 2H), 2.30 (s, 3H), 2.25 (s, 6H); **^{13}C NMR** (125 MHz, $CDCl_3$) δ 162.7, 150.1, 148.3, 137.6, 136.9, 135.3, 131.3, 129.1, 126.5, 122.7, 21.1, 18.6.

The spectroscopic data are consistent with those reported in the literature.[13]

N-Cyclopentylpicolinamide (14)

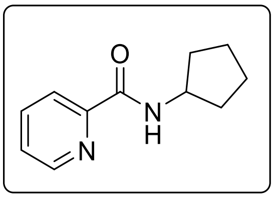

Conducted with 20 mol% of **Rh1a** and 0.1 mL substrate loading. Colorless solid (7.5 mg, 39%); **^{1}H NMR** (500 MHz, $CDCl_3$) δ 8.53 (d, *J* = 4.5 Hz, 1H), 8.19 (d, *J* = 7.8 Hz, 1H), 7.98 (s, 1H), 7.87 – 7.80 (m, 1H), 7.40 (dd, *J* = 7.4, 4.9 Hz, 1H), 4.40 (h, *J* = 6.9 Hz, 1H), 2.13 – 2.03 (m, 2H), 1.76 (dd, *J* = 6.6, 4.4 Hz, 2H), 1.66 (dd, *J* = 7.6, 4.6 Hz, 2H), 1.57 (td, *J* = 6.7, 2.1 Hz, 2H); **^{13}C NMR** (150 MHz, $CDCl_3$) δ 163.9, 150.3, 148.1, 137.5, 126.0, 122.3, 51.2, 33.3, 24.0.

The spectroscopic data are consistent with those reported in the literature [15].

N-(Tetrahydrofuran-2-yl)picolinamide (15)

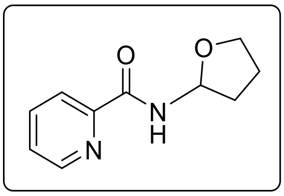

Conducted with 20 mol% of **Rh1a** and 0.1 mL substrate loading. Colorless resin (5.7 mg, 30%); **m.p.** 69–71 °C; **^{1}H NMR** (500 MHz, $CDCl_3$) δ 8.54 (d, *J* = 5.3 Hz, 1H), 8.44 – 8.27 (m, 1H), 8.20 (d, *J* = 7.8 Hz, 1H), 7.84 (t, *J* = 8.6 Hz, 1H), 7.43 (dd, *J* = 7.8, 4.6 Hz, 1H), 5.94 (ddd, *J* = 8.7, 6.5, 4.2 Hz, 1H), 4.01 (q, *J* = 7.9 Hz, 1H), 3.88 (q, *J* = 6.8 Hz, 1H), 2.34 – 2.20 (m, 1H), 2.14 – 1.96 (m, 2H), 1.96 – 1.88 (m, 1H); **^{13}C NMR** (125 MHz, $CDCl_3$) δ 164.2, 149.7, 148.2, 137.5, 126.5, 122.6, 81.1, 67.7, 32.3, 24.9; **IR** (cm^{-1}) 3320; 1672; 1507; 1463; 1433; 1042; 966; 750; **HRMS** (ESI) *m/z* calcd. for $C_{10}H_{12}N_2NaO_2^+$ $[M+Na]^+$: 215.0791, found: 215.0799.

_N_-(1,4-Dioxan-2-yl)picolinamide (16)

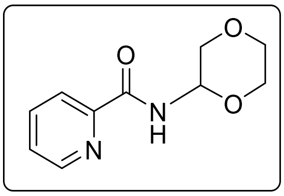

Conducted with 20 mol% of **Rh1a** and 0.1 mL substrate loading. Colorless resin (7.7 mg, 37%); **^{1}H NMR** (500 MHz, $CDCl_3$) δ 8.72 (d, *J* = 9.4 Hz, 1H), 8.57 (ddd, *J* = 4.7, 1.7, 1.1 Hz, 1H), 8.22 (dt, *J* = 7.8, 1.1 Hz, 1H), 7.86 (td, *J* = 7.6, 1.7 Hz, 1H), 7.46 (ddd, *J* = 7.6, 4.9, 1.4 Hz, 1H), 5.56 (ddd, *J* = 9.3, 6.5, 2.7 Hz, 1H), 4.00 – 3.92 (m, 2H), 3.84 – 3.75 (m, 2H), 3.73 – 3.67 (m, 1H), 3.60 (dd, *J* = 11.4, 6.5 Hz, 1H); **^{13}C NMR** (125 MHz, $CDCl_3$) δ 164.4, 149.3, 148.3, 137.6, 126.8, 122.8, 74.3, 69.6, 66.5, 64.1; **IR** (cm^{-1}) 3374; 1681; 1505; 1464; 1120; 1080; 910; 865; 636; **HRMS** (ESI) *m/z* calcd. for $C_{10}H_{13}N_2O_3^+$ $[M+H]^+$: 209.0921, found: 209.0926.

_N_-(1-Ethoxyethyl)picolinamide (17)

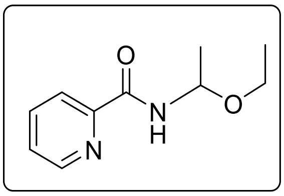

Conducted with 20 mol% of **Rh1a** and 0.1 mL substrate loading. Colorless resin (5.6 mg, 29%); **^{1}H NMR** (500 MHz, $CDCl_3$) δ 8.57 (d, *J* = 4.9 Hz, 1H), 8.34 – 8.22 (m, 1H), 8.21 (dd, *J* = 7.8, 0.9 Hz, 1H), 7.86 (td, *J* = 7.7, 1.8 Hz, 1H), 7.50 – 7.39 (m, 1H), 5.64 – 5.49 (m, 1H), 3.61 (ddq, *J* = 47.3, 9.6, 7.0 Hz, 2H), 1.48 (d, *J* = 6.0 Hz, 3H), 1.20 (t, *J* = 7.1 Hz, 3H); **^{13}C NMR** (125 MHz, $CDCl_3$) δ 164.3, 149.6, 148.3, 137.5, 126.6, 122.7, 76.4, 63.7, 22.2, 15.3; **IR** (cm^{-1}) 3376; 1679; 1591; 1570; 1465; 1126; 1087; **HRMS** (ESI) *m/z* calcd. for $C_{10}H_{14}N_2NaO_2^+$ $[M+Na]^+$: 217.0947, found: 217.0955.

_N_-(_tert_-Butoxy)picolinamide (18)

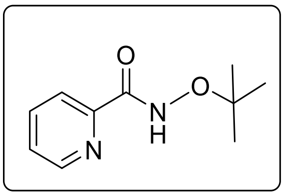

Conducted with 5 mol% of **Rh1f** and 0.1 mL substrate loading. Colorless resin (9.9 mg, 51%); **^{1}H NMR** (500 MHz, $CDCl_3$) δ 9.89 (s, 1H), 8.52 (ddd, *J* = 4.7, 1.8, 0.9 Hz, 1H), 8.18 (dt, *J* = 7.8, 1.1 Hz, 1H), 7.85 (td, *J* = 7.8, 1.8 Hz, 1H), 7.44 (ddd, *J* = 7.6, 4.7, 1.4 Hz, 1H), 1.37 (s, 9H); **^{13}C NMR** (100 MHz, $CDCl_3$) δ 162.9, 149.5, 148.3, 137.6, 126.7, 122.6, 82.8, 26.5; **IR** (cm^{-1}) 3340; 1692; 1464; 1431; 1366; 1279; 1184; 908; **HRMS** (ESI) *m/z* calcd. for $C_{10}H_{14}N_2NaO_2^+$ $[M+Na]^+$: 217.0947, found: 217.0954.

_N_'-Methyl-_N_'-phenylpicolinohydrazide (19)

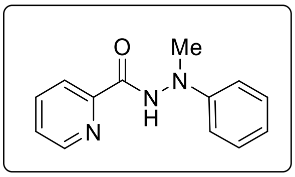

Conducted with 5 mol% of **Rh1f**. Colorless resin (11.3 mg, 50%); **^{1}H NMR** (500 MHz, $CDCl_3$) δ 9.59 (s, 1H), 8.58 (d, *J* = 4.7 Hz, 1H), 8.24 (d, *J* = 7.8 Hz, 1H), 7.89 (td, *J* = 7.8, 1.8 Hz, 1H), 7.55 – 7.45 (m, 1H), 7.30 – 7.23 (m, 2H), 6.93 (dd, *J* = 8.9, 1.1 Hz, 2H), 6.88 (tt, *J* = 7.3, 1.1 Hz, 1H), 3.32 (s, 3H); **^{13}C NMR** (125 MHz, $CDCl_3$) δ 163.0, 149.6, 149.4, 148.4, 137.6, 129.3, 126.9, 122.8, 120.1, 113.5, 41.1. The spectroscopic data are consistent with those reported in the literature.[16]

N-(3,4-Dihydroquinolin-1(2*H*)-yl)picolinamide (20)

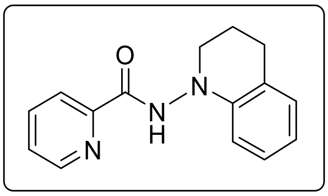

Conducted with 5 mol% of **Rh1f**. Colorless resin (9.2 mg, 36%); Major rotamer form of NMR spectra: **^{1}H NMR** (500 MHz, $CDCl_3$) δ 9.50 (s, 1H), 8.65 – 8.55 (m, 1H), 8.31 – 8.21 (m, 1H), 7.89 (td, *J* = 7.6, 1.7 Hz, 1H), 7.49 (ddd, *J* = 7.6, 4.8, 1.3 Hz, 1H), 7.07 – 6.98 (m, 2H), 6.82 (dd, *J* = 8.1, 1.2 Hz, 1H), 6.75 (td, *J* = 7.3, 1.3 Hz, 1H), 3.58 (dd, *J* = 6.5, 4.8 Hz, 2H), 2.85 (t, *J* = 6.5 Hz, 2H), 2.19 (qd, *J* = 6.4, 4.8 Hz, 2H); **^{13}C NMR** (125 MHz, $CDCl_3$) δ 163.0, 149.6, 148.4, 145.6, 137.6, 129.4, 127.2, 126.8, 123.6, 122.8, 119.7, 113.0, 51.8, 27.2, 22.5; **IR** (cm^{-1}) 3294; 1683; 1601; 1580; 1489; 1457; 1432; 1303; 1278; 1135; 997; 745; **HRMS** (ESI) *m/z* calcd. for $C_{15}H_{15}N_3NaO^+$ $[M+Na]^+$: 276.1107, found: 276.1111.

3-4. Low-yielding substrate scope

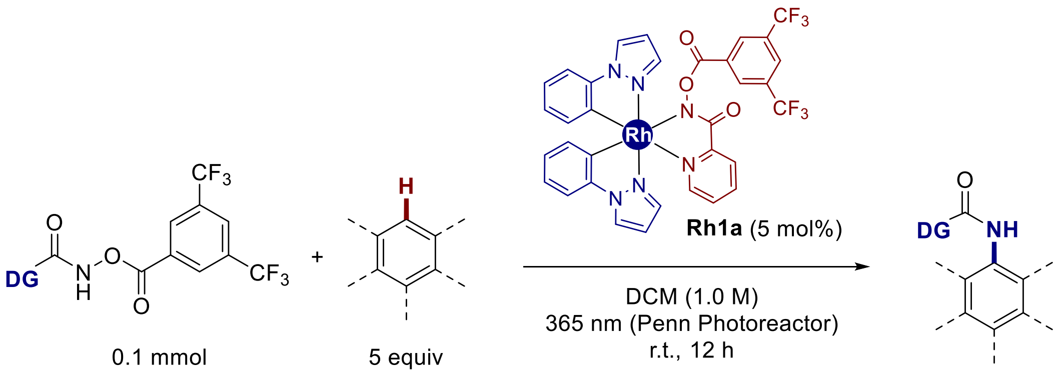

Hydroxamate substrates *(with 5 equiv of benzene)*

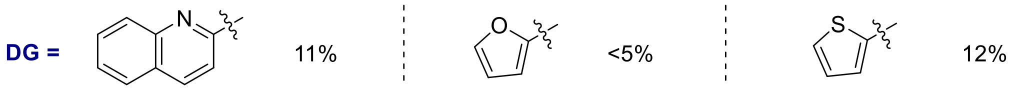

Arene substrates *(with 0.1 mmol of **1a**)*

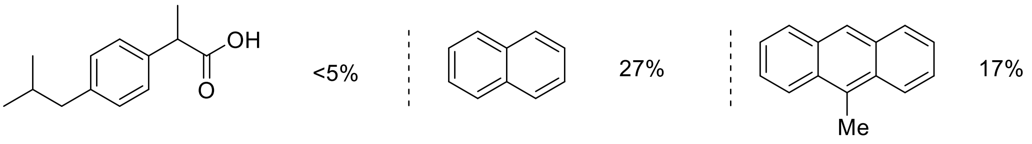

* Yields were measured by ^{1}H NMR of crude reaction mixture in the presence of internal standard (dibromomethane)

4. Mechanistic experiments

4-1. UV-Vis measurements of Rh1a and Rh2 in CH_2Cl_2

UV–Vis spectra were measured using a Shimadzu UV-2600 spectrophotometer. Solutions of **Rh1a** and **Rh2** in dichloromethane with different concentrations were prepared and sampled in quartz cuvettes (optical path length 1cm) for absorption measurements (Figures S1 and S2).

The absorption coefficient of **Rh1a** at 365 nm is determined as 610 $cm^{-1}M^{-1}$.

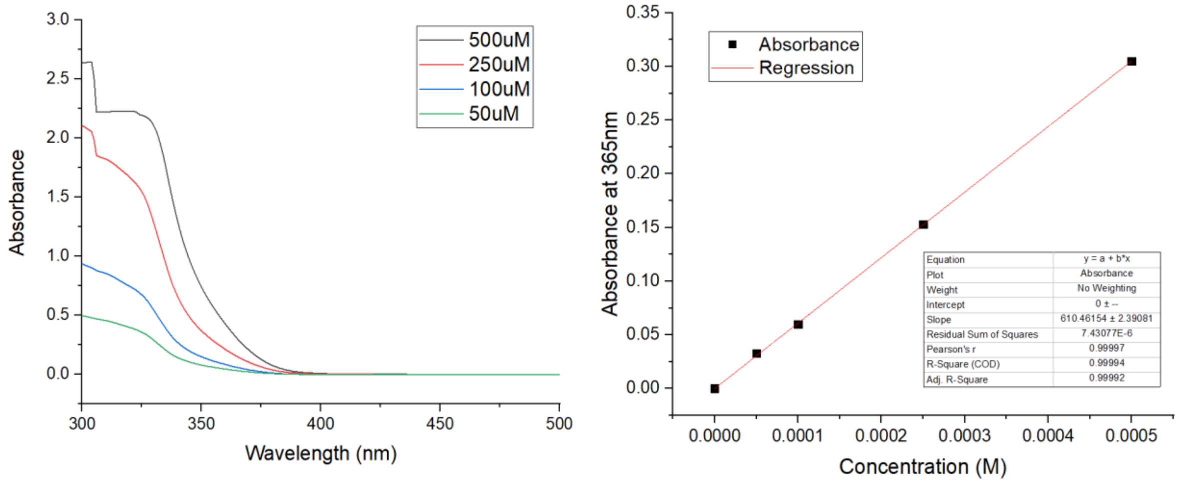

Figure S1. Concentration dependent UV–Vis spectra of **Rh1a** in CH_2Cl_2 (left) and absorption coefficient at 365 nm (right)

The absorption coefficient of **Rh2** at 400 nm is determined as 598 $cm^{-1}M^{-1}$.

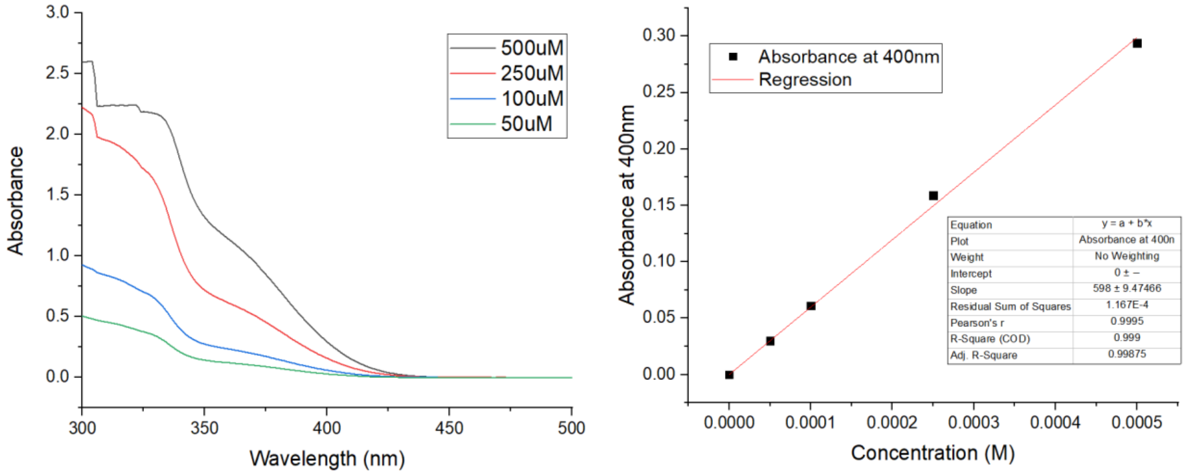

Figure S2. Concentration dependent UV–Vis spectra of **Rh2** in CH_2Cl_2 (left) and absorption coefficient at 400 nm (right)

The UV–Vis absorption spectra of rhodium complexes **Rh1a**, **Rh3**, **Rh4**, **Rh5,** and **Rh1f** are shown in Figure S3. Except for **Rh1a**, all rhodium complexes showed absorption at 400 nm.

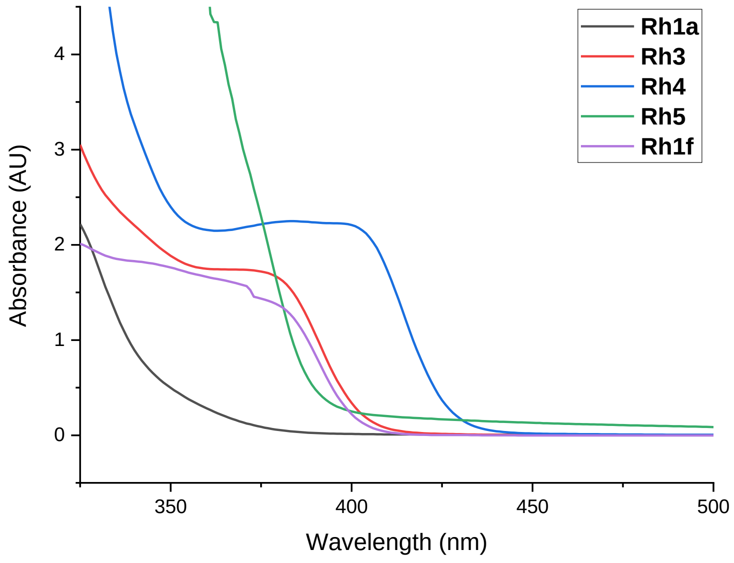

Figure S3. UV–Vis absorption spectra of **Rh1a**, **Rh3**, **Rh4**, **Rh5**, and **Rh1f** (250 µM)

4-2. UV-Vis measurements of Rh1b, Rh1c and Rh1d in benzene

Solutions of **Rh1b**, **Rh1c**, and **Rh1d** in benzene with different concentrations were prepared and sampled in quartz cuvettes (optical path length 1cm) for absorption measurements.

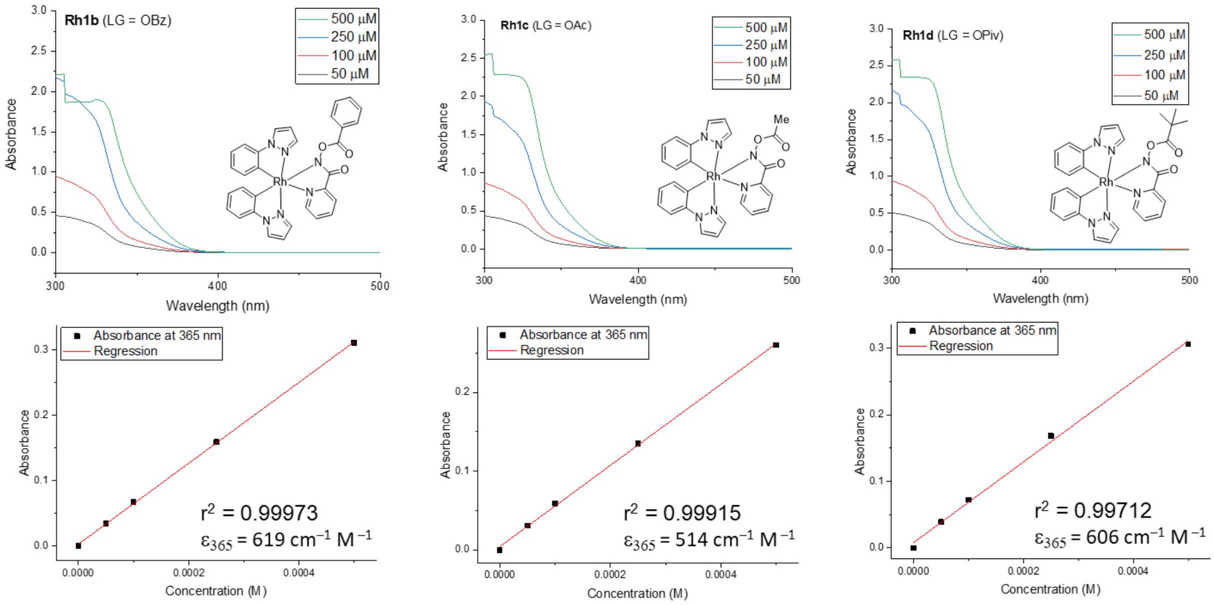

Figure S4. Concentration dependent UV–Vis spectra of **Rh1b** (left), **Rh1c** (middle), and **Rh1d** (right) in benzene (top) and absorption coefficient at 365 nm (bottom)

4-3. UV-Vis monitoring of ^{14}N-Rh1a under light irradiation

Under N_2 atmosphere, a solution of **Rh1a** (100 μM) in benzene was prepared and transferred to a quartz cuvette. For UV-Vis measurements with *in situ* photoirradiation of the sample, an optical fiber connected to Mic-LED was placed in the solution (365 nm), which was covered by a thin glass tube. After the experimental setup, UV-Vis time-monitoring was performed under the 365 nm light irradiation for 25 min. During the time monitoring, benzene-amidated product **Rh2** was increased.

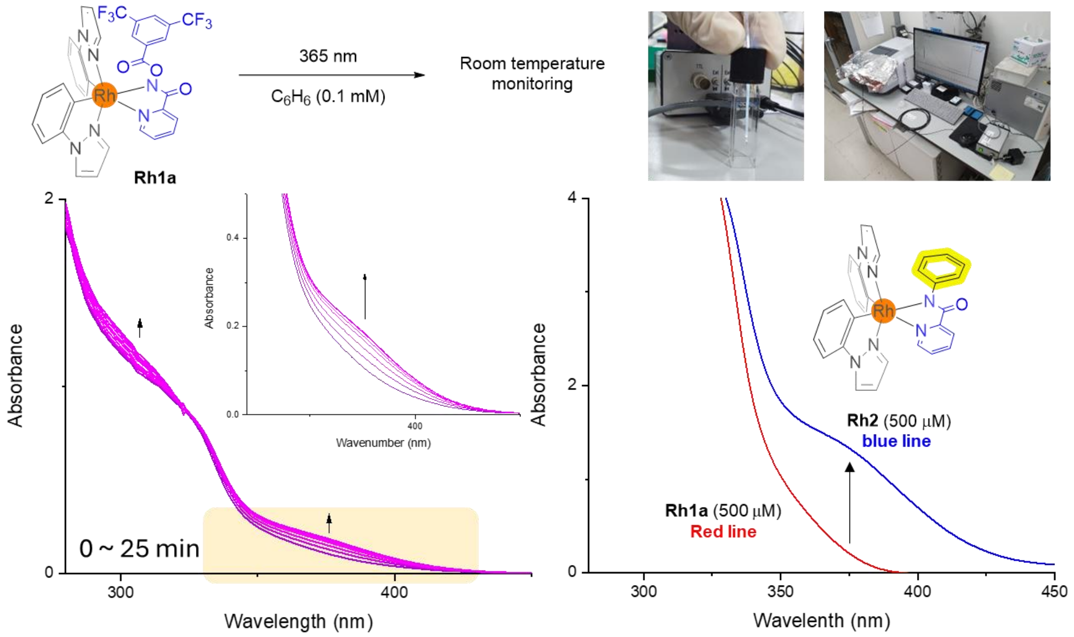

Figure S5. UV-Vis time monitoring of **Rh1a** solution in benzene under light irradiation (365nm, left) and UV-Vis spectrum of **Rh1a** and **Rh2** in benzene (500 μM)

4-4. EPR (Electron Paramagnetic Resonance) experiment

X-band CW electron paramagnetic resonance (EPR) spectroscopy was performed using Bruker EMXplus spectrometer equipped with a standard resonator. For EPR measurements with *in situ* photoirradiation of sample, we utilized a 5 mm EPR tube and a coaxial inner cell (NE-379-5-COIC-Sp-A, New Era©). The optical fiber connected to Mic-LED was placed into the coaxial inner cell, and the whole tube was inserted into the EPR machine.

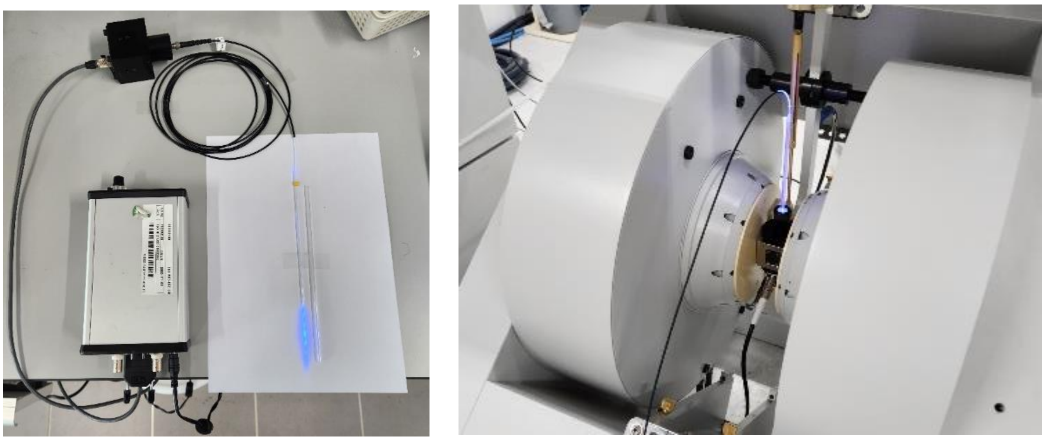

Figure S6. Setup for EPR experiments under photoirradiation.

Under N_2 atmosphere, **^{14}N-Rh1a** (0.005 mmol) was dissolved in 0.5 mL of benzene, and the solution was transferred to a 5 mm EPR tube (**Experiment 1**). Another solution of **^{15}N-Rh1a** (0.005 mmol) was independently prepared (**Experiment 2**). The coaxial tube was inserted into the outer cell, and the joint of tubes was sealed with Teflon tape. Before X-band EPR measurement, optical fiber was inserted into the coaxial inner tube. EPR spectrum of the solution sample was collected at room temperature with the following spectrometer settings: MW frequency = 9.377 GHz, MW power = 2.0 mW, center field = 3330 G, sweep width = 200 G, sweep time = 30 s, modulation frequency = 100 kHz, modulation amplitude = 1 G, power attenuation = 20 dB, time constant = 0.01 ms, conversion time = 15 ms, and gain = 30 dB.

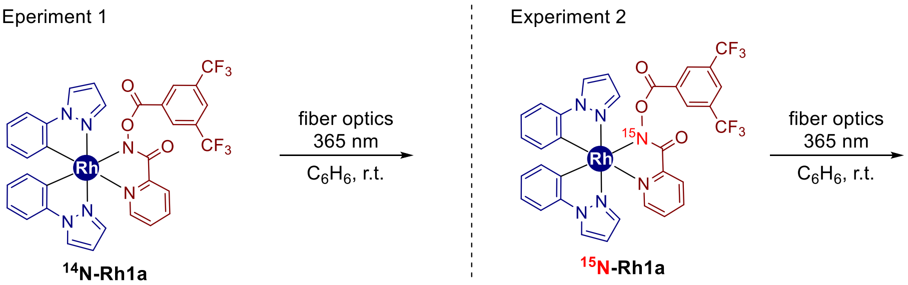

After the light was turned on, photo-EPR monitoring experiment was started, and the signal was monitored for 20 min. From **Experiment 1**, a triplet signal arose and diminished over time (Figure S7). From **Experiment 2**, a doublet signal arose and diminished over time (Figure S8).

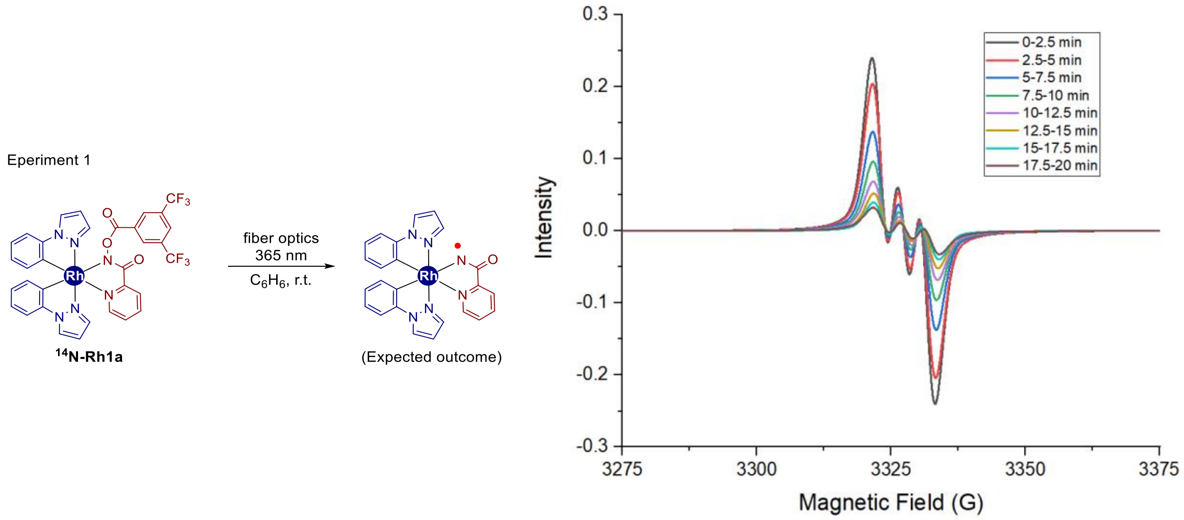

Figure S7. Photo-EPR monitoring of ^{14}**N-Rh1a** (Experiment 1).

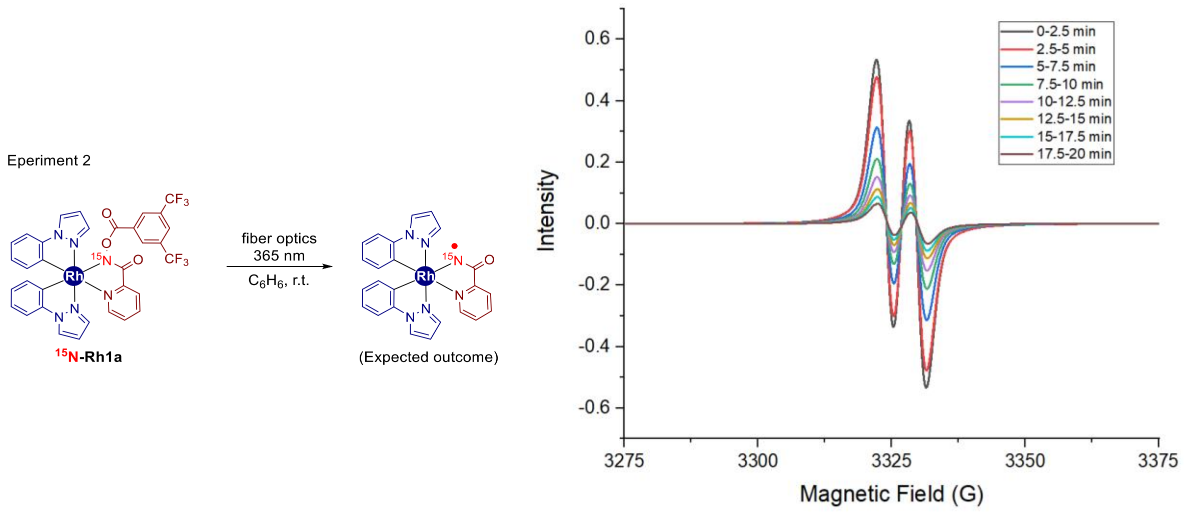

Figure S8. Photo-EPR monitoring of ^{15}**N-Rh1a** (Experiment 2).

Under N_2 atmosphere, **^{14}N-Rh1a** (0.005 mmol) and DMPO (5,5-dimethyl-1-pyrroline-*N*-oxide, 2 equiv, 0.01 mmol) were dissolved in 0.5 mL of benzene, and the solution was transferred to a 5 mm EPR tube (**Experiment 3**). A solution of **^{15}N-Rh1a** (0.005 mmol) and DMPO (0.01 mmol) in benzene was independently prepared (**Experiment 4**). Also, the solution of DMPO (0.01 mmol) in benzene was prepared for control experiment (**Experiment 5**).

The coaxial tube was inserted into the outer cell, and the joint of tubes was sealed with Teflon tape. Before X-band EPR measurement, optical fiber was inserted into the coaxial inner tube. EPR spectrum of the solution sample was collected at room temperature with the following spectrometer settings: MW frequency = 9.759 GHz, MW power = 2.0 mW, center field = 3484.80 G, sweep width = 200 G, sweep time = 30 s, modulation frequency = 100 kHz, modulation amplitude = 1 G, power attenuation = 20 dB, time constant = 0.01 ms, conversion time = 15 ms, and gain = 30 dB.

Eperiment 3

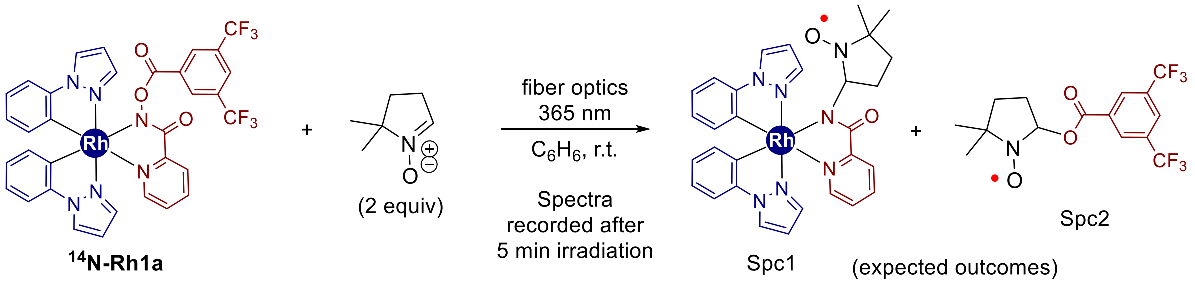

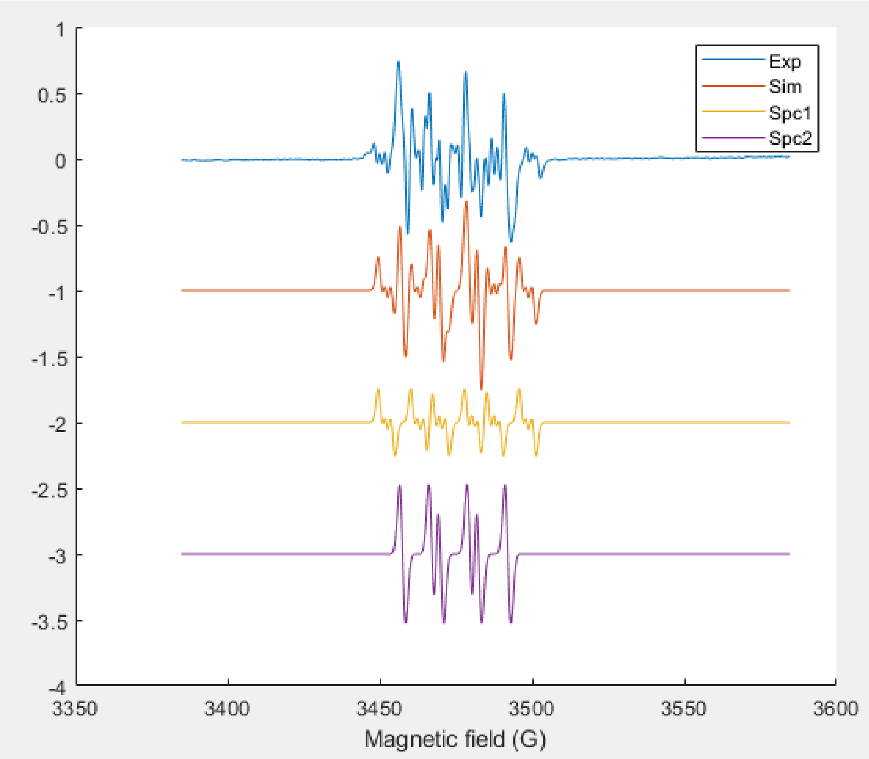

Figure S9. Photo-EPR monitoring of **^{14}N-Rh1a** and DMPO (Experiment 3).

Simulation parameters for Experiment 3:

Spc1) S = 1/2; Nucs = '14N,14N,H'; g_{iso} = 2.00625; A = [5.5, 30, 50]; lwpp = [0.18]; weight = 0.34.

Spc2) S = 1/2; Nucs = '14N,H'; g_{iso} = 2.00661; A = [35. 27]; lwpp = [0.2]; weight = 0.3.

Eperiment 4

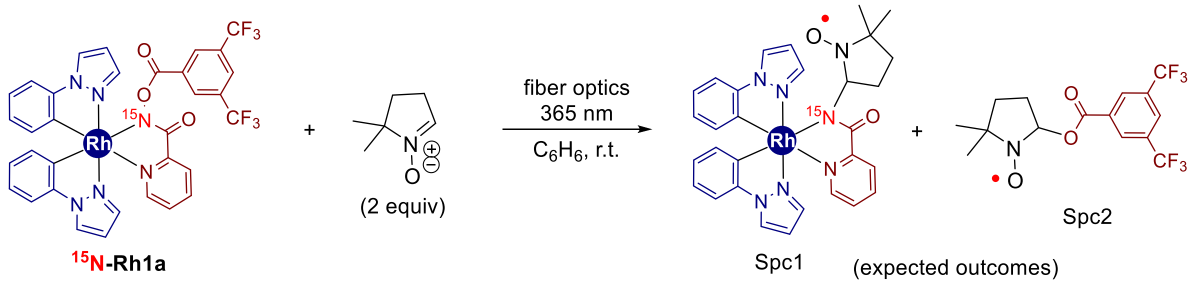

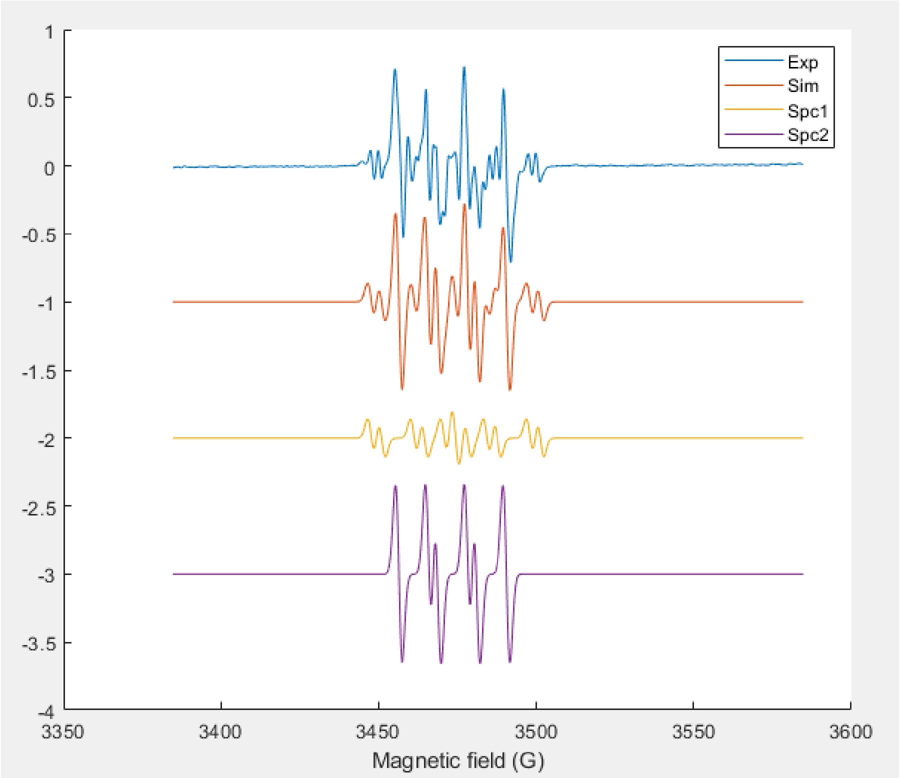

Figure S10. Photo-EPR monitoring of **^{15}N-Rh1a** and DMPO solution in benzene (Experiment 4).

Simulation parameters forExperiment 4:

Spc1) S = 1/2; Nucs = '15N,14N,H'; g = 2.00609; A = [9.3, 38, 65]; lwpp = [0.23]; weight = 0.21.

Spc2) S = 1/2; Nucs = '14N,H'; g = 2.00669; A = [35. 27]; lwpp = [0.22]; weight = 0.45.

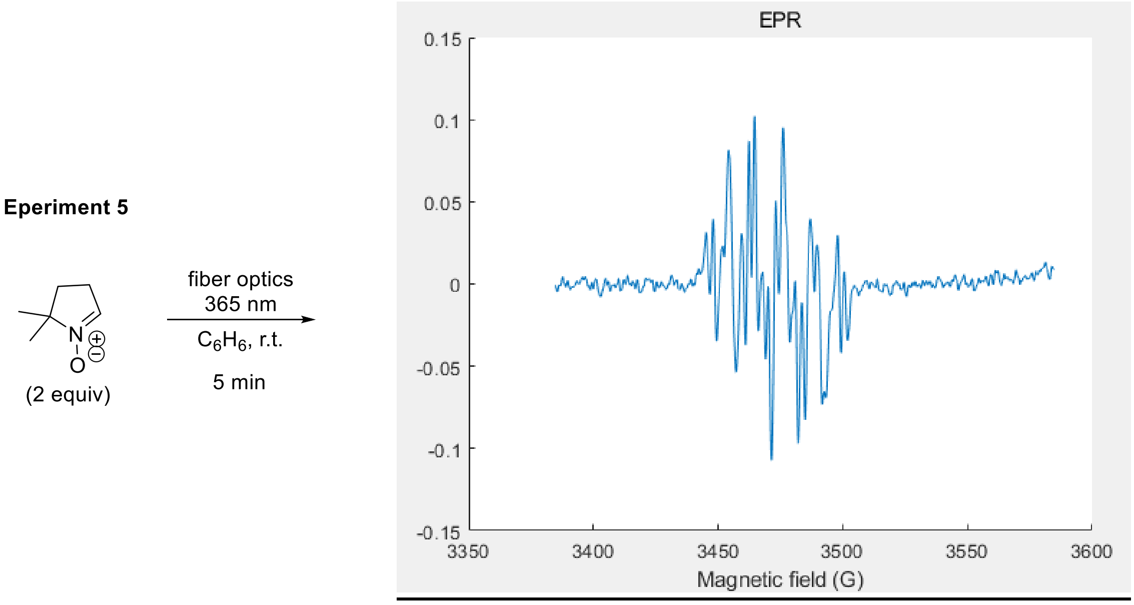

Figure S11. Photo-EPR monitoring with DMPO solution in benzene (Experiment 5).

4-5. Quantitative EPR analysis

Standard curve using TEMPO

Under N_2 atmosphere, TEMPO solutions with variated concentrations (1 μM ~ 100 μM) in benzene (0.5 mL) were prepared, and each solution was transferred to a 5 mm EPR tubes. EPR spectrum of each TEMPO solution sample was collected at room temperature twice with the following spectrometer settings: MW frequency = 9.377 GHz, MW power = 2.0 mW, center field = 3330 G, sweep width = 200 G, sweep time = 30 s, modulation frequency = 100 kHz, modulation amplitude = 1 G, power attenuation = 20 dB, time constant = 0.01 ms, conversion time = 15 ms, and gain = 30 dB (**Note**: same with section 4-2, **Experiment 1**).

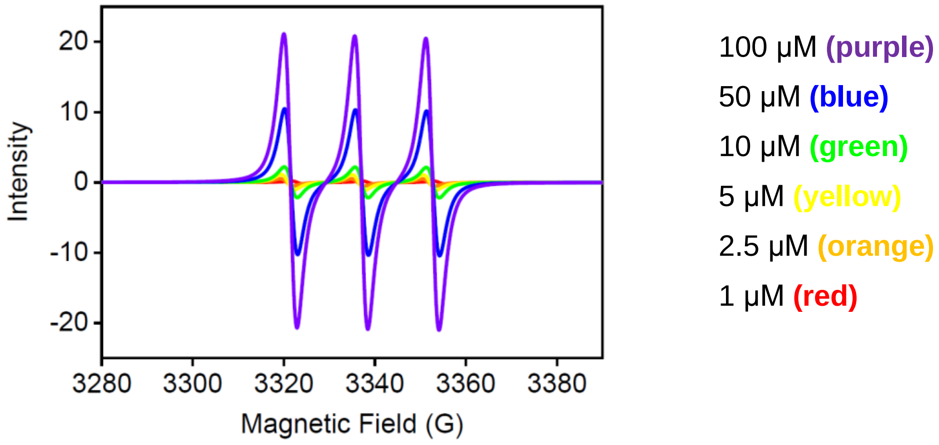

Figure S12. EPR spectrum of standard solutions of TEMPO. Spectra of the first trial is shown.

The obtained EPR spectrum of TEMPO (indicated concentration) was doubly integrated. By linear regression of each point (x = concentration, y = double integration value), the equation of the standard curve was obtained as shown in Figure S13

Table S2. Double integration value of EPR spectrum obtained from standard TEMPO solutions

Conc. of TEMPO (μM)	**1st**	**2nd**	**Average**	**Error**
**1**	15.41	15.25	15.33	0.08
**2.5**	38.05	40.49	39.27	1.22
**5**	80.67	78.81	79.74	0.93
**10**	161.3	160.6	160.95	0.35
**50**	745.8	730.8	738.3	7.5
**100**	1563	1597	1580	17

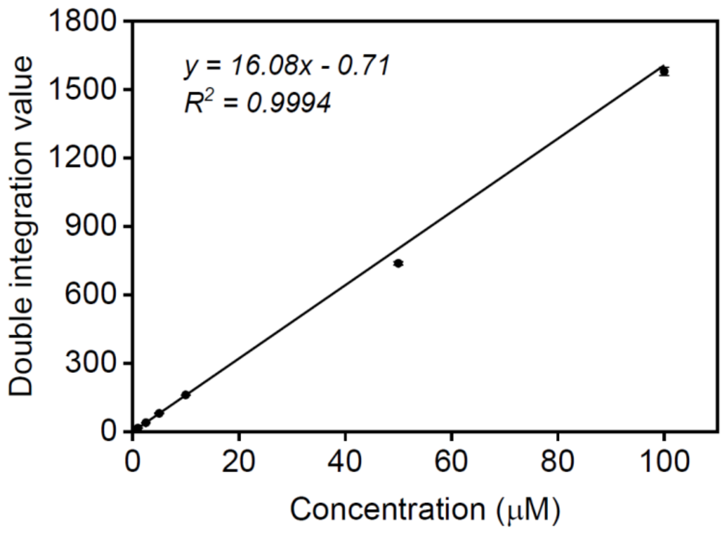

Figure S13. Standard curve using TEMPO as an external standard reagent

Quantification of obtained EPR spectrum

According to the standard curve obtained using TEMPO as an external standard, the obtained EPR spectrum from **^{14}N-Rh1a** upon the 365 nm light irradiation (**Experiment 1**) was doubly integrated, and calculated the concentration of detected radical species.

Table S3. Calculated radical concentration in **Experiment 1**

Time (min)	0~2.5	2.5~5	5~7.5	7.5~10	10~12.5	12.5~15	15~17.5	17.5~20
Double integration value	14.79	13.01	9.11	6.64	5.06	3.600	2.66	2.84
Concetration (μM)	0.88	0.77	0.52	0.37	0.27	0.18	0.12	0.13

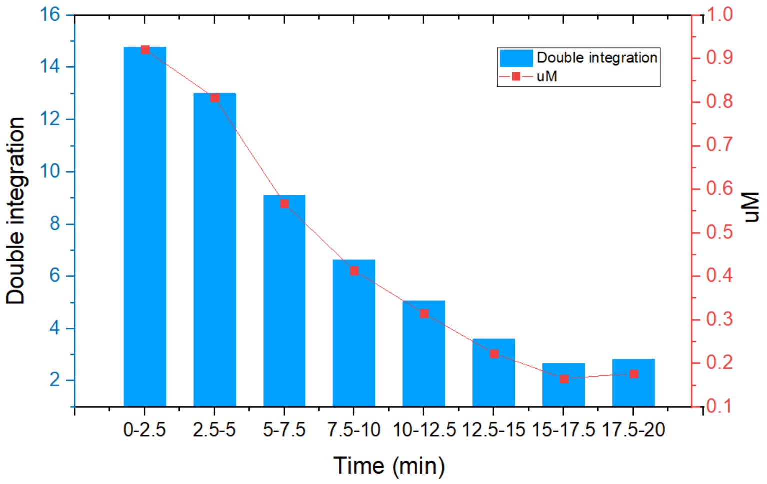

Figure S14. Graph for calculated double integration value and concentration in **Experiment 1**

4-6. Low temperature (100 K) EPR spectroscopy

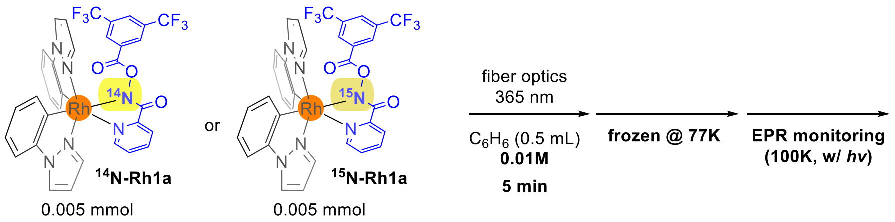

Under N_2 atmosphere, **^{14}N-Rh1a** (0.005 mmol) was dissolved in 0.5 mL of benzene, and the solution was transferred to a 5 mm EPR tube (**Experiment 6**). A solution of **^{15}N-Rh1a** (0.005 mmol) in benzene was independently prepared (**Experiment 7**). The coaxial tube was inserted into the outer cell, and the joint of tubes was sealed with Teflon tape. Before X-band EPR measurement, optical fiber was inserted into the coaxial inner tube. After 365 nm light irradiation for 5 min at room temperature, the resulting solution was frozen at 77 K (liquid N_2). EPR spectrum of the frozen sample was collected at 100 K with the following spectrometer settings: MW frequency = 9.367 GHz, MW power = 2.0 mW, center field = 3330.0 G, sweep width = 200 G, sweep time = 30 s, modulation frequency = 100 kHz, modulation amplitude = 1 G, power attenuation = 20 dB, time constant = 0.01 ms, conversion time = 15 ms, and gain = 30 dB.

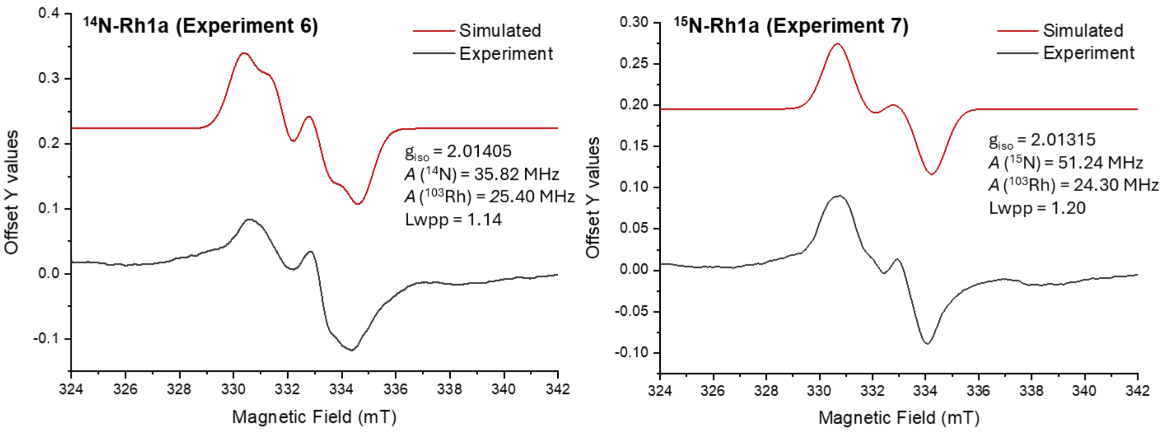

Figure S15. EPR spectrum of frozen ^{14}**N**(or ^{15}**N**)-**Rh1a** sample after 365 nm light irradiation

4-7. Light on-off experiment using EPR spectroscopy

Under N_2 atmosphere, ^{14}**N-Rh1a** (0.005 mmol) was dissolved in 0.5 mL of benzene, and the solution was transferred to a 5 mm EPR tube. The coaxial tube was inserted into the outer cell, and the joint of tubes was sealed with Teflon tape. Before X-band EPR measurement, optical fiber was inserted into the coaxial inner tube. The EPR spectrum of the solution sample was monitored at room temperature, with the light being turned on and off alternatively. The radical signal was diminished immediately when the light was turned off.

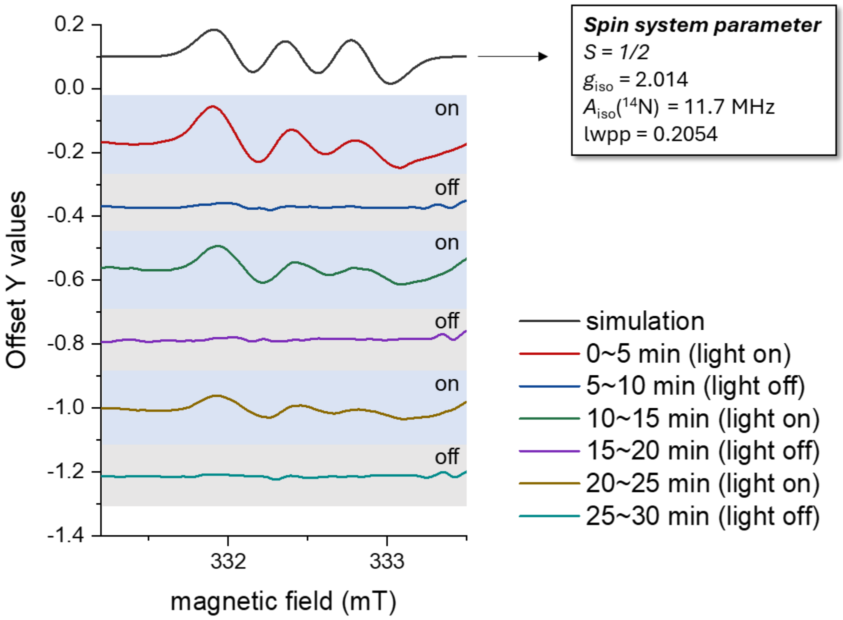

Figure S16. EPR monitoring of **Rh1a** solution in benzene at room temperature under alternative light irradiation conditions

4-8. Indirect detection of acyloxy radical decomposition

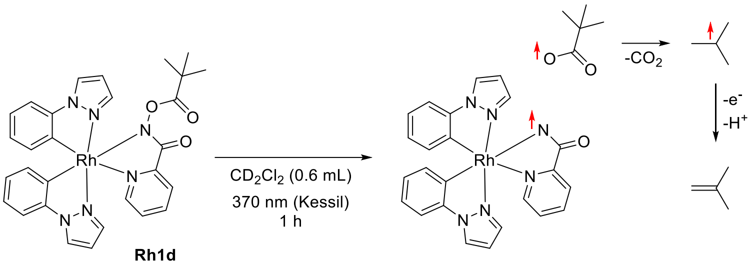

Under N_2 atmosphere, **Rh1d** (0.005 mmol) was dissolved in 0.6 mL of CD_2Cl_2, and the solution was transferred to a J-Young NMR tube. The solution was irradiated using a Kessil lamp (370 nm) for 1 h, and ^1H NMR was obtained.

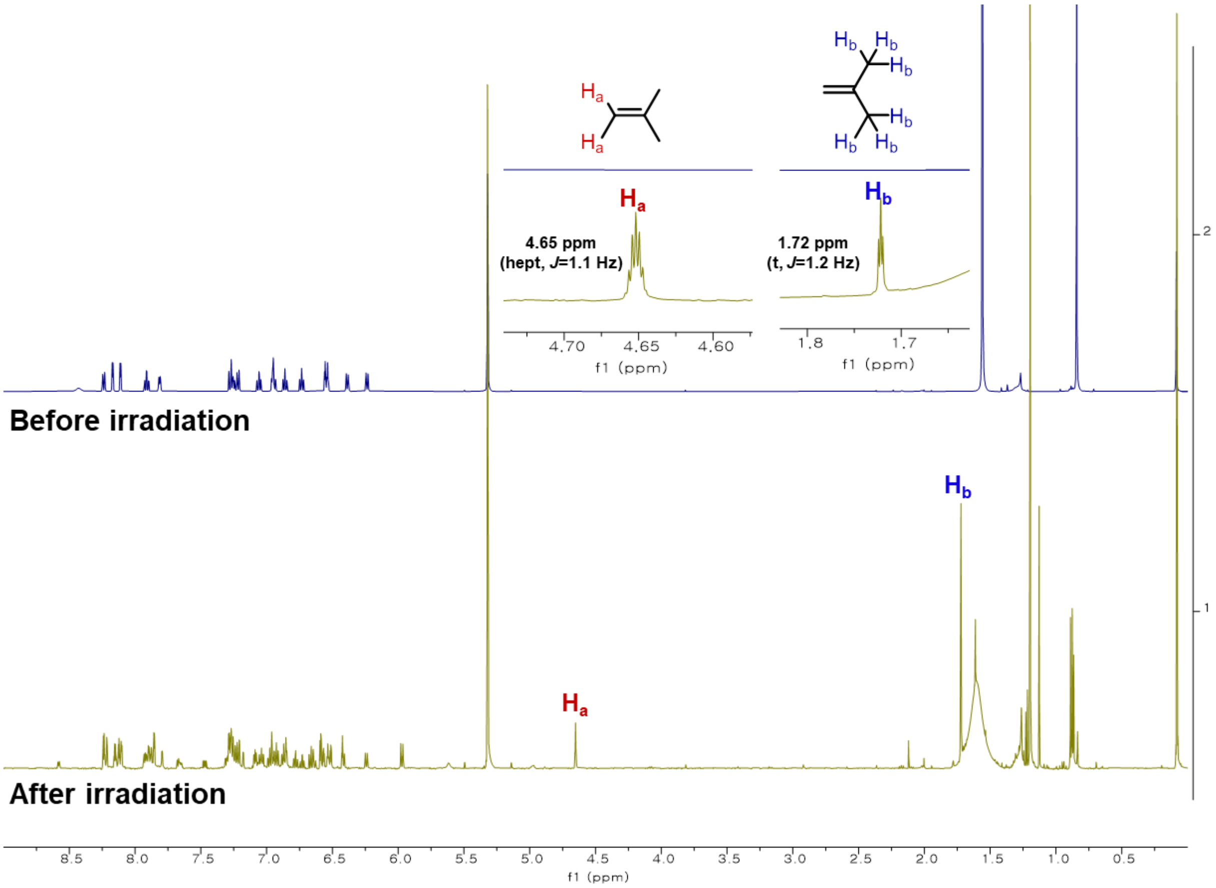

Figure S17. ^1H NMR spectra of **Rh1d** (top) and the crude mixture after light irradiation (bottom)

4-9. ESI–MS study on the photoactivation of Rh-hydroxamate complexes

Electrospray ionization mass spectrometry (ESI–MS) data were obtained using an Agilent 6530 Accurate Mass quadrupole time-of-flight (Q-TOF) mass spectrometer (Agilent Technologies, Santa Clara, USA) equipped with an ESI source in a positive ion mode. Solutions of **^{14}N-Rh1a**, **^{15}N-Rh1a**, and **^{14}N-Rh1e** in acetonitrile or methanol (10 μM) were prepared for the ESI–MS study. The solutions of rhodium complexes were irradiated with a 365 nm light source (Penn PhD Photoreactor M2, 100% intensity) for 30 s prior to injection into the mass spectrometer. The capillary voltage, fragmentor voltage, gas temperature, and drying gas flow were set to 3 kV, 50 V, 250 °C, and 12 L/min, respectively.

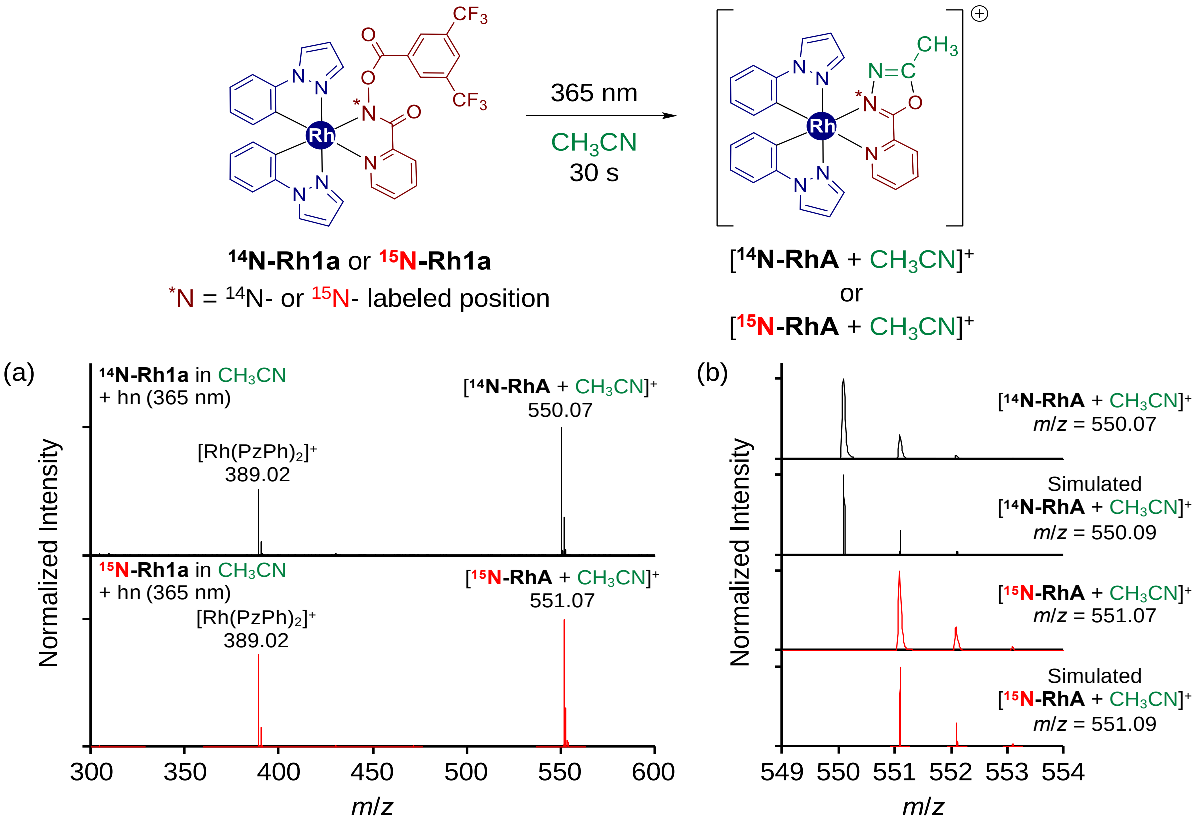

Figure S18. (a) ESI–MS spectra of **^{14}N-Rh1a** and **^{15}N-Rh1a** after irradiation of light (365 nm) in CH_3CN. (b) Isotopic patterns of [**^{14}N-RhA** + CH_3CN]$^+$ and [**^{15}N-RhA** + CH_3CN]$^+$ ions observed in (a).

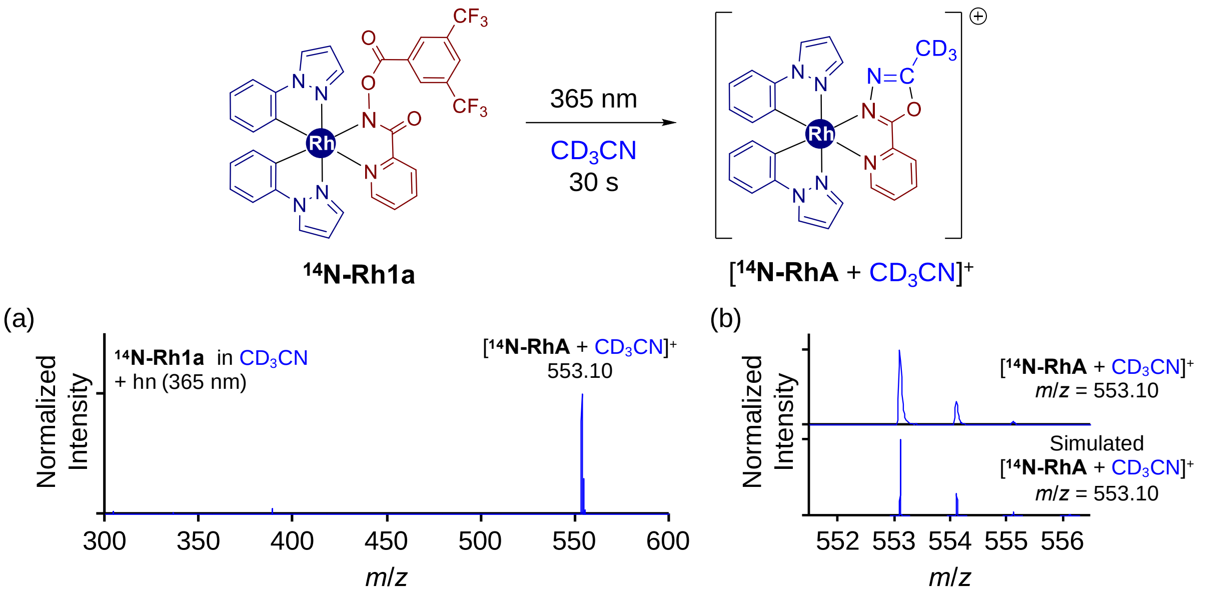

Figure S19. (a) ESI–MS spectra of **^{14}N-Rh1a** after irradiation of light (365 nm) in CD_3CN. (b) Isotopic patterns of [**^{14}N-RhA** + CD_3CN]$^+$ ion observed in (a).

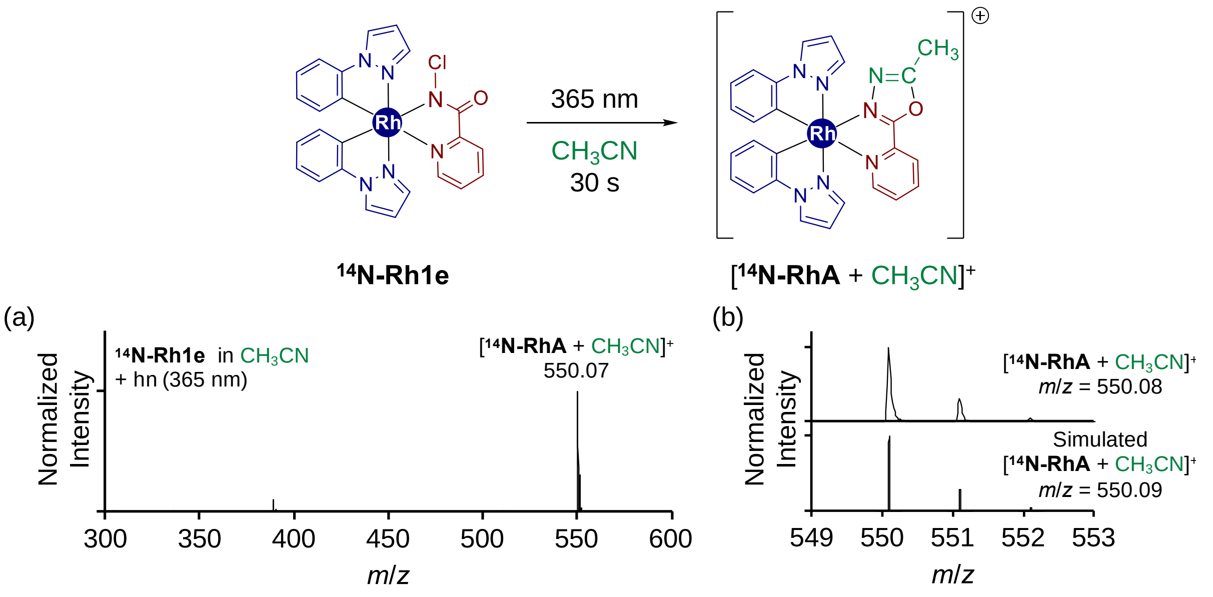

Figure S20. (a) ESI–MS spectra of **^{14}N-Rh1e** after irradiation of light (365 nm) in CH_3CN. (b) Isotopic patterns of [**^{14}N-RhA** + CH_3CN]$^+$ ion observed in (a).

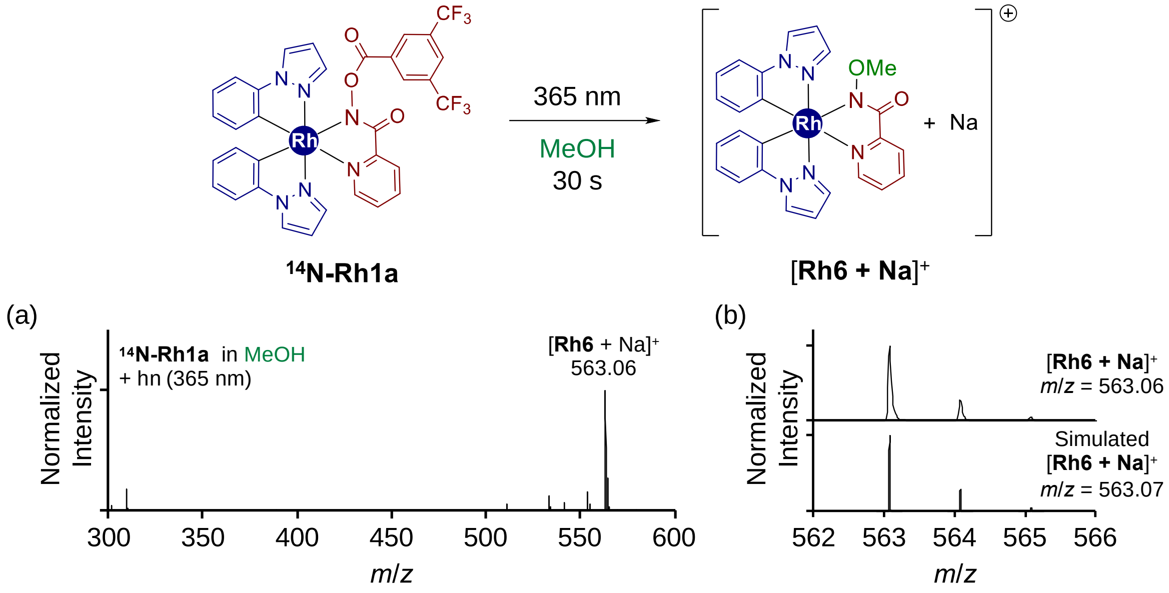

Figure S21. (a) ESI–MS spectra of ^{14}**N-Rh1a** after irradiation of light (365 nm) in MeOH. (b) Isotopic patterns of [**Rh6** + Na]$^+$ ion observed in (a).

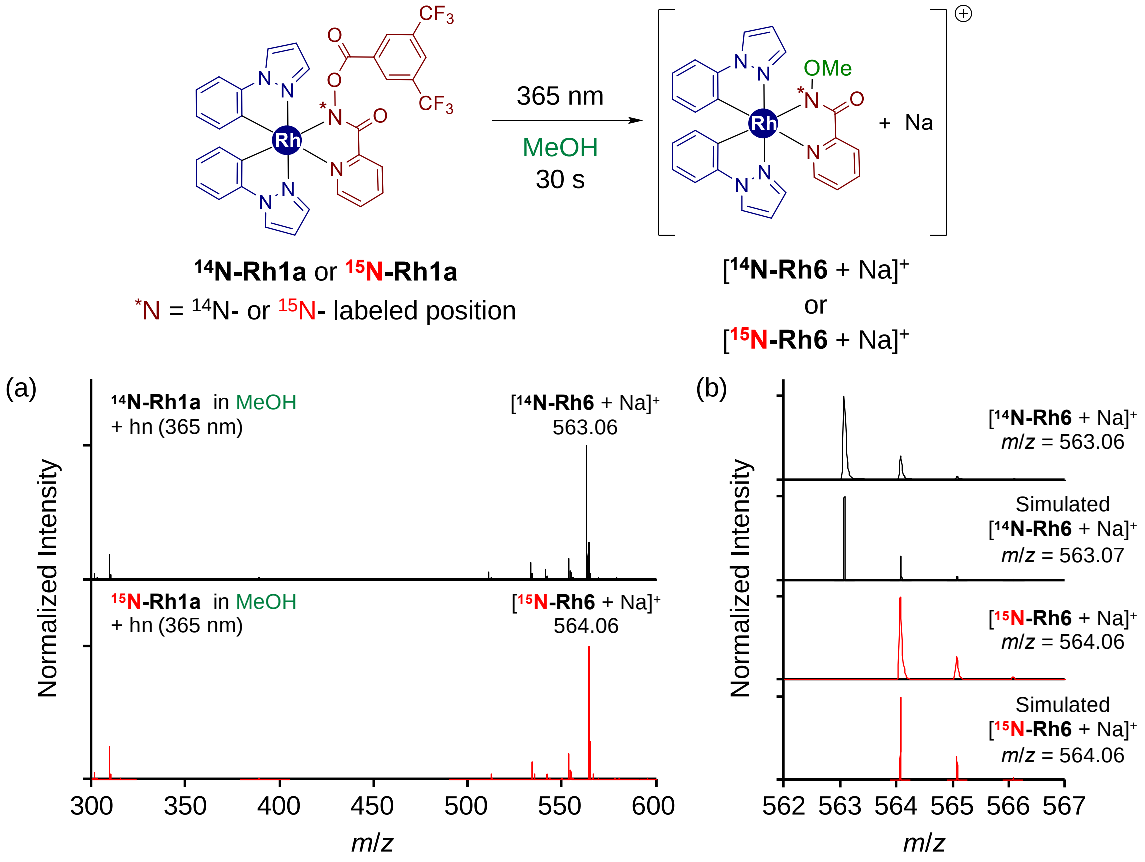

Figure S22. (a) ESI–MS spectra of ^{14}**N-Rh1a** and ^{15}**N-Rh1a** after irradiation of light (365 nm) in MeOH. (b) Isotopic patterns of [^{14}**N-Rh6** + Na]$^+$ and [^{15}**N-Rh6** + Na]$^+$ ions observed in (a).

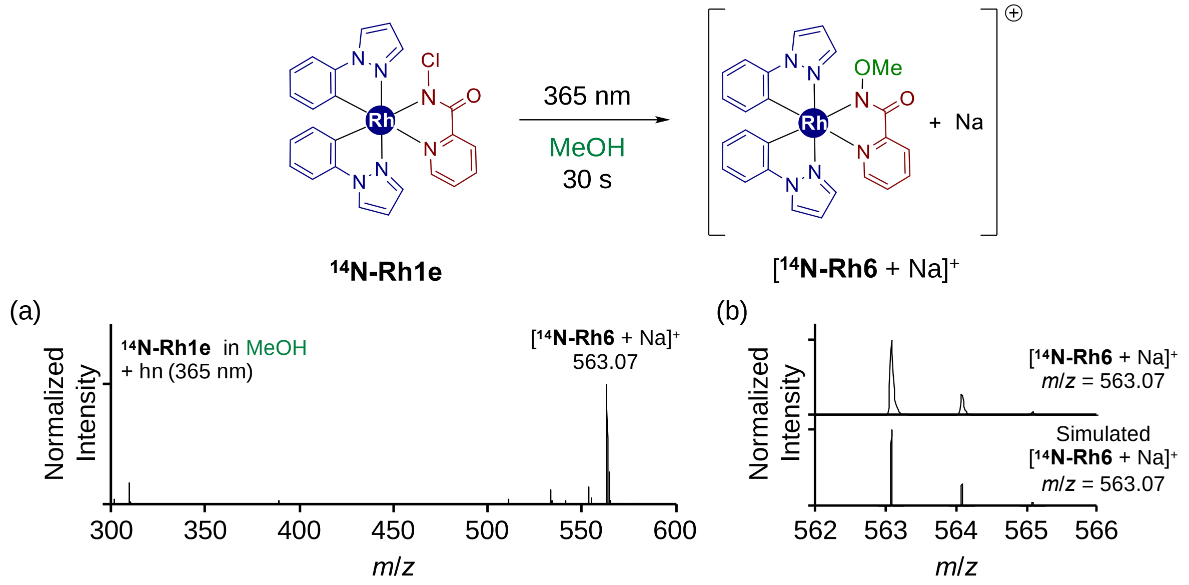

Figure S23. (a) ESI–MS spectra of **^{14}N-Rh1e** after irradiation of light (365 nm) in MeOH. (b) Isotopic patterns of [^{14}**N-Rh6** + Na]$^+$ ion observed in (a).

4-10. Kinetic isotope effect (KIE) experiment

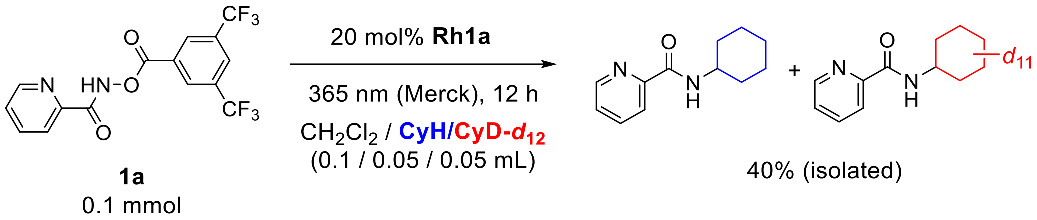

To a pre-dried 4 mL teflon-lined vial equipped with a stir bar were added **Rh1a** (0.02 mmol, 15.3 mg), **1a** (0.1 mmol, 37.8 mg), and anhydrous CH_2Cl_2 (1 M, 0.1 mL) under N_2 atmosphere. Cyclohexane (5 equiv, 54.0 µL) and cyclohexane-d_{12} (5 equiv, 54.0 µL) was added to the reaction mixture. The vial was sealed with a cap, and equipped in a Merck Penn PhD photoreactor M2.[5] The reaction mixture was irradiated for 12 min (365 nm, 100% intensity) and maintained at room temperature. After completion, saturated $NaHCO_3$ (*aq*, 10 mL) was added to the reaction mixture and was extracted with dichloromethane twice. The combined organic layers were dried over anhydrous $MgSO_4$ and the solvent was removed under reduced pressure. The C–H amidation product was isolated by silica chromatography (*n*-hexane to *n*-hexane/EtOAc/MeOH = 20:10:1, 40% combined yield of $\mathbf{3_H}$ + $\mathbf{3_D}$). The KIE value was determined based on the ^{1}H NMR analysis (Figure S24) on the isolated product. The average value of three independent results is reported ([$\mathbf{3_H}$]/[$\mathbf{3_D}$] = 1.98 ± 0.24).

Table S4. Results of intermolecular KIE measurements.

Trials	1st run	2nd run	3rd run	Average	Stdev
**[3_H]/[3_D]**	2.125	2.125	1.70	1.98	0.24

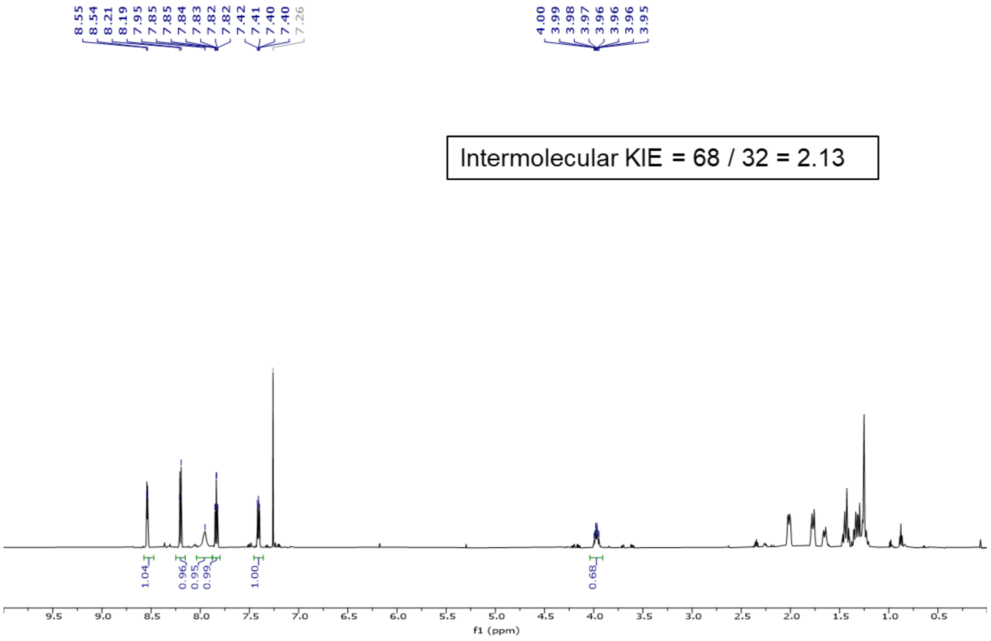

Figure S24. Sample ^{1}H NMR analysis on the obtained product mixture of $\mathbf{3_H}$ + $\mathbf{3_D}$.

4-11. Stereoretentive C–H amidation

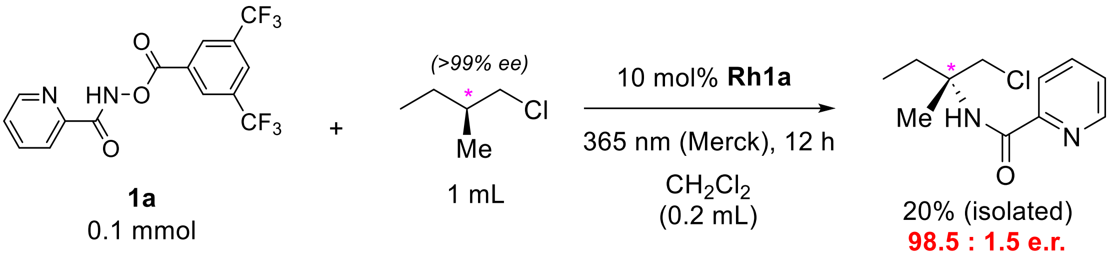

To a pre-dried 4 mL teflon-lined vial equipped with a stir bar were added **Rh1a** (0.01 mmol, 7.7 mg), **1a** (0.1 mmol, 37.8 mg), and anhydrous CH_2Cl_2 (1 M, 0.2 mL) under N_2 atmosphere. (*S*)-1-Chloro-2-methylbutane (1 mL) was added to the reaction mixture. The vial was sealed with a cap, and equipped in a Merck Penn PhD photoreactor M2.[5] The reaction mixture was irradiated for 12 min (365 nm, 100% intensity) and maintained at room temperature. After completion, saturated $NaHCO_3$ (*aq*, 10 mL) was added to the reaction mixture and was extracted with dichloromethane twice. The combined organic layers were dried over anhydrous $MgSO_4$ and the solvent was removed under reduced pressure. The C–H amidation product was isolated by silica chromatography (*n*-hexane to *n*-hexane/EtOAc/MeOH = 20:10:1).

(*R*)-*N*-(1-Chloro-2-methylbutan-2-yl)picolinamide ((*R*)-4)

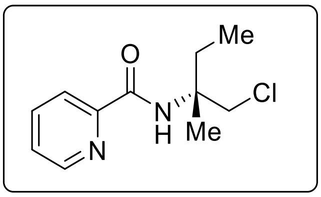

Colorless resin (4.5 mg, 20%); **^{1}H NMR** (500 MHz, $CDCl_3$) δ 8.54 (d, J = 6.4 Hz, 1H), 8.16 (d, J = 7.8 Hz, 1H), 8.02 (s, 1H), 7.84 (t, J = 8.6 Hz, 1H), 7.42 (dd, J = 8.2, 5.3 Hz, 1H), 4.08 (d, J = 11.0 Hz, 1H), 3.90 (d, J = 11.0 Hz, 1H), 2.19 – 2.08 (m, 1H), 1.76 (dq, J = 15.0, 7.5 Hz, 1H), 1.49 (s, 3H), 0.96 (t, J = 7.5 Hz, 3H); **^{13}C NMR** (100 MHz, $CDCl_3$) δ 163.8, 150.3, 148.1, 137.5, 126.3, 121.9, 57.0, 49.9, 29.6, 22.1, 8.1; HPLC Analysis (210 nm, CHIRALPAK AD-H column, 98:2 *n*-hexane/2-propanol, 1.0 mL/min, 32 °C) indicated 98.5 : 1.5 e.r.: t_R (major) = 11.2 min, t_S (minor) = 11.6 min (Fig. S5).

The spectroscopic data are consistent with those reported in the literature.[1] HPLC chromatogram of the racemic sample of **4** has been excerpted from the literature.[1]

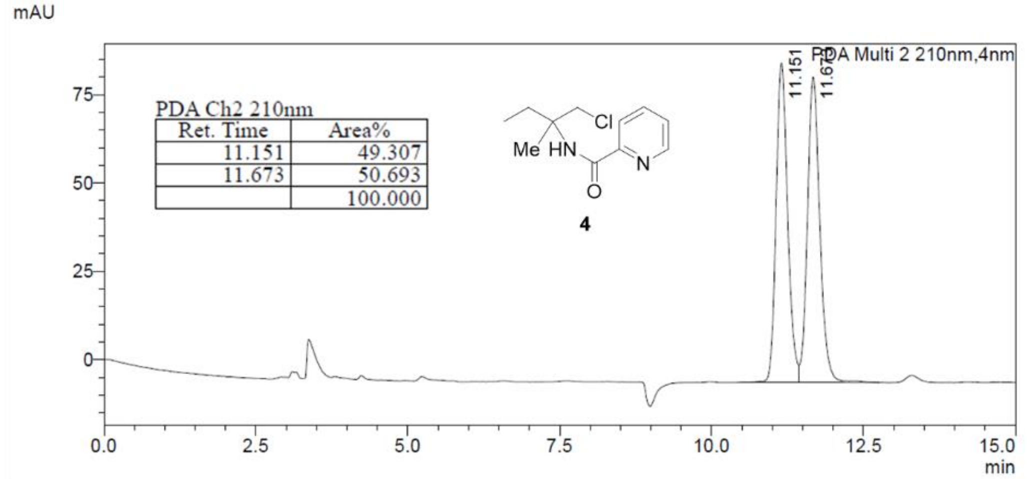

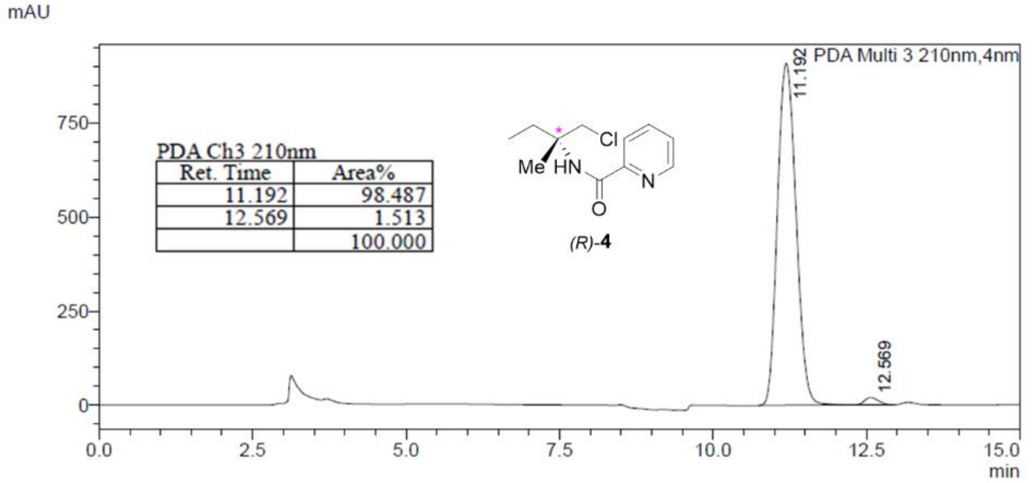

Figure S25. HPLC traces of racemic and scalemic compounds of **4**.

4-12. Methanol solvolysis experiment

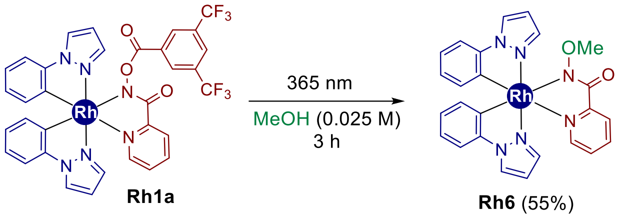

To a pre-dried, teflon-lined 4 mL vial equipped with a stir bar were added **Rh1a** (0.025 mmol, 19.1 mg), anhydrous methanol (1 mL) under N_2 atmosphere. The vial was sealed with a cap and equipped with a Merck Penn PhD photoreactor M2.[5] The reaction mixture was irradiated for 3 h (365 nm, 100% intensity) and maintained at room temperature. Upon completion, the solvent was removed under reduced pressure, and the product yield of **Rh6** was determined using ^{1}H NMR analysis in the presence of 1,1,2-trichloroethane as an internal standard in CD_2Cl_2. Detailed results are summarized in Figure S26. The structure of **Rh6** was confirmed by preparing an authentic sample of **Rh6** (see section 3). Also a single-crystal X-ray diffraction analysis of the crystal grown from the crude photolysis mixture confirmed the structure of **Rh6** (see section 6).

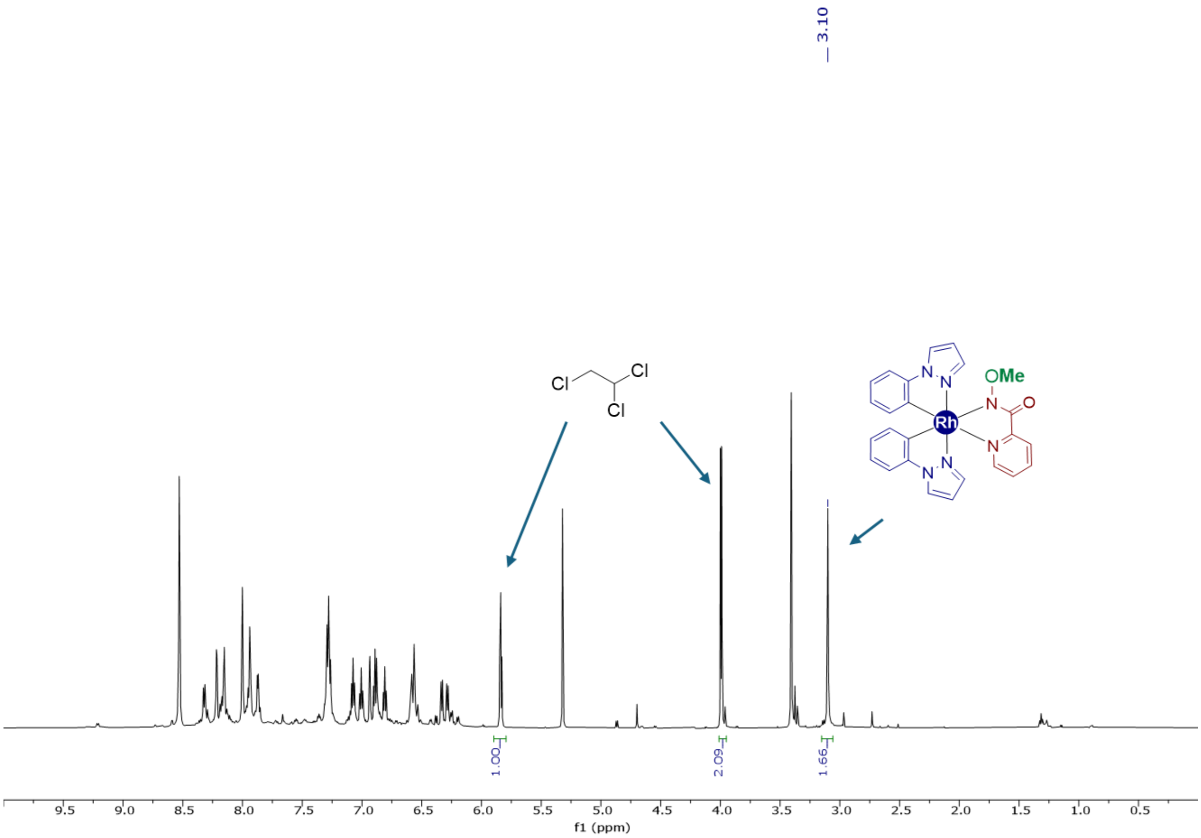

Figure S26. ^{1}H NMR of the crude reaction mixture of **Rh1a** photolysis in methanol.

5. Computational study

5-1. DFT study

- Computational details

All computational studies were conducted using the Gaussian 16[17] program package in the density functional theory (DFT) framework,[18] using High Performance Computing Resources in the Institute for Basic Science (IBS) Research Solution Center. Geometry optimizations were performed using B3LYP[19,20] functional with Grimme's D3[21] dispersion and a mixed basis set of LANL2DZ[22–24] (Rh) and 6-31G**[25–29] for all other atoms. Frequency calculations were performed at the same level as that used for geometry optimizations, wherein thermochemistry correction energy (G – E) was acquired. Transition states were verified by one imaginary frequency and confirmed by intrinsic reaction coordinate calculations (IRC).[30,31] With the optimized geometries of intermediates and transition structures, single-point energy calculations were conducted at B3LYP-D3 functional with a different basis set combination of SDD[32] (Rh) and 6-311+G**[28,33–36] for others. The solvent effect was reflected by using the SMD[37] solvation model and carried out at the same level as single-point calculations (solvent = methanol). Finally, to increase the accuracy of the integration grid, we used the *int* = *ultrafine* option for all types of calculations. The final solution phase Gibbs free energies were calculated as follows:

$$G_{sol} = E_{sol} + (G - E) \quad (1)$$

$$\Delta G(sol) = \Sigma G(sol) \text{ for products} - \Sigma G(sol) \text{ for reactants} \quad (2)$$

- Gibbs energy profile on Rh1a photolysis in methanol

As shown in Figure S27, the triplet energy of **Rh1a** in methanol is computed as 57.4 kcal/mol, where the following N–O bond cleavage to furnish a radical pair of ^{2}**RhA** and acyloxyradical is 1.3 kcal/mol. Next, while the direct N–OMe bond formation barrier is very high with 39.5 kcal/mol, that barrier from ^{1}**RhA** to form N–OMe complex ^{1}**RhB** is much lower with 8.9 kcal/mol barrier.

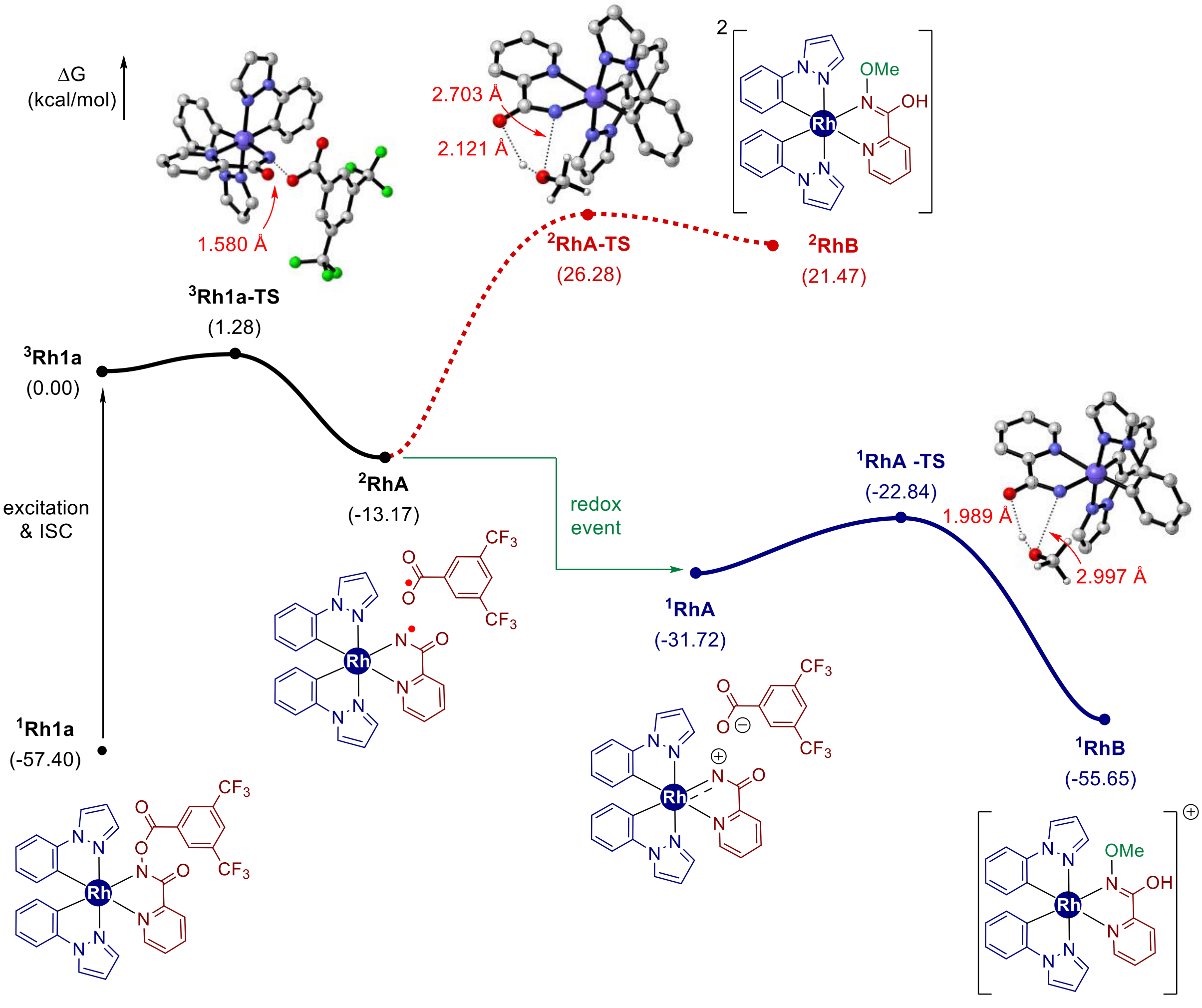

Figure S27. Gibbs energy profile on the methanol solvolysis type reaction.

- Gibbs energy profile on photoactivation of Rh1a in dichloromethane

As shown in Figure S28, the triplet energy of **Rh1a** in dichloromethane is computed as 56.3 kcal/mol. The following N–O bond cleavage barrier via ^{3}**Rh1a-TS** is 2.6 kcal/mol to form the Rh-nitrene radical species ^{2}**RhA**. The following redox event with acyloxyradical species to form ^{1}**RhA** shows the thermodynamic driving force of –8.6 kcal/mol.

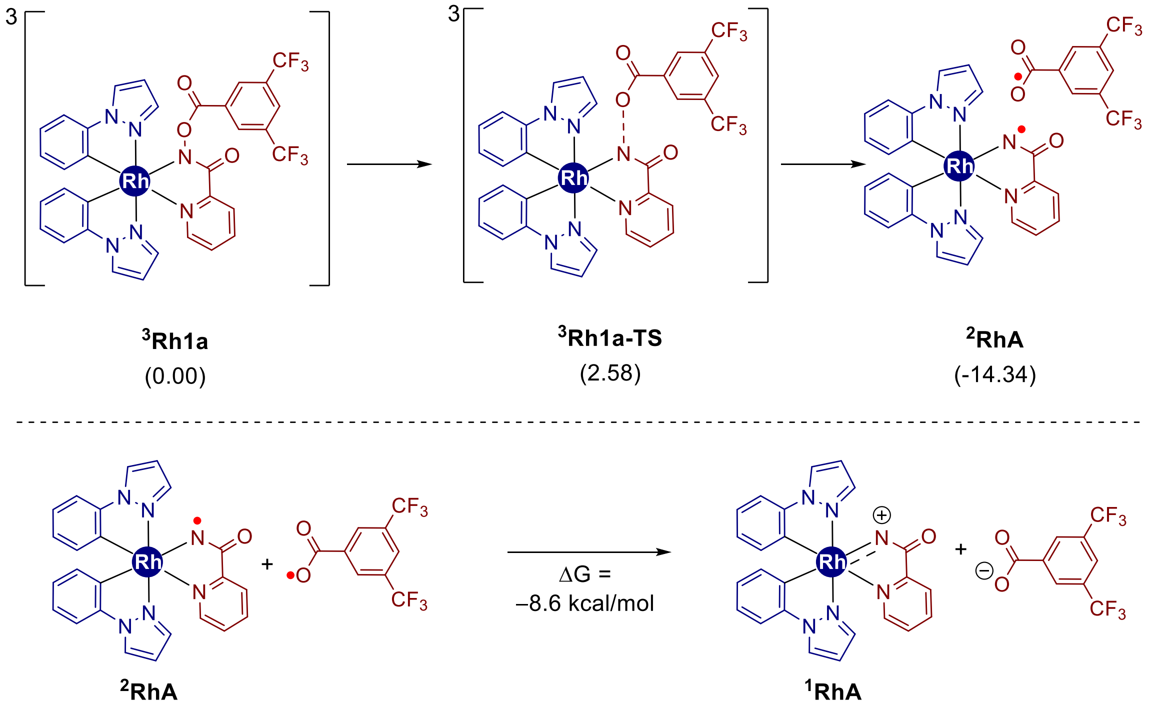

Figure S28. Photoactivation barrier of **Rh1a** in dichloromethane.

- **Frontier orbital analysis and spin density plot of ^{2}RhA**

As shown in Figure S29, computed SOMO showed π*-character of Rh-d_{xy} and N-*p* orbital (see α-HOMO). While this SOMO showed significant involvement of d_{xy} orbital of the rhodium center, β-HOMO orbital also displayed a notable proportion of the same d_{xy} orbital, rationalizing why the spin density on the rhodium center (0.090) is remarkably lower than that of nitrogen (0.857). Additionally, singly unoccupied molecular orbital (SUMO) showed a large proportion of nitrogen *p* orbital along with a small proportion of Rh-d_{yz} character. Furthermore, α-HOMO-1 showed a moderate proportion of the d_{yz} orbital, explaining the small portion of the spin density of Rh showed similar geometry to the Rh-d_{yz} orbital.

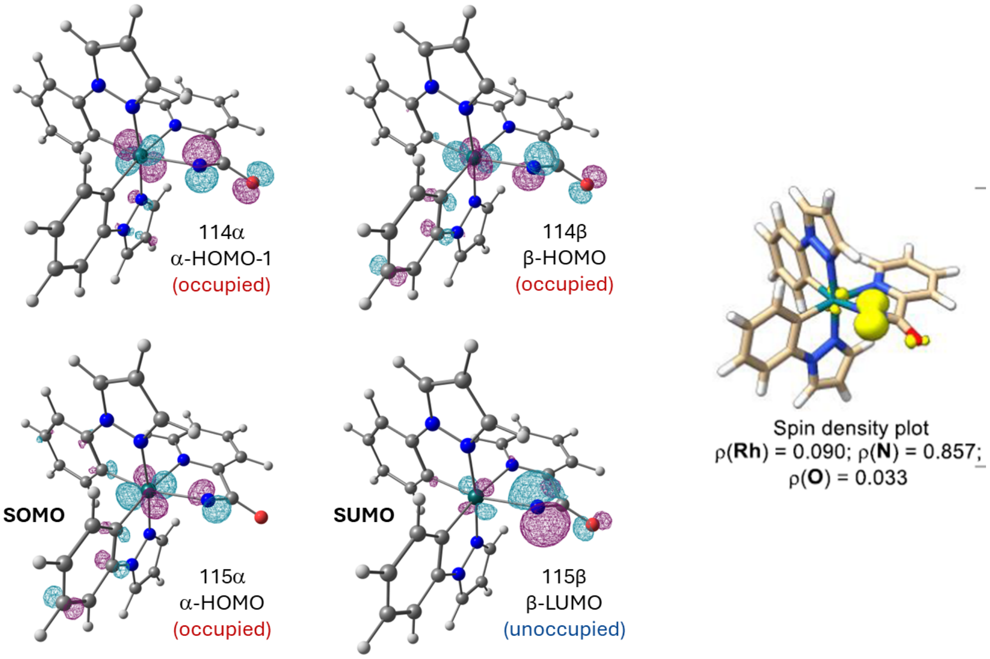

Figure S29. Frontier orbital analysis (left) and spin density plot (right) of **^{2}RhA**.

5-2. TD-DFT calculation.

Time-dependent-DFT (TD-DFT) calculations[38] were conducted using the Gaussian 16[17] suite of program using DFT optimized structure of **Rh1a, Rh3, Rh4, Rh5** at SMD(dichloromethane)-B3LYP-D3/LANL2DZ|6-31G** levels of theory. For the TD-DFT calculations, 50 eigenstates of the time-dependent Hamiltonian were constructed.

- Simulated UV-Vis spectra and key frontier orbital transitions of Rh1a

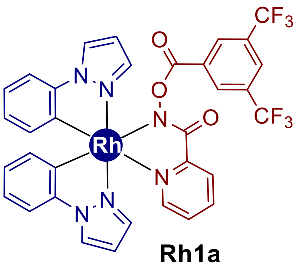

Rh1a

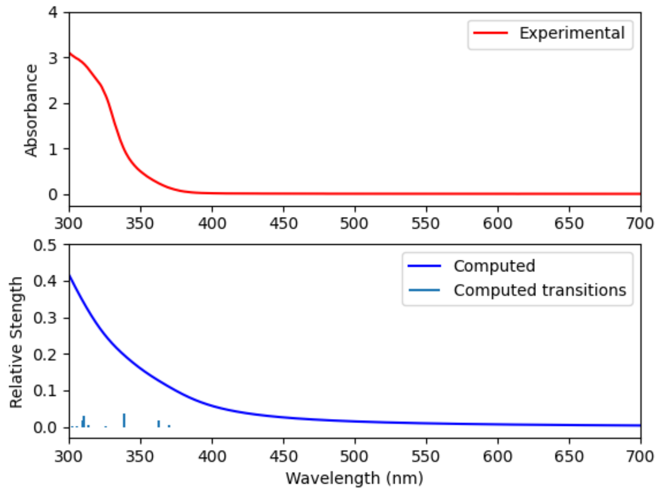

Figure S30. Experimental (upper, red line) and TDDFT simulated (down, blue line) UV–Vis spectrum of **Rh1a**.

Table S5. Frontier orbital transitions result from TDDFT simulation of **Rh1a**.

Excited State 3: Singlet-?Sym 3.6583 eV 338.91 nm f=0.0361 <S**2>=0.000<br>177 ->180 0.67447	
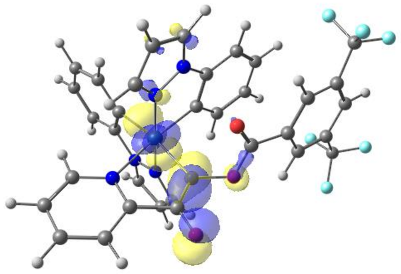<br>#177 (HOMO – 1)	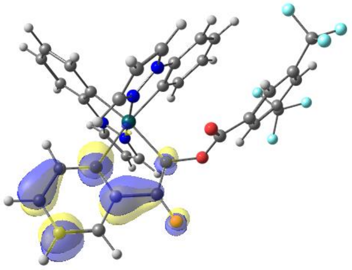<br>#180 (LUMO + 1)

- Simulated UV-Vis spectra and key frontier orbital transitions of Rh3

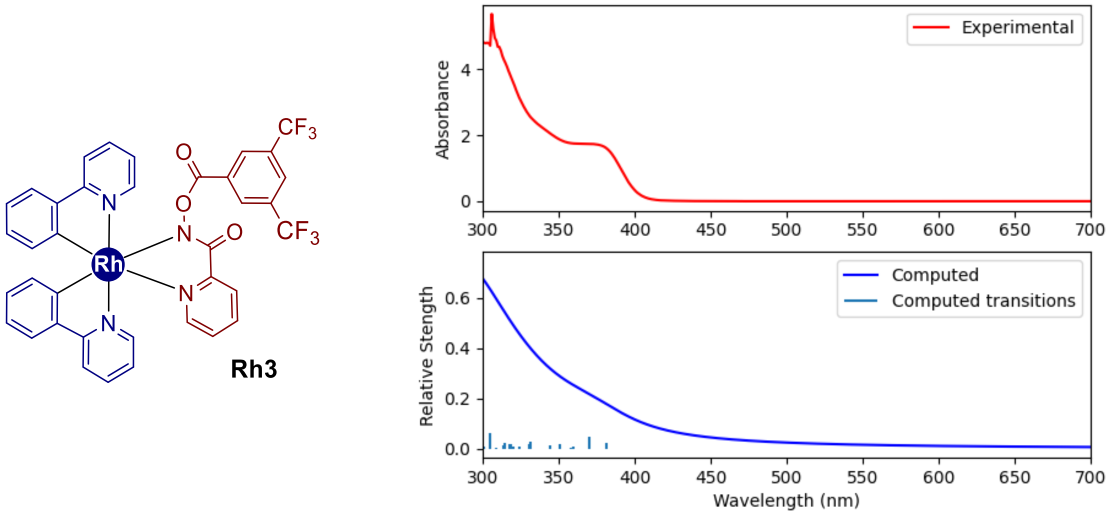

Figure S31. Experimental (upper, red line) and TDDFT simulated (down, blue line) UV–Vis spectrum of **Rh3**

Table S6. Frontier orbital transitions result from TDDFT simulation of **Rh3**

```
Excited State   2:      Singlet-?Sym    3.3517 eV  369.91 nm  f=0.0474  <S**2>=0.000
     184 ->186         0.68317

Excited State   7:      Singlet-?Sym    3.7395 eV  331.56 nm  f=0.0278  <S**2>=0.000
     183 ->186         0.10698
     183 ->187         0.56806
     183 ->188         0.36478
```

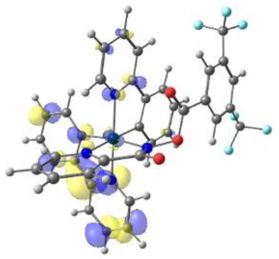 #186 (LUMO + 1)	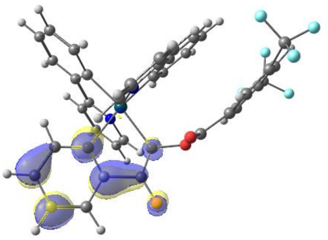 #187 (LUMO + 2)
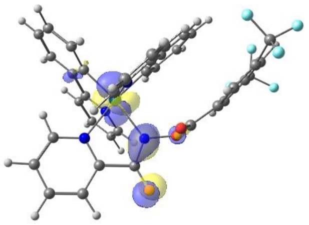 #183 (HOMO – 1)	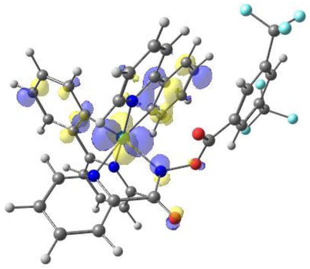 #184 (HOMO)

- Simulated UV-Vis spectra and key frontier orbital transitions of Rh4

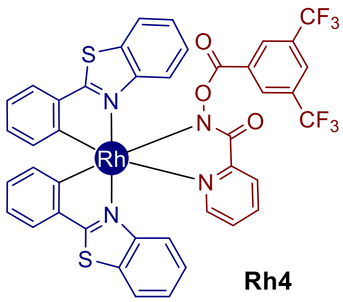

Rh4

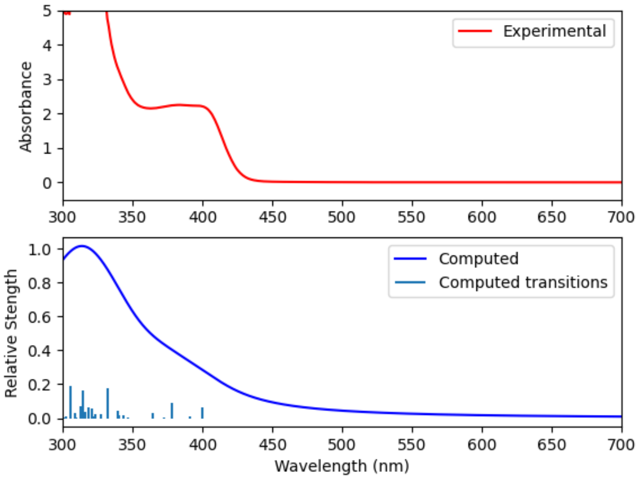

Figure S32. Experimental (upper, red line) and TDDFT simulated (down, blue line) UV–Vis spectrum of **Rh4**

Table S7. Frontier orbital transitions result from TDDFT simulation of **Rh4**

```
Excited State   3:      Singlet-?Sym    3.2802 eV  377.98 nm  f=0.0874  <S**2>=0.000
     211 ->213         0.66529
     212 ->215         0.16105


Excited State   7:      Singlet-?Sym    3.6119 eV  343.26 nm  f=0.0192  <S**2>=0.000
     210 ->213         0.11217
     211 ->215        -0.14261
     212 ->216         0.46126
     212 ->219        -0.40906
```

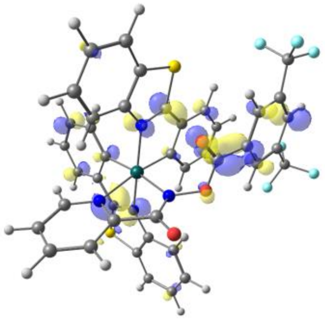

#213 (LUMO)

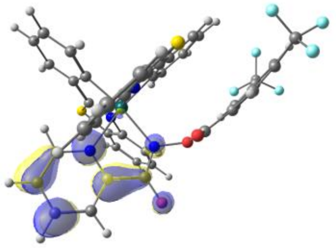

#216 (LUMO + 3)

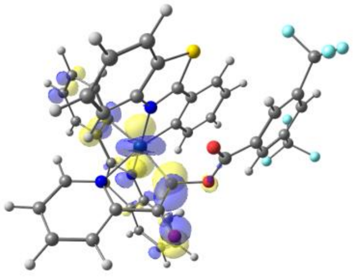

#211 (HOMO – 1)

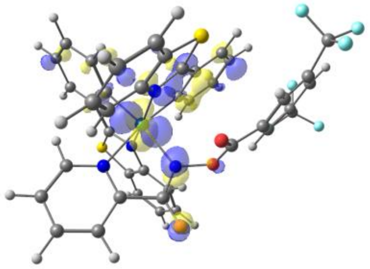

#212 (HOMO)

- Simulated UV-Vis spectra and key frontier orbital transitions of Rh5

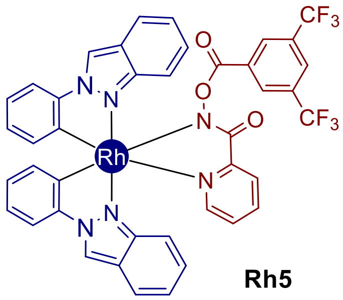

Rh5

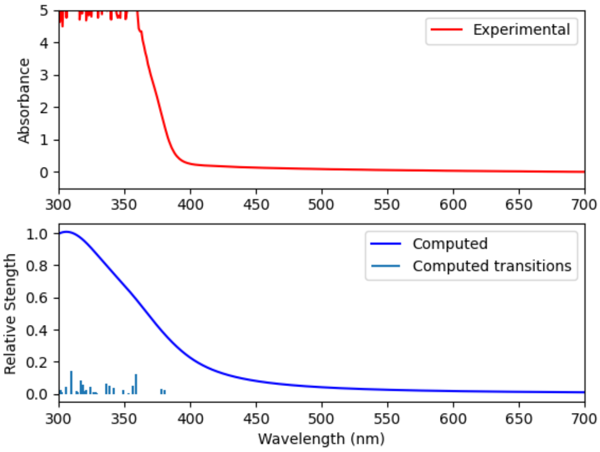

Figure S33. Experimental (upper, red line) and TDDFT simulated (down, blue line) UV–Vis spectrum of **Rh5**

Table S8. Frontier orbital transitions result from TDDFT simulation of **Rh5**

```
Excited State   3:      Singlet-?Sym    3.4519 eV  359.18 nm  f=0.1236  <S**2>=0.000
     203 ->205         0.15626
     204 ->207         0.64387
     204 ->208         0.15435

Excited state symmetry could not be determined.
 Excited State   6:      Singlet-?Sym    3.5479 eV  349.46 nm  f=0.0224  <S**2>=0.000
     203 ->206         0.10394
     204 ->207        -0.17951
     204 ->208         0.63693
```

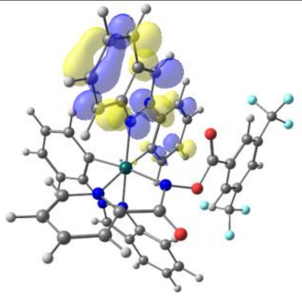

#207 (LUMO)

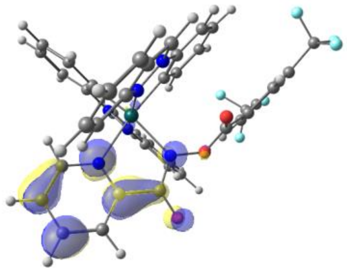

#208 (LUMO + 1)

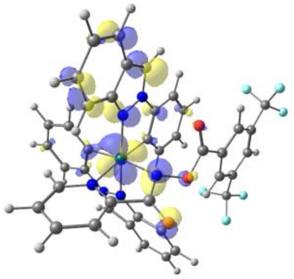

#204 (HOMO)

5-3. DFT-based electron paramagnetic resonance parameter (EPR) simulation

DFT-based electron paramagnetic resonance spectrum simulation of assumed Rh-nitrene radical ^{2}**RhA** conducted using ORCA software.[39] All EPR simulation of ^{2}**RhA** was conducted by using the optimized geometry by employing Gaussian 16[17] program package in the density functional theory (DFT) framework,[18] with B3LYP[19,20] functional with Grimme's D3[21] dispersion and a mixed basis set of LANL2DZ[22–24] (Rh) and 6-31G**[25–29] for all other atoms.

Table S9. DFT-simulated g-value and hyperfine couplings (N and Rh) using ORCA software.

Experimental	g_{iso}	A_{iso}(N)/MHz	A_{iso}(Rh)/MHz
Simulated	2.014	11.70	N/A
Functional / Basis set	g_{iso}	A_{iso}(N)	A_{iso}(Rh)
B3LYP-D3 / def2-SVP def2/J, def2-TZVP(Rh)	2.0052	83.26	0.012
B3LYP-D3 / def2-TZVP def2/J	2.0052	29.69	0.013
B3LYP-D3 / def2-TZVP def2/J (CPCM solv. w/ C_6H_6)	2.0052	26.88	0.015
B3LYP / def2-TZVP def2/J	2.0052	29.69	0.013
BP86 / def2-TZVP def2/J	2.0052	16.03	0.017
BP86 / def2-TZVP def2/J (CPCM solv. w/ C_6H_6)	2.0052	13.37	0.020
PBE0 / def2-TZVP def2/J	2.0050	29.02	0.011
TPSSh / def2-TZVP def2/J	2.0046	22.04	0.010
TPSSh / def2-TZVP def2/J (CPCM solv. w/ C_6H_6)	2.0046	19.25	0.012
B3LYP / EPR-II, def2-TZVP (Rh)	2.0038	23.13	0.030
B3LYP-D3 / EPR-II, def2-TZVP (Rh)	2.0038	23.13	0.030
BP86 / EPR-II, def2-TZVP (Rh)	2.0059	15.93	0.138
PBE0 / EPR-II, def2-TZVP (Rh)	2.0031	16.77	0.097
TPSSh / EPR-II, def2-TZVP (Rh)	2.0037	22.13	0.034

5-4. Energy components of all DFT optimized geometries

Table S10. Summarized energy components of DFT-optimized structures (in methanol).

Structure label	**E(sol)** (SCF/TZ) [eV] SMD(MeOH)-B3LYP-D3/6-311+G**, SDD(Ir)	**G–E** [eV] B3LYP-D3/6-31G**, LANL2DZ(Ir)	**G(sol)** [eV]
**^{1}Rh1a**	-68976.111	11.405	-68964.707
**^{3}Rh1a**	-68973.466	11.249	-68962.217
**^{3}Rh1a-TS**	-68973.396	11.234	-68962.162
**^{2}RhA**	-39188.063	8.739	-39179.324
**ArFCOO radical**	-29785.242	1.778	-29783.464
**^{1}RhA**	-39182.686	8.816	-39173.870
**ArFCOO$^-$**	-29791.524	1.801	-29789.722
**MeOH**	-3150.397	0.779	-3149.618
**^{1}RhA-TS**	-42333.237	10.133	-42323.103
**^{1}RhB**	-42334.786	10.260	-42324.526
**^{2}RhA-TS**	-42337.343	10.112	-42327.231
**^{2}RhB**	-42337.600	10.161	-42327.440
**^{2}RhA-TS'**	-42338.262	9.879	-42328.383
**^{1}RhC**	-39206.511	9.124	-39197.387
**MeO radical**	-3131.966	0.369	-3131.597

Table S11. Summarized energy components of DFT-optimized structures (in dichloromethane).

Structure label	**E(sol)** (SCF/TZ) [eV] SMD(MeOH)-B3LYP-D3/6-311+G**, SDD(Ir)	**G–E** [eV] B3LYP-D3/6-31G**, LANL2DZ(Ir)	**G(sol)** [eV]
**^{1}Rh1a**	-68976.246	11.405	-68964.841
**^{3}Rh1a**	-68973.650	11.249	-68962.401
**^{3}Rh1a-TS**	-68973.523	11.234	-68962.289
**^{2}RhA**	-39188.202	8.739	-39179.463
**ArFCOO radical**	-29785.337	1.778	-29783.560
**^{1}RhA**	-39182.807	8.816	-39173.991
**ArFCOO$^-$**	-29791.102	1.801	-29789.300
**^{1}Rh3**	-70178.986	12.415	-70166.571
**^{3}Rh3**	-70176.470	12.102	-70164.368
**^{1}Rh4**	-96000.515	12.955	-95987.560
**^{3}Rh4**	-95997.912	12.837	-95985.074
**^{1}Rh5**	-77340.156	13.835	-77326.320
**^{3}Rh5**	-77337.812	13.658	-77324.154

5-5. Catesian coordinates of all optimized geometries

================================
[1]Rh1a
================================
Charge = 0 Multiplicity = 1
Cartesian coordinates:

ATOM	X	Y	Z
Rh	1.088563	-0.690243	0.217747
N	0.219262	-2.319259	-0.650643
N	1.087013	-3.159772	-1.269297
C	0.419872	-4.231316	-1.768261
C	-0.921366	-4.067871	-1.457518
C	-0.998502	-2.850980	-0.754580
C	2.454870	-2.763103	-1.275659
C	2.729455	-1.565870	-0.582943
C	4.061609	-1.139344	-0.577467
H	4.322717	-0.224484	-0.052697
C	5.062249	-1.867785	-1.231596
H	6.089735	-1.514304	-1.210792
C	4.749745	-3.049425	-1.907568
H	5.525642	-3.615571	-2.413562
C	3.431741	-3.507824	-1.935408
H	3.182483	-4.423880	-2.462978
N	2.131067	0.780231	1.181420
N	2.510743	0.458541	2.442650
C	3.139255	1.508789	3.024802
C	3.174346	2.537463	2.098269
C	2.524150	2.033310	0.957845
C	2.112961	-0.818931	2.914897
C	1.429789	-1.613875	1.974241
C	0.969672	-2.857932	2.413528
H	0.423538	-3.498573	1.727870
C	1.163147	-3.276737	3.735373
H	0.753275	-4.226187	4.065320
C	1.836125	-2.457548	4.643931
H	1.957241	-2.758456	5.679063
C	2.320132	-1.214846	4.235746
H	2.804619	-0.561540	4.954478
C	1.219108	0.795963	-2.619194
C	0.789492	1.663736	-3.619805
H	1.386178	1.792098	-4.516636
C	-0.646194	1.264250	-1.308782
C	-0.412002	2.353014	-3.437589
H	-0.774448	3.037911	-4.198632
C	-1.138111	2.150129	-2.267222
H	-2.076141	2.649548	-2.052873
C	-1.441096	1.055039	-0.023104
O	-2.495816	1.665494	0.162222
N	-0.814036	0.151266	0.747737
H	2.145114	0.234076	-2.698939
N	0.517710	0.603138	-1.491461
O	-1.481433	-0.150970	1.968142
C	-0.902118	0.435119	3.043822
O	-0.206285	1.426389	3.011435
C	-1.136629	-0.365955	4.286142
C	-0.491749	0.057237	5.449810
C	-1.828569	-1.580376	4.278515
C	-0.526108	-0.736429	6.593463
C	-1.875155	-2.356557	5.435821
C	-1.219498	-1.945922	6.596303
H	0.064547	0.987105	5.429109
H	-2.305024	-1.923665	3.369537
H	-1.239218	-2.564587	7.485571
C	-2.524732	-3.712249	5.391357
F	-1.642217	-4.663966	4.991443
F	-2.985226	-4.091910	6.603131
F	-3.558513	-3.749819	4.524950
C	0.327324	-0.353683	7.768825
F	1.615647	-0.747900	7.569404
F	0.357812	0.981040	7.962871
F	-0.086676	-0.931695	8.915438
H	-1.730583	-4.737519	-1.704199
H	-1.838804	-2.332245	-0.318016
H	2.314296	2.499453	0.007303
H	3.602830	3.518352	2.232204
H	0.935519	-5.017080	-2.296945
H	3.508216	1.450179	4.036097

================================
[3]Rh1a
================================
Charge = 0 Multiplicity = 3
Cartesian coordinates:

ATOM	X	Y	Z
Rh	-1.728697	-0.178132	0.284429
N	-1.500380	-1.536325	-1.236668
N	-2.211937	-1.247349	-2.354649
C	-1.961532	-2.169546	-3.317989
C	-1.058782	-3.082119	-2.795646
C	-0.798839	-2.644116	-1.484807
C	-3.047144	-0.098946	-2.290832
C	-2.978295	0.618248	-1.082105
C	-3.789899	1.749413	-0.966796
H	-3.764773	2.333792	-0.051992
C	-4.635001	2.139147	-2.012624
H	-5.258508	3.021953	-1.901218
C	-4.679312	1.403742	-3.198865
H	-5.334059	1.707348	-4.009608
C	-3.879772	0.269650	-3.346289
H	-3.914402	-0.309079	-4.264346
N	-1.915666	1.358830	1.609038
N	-1.055766	2.388567	1.401176
C	-1.216310	3.328105	2.363398

C	-2.220010	2.889281	3.212964
C	-2.625608	1.645002	2.700425
C	-0.162978	2.258507	0.303718
C	-0.300361	1.068050	-0.432376
C	0.566024	0.885748	-1.512674
H	0.512444	-0.025188	-2.101140
C	1.544429	1.836369	-1.822771
H	2.232761	1.645594	-2.640272
C	1.665817	2.998924	-1.059712
H	2.449475	3.716785	-1.273721
C	0.803336	3.220566	0.015646
H	0.903316	4.117386	0.619445
C	-4.520972	-1.478786	1.078656
C	-5.365176	-2.487318	1.516838
H	-6.436976	-2.393213	1.384802
C	-2.612870	-2.628759	1.822456
C	-4.788106	-3.637245	2.119264
H	-5.419171	-4.451596	2.462169
C	-3.421321	-3.706422	2.262907
H	-2.925417	-4.559070	2.713672
C	-1.180876	-2.676585	1.976291
O	-0.506056	-3.622997	2.407309
N	-0.549225	-1.427825	1.565614
H	-4.907623	-0.593111	0.581320
N	-3.185571	-1.523229	1.217398
O	0.792056	-1.489867	1.684380
C	1.391862	-0.217129	1.910824
O	0.915893	0.564578	2.703881
C	2.579791	-0.030916	1.081290
C	3.367595	1.113397	1.303639
C	2.869599	-0.867934	-0.008960
C	4.426994	1.400082	0.453944
C	3.916772	-0.550008	-0.865460
C	4.709726	0.578206	-0.643124
H	3.121423	1.769996	2.128896
H	2.250093	-1.732341	-0.208927
H	5.524462	0.818186	-1.315584
C	4.079116	-1.338866	-2.133364
F	3.261548	-0.849833	-3.105624
F	5.338045	-1.284208	-2.615470
F	3.752123	-2.639138	-1.972249
C	5.212413	2.670116	0.629572
F	4.761000	3.644921	-0.202696
F	5.127645	3.155134	1.885390
F	6.520467	2.493396	0.340791
H	-0.645308	-3.945306	-3.293288
H	-0.164306	-3.063993	-0.718387
H	-3.369588	0.945255	3.050026
H	-2.598748	3.396163	4.086679
H	-2.434570	-2.112753	-4.285269
H	-0.613534	4.221834	2.379616

==============================

^{3}Rh1a-TS

==============================

Charge = 0 Multiplicity = 3

Imaginary frequency: -642.92 cm^{-1}

Cartesian coordinates:

ATOM	X	Y	Z
Rh	-1.732041	0.110003	0.437045
N	-0.954135	-1.022911	-1.092547
N	-1.458130	-0.728777	-2.320054
C	-0.806269	-1.440204	-3.275365
C	0.144738	-2.220103	-2.637237
C	0.007840	-1.928509	-1.268474
C	-2.532284	0.198086	-2.352298
C	-2.865528	0.755411	-1.105159
C	-3.931345	1.657319	-1.072609
H	-4.219179	2.110766	-0.128949
C	-4.629970	1.986316	-2.240398
H	-5.455024	2.691749	-2.194272
C	-4.271361	1.416634	-3.464104
H	-4.813795	1.672542	-4.368839
C	-3.213199	0.509052	-3.528461
H	-2.935965	0.056213	-4.475600
N	-2.466521	1.437975	1.806130
N	-1.781778	2.608329	1.865797
C	-2.279922	3.387213	2.856588
C	-3.320866	2.691041	3.452388
C	-3.398072	1.470305	2.760327
C	-0.724695	2.774403	0.929214
C	-0.510486	1.676775	0.076509
C	0.504272	1.799424	-0.874427
H	0.709195	0.976335	-1.552222
C	1.288170	2.957026	-0.949985
H	2.092046	3.016878	-1.676762
C	1.061431	4.019287	-0.072762
H	1.674569	4.913456	-0.124987
C	0.041973	3.935972	0.877408
H	-0.137624	4.760776	1.560220
C	-4.287542	-1.811579	0.500518
C	-5.129103	-2.786562	1.020461
H	-6.098275	-2.965369	0.568927
C	-2.627227	-2.263286	2.091106
C	-4.691720	-3.513017	2.148917
H	-5.327186	-4.277511	2.587085
C	-3.447967	-3.251906	2.685653
H	-3.057493	-3.780959	3.547453
C	-1.331791	-1.942617	2.655666
O	-0.849730	-2.456621	3.700243
N	-0.660558	-0.893621	2.012732
H	-4.580033	-1.203547	-0.350665
N	-3.080320	-1.538371	1.018236
O	0.825170	-1.352275	1.734583
C	1.636700	-0.271211	1.707496
O	1.463131	0.746613	2.343008

C	2.760112	-0.437211	0.726425
C	3.441935	0.717456	0.325002
C	3.078351	-1.665318	0.145734
C	4.395165	0.642467	-0.685631
C	4.035229	-1.729757	-0.869441
C	4.692234	-0.579220	-1.297556
H	3.184253	1.662489	0.786452
H	2.580095	-2.567324	0.481357
H	5.429162	-0.631562	-2.090011
C	4.274393	-3.055581	-1.537752
F	4.480997	-4.038541	-0.637237
F	3.189692	-3.428000	-2.271676
F	5.330253	-3.029106	-2.374778
C	5.041574	1.898888	-1.203416
F	4.377920	2.374395	-2.291423
F	5.047797	2.884403	-0.285242
F	6.316170	1.683026	-1.594153
H	0.855071	-2.894286	-3.088400
H	0.535603	-2.304854	-0.409752
H	-4.051039	0.621546	2.897698
H	-3.933336	3.019563	4.277443
H	-1.062508	-1.341624	-4.318228
H	-1.864014	4.359285	3.067729

==============================

^{2}RhA

==============================

Charge = 0 Multiplicity = 2

Cartesian coordinates:

ATOM	X	Y	Z
Rh	1.275673	-0.844762	0.220498
N	0.408615	-2.490615	-0.615046
N	1.218135	-3.215854	-1.427848
C	0.556266	-4.310115	-1.883997
C	-0.719762	-4.278891	-1.342898
C	-0.765211	-3.115558	-0.550389
C	2.531738	-2.704459	-1.628782
C	2.830264	-1.535044	-0.901445
C	4.106655	-0.993703	-1.081510
H	4.381463	-0.092364	-0.539826
C	5.035090	-1.590530	-1.942734
H	6.021880	-1.151333	-2.063569
C	4.701720	-2.750011	-2.647022
H	5.421149	-3.213487	-3.315101
C	3.435918	-3.318421	-2.495051
H	3.170232	-4.217151	-3.044157
N	2.313203	0.664718	1.131623
N	2.934959	0.294155	2.279440
C	3.572473	1.357269	2.830136
C	3.351589	2.448404	2.004685
C	2.554114	1.964700	0.952792
C	2.788387	-1.065548	2.668168
C	2.002900	-1.849790	1.802670
C	1.833129	-3.193434	2.146424
H	1.233476	-3.836757	1.509988
C	2.415375	-3.721364	3.303474
H	2.265717	-4.768616	3.552137
C	3.186747	-2.913395	4.142232
H	3.637616	-3.324402	5.040159
C	3.379383	-1.568560	3.826637
H	3.976302	-0.934081	4.475046
C	0.886880	0.698994	-2.609837
C	0.300149	1.644240	-3.447391
H	0.676647	1.780652	-4.455636
C	-0.583249	1.217970	-0.885034
C	-0.763434	2.409467	-2.957965
H	-1.233742	3.160250	-3.586676
C	-1.205611	2.200158	-1.654307
H	-2.004892	2.772063	-1.196266
C	-0.992933	0.967193	0.570853
O	-1.595258	1.866298	1.177853
N	-0.625387	-0.247953	0.991626
H	1.731569	0.091776	-2.921468
N	0.448488	0.492393	-1.359067
H	-1.507435	-4.999721	-1.498667
H	-1.547899	-2.688437	0.059235
H	2.137416	2.474385	0.097600
H	3.710897	3.455356	2.148799
H	1.030849	-5.017566	-2.545245
H	4.121758	1.264914	3.753239

==============================

ArFCOO radical

==============================

Charge = 0 Multiplicity = 2

Cartesian coordinates:

ATOM	X	Y	Z
O	-0.058250	-0.878817	2.780795
C	-0.343107	0.119149	3.502382
O	0.266102	1.159869	3.124081
C	-1.273647	0.074070	4.648685
C	-1.524164	1.238477	5.380357
C	-1.900588	-1.129920	4.981698
C	-2.412268	1.191565	6.452389
C	-2.786446	-1.162356	6.056333
C	-3.045546	-0.005156	6.792785
H	-1.038080	2.168194	5.104908
H	-1.704489	-2.023718	4.399257
H	-3.750514	-0.031583	7.615724
C	-3.432764	-2.463635	6.463124
F	-2.694347	-3.099069	7.398064
F	-4.658447	-2.260962	6.990069
F	-3.562660	-3.301112	5.414064
C	-2.656597	2.424958	7.286401
F	-1.819199	2.466931	8.344783
F	-2.461756	3.552390	6.572685

F	-3.915956	2.449631	7.770890

==============================

[1]RhA

==============================

Charge = 1 Multiplicity = 1

Cartesian coordinates:

ATOM	X	Y	Z
Rh	1.396056	-1.225470	0.171137
N	0.557821	-2.769038	-0.919802
N	1.337576	-3.269276	-1.912028
C	0.704861	-4.305462	-2.521825
C	-0.517717	-4.470257	-1.892862
C	-0.568531	-3.481787	-0.892266
C	2.574091	-2.616563	-2.113809
C	2.839698	-1.560059	-1.228205
C	4.022854	-0.837114	-1.375602
H	4.244050	-0.009603	-0.709868
C	4.937807	-1.194191	-2.373779
H	5.867448	-0.642629	-2.473287
C	4.660792	-2.256779	-3.237597
H	5.370103	-2.526283	-4.013067
C	3.471772	-2.978234	-3.116936
H	3.256970	-3.799122	-3.793245
N	2.417967	0.151178	1.291481
N	3.069858	-0.390802	2.352196
C	3.659774	0.586230	3.083335
C	3.375672	1.797304	2.469696
C	2.593625	1.476303	1.350314
C	2.993843	-1.801404	2.483593
C	2.225587	-2.464760	1.504871
C	2.123167	-3.855993	1.569731
H	1.539641	-4.393989	0.831879
C	2.760823	-4.559302	2.594734
H	2.671342	-5.639843	2.642670
C	3.511876	-3.879687	3.557528
H	4.006050	-4.430035	4.351204
C	3.636784	-2.489954	3.508335
H	4.223564	-1.967263	4.256349
C	1.178941	1.007264	-2.326236
C	0.619616	2.125969	-2.946376
H	1.088722	2.536448	-3.833826
C	-0.490523	1.011119	-0.722688
C	-0.534684	2.699841	-2.408151
H	-0.982546	3.573053	-2.871842
C	-1.104849	2.130656	-1.268399
H	-1.999129	2.520456	-0.793684
C	-1.005187	0.307362	0.497753
O	-1.869741	0.753158	1.257144
N	-0.446208	-0.894968	0.727090
H	2.084074	0.538359	-2.697076
N	0.629642	0.463717	-1.232390
H	-1.272980	-5.204671	-2.126001
H	-1.331341	-3.242178	-0.166825
H	2.152167	2.114348	0.599853
H	3.690979	2.777389	2.791882
H	1.159047	-4.837302	-3.343191
H	4.225676	0.360307	3.973433

==============================

ArFCOO$^-$

==============================

Charge = -1 Multiplicity = 1

Cartesian coordinates:

ATOM	X	Y	Z
O	3.165866	0.200103	-0.988244
C	4.382559	-0.009238	-0.767880
O	5.179768	0.528799	0.036928
C	5.013269	-1.131567	-1.647011
C	4.240714	-1.819705	-2.585735
C	6.360436	-1.473730	-1.506550
C	4.803788	-2.832691	-3.367660
C	6.924030	-2.486652	-2.288121
C	6.151069	-3.173034	-3.227374
H	3.195436	-1.538252	-2.666677
H	6.933625	-0.928067	-0.763553
H	6.584073	-3.967464	-3.823173
C	3.971887	-3.522287	-4.405999
F	4.454399	-4.753565	-4.726124
F	2.687954	-3.693150	-4.012015
F	3.921456	-2.824321	-5.572695
C	8.383350	-2.802523	-2.160235
F	8.829306	-2.686670	-0.887253
F	8.680262	-4.065661	-2.569793
F	9.157730	-1.973272	-2.910990

==============================

MeOH

==============================

Charge = 0 Multiplicity = 1

Cartesian coordinates:

ATOM	X	Y	Z
C	-2.441100	0.028836	-0.626156
H	-3.494208	0.058943	-0.304542
H	-2.005379	-0.903398	-0.255570
H	-1.910898	0.864200	-0.141688
O	-2.293346	0.023639	-2.036482
H	-2.674988	0.841281	-2.377999

==============================

[1]RhA-TS

==============================

Charge = 1 Multiplicity = 1

Imaginary frequency: -77.66 cm^{-1}

Cartesian coordinates:

ATOM X Y Z

Rh	0.923909	-0.271924	-0.020821
N	-0.256529	-1.712573	-0.861525
N	0.376909	-2.451004	-1.813678
C	-0.491175	-3.352904	-2.343942
C	-1.708682	-3.181027	-1.708239
C	-1.518009	-2.140028	-0.778442
C	1.746429	-2.164398	-2.030681
C	2.295366	-1.127917	-1.246930
C	3.634975	-0.785870	-1.426234
H	4.070747	0.020387	-0.846228
C	4.421251	-1.485406	-2.348562
H	5.467585	-1.225199	-2.473527
C	3.863669	-2.519478	-3.105106
H	4.474171	-3.059799	-3.821108
C	2.520734	-2.869521	-2.950771
H	2.095891	-3.676102	-3.539199
N	2.275834	1.014615	0.848896
N	2.778709	0.562552	2.025500
C	3.618051	1.488175	2.560123
C	3.649393	2.567521	1.694393
C	2.785978	2.227445	0.636468
C	2.305857	-0.695508	2.461415
C	1.371642	-1.319516	1.606963
C	0.838030	-2.555235	1.984569
H	0.113634	-3.050964	1.349742
C	1.232448	-3.148175	3.185838
H	0.815286	-4.107219	3.475683
C	2.161348	-2.512902	4.016167
H	2.464218	-2.978198	4.948366
C	2.706631	-1.277447	3.660696
H	3.421511	-0.786727	4.312689
C	1.012040	1.103954	-3.123281
C	0.451905	1.826176	-4.177994
H	0.974912	1.882870	-5.126317
C	-0.774729	1.635937	-1.752870
C	-0.779051	2.459447	-3.992126
H	-1.237328	3.019838	-4.800902
C	-1.410079	2.362969	-2.751060
H	-2.367640	2.823442	-2.533515
C	-1.363347	1.462793	-0.379204
O	-2.537070	1.755832	-0.092274
N	-0.474813	0.983121	0.491225
H	1.957607	0.581459	-3.225110
N	0.406677	1.013485	-1.932414
O	-2.606340	-0.473747	1.587682
H	-2.615374	-3.738348	-1.885670
H	-2.178673	-1.690670	-0.043464
H	2.511837	2.782397	-0.248083
H	4.216067	3.477935	1.812414
H	-0.186325	-4.039772	-3.117904
H	4.119999	1.317743	3.499490
C	-2.248071	-0.383906	2.961193
H	-2.863646	0.405714	1.255206
H	-1.495646	0.395216	3.139495
H	-1.816382	-1.346086	3.250071
H	-3.120672	-0.189688	3.599353

================================

^{1}RhB

================================

Charge = 1 Multiplicity = 1

Cartesian coordinates:

ATOM	X	Y	Z
Rh	1.199329	-1.543476	0.358652
N	0.414551	-2.972469	-0.879779
N	1.286818	-3.432830	-1.813402
C	0.714805	-4.427273	-2.537081
C	-0.570226	-4.610570	-2.050050
C	-0.712937	-3.676900	-1.009393
C	2.568200	-2.818906	-1.832440
C	2.785152	-1.860021	-0.824629
C	4.025678	-1.219977	-0.799546
H	4.236533	-0.479761	-0.034443
C	5.006147	-1.524822	-1.752026
H	5.965797	-1.017863	-1.716815
C	4.760638	-2.478913	-2.741225
H	5.522653	-2.714563	-3.476688
C	3.530385	-3.135685	-2.789843
H	3.334405	-3.874190	-3.560779
N	2.198354	-0.277538	1.625592
N	2.907027	-0.911918	2.596136
C	3.578310	-0.007884	3.351816
C	3.290665	1.252042	2.849247
C	2.422531	1.032359	1.767167
C	2.810765	-2.330382	2.625583
C	2.019341	-2.895057	1.608239
C	1.893692	-4.286152	1.592895
H	1.300168	-4.766487	0.821669
C	2.518382	-5.073265	2.567658
H	2.405195	-6.152939	2.540812
C	3.288233	-4.480845	3.570823
H	3.772151	-5.092869	4.324704
C	3.440825	-3.094527	3.606750
H	4.038584	-2.630605	4.385036
C	0.887016	0.686961	-1.927188
C	0.260065	1.691996	-2.665977
H	0.777110	2.146313	-3.503868
C	-0.952312	0.476946	-0.534473
C	-1.027630	2.087342	-2.307522
H	-1.543326	2.863745	-2.863151
C	-1.649873	1.470522	-1.221477
H	-2.649477	1.743153	-0.905306
C	-1.541674	-0.215625	0.626201
O	-2.806572	0.083368	0.917940
N	-0.825233	-1.074239	1.285380
H	1.887020	0.337714	-2.164200

N	0.294761	0.090991	-0.885091
H	-1.300755	-5.323510	-2.399299
H	-1.544967	-3.485557	-0.348212
H	1.960692	1.734606	1.089607
H	3.660061	2.196964	3.216164
H	1.249882	-4.925833	-3.329745
H	4.201721	-0.315840	4.176205
C	-0.791882	-2.094869	3.395030
H	-1.500770	-2.511680	4.111536
H	-0.072264	-2.853331	3.081900
H	-0.271826	-1.236552	3.832019
O	-1.601727	-1.681189	2.280227
H	-3.045108	-0.420750	1.718740

==============================

[2]RhA-TS

==============================

Charge = 0 Multiplicity = 2

Imaginary frequency: -519.41 cm^{-1}

Cartesian coordinates:

ATOM	X	Y	Z
Rh	1.206263	-1.012840	0.225010
N	0.221354	-2.461422	-0.808019
N	0.959630	-3.077334	-1.767844
C	0.227248	-4.058351	-2.354776
C	-1.017956	-4.067071	-1.746143
C	-0.975245	-3.046803	-0.775203
C	2.297116	-2.618818	-1.928368
C	2.693768	-1.616806	-1.019724
C	4.000479	-1.136673	-1.148977
H	4.351477	-0.364536	-0.469229
C	4.863207	-1.628178	-2.136530
H	5.874636	-1.237918	-2.215012
C	4.432273	-2.619820	-3.020487
H	5.099792	-3.001536	-3.786984
C	3.134381	-3.124707	-2.921871
H	2.792203	-3.891799	-3.610590
N	2.365231	0.274749	1.316477
N	2.974032	-0.298751	2.384369
C	3.709976	0.623547	3.054619
C	3.565896	1.832036	2.391311
C	2.711101	1.562562	1.307029
C	2.736948	-1.687747	2.578744
C	1.898220	-2.287961	1.619905
C	1.655375	-3.657502	1.768659
H	1.013274	-4.165005	1.055345
C	2.208084	-4.380843	2.830554
H	1.998178	-5.442863	2.926505
C	3.025755	-3.748319	3.770588
H	3.452555	-4.309739	4.595949
C	3.298356	-2.385963	3.647657
H	3.934368	-1.887911	4.373616
C	1.072555	1.108049	-2.182984
C	0.551442	2.191465	-2.885357
H	1.073124	2.579590	-3.752849
C	-0.704031	1.124472	-0.661600
C	-0.657978	2.753925	-2.438361
H	-1.097786	3.595739	-2.965806
C	-1.290079	2.224134	-1.327301
H	-2.226677	2.620164	-0.953169
C	-1.261294	0.528575	0.528706
O	-2.538001	0.872976	0.853279
N	-0.569180	-0.312160	1.258172
H	2.001238	0.627384	-2.477798
N	0.475222	0.589072	-1.104196
H	-1.843692	-4.724182	-1.971512
H	-1.692606	-2.707471	-0.038563
H	2.328612	2.211429	0.533772
H	4.014787	2.776340	2.657589
H	0.637844	-4.668164	-3.143807
H	4.271004	0.360173	3.936851
C	-1.413375	-1.863203	3.077164
H	-2.190409	-2.543223	3.456528
H	-0.457492	-2.401737	3.061419
H	-1.323353	-1.017688	3.775526
O	-1.809596	-1.451798	1.791421
H	-2.775114	0.173947	1.499587

==============================

[2]RhB

==============================

Charge = 0 Multiplicity = 2

Cartesian coordinates:

ATOM	X	Y	Z
Rh	1.174437	-1.040081	0.226137
N	0.201574	-2.450974	-0.862442
N	0.953076	-3.043309	-1.824342
C	0.218885	-3.982640	-2.473550
C	-1.044733	-3.989157	-1.903555
C	-1.007561	-3.009516	-0.892776
C	2.299508	-2.595112	-1.937765
C	2.679503	-1.614011	-0.999827
C	3.993645	-1.144416	-1.083066
H	4.332369	-0.386953	-0.381367
C	4.878249	-1.625846	-2.055598
H	5.894347	-1.242470	-2.099673
C	4.463779	-2.596976	-2.969737
H	5.148961	-2.970321	-3.724608
C	3.159515	-3.091326	-2.916379
H	2.829849	-3.842700	-3.628062
N	2.326332	0.217138	1.353610
N	2.960018	-0.383733	2.392523
C	3.714437	0.522186	3.066107
C	3.556199	1.745677	2.434595
C	2.674367	1.503438	1.364447
C	2.716398	-1.775234	2.564256

C	1.859570	-2.351497	1.604596
C	1.595118	-3.718073	1.745052
H	0.940588	-4.207965	1.029843
C	2.145081	-4.462205	2.794452
H	1.919134	-5.521755	2.882049
C	2.982820	-3.852877	3.732020
H	3.408808	-4.429648	4.547221
C	3.276063	-2.493132	3.620989
H	3.925554	-2.013047	4.347245
C	1.075158	1.127704	-2.113401
C	0.531292	2.159130	-2.865276
H	1.079628	2.569051	-3.705551
C	-0.802033	1.040920	-0.694576
C	-0.756969	2.644329	-2.505655
H	-1.217090	3.446266	-3.076070
C	-1.416214	2.093114	-1.434299
H	-2.398321	2.436033	-1.128243
C	-1.393120	0.427442	0.430076
O	-2.696943	0.684344	0.715367
N	-0.659924	-0.386447	1.216984
H	2.050463	0.709498	-2.349297
N	0.455367	0.571092	-1.062647
H	-1.877638	-4.616194	-2.181533
H	-1.753753	-2.683517	-0.183147
H	2.275167	2.166543	0.611834
H	4.014286	2.682645	2.711141
H	0.639300	-4.566697	-3.276573
H	4.297634	0.237421	3.927184
C	-1.085698	-1.418359	3.268950
H	-1.796850	-2.087835	3.761597
H	-0.089152	-1.869229	3.277202
H	-1.055560	-0.453044	3.790417
O	-1.558146	-1.264770	1.933924
H	-2.902923	0.089774	1.458365

==============================

^{2}RhA-TS'

==============================

Charge = 0 Multiplicity = 2

Imaginary frequency: -1690.65 cm^{-1}

Cartesian coordinates:

ATOM	X	Y	Z
Rh	1.207478	-0.916130	0.168931
N	0.191697	-2.319508	-0.894592
N	0.927525	-2.951019	-1.846583
C	0.178582	-3.912440	-2.445086
C	-1.074251	-3.892253	-1.852838
C	-1.019213	-2.877130	-0.878029
C	2.280426	-2.529253	-1.985774
C	2.698926	-1.548536	-1.062338
C	4.021135	-1.107922	-1.173717
H	4.388728	-0.354469	-0.481645
C	4.879563	-1.616884	-2.156435
H	5.903676	-1.258342	-2.219919
C	4.427525	-2.586541	-3.053876
H	5.091266	-2.982452	-3.816407
C	3.113361	-3.051565	-2.974409
H	2.755984	-3.801422	-3.674228
N	2.394679	0.312598	1.298135
N	2.938465	-0.292338	2.383203
C	3.704401	0.587559	3.076186
C	3.648604	1.801475	2.409871
C	2.812646	1.579019	1.300732
C	2.610633	-1.662519	2.574308
C	1.783235	-2.220174	1.581601
C	1.432281	-3.564671	1.733842
H	0.784976	-4.034622	1.001644
C	1.878012	-4.306110	2.831941
H	1.583617	-5.347158	2.933364
C	2.690104	-3.717801	3.805242
H	3.030285	-4.293888	4.660160
C	3.064935	-2.380272	3.680877
H	3.691234	-1.914396	4.435880
C	1.157238	1.274390	-2.202400
C	0.597227	2.314732	-2.938920
H	1.148817	2.749804	-3.765613
C	-0.714681	1.148355	-0.829182
C	-0.680815	2.768437	-2.597463
H	-1.146149	3.573018	-3.159533
C	-1.351341	2.168715	-1.535128
H	-2.354862	2.445875	-1.232959
C	-1.424354	0.416525	0.308625
O	-2.661357	0.457733	0.345255
N	-0.564460	-0.205677	1.137633
H	2.136256	0.862132	-2.429427
N	0.513699	0.713028	-1.167117
H	-1.913380	-4.527987	-2.089004
H	-1.750919	-2.539766	-0.157605
H	2.491129	2.249853	0.518617
H	4.141400	2.719428	2.690142
H	0.583863	-4.531193	-3.229883
H	4.222775	0.293646	3.974677
C	-1.179991	-2.072928	3.606822
H	-1.529488	-1.219869	4.211525
H	-1.599681	-2.988326	4.054664
H	-0.085299	-2.150442	3.688954
O	-1.631559	-2.027730	2.284154
H	-1.141443	-1.009106	1.816416

==============================

^{1}RhC

==============================

Charge = 0 Multiplicity = 1

Cartesian coordinates:

ATOM	X	Y	Z
Rh	1.166844	-0.933109	0.151592

N	0.191980	-2.334238	-0.963752
N	0.967054	-2.981398	-1.871415
C	0.228976	-3.906068	-2.536841
C	-1.062710	-3.845594	-2.037031
C	-1.036795	-2.841839	-1.050464
C	2.328873	-2.569405	-1.951052
C	2.698231	-1.564121	-1.034136
C	4.025889	-1.126500	-1.096698
H	4.361351	-0.353910	-0.409428
C	4.929809	-1.660647	-2.022713
H	5.955391	-1.301384	-2.048021
C	4.522795	-2.656498	-2.913668
H	5.222964	-3.072863	-3.631433
C	3.206713	-3.120580	-2.883931
H	2.883732	-3.891779	-3.577372
N	2.319352	0.305555	1.296522
N	2.891079	-0.297551	2.368761
C	3.632167	0.597429	3.069959
C	3.530483	1.818459	2.422654
C	2.694125	1.584906	1.315715
C	2.605733	-1.679736	2.543114
C	1.768026	-2.239694	1.559248
C	1.463791	-3.597943	1.693038
H	0.823287	-4.075794	0.957616
C	1.966823	-4.351560	2.758959
H	1.713753	-5.405315	2.841114
C	2.792926	-3.760848	3.718285
H	3.182599	-4.346442	4.545202
C	3.119918	-2.408704	3.614999
H	3.760556	-1.942274	4.357693
C	1.005380	1.108317	-2.306862
C	0.447722	2.133753	-3.065297
H	0.940264	2.463169	-3.974116
C	-0.718880	1.222603	-0.746271
C	-0.746228	2.715827	-2.629574
H	-1.206871	3.517601	-3.199745
C	-1.335237	2.253633	-1.456536
H	-2.258656	2.652477	-1.052077
C	-1.341849	0.708058	0.547315
O	-2.401849	1.226421	0.934779
N	-0.631078	-0.275514	1.108845
H	1.930189	0.615037	-2.591241
N	0.433214	0.667581	-1.175243
H	-1.905717	-4.445059	-2.343950
H	-1.810235	-2.454325	-0.404651
H	2.343022	2.256170	0.546901
H	3.993391	2.748505	2.713813
H	0.665847	-4.527475	-3.302243
H	4.164882	0.308157	3.961571
H	-1.037867	-0.588716	1.985241

==============================

MeO radical

==============================

Charge = 0 Multiplicity = 2

Cartesian coordinates:

ATOM	X	Y	Z
C	-1.174715	-2.060072	3.598426
H	-1.541602	-1.220829	4.213939
H	-1.603328	-2.982636	4.044078
H	-0.078691	-2.158980	3.682699
O	-1.627681	-2.036777	2.306977

==============================

[1]Rh3

==============================

Charge = 0 Multiplicity = 1

Cartesian coordinates:

ATOM	X	Y	Z
H	-1.987814	-4.042515	-3.111474
H	-4.432698	-0.910152	-7.712853
H	-6.049162	-4.125217	-5.325311
H	-3.923974	-4.130102	-4.045959
H	-1.175496	4.168295	-4.394033
H	0.606212	4.061016	-5.580306
H	2.601613	3.725810	-7.012972
H	1.047822	-0.155834	-8.040564
H	-4.866864	2.231653	-3.400986
H	1.735609	-1.926076	-2.897248
C	-1.746720	-2.411202	-4.524113
C	-1.336885	-3.252215	-3.475772
C	-0.085369	-3.080428	-2.892699
C	0.759346	-2.065263	-3.355427
C	0.355903	-1.227009	-4.398148
C	-4.383224	-1.643828	-6.915846
C	-5.423550	-2.518973	-6.629015
C	-5.256760	-3.428120	-5.581172
C	-4.066707	-3.433617	-4.863544
C	-3.046677	-2.528608	-5.191839
N	-3.236497	-1.650476	-6.217381
C	-0.895662	-1.382200	-5.006702
C	-2.308920	1.056989	-5.329660
C	-3.532317	1.092788	-4.651312
C	-3.900941	2.210275	-3.898483
C	-3.043430	3.310758	-3.791891
C	-1.818696	3.295439	-4.449764
C	-1.449376	2.179131	-5.219843
C	-0.214713	2.110994	-6.001330
C	0.747970	3.125199	-6.106857
C	1.857659	2.940205	-6.921400
C	1.999947	1.743694	-7.627244
C	1.008875	0.780785	-7.497753
N	-0.060967	0.955948	-6.709176
Rh	-1.648066	-0.368032	-6.572370
H	-4.216010	0.252541	-4.718844
H	-3.347375	4.184720	-3.225477

H	2.846436	1.564058	-8.279967
H	1.025426	-0.441891	-4.737219
H	-6.336680	-2.482453	-7.212161
H	0.230625	-3.729875	-2.081796
C	-0.291470	-2.891612	-8.043300
C	0.015462	-3.729394	-9.112331
H	0.590160	-4.633705	-8.942530
C	-1.435775	-1.416695	-9.430493
C	-0.427894	-3.375502	-10.389135
H	-0.202611	-4.005723	-11.244543
C	-1.161377	-2.203176	-10.549488
H	-1.531439	-1.858163	-11.508262
C	-2.215040	-0.118328	-9.614936
O	-2.490245	0.282783	-10.746208
N	-2.511776	0.405218	-8.409058
H	0.026341	-3.110442	-7.028094
N	-0.996435	-1.759980	-8.200145
O	-3.127380	1.694102	-8.457913
C	-2.224521	2.703883	-8.524106
O	-1.105957	2.611959	-8.978941
C	-2.723022	3.924937	-7.816177
C	-1.803900	4.957226	-7.620684
C	-3.967540	3.966990	-7.178691
C	-2.116961	6.010969	-6.764630
C	-4.278449	5.039609	-6.346981
C	-3.355487	6.063653	-6.128147
H	-0.830665	4.886427	-8.091778
H	-4.655716	3.139443	-7.284668
H	-3.582692	6.868878	-5.439322
C	-5.596530	5.085615	-5.621826
F	-5.436330	5.501865	-4.341129
F	-6.458191	5.949350	-6.205389
F	-6.199678	3.879695	-5.582972
C	-1.038322	6.988106	-6.395195
F	-0.239271	6.464782	-5.416928
F	-0.230417	7.277133	-7.434524
F	-1.531286	8.146341	-5.913428

==============================

^{3}Rh3

==============================

Charge = 0 Multiplicity = 3

Cartesian coordinates:

ATOM	X	Y	Z
H	-1.432813	-5.211519	-4.121223
H	-4.538406	-0.600488	-6.590651
H	-5.792954	-4.585297	-5.529619
H	-3.483325	-5.017797	-4.736039
H	-0.270724	2.750779	-2.200551
H	1.260536	3.117372	-3.678466
H	2.914343	3.374141	-5.510562
H	0.772621	0.333489	-7.686234
H	-3.778170	0.475366	-1.244512
H	2.365097	-3.247488	-3.794541
C	-1.373387	-3.155823	-4.813902
C	-0.821438	-4.324935	-4.264791
C	0.520926	-4.359110	-3.900101
C	1.316775	-3.223207	-4.081302
C	0.773511	-2.056079	-4.625642
C	-4.359945	-1.591748	-6.190164
C	-5.358526	-2.555301	-6.127672
C	-5.039025	-3.806667	-5.595101
C	-3.745756	-4.051266	-5.148980
C	-2.773600	-3.044384	-5.234577
N	-3.112970	-1.831304	-5.754137
C	-0.571598	-2.000528	-5.001834
C	-1.854074	0.328856	-4.056734
C	-2.880728	0.037647	-3.153942
C	-2.971521	0.712581	-1.933702
C	-2.030802	1.689579	-1.591109
C	-0.997379	1.990315	-2.472856
C	-0.901391	1.315451	-3.700557
C	0.147160	1.574448	-4.689176
C	1.185479	2.508109	-4.571119
C	2.109899	2.650206	-5.599616
C	1.989676	1.863895	-6.746586
C	0.939395	0.958278	-6.818083
N	0.059293	0.812786	-5.816504
Rh	-1.554686	-0.477380	-5.872328
H	-3.619584	-0.719777	-3.398161
H	-2.104328	2.211802	-0.641978
H	2.678411	1.957044	-7.577927
H	1.408007	-1.184913	-4.755758
H	-6.354516	-2.325594	-6.488232
H	0.945638	-5.263964	-3.476121
C	-0.579617	-2.333636	-8.314063
C	-0.603652	-2.848169	-9.602987
H	-0.044985	-3.746399	-9.839750
C	-1.988295	-0.552246	-8.899558
C	-1.381171	-2.177164	-10.580256
H	-1.426391	-2.555275	-11.597510
C	-2.072153	-1.040165	-10.230233
H	-2.676645	-0.482315	-10.936780
C	-2.687790	0.632063	-8.506099
O	-3.417579	1.368668	-9.200209
N	-2.527977	0.958336	-7.094767
H	-0.016193	-2.819479	-7.522347
N	-1.243223	-1.224942	-7.950444
O	-2.521311	2.306244	-6.853207
C	-1.921462	3.122062	-7.848179
O	-0.881127	2.793747	-8.373878
C	-2.654386	4.379952	-8.033844
C	-2.025195	5.405588	-8.756355
C	-3.950934	4.572164	-7.533489
C	-2.687408	6.610366	-8.966134
C	-4.600151	5.783264	-7.753208

C	-3.976844	6.810311	-8.466546
H	-1.031804	5.237831	-9.155466
H	-4.448646	3.771662	-7.001716
H	-4.496202	7.743553	-8.649160
C	-5.971069	6.016797	-7.175403
F	-5.897362	6.630717	-5.971132
F	-6.719979	6.809577	-7.974280
F	-6.645933	4.862391	-6.991621
C	-1.994043	7.734865	-9.688788
F	-1.373213	8.567094	-8.819777
F	-1.053358	7.281399	-10.543974
F	-2.867002	8.486290	-10.396026

================================

^{1}Rh4

================================

Charge = 0 Multiplicity = 1
Cartesian coordinates:

ATOM	X	Y	Z
Rh	1.031076	-0.828268	0.250821
N	0.254590	-2.393194	-0.902180
C	1.187181	-3.019643	-1.599016
S	0.572618	-4.304553	-2.621316
C	-1.052358	-3.969682	-2.036686
C	-1.021453	-2.906043	-1.106238
C	2.569235	-2.619995	-1.441691
C	2.751218	-1.570687	-0.501644
C	4.061836	-1.132394	-0.276822
H	4.248992	-0.334497	0.435160
C	5.137739	-1.705515	-0.961244
H	6.146634	-1.348437	-0.769739
C	4.936069	-2.735862	-1.889688
H	5.780115	-3.173083	-2.414049
C	3.647749	-3.197530	-2.132690
H	3.480594	-3.998260	-2.849522
N	2.017929	0.522549	1.511783
C	2.220242	0.031100	2.723111
S	2.912487	1.168387	3.856615
C	2.933545	2.385459	2.591165
C	2.410523	1.848170	1.393500
C	1.808744	-1.323976	3.003594
C	1.206750	-1.971075	1.894174
C	0.716118	-3.266642	2.090977
H	0.233589	-3.796484	1.276501
C	0.813383	-3.883454	3.341552
H	0.399919	-4.878331	3.482189
C	1.414936	-3.229900	4.425182
H	1.455343	-3.712589	5.395510
C	1.914914	-1.944586	4.260067
H	2.339488	-1.411299	5.106902
C	1.174574	0.580327	-2.643319
C	0.725509	1.369048	-3.698814
H	1.300278	1.419732	-4.617401
C	-0.655391	1.161544	-1.332222
C	-0.464173	2.084754	-3.541437
H	-0.838835	2.713461	-4.343848
C	-1.163921	1.976844	-2.343189
H	-2.094691	2.496641	-2.146652
C	-1.430877	1.037513	-0.027728
O	-2.449888	1.703745	0.160328
N	-0.841497	0.117400	0.755690
H	2.094973	0.007767	-2.704164
N	0.505404	0.488365	-1.482730
O	-1.477919	-0.084262	2.014886
C	-0.960600	0.687216	3.003942
O	-0.401103	1.748254	2.840950
C	-1.037155	-0.012182	4.324504
C	-0.372956	0.585786	5.396418
C	-1.557683	-1.304589	4.456639
C	-0.200057	-0.120551	6.585399
C	-1.400623	-1.989067	5.658412
C	-0.715618	-1.406896	6.726957
H	0.052570	1.573518	5.264496
H	-2.023625	-1.788130	3.608976
H	-0.552490	-1.963009	7.642786
C	-1.912421	-3.396274	5.809521
F	-0.993033	-4.187105	6.417969
F	-3.028709	-3.444177	6.570443
F	-2.208395	-3.962822	4.621467
C	0.701221	0.454807	7.641171
F	2.005864	0.245985	7.311009
F	0.543345	1.787022	7.774645
F	0.505473	-0.111383	8.850045
C	3.382170	3.704021	2.665789
H	3.777349	4.106592	3.592677
C	3.303045	4.488608	1.518089
H	3.641115	5.519575	1.551780
C	2.791113	3.960823	0.321127
H	2.739444	4.590700	-0.561551
C	2.345274	2.646716	0.245454
H	1.949341	2.237009	-0.671988
C	-2.203223	-2.491391	-0.476581
H	-2.167565	-1.687222	0.245561
C	-3.388589	-3.137617	-0.807961
H	-4.311764	-2.825171	-0.330257
C	-2.244545	-4.614530	-2.368223
H	-2.258552	-5.429165	-3.084847
C	-3.413886	-4.183939	-1.745710
H	-4.354718	-4.669358	-1.986000

================================

3Rh4

================================

Charge = 0 Multiplicity = 3
Cartesian coordinates:

ATOM	X	Y	Z

Rh	1.121389	-0.388758	0.547238
N	0.169050	-2.145711	-0.145718
C	1.043324	-3.034701	-0.589947
S	0.344002	-4.579121	-1.021640
C	-1.242381	-3.964351	-0.584118
C	-1.137894	-2.623838	-0.147451
C	2.440317	-2.672850	-0.669027
C	2.722654	-1.377782	-0.168759
C	4.042128	-0.922626	-0.249494
H	4.300751	0.064964	0.117076
C	5.041421	-1.730751	-0.802255
H	6.060790	-1.357491	-0.857172
C	4.748778	-3.013725	-1.280726
H	5.534109	-3.633164	-1.702365
C	3.443805	-3.488550	-1.216628
H	3.204333	-4.479762	-1.593991
N	2.315943	1.107070	1.366390
C	2.842497	0.761767	2.534320
S	3.845451	1.995804	3.257435
C	3.496707	3.054112	1.899248
C	2.645523	2.398529	0.979668
C	2.529238	-0.531555	3.087516
C	1.600526	-1.268429	2.311287
C	1.182133	-2.509663	2.803306
H	0.439720	-3.092934	2.268493
C	1.678940	-2.996773	4.014771
H	1.319592	-3.947432	4.397888
C	2.613810	-2.265870	4.760738
H	2.971448	-2.650661	5.709282
C	3.036231	-1.025975	4.301500
H	3.743124	-0.439325	4.883066
C	1.193813	0.353167	-2.549608
C	0.683515	0.785431	-3.766407
H	1.247321	0.620256	-4.677478
C	-0.661101	1.169971	-1.386812
C	-0.572955	1.432511	-3.781789
H	-1.003243	1.778046	-4.716624
C	-1.245056	1.618634	-2.590205
H	-2.213151	2.103533	-2.535450
C	-1.369455	1.354172	-0.127063
O	-2.460867	1.910029	-0.000631
N	-0.675720	0.764618	1.002041
H	2.150284	-0.156213	-2.485497
N	0.556581	0.535865	-1.381807
O	-1.342454	1.037999	2.137204
C	-0.542489	0.874373	3.340421
O	0.336662	1.678128	3.569914
C	-0.957413	-0.272851	4.122456
C	-0.288014	-0.520196	5.341083
C	-1.906438	-1.204732	3.660794
C	-0.549477	-1.679920	6.049474
C	-2.143229	-2.370226	4.381069
C	-1.477414	-2.623252	5.581110
H	0.464212	0.180647	5.678820
H	-2.427332	-1.036426	2.729992
H	-1.654318	-3.542595	6.125789
C	-3.024869	-3.422450	3.769033
F	-2.353382	-4.117843	2.814223
F	-3.461768	-4.321510	4.676833
F	-4.112393	-2.888265	3.167007
C	0.207328	-1.999726	7.308063
F	0.813213	-3.213194	7.213853
F	1.169130	-1.094424	7.580638
F	-0.610242	-2.064277	8.383322
C	3.944133	4.356587	1.680762
H	4.595033	4.848731	2.396007
C	3.524414	5.009593	0.523608
H	3.855332	6.025871	0.333950
C	2.675331	4.368972	-0.392619
H	2.354555	4.897742	-1.284825
C	2.229479	3.068997	-0.178385
H	1.574697	2.574579	-0.880172
C	-2.299751	-1.925904	0.208306
H	-2.245009	-0.895024	0.520595
C	-3.522552	-2.583147	0.149024
H	-4.422170	-2.050521	0.437186
C	-2.469759	-4.625626	-0.639847
H	-2.531610	-5.657759	-0.968509
C	-3.609731	-3.922292	-0.261751
H	-4.575816	-4.415908	-0.288245

==============================

[1]Rh5

==============================

Charge = 0 Multiplicity = 1
Cartesian coordinates:

ATOM	X	Y	Z
Rh	1.163937	-0.424401	0.237863
N	0.151148	-2.086610	-0.477526
N	0.993222	-3.063136	-0.924368
C	0.372645	-4.253435	-1.048486
C	-0.959445	-4.065986	-0.661486
C	-1.057485	-2.677999	-0.312466
C	2.353874	-2.691945	-1.110038
C	2.700877	-1.435053	-0.582813
C	4.030072	-1.029045	-0.746314
H	4.342965	-0.066399	-0.351984
C	4.957824	-1.841559	-1.407543
H	5.984813	-1.505190	-1.522709
C	4.574413	-3.083722	-1.921958
H	5.294419	-3.710517	-2.438762
C	3.257130	-3.518880	-1.779582
H	2.948527	-4.474132	-2.193599
N	2.341878	1.003116	1.131622
N	2.708680	0.698091	2.406532
C	3.365828	1.711380	3.002427

C	3.446728	2.752062	2.071652
C	2.777399	2.260462	0.902774
C	2.292434	-0.565046	2.897816
C	1.538177	-1.334412	1.994076
C	1.081271	-2.574361	2.454486
H	0.475274	-3.205247	1.813861
C	1.341457	-3.004324	3.759372
H	0.931320	-3.950784	4.097714
C	2.076771	-2.204525	4.636819
H	2.240825	-2.515755	5.662504
C	2.564027	-0.972053	4.204827
H	3.120729	-0.338987	4.888230
C	1.225842	0.791163	-2.730368
C	0.748329	1.534984	-3.805595
H	1.311138	1.567426	-4.732364
C	-0.600157	1.381722	-1.416651
C	-0.460144	2.222512	-3.661398
H	-0.860396	2.809685	-4.482658
C	-1.148262	2.135519	-2.454957
H	-2.103271	2.617023	-2.279079
C	-1.373944	1.232583	-0.116871
O	-2.569334	1.540521	-0.070334
N	-0.580648	0.719214	0.841277
H	2.155939	0.233046	-2.780444
N	0.570235	0.726519	-1.560705
O	-1.343811	0.307950	1.975153
C	-0.761533	0.591492	3.160908
O	0.015930	1.496279	3.374485
C	-1.143955	-0.438297	4.178479
C	-0.672215	-0.287155	5.484104
C	-1.804743	-1.613697	3.810994
C	-0.865725	-1.307034	6.414158
C	-1.980457	-2.632221	4.744631
C	-1.513844	-2.488506	6.050321
H	-0.134016	0.616211	5.746437
H	-2.138102	-1.751571	2.790945
H	-1.642497	-3.287523	6.770621
C	-2.541629	-3.943969	4.270786
F	-1.592586	-4.661701	3.613317
F	-2.982892	-4.712087	5.289804
F	-3.565723	-3.775628	3.406343
C	-0.249849	-1.187227	7.780270
F	1.037883	-1.622968	7.774683
F	-0.225643	0.089964	8.214596
F	-0.907128	-1.925973	8.700110
H	0.903087	-5.133245	-1.375333
H	3.713595	1.638219	4.019495
C	4.004816	4.052721	2.079429
H	4.510814	4.430776	2.962309
C	3.880963	4.819122	0.942115
H	4.295325	5.822297	0.919060
C	3.211348	4.322875	-0.214248
H	3.132711	4.964536	-1.087032
C	2.658547	3.061040	-0.256258
H	2.146050	2.692257	-1.133820
C	-2.280794	-2.127106	0.139288
H	-2.379289	-1.076920	0.375633
C	-3.348856	-2.989819	0.266262
H	-4.296899	-2.600763	0.624241
C	-2.083169	-4.918653	-0.531332
H	-2.010418	-5.970797	-0.788241
C	-3.254959	-4.374760	-0.057538
H	-4.130211	-5.003918	0.069844

==============================

^{3}Rh5

==============================

Charge = 0 Multiplicity = 3

Cartesian coordinates:

ATOM	X	Y	Z
Rh	1.186416	-0.401639	0.259314
N	0.144951	-2.065930	-0.472192
N	0.985536	-3.056072	-0.890766
C	0.363645	-4.246971	-0.991084
C	-0.969984	-4.047807	-0.617034
C	-1.067066	-2.651759	-0.300888
C	2.348474	-2.698192	-1.076613
C	2.709244	-1.444014	-0.556533
C	4.042136	-1.050620	-0.717014
H	4.365016	-0.091818	-0.322194
C	4.962863	-1.875715	-1.372270
H	5.993848	-1.551090	-1.484270
C	4.567066	-3.115256	-1.882824
H	5.281398	-3.751738	-2.395544
C	3.245150	-3.536039	-1.741409
H	2.927038	-4.489281	-2.152493
N	2.354596	0.976607	1.136889
N	2.834016	0.592410	2.374348
C	3.496690	1.671967	2.982017
C	3.452470	2.751919	2.092921
C	2.723778	2.289540	0.941592
C	2.382945	-0.615717	2.876673
C	1.543861	-1.349126	2.004455
C	1.040601	-2.563377	2.464539
H	0.373132	-3.152097	1.845271
C	1.331421	-3.025926	3.756834
H	0.884464	-3.952430	4.102921
C	2.148767	-2.275142	4.604146
H	2.341984	-2.610474	5.617589
C	2.688887	-1.065529	4.169710
H	3.313074	-0.475822	4.833550
C	1.290934	0.800845	-2.731540
C	0.809757	1.505365	-3.831766
H	1.389855	1.539054	-4.747735
C	-0.580158	1.354417	-1.467543
C	-0.424600	2.152269	-3.726034

H	-0.828378	2.707634	-4.567426
C	-1.133619	2.067616	-2.531128
H	-2.106363	2.522332	-2.383456
C	-1.365323	1.217665	-0.174934
O	-2.560782	1.525199	-0.137475
N	-0.580793	0.718681	0.800006
H	2.240019	0.273582	-2.751987
N	0.613992	0.736766	-1.574902
O	-1.341773	0.342091	1.940174
C	-0.706932	0.588272	3.113269
O	0.115461	1.457972	3.298176
C	-1.102252	-0.426674	4.137113
C	-0.594479	-0.296620	5.431724
C	-1.814749	-1.575239	3.782463
C	-0.809010	-1.309285	6.364671
C	-2.006204	-2.589553	4.717147
C	-1.508969	-2.464919	6.013199
H	-0.013589	0.582886	5.682986
H	-2.174258	-1.698801	2.769639
H	-1.652368	-3.259984	6.735148
C	-2.617855	-3.880351	4.249150
F	-1.707926	-4.619323	3.561787
F	-3.056138	-4.643341	5.273297
F	-3.659085	-3.675165	3.412286
C	-0.169521	-1.216103	7.722324
F	1.079727	-1.748565	7.714003
F	-0.046289	0.062097	8.137207
F	-0.873134	-1.889482	8.659023
H	0.894691	-5.135759	-1.291632
H	3.907932	1.581255	3.973379
C	3.939485	4.064643	2.125229
H	4.483306	4.435019	2.987724
C	3.691214	4.911971	1.003613
H	4.062555	5.931876	1.024190
C	2.989152	4.458118	-0.088747
H	2.803763	5.116408	-0.931243
C	2.481283	3.112421	-0.140696
H	1.920080	2.764708	-0.995479
C	-2.294059	-2.092294	0.130277
H	-2.398107	-1.037874	0.342290
C	-3.362487	-2.952386	0.272300
H	-4.311897	-2.554698	0.616821
C	-2.093338	-4.898272	-0.472159
H	-2.017691	-5.956180	-0.703051
C	-3.267488	-4.344562	-0.016627
H	-4.142818	-4.970865	0.123113

6. Crystallographic data

Crystallographic data of **6o** (CCDC 2355152).

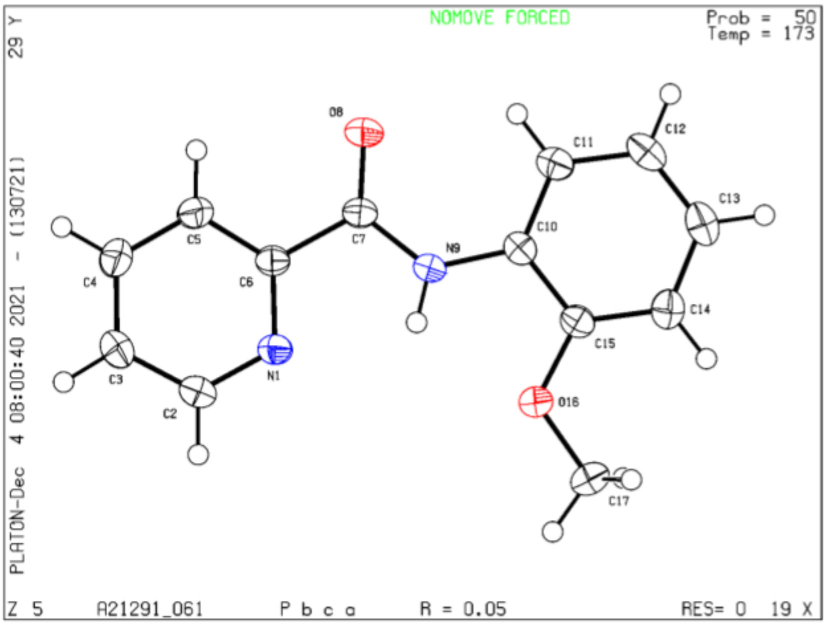

Table S12. Crystal data and structure refinement for **6o**.

Empirical formula	C13 H12 N2 O2	
Formula weight	228.25	
Temperature	173(2) K	
Wavelength	0.71073 Å	
Crystal system	Orthorhombic	
Space group	Pbca	
Unit cell dimensions	a = 7.3553(4) Å	α = 90°
	b = 14.4949(6) Å	β = 90°
	c = 21.0890(9) Å	γ = 90°
Volume	2248.39(18) Å3	
Z	8	
Density (calculated)	1.349 Mg/m3	
Absorption coefficient	0.093 mm-1	
F(000)	960	
Crystal size	0.288 x 0.089 x 0.071 mm3	
Theta range for data collection	3.253 to 28.306°.	
Index ranges	–9<=h<=9, –19<=k<=19, –27<=l<=27	
Reflections collected	29951	
Independent reflections	2763 [R(int) = 0.0448]	
Completeness to theta = 25.242°	98.4 %	
Absorption correction	Semi-empirical from equivalents	
Max. and min. transmission	0.7457 and 0.6947	
Refinement method	Full-matrix least-squares on F2	
Data / restraints / parameters	2763 / 0 / 158	
Goodness-of-fit on F2	1.093	
Final R indices [I>2sigma(I)]	R1 = 0.0450, wR2 = 0.0965	
R indices (all data)	R1 = 0.0544, wR2 = 0.1009	
Largest diff. peak and hole	0.277 and –0.174 e·Å–3	

Crystallographic data of **6p** (CCDC 2355153).

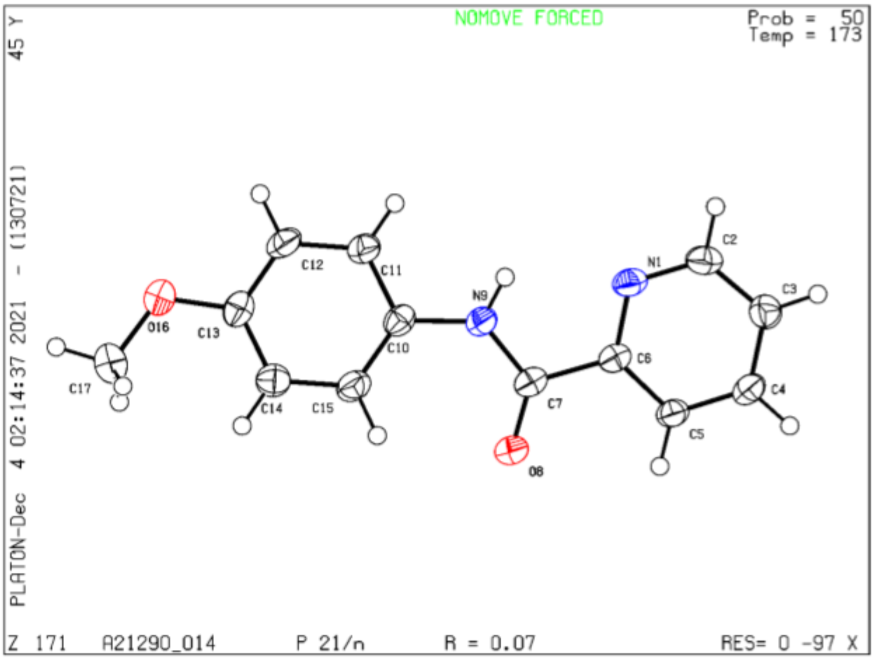

Table S13. Crystal data and structure refinement for **6p**.

Empirical formula	C13 H12 N2 O2	
Formula weight	228.25	
Temperature	173(2) K	
Wavelength	0.71073 Å	
Crystal system	Monoclinic	
Space group	P21/n	
Unit cell dimensions	a = 4.9537(3) Å	α = 90°
	b = 20.6051(15) Å	β = 96.8830(19)°
	c = 11.1246(7) Å	γ = 90°
Volume	1127.32(13) Å3	
Z	4	
Density (calculated)	1.345 Mg/m3	
Absorption coefficient	0.093 mm-1	
F(000)	480	
Crystal size	0.118 x 0.057 x 0.021 mm3	
Theta range for data collection	2.704 to 27.108°.	
Index ranges	–6<=h<=6, –26<=k<=26, –14<=l<=14	
Reflections collected	16804	
Independent reflections	2463 [R(int) = 0.0857]	
Completeness to theta = 25.242°	99.0 %	
Absorption correction	Semi-empirical from equivalents	
Max. and min. transmission	0.7455 and 0.6151	
Refinement method	Full-matrix least-squares on F2	
Data / restraints / parameters	2463 / 0 / 158	
Goodness-of-fit on F2	1.157	
Final R indices [I>2sigma(I)]	R1 = 0.0724, wR2 = 0.1251	
R indices (all data)	R1 = 0.0948, wR2 = 0.1319	
Largest diff. peak and hole	0.217 and –0.231 e·Å–3	

Crystallographic data of **11a** (CCDC 2355154).

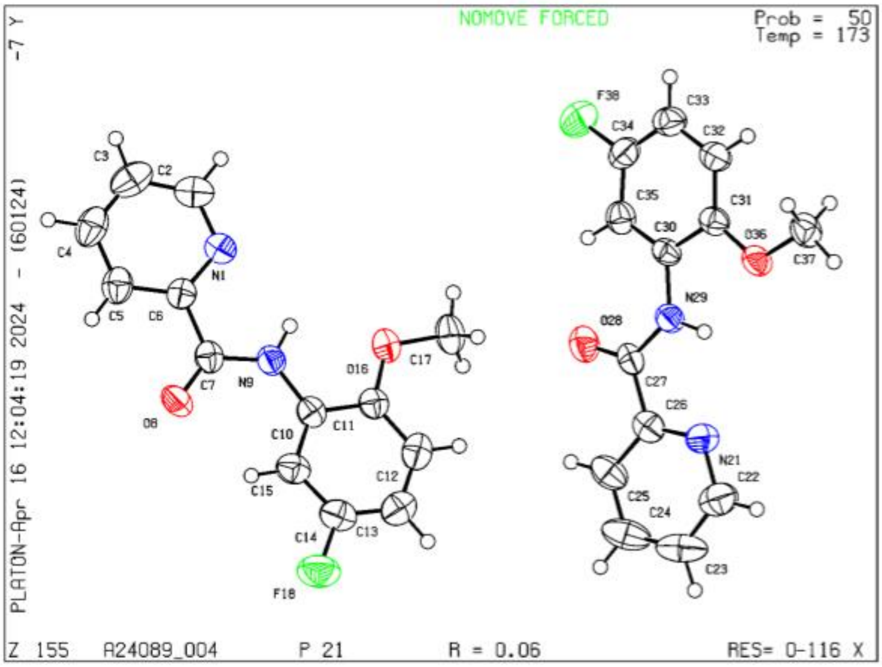

Table S14. Crystal data and structure refinement for **11a**.

Empirical formula	C13 H11 F N2 O2	
Formula weight	246.24	
Temperature	173(2) K	
Wavelength	0.71073 Å	
Crystal system	Monoclinic	
Space group	P21	
Unit cell dimensions	a = 15.656(2) Å	α = 90°
	b = 3.9084(5) Å	β = 93.320(5)°
	c = 18.999(3) Å	γ = 90°
Volume	1160.6(3) Å3	
Z	4	
Density (calculated)	1.409 Mg/m3	
Absorption coefficient	0.108 mm-1	
F(000)	512	
Crystal size	0.165 x 0.062 x 0.024 mm3	
Theta range for data collection	2.447 to 27.579°.	
Index ranges	–20<=h<=20, –5<=k<=5, –17<=l<=24	
Reflections collected	16885	
Independent reflections	5128 [R(int) = 0.0878]	
Completeness to theta = 25.242°	99.8 %	
Absorption correction	Semi-empirical from equivalents	
Max. and min. transmission	0.7455 and 0.5963	
Refinement method	Full-matrix least-squares on F2	
Data / restraints / parameters	5128 / 1 / 333	
Goodness-of-fit on F2	1.134	
Final R indices [I>2sigma(I)]	R1 = 0.0592, wR2 = 0.1005	
R indices (all data)	R1 = 0.1289, wR2 = 0.1274	
Absolute structure parameter	-0.6(8)	
Largest diff. peak and hole	0.168 and –0.228 e·Å–3	

Crystallographic data of **12** (CCDC 2355156).

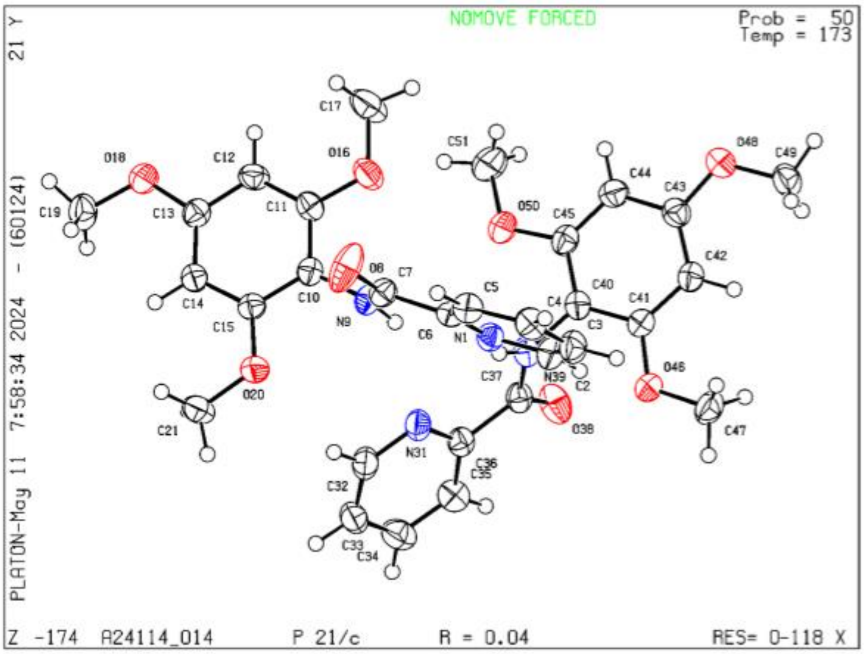

Table S15. Crystal data and structure refinement for **12**.

Empirical formula	C_{15} H_{16} N_2 O_4	
Formula weight	288.30	
Temperature	173(2) K	
Wavelength	0.71073 Å	
Crystal system	Monoclinic	
Space group	$P2_1/c$	
Unit cell dimensions	a = 11.3208(6) Å	α = 90°
	b = 30.5217(15) Å	β = 92.4635(18)°
	c = 8.1329(4) Å	γ = 90°
Volume	2807.6(2) Å^3	
Z	8	
Density (calculated)	1.364 Mg/m^3	
Absorption coefficient	0.100 mm^{-1}	
F(000)	1216	
Crystal size	0.163 x 0.082 x 0.029 mm^3	
Theta range for data collection	2.594 to 27.020°.	
Index ranges	–14<=h<=14, –39<=k<=38, –10<=l<=10	
Reflections collected	42961	
Independent reflections	6152 [R(int) = 0.0855]	
Completeness to theta = 25.242°	99.9 %	
Absorption correction	Semi-empirical from equivalents	
Max. and min. transmission	0.7455 and 0.6593	
Refinement method	Full-matrix least-squares on F^2	
Data / restraints / parameters	6152 / 0 / 391	
Goodness-of-fit on F^2	1.070	
Final R indices [I>2sigma(I)]	R1 = 0.0435, wR2 = 0.0937	
R indices (all data)	R1 = 0.0703, wR2 = 0.1055	
Largest diff. peak and hole	0.196 and –0.189 e·Å^{-3}	

Crystallographic data of **Rh1a** (CCDC 2355155).

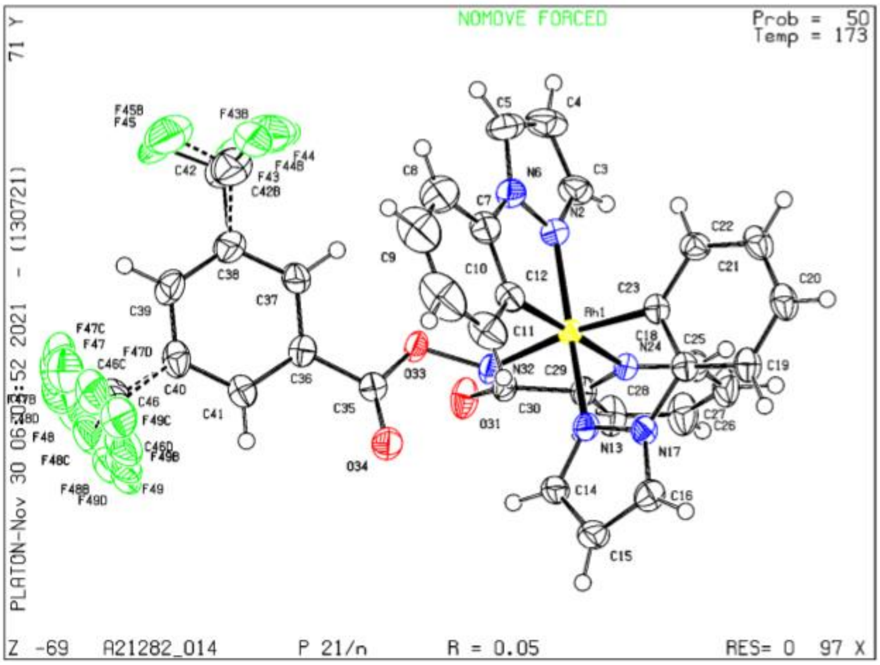

Table S16. Crystal data and structure refinement for **Rh1a**.

Empirical formula	$C_{33} H_{21} F_6 N_6 O_3$ Rh	
Formula weight	766.47	
Temperature	173(2) K	
Wavelength	0.71073 Å	
Crystal system	Monoclinic	
Space group	$P2_1/n$	
Unit cell dimensions	a = 13.5490(9) Å	α = 90°
	b = 17.0360(10) Å	β = 91.537(2)°
	c = 13.5794(8) Å	γ = 90°
Volume	3133.3(3) $Å^3$	
Z	4	
Density (calculated)	1.625 Mg/m^3	
Absorption coefficient	0.626 mm^{-1}	
F(000)	1536	
Crystal size	0.113 x 0.042 x 0.037 mm^3	
Theta range for data collection	2.413 to 27.558°.	
Index ranges	–17<=h<=17, –22<=k<=22, –17<=l<=17	
Reflections collected	61886	
Independent reflections	7215 [R(int) = 0.1215]	
Completeness to theta = 25.242°	99.8 %	
Absorption correction	Semi-empirical from equivalents	
Max. and min. transmission	0.7456 and 0.3072	
Refinement method	Full-matrix least-squares on F^2	
Data / restraints / parameters	7215 / 871 / 591	
Goodness-of-fit on F^2	1.030	
Final R indices [I>2sigma(I)]	R1 = 0.0522, wR2 = 0.1248	
R indices (all data)	R1 = 0.0724, wR2 = 0.1376	
Largest diff. peak and hole	1.006 and –0.842 e·$Å^{-3}$	

Crystallographic data of **Rh1e** (CCDC 2355158).

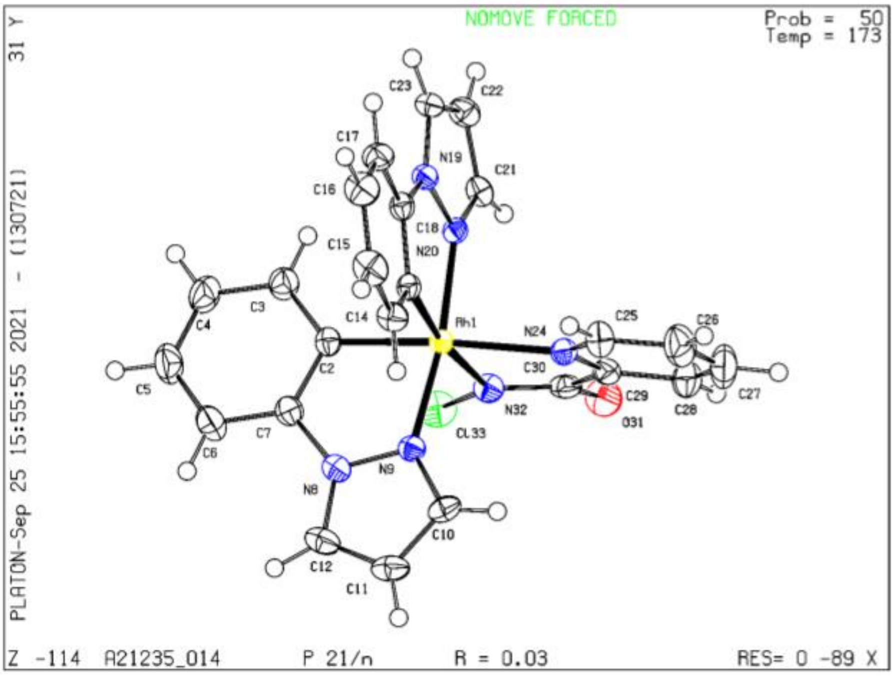

Table S17. Crystal data and structure refinement for **Rh1e**.

Empirical formula	$C_{24}H_{18}ClN_6ORh$	
Formula weight	544.80	
Temperature	173(2) K	
Wavelength	0.71073 Å	
Crystal system	Monoclinic	
Space group	$P2_1/n$	
Unit cell dimensions	a = 9.5312(4) Å	α = 90°
	b = 15.7119(7) Å	β = 98.0040(13)°
	c = 15.2165(6) Å	γ = 90°
Volume	2256.52(16) $Å^3$	
Z	4	
Density (calculated)	1.604 Mg/m^3	
Absorption coefficient	0.905 mm^{-1}	
F(000)	1096	
Crystal size	0.071 x 0.064 x 0.042 mm^3	
Theta range for data collection	2.517 to 27.481°.	
Index ranges	–12<=h<=12, –20<=k<=20, –19<=l<=19	
Reflections collected	38275	
Independent reflections	5158 [R(int) = 0.0619]	
Completeness to theta = 25.242°	99.6 %	
Absorption correction	Semi-empirical from equivalents	
Max. and min. transmission	0.7456 and 0.6989	
Refinement method	Full-matrix least-squares on F^2	
Data / restraints / parameters	5158 / 0 / 298	
Goodness-of-fit on F^2	1.090	
Final R indices [I>2sigma(I)]	R1 = 0.0332, wR2 = 0.0558	
R indices (all data)	R1 = 0.0450, wR2 = 0.0593	
Largest diff. peak and hole	0.383 and –0.606 e·$Å^{-3}$	

Crystallographic data of **Rh2** (CCDC 2355157).

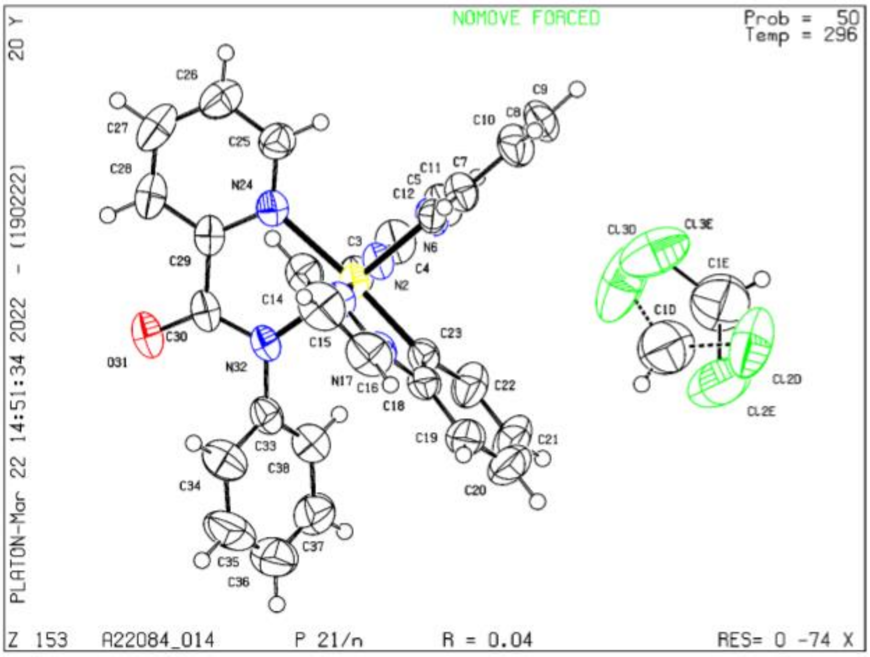

Table S18. Crystal data and structure refinement for **Rh2**.

Empirical formula	C_{31} H_{25} Cl_2 N_6 O Rh	
Formula weight	671.38	
Temperature	296(2) K	
Wavelength	0.71073 Å	
Crystal system	Monoclinic	
Space group	P 21/n	
Unit cell dimensions	a = 9.9322(7) Å	α = 90°
	b = 17.2195(11) Å	β = 93.3134(16)°
	c = 17.0004(11) Å	γ = 90°
Volume	2902.7(3) $Å^3$	
Z	4	
Density (calculated)	1.536 Mg/m^3	
Absorption coefficient	0.809 mm^{-1}	
F(000)	1360	
Crystal size	0.187 x 0.042 x 0.038 mm^3	
Theta range for data collection	2.603 to 27.593°.	
Index ranges	-12<=h<=12, -22<=k<=22, -22<=l<=22	
Reflections collected	43280	
Independent reflections	6689 [R(int) = 0.0838]	
Completeness to theta = 25.242°	99.8 %	
Absorption correction	Semi-empirical from equivalents	
Max. and min. transmission	0.7456 and 0.6420	
Refinement method	Full-matrix least-squares on F^2	
Data / restraints / parameters	6689 / 102 / 398	
Goodness-of-fit on F^2	1.032	
Final R indices [I>2sigma(I)]	R1 = 0.0441, wR2 = 0.0704	
R indices (all data)	R1 = 0.0869, wR2 = 0.0861	
Largest diff. peak and hole	0.424 and –0.456 e·$Å^{-3}$	

Crystallographic data of **Rh3** (CCDC 2355160).

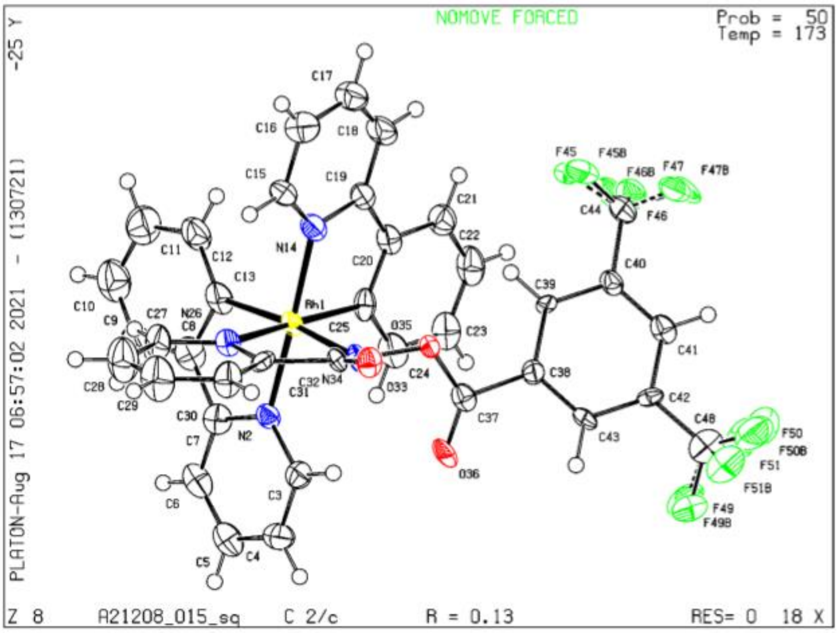

Table S19. Crystal data and structure refinement for **Rh3**.

Empirical formula	$C_{37} H_{23} F_6 N_4 O_3$ Rh	
Formula weight	788.50	
Temperature	173(2) K	
Wavelength	0.71073 Å	
Crystal system	Monoclinic	
Space group	*C*2/*c*	
Unit cell dimensions	a = 26.383(2) Å	a = 90°
	b = 9.0674(7) Å	b = 110.167(3)°
	c = 34.851(4) Å	g = 90°
Volume	7826.0(12) Å^3	
Z	8	
Density (calculated)	1.338 Mg/m^3	
Absorption coefficient	0.502 mm^{-1}	
F(000)	3168	
Crystal size	0.274 x 0.033 x 0.029 mm^3	
Theta range for data collection	2.466 to 25.498°.	
Index ranges	–31<=h<=30, –10<=k<=10, –42<=l<=42	
Reflections collected	36529	
Independent reflections	7185 [R(int) = 0.1760]	
Completeness to theta = 25.242°	98.9 %	
Absorption correction	Semi-empirical from equivalents	
Max. and min. transmission	0.7455 and 0.3978	
Refinement method	Full-matrix least-squares on F^2	
Data / restraints / parameters	7185 / 486 / 444	
Goodness-of-fit on F^2	1.130	
Final R indices [I>2sigma(I)]	R1 = 0.1266, wR2 = 0.2461	
R indices (all data)	R1 = 0.1606, wR2 = 0.2614	
Largest diff. peak and hole	1.295 and –2.719 e·Å^{-3}	

Crystallographic data of **Rh6** (CCDC 2355159).

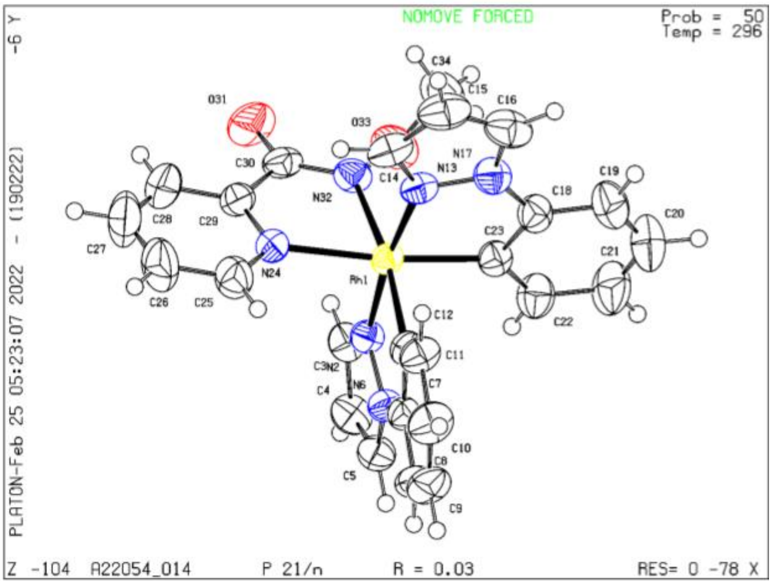

Table S20. Crystal data and structure refinement for **Rh6**.

Empirical formula	$C_{25} H_{21} N_6 O_2$ Rh	
Formula weight	540.39	
Temperature	296(2) K	
Wavelength	0.71073 Å	
Crystal system	Monoclinic	
Space group	$P2_1/n$	
Unit cell dimensions	a = 9.5243(4) Å	α = 90°
	b = 15.7361(7) Å	β = 97.3074(11)°
	c = 15.5814(6) Å	γ = 90°
Volume	2316.30(17) $Å^3$	
Z	4	
Density (calculated)	1.550 Mg/m^3	
Absorption coefficient	0.772 mm^{-1}	
F(000)	1096	
Crystal size	0.164 x 0.115 x 0.051 mm^3	
Theta range for data collection	2.937 to 27.501°.	
Index ranges	–12<=h<=12, –20<=k<=20, –20<=l<=20	
Reflections collected	43111	
Independent reflections	5312 [R(int) = 0.0472]	
Completeness to theta = 25.242°	99.7 %	
Absorption correction	Semi-empirical from equivalents	
Max. and min. transmission	0.7456 and 0.6610	
Refinement method	Full-matrix least-squares on F^2	
Data / restraints / parameters	5312 / 0 / 308	
Goodness-of-fit on F^2	1.141	
Final R indices [I>2sigma(I)]	R1 = 0.0321, wR2 = 0.0582	
R indices (all data)	R1 = 0.0539, wR2 = 0.0718	
Largest diff. peak and hole	0.941 and –0.423 e·$Å^{-3}$	

7. ^{1}H, ^{13}C, ^{19}F, and ^{31}P NMR spectra

N-[{3,5-Bis(trifluoromethyl)benzoyloxy}picolinamide] (1a)

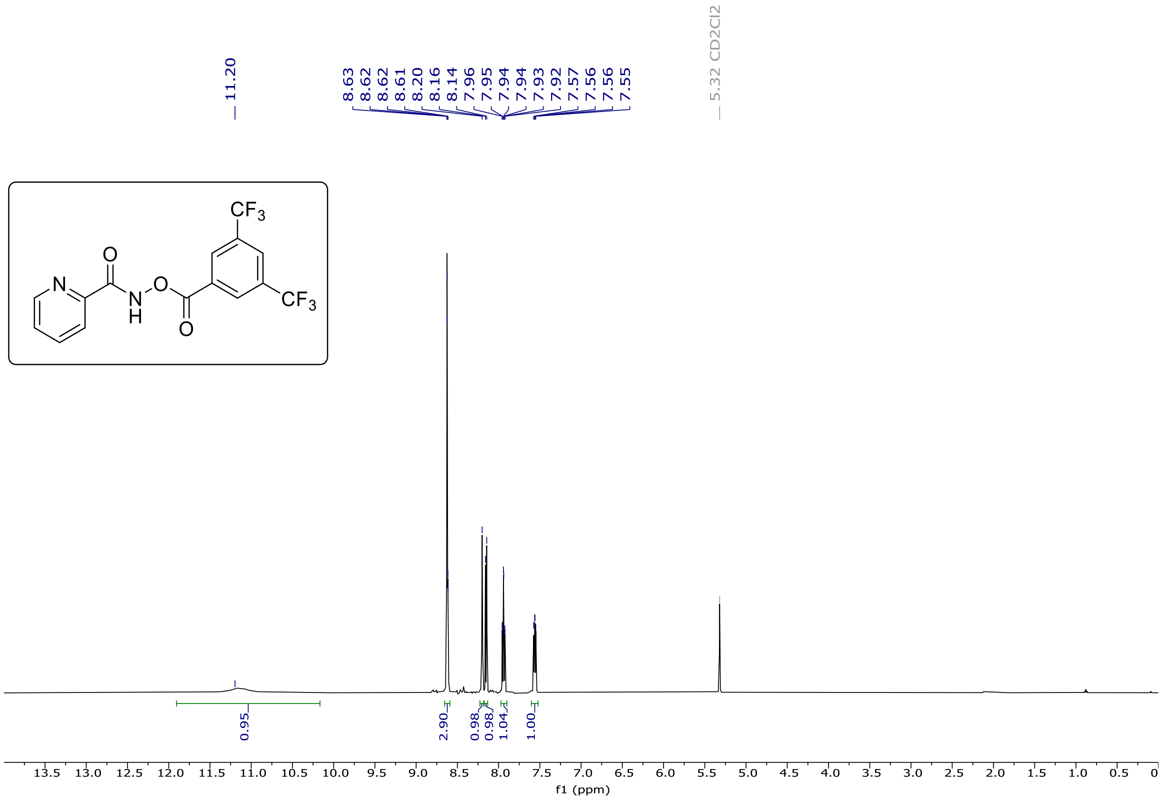

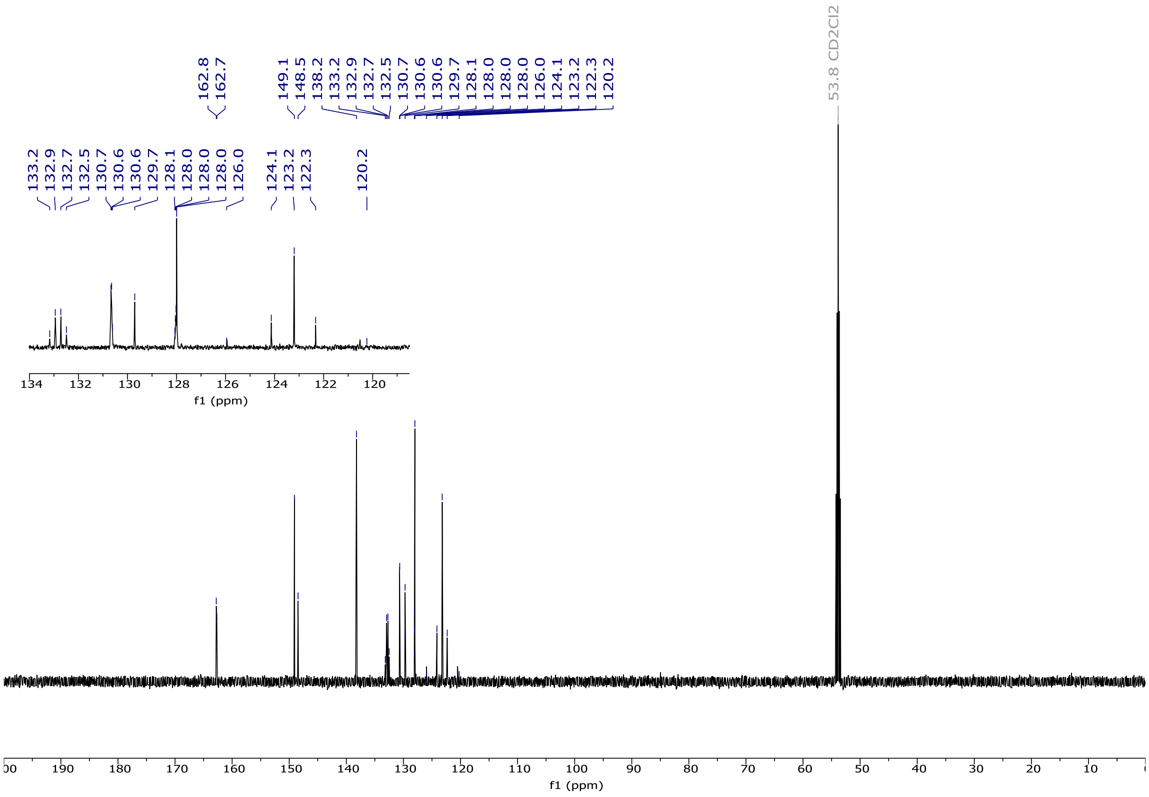

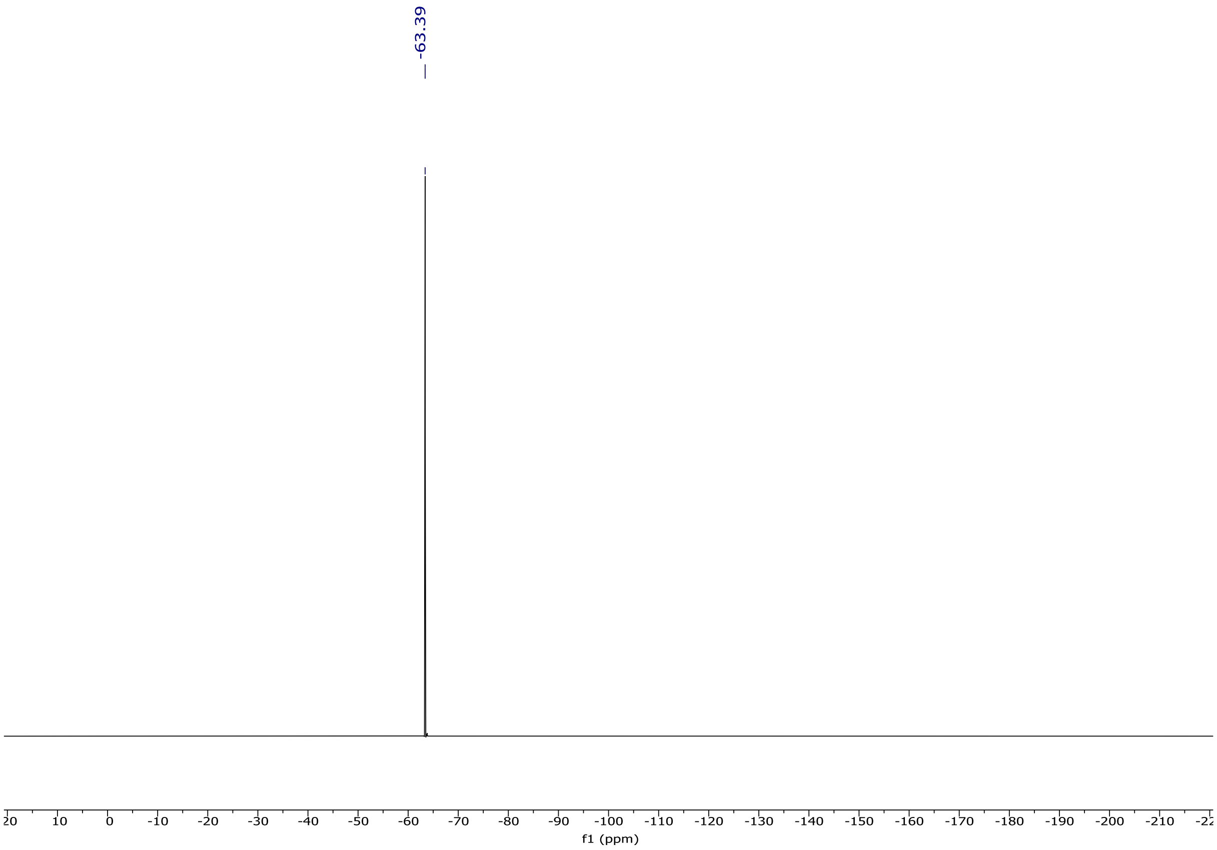

-63.39
20
10
0
-10
-20
-30
-40
-50
-60
-70
-80
-90
-100
-110
-120
-130
-140
-150
-160
-170
-180
-190
-200
-210
f1 (ppm)

^{15}*N*-[{3,5-Bis(trifluoromethyl)benzoyloxy}picolinamide] (1a-^{15}N)

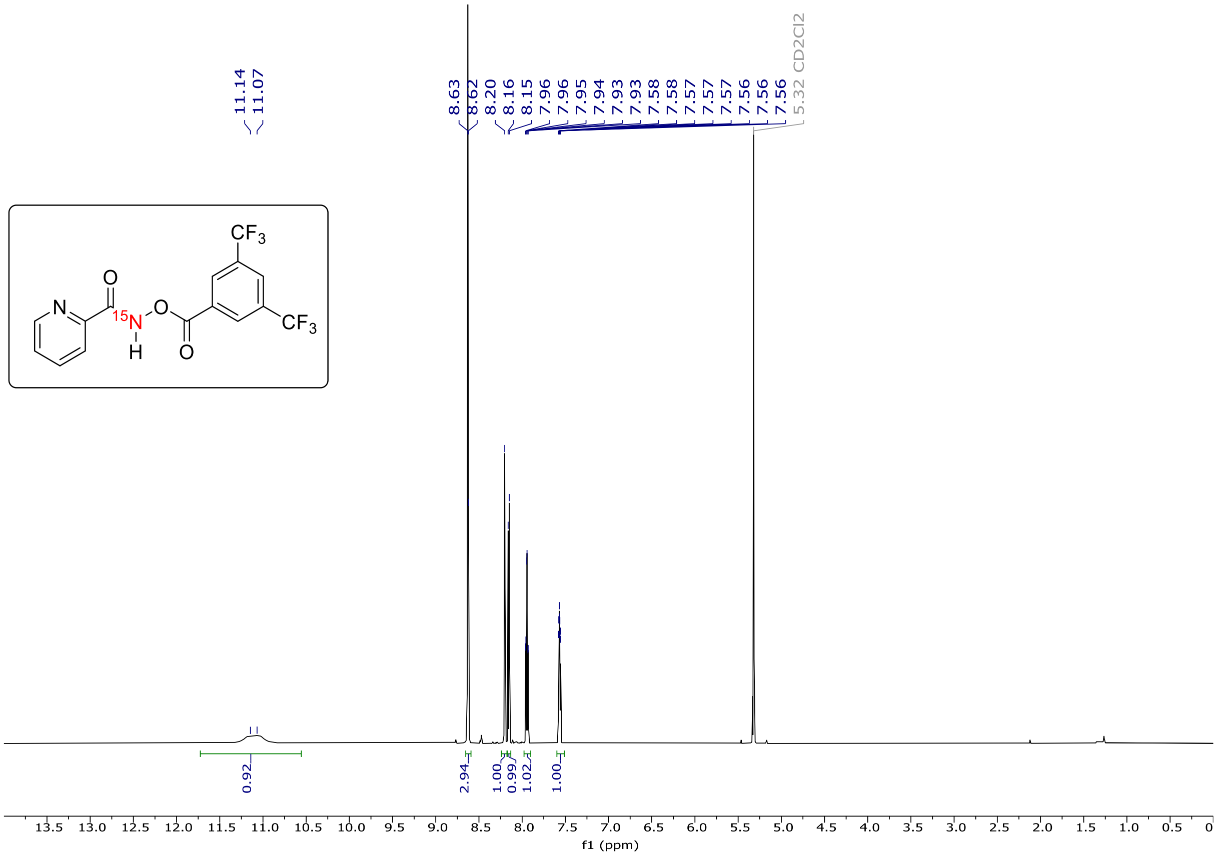

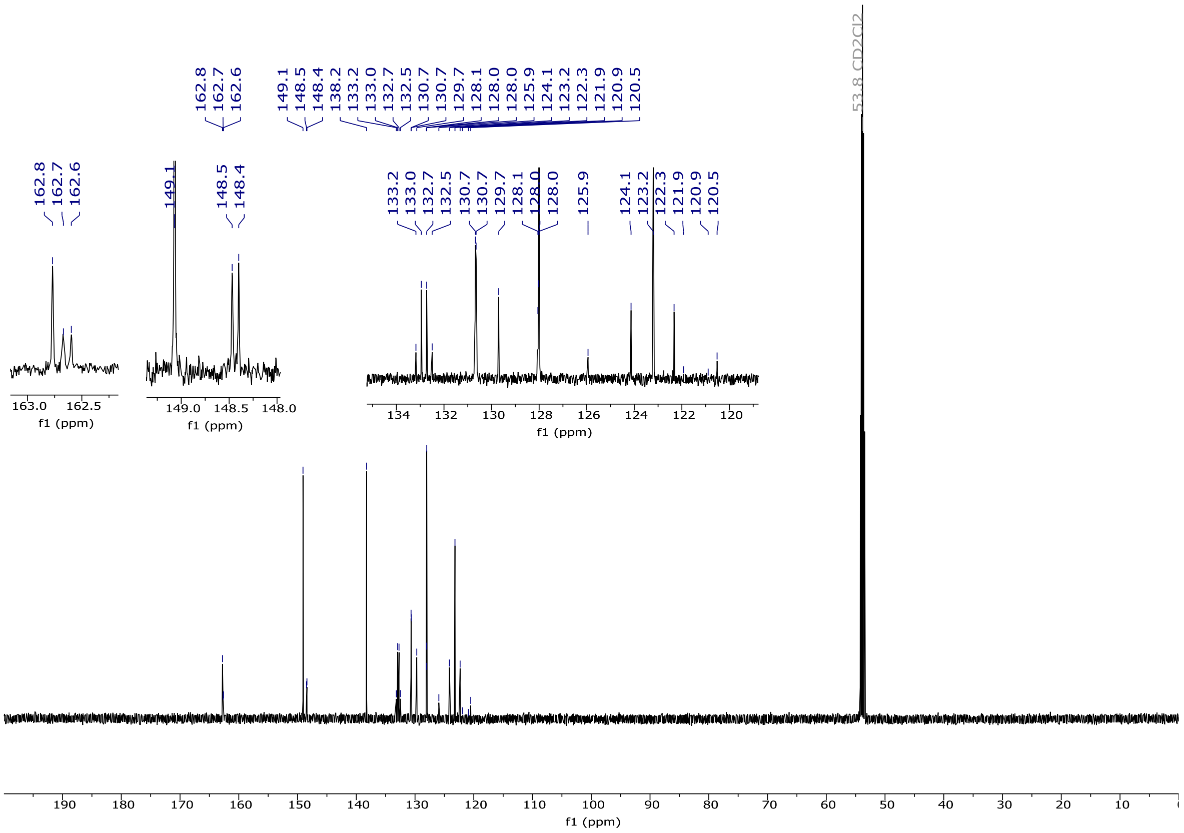

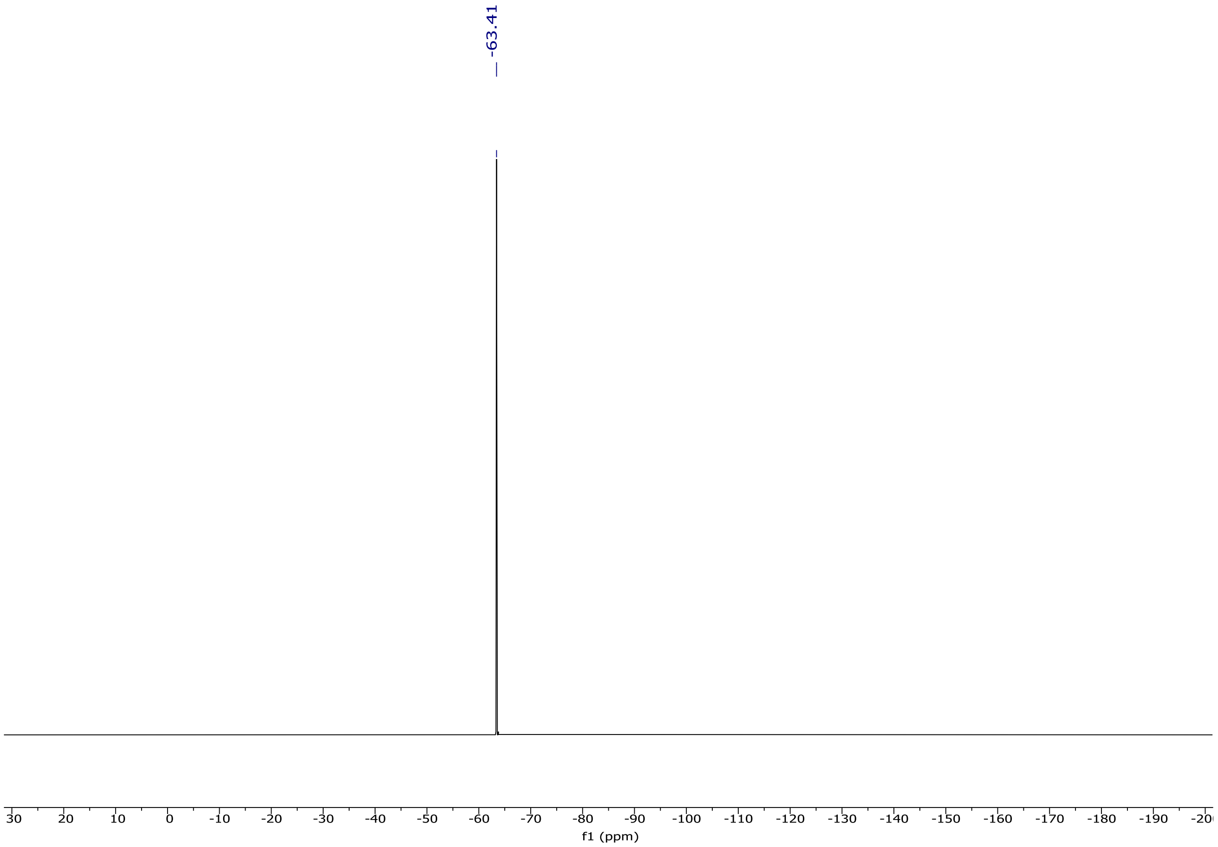

-63.41
30 20 10 0 -10 -20 -30 -40 -50 -60 -70 -80 -90 -100 -110 -120 -130 -140 -150 -160 -170 -180 -190
f1 (ppm)

_N_-(Benzoyloxy)picolinamide (1b)

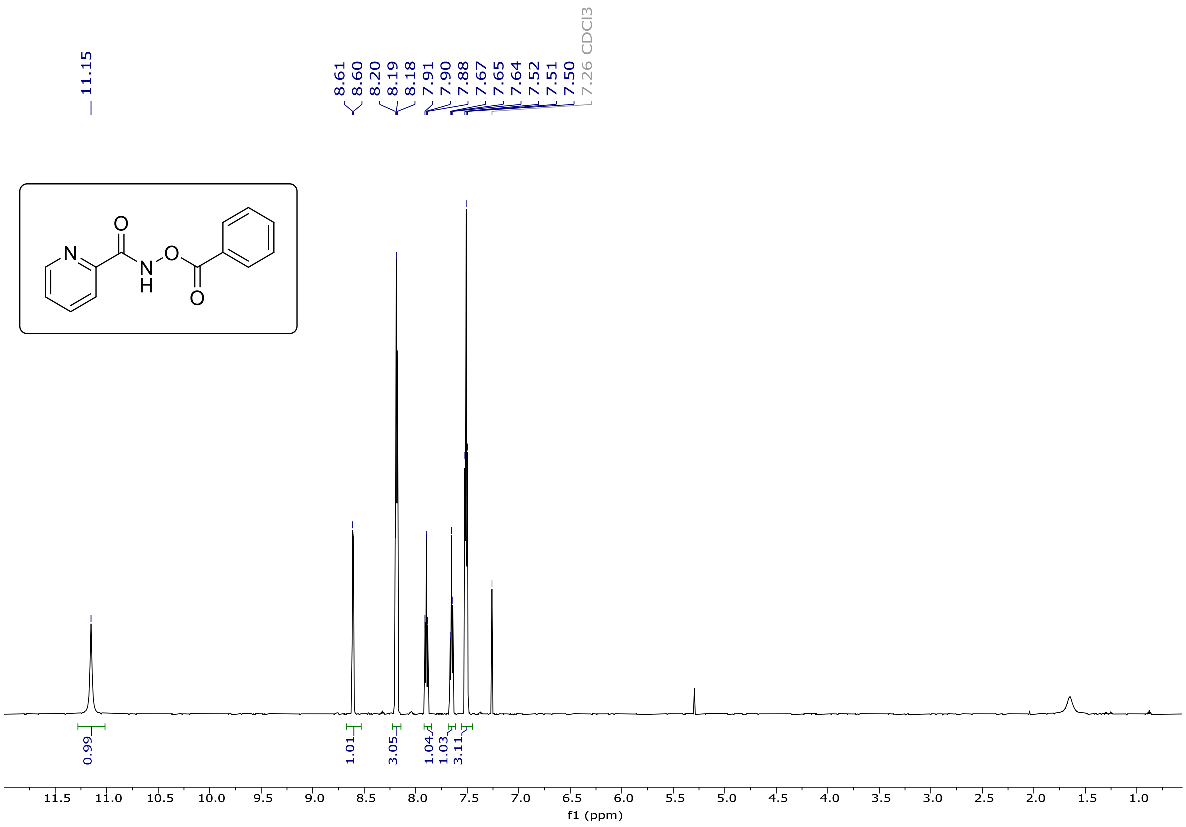

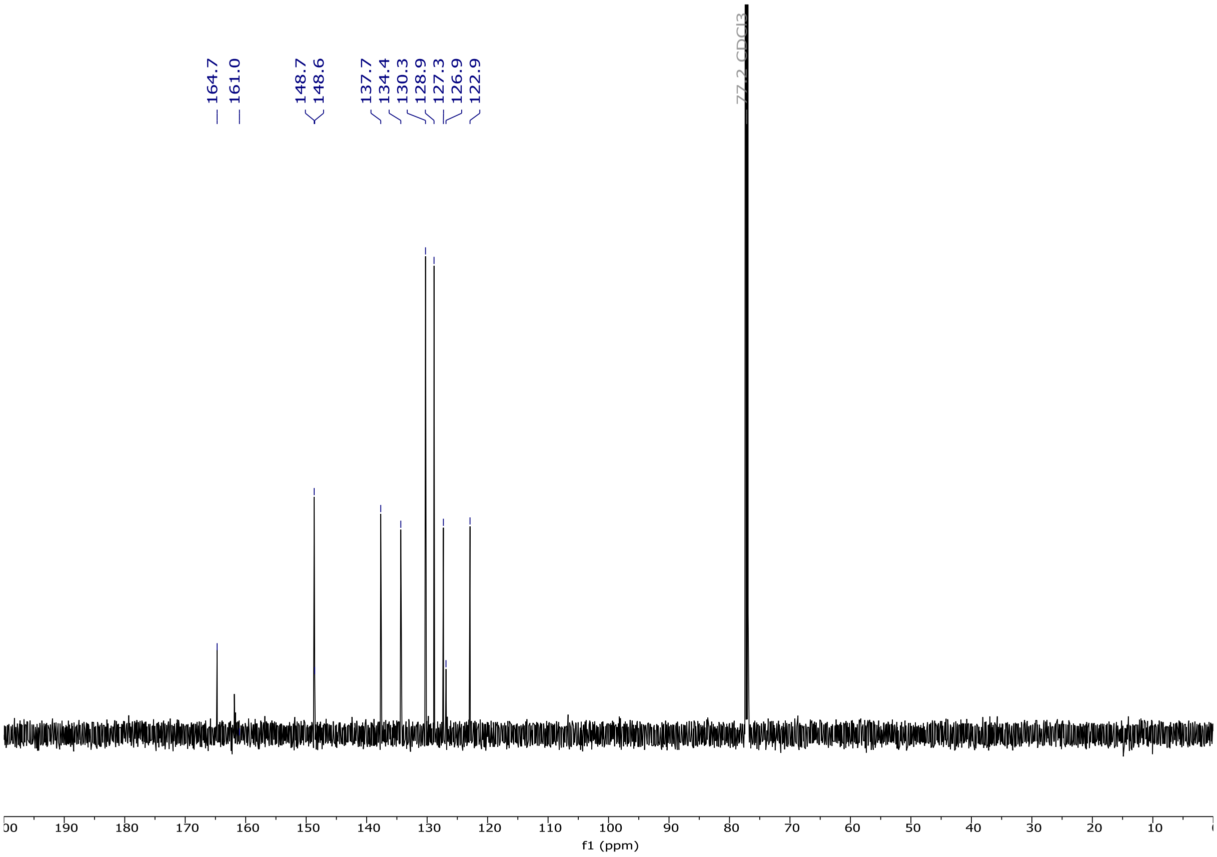

_N_-Acetoxypicolinamide (1c)

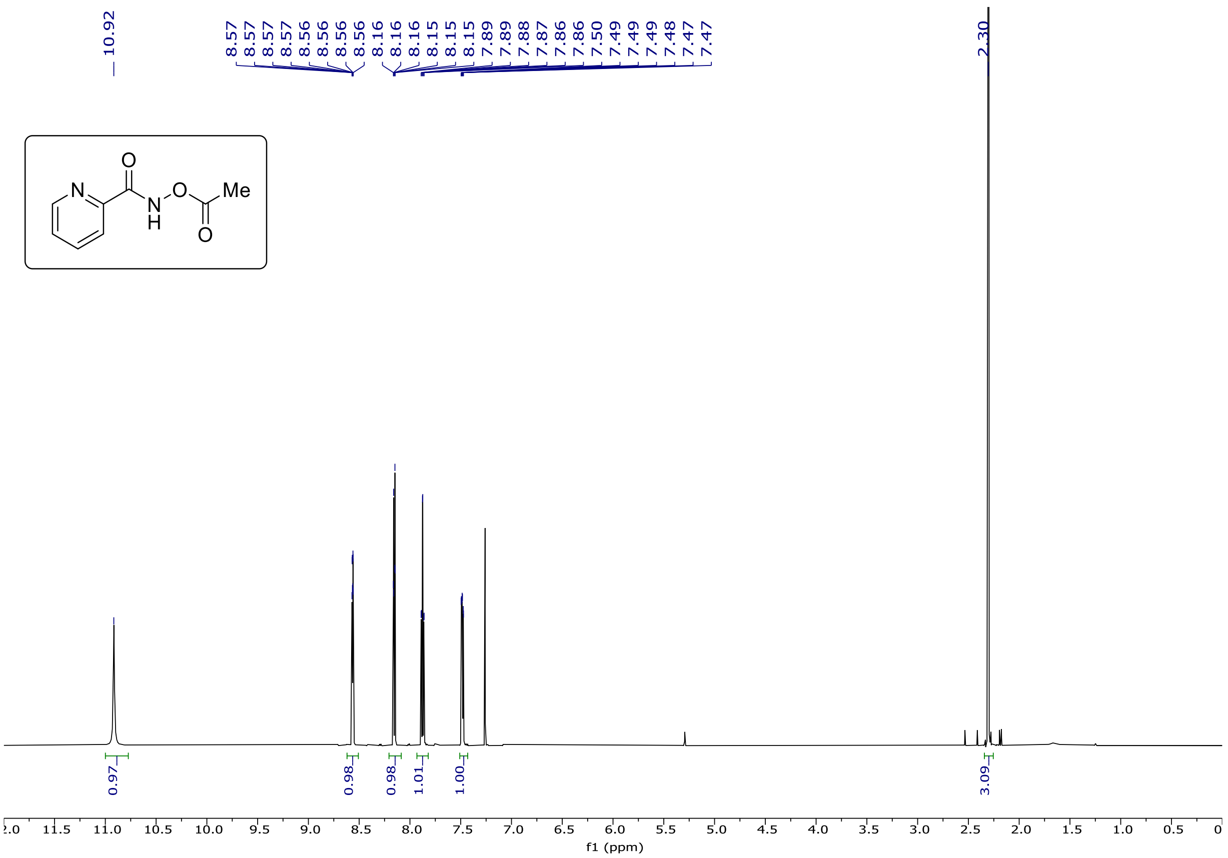

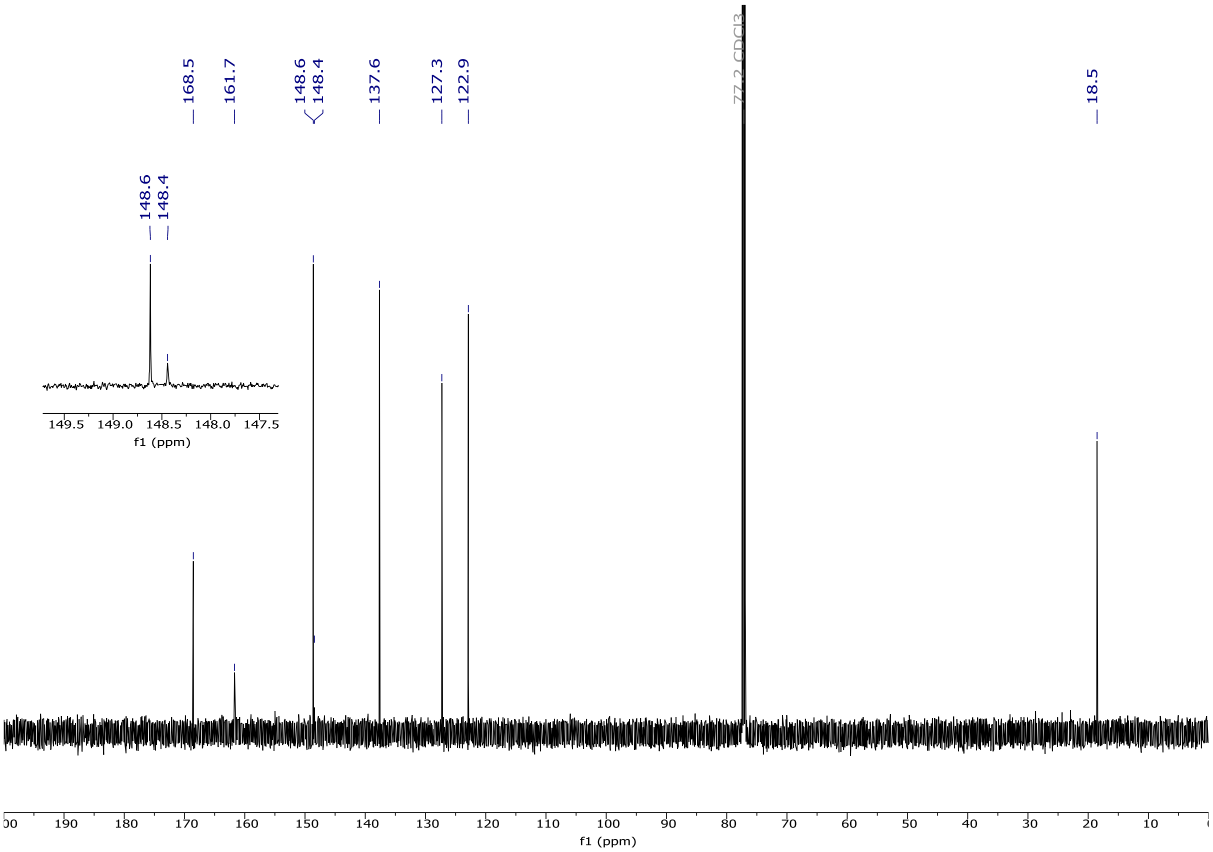

***N*-(Pivaloyloxy)picolinamide (1d)**

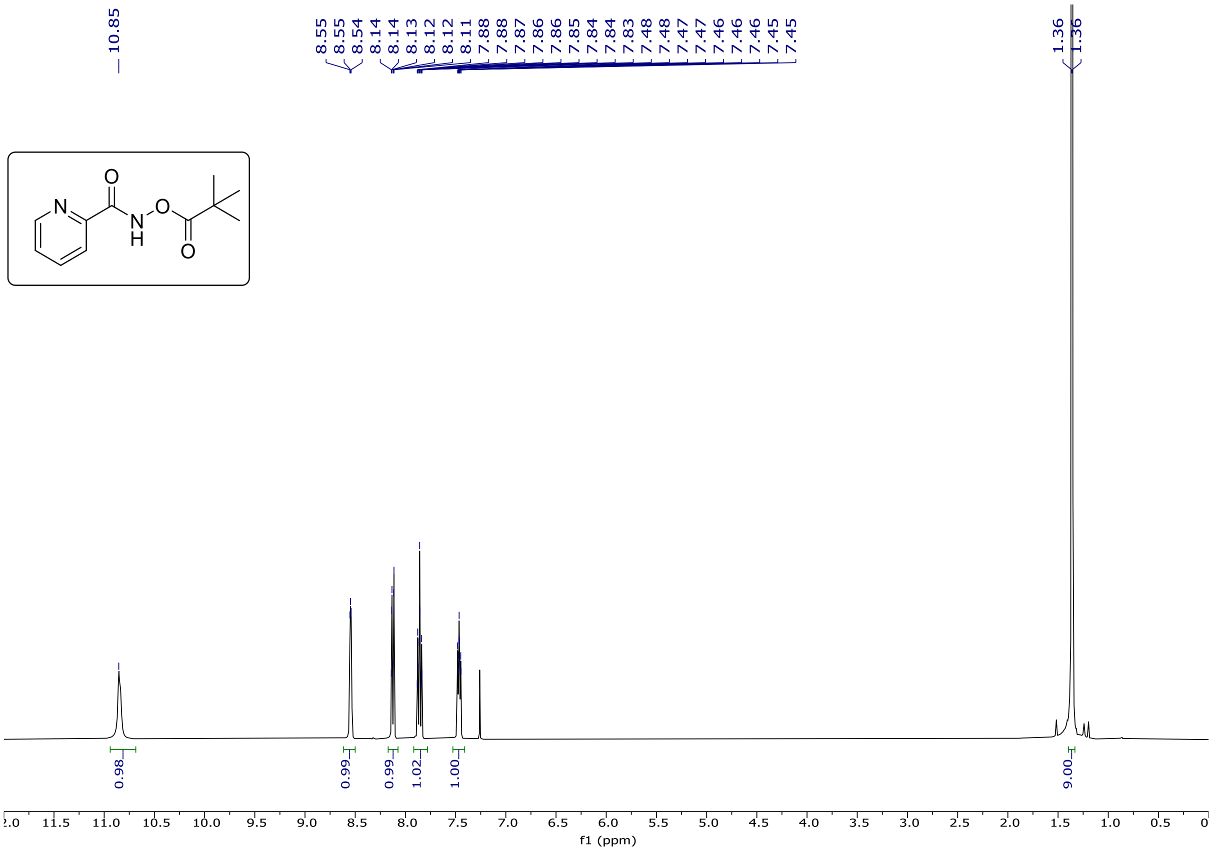

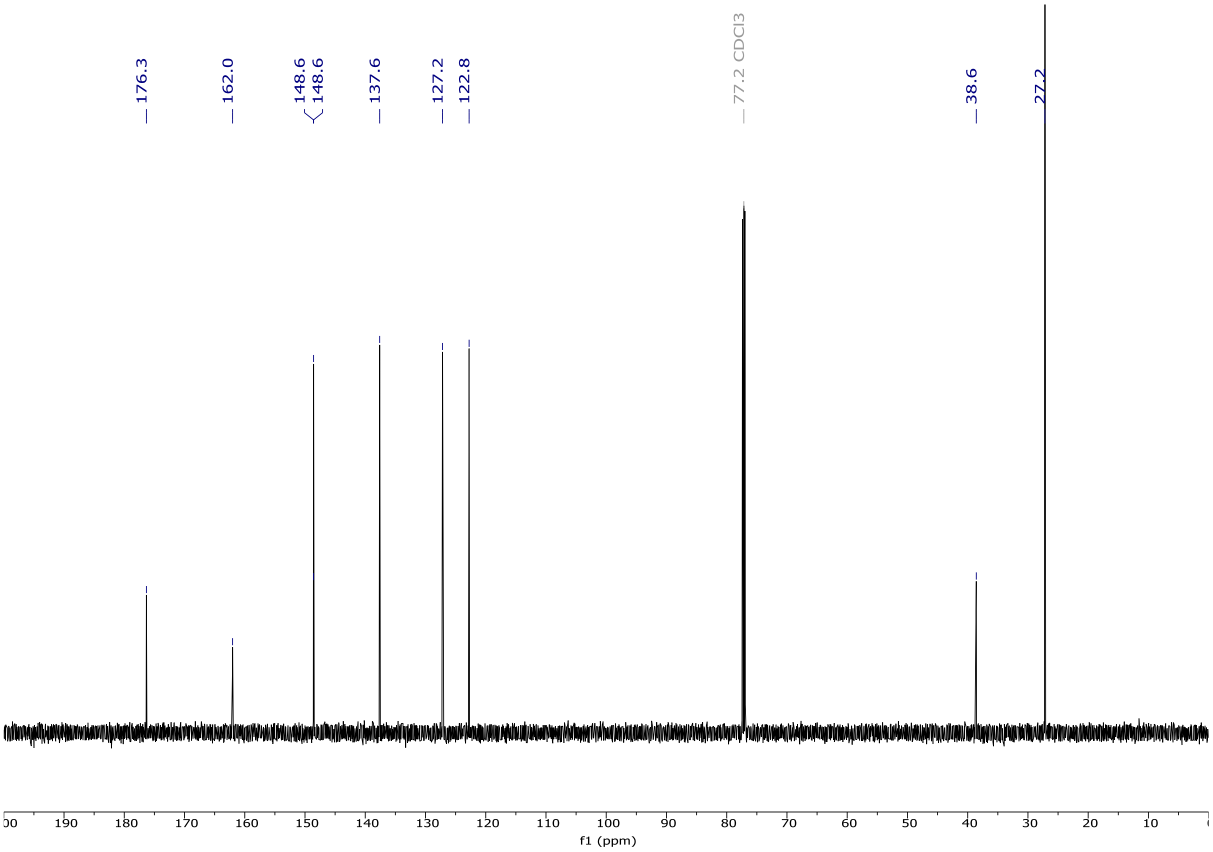

N-Chloropicolinamide (1e)

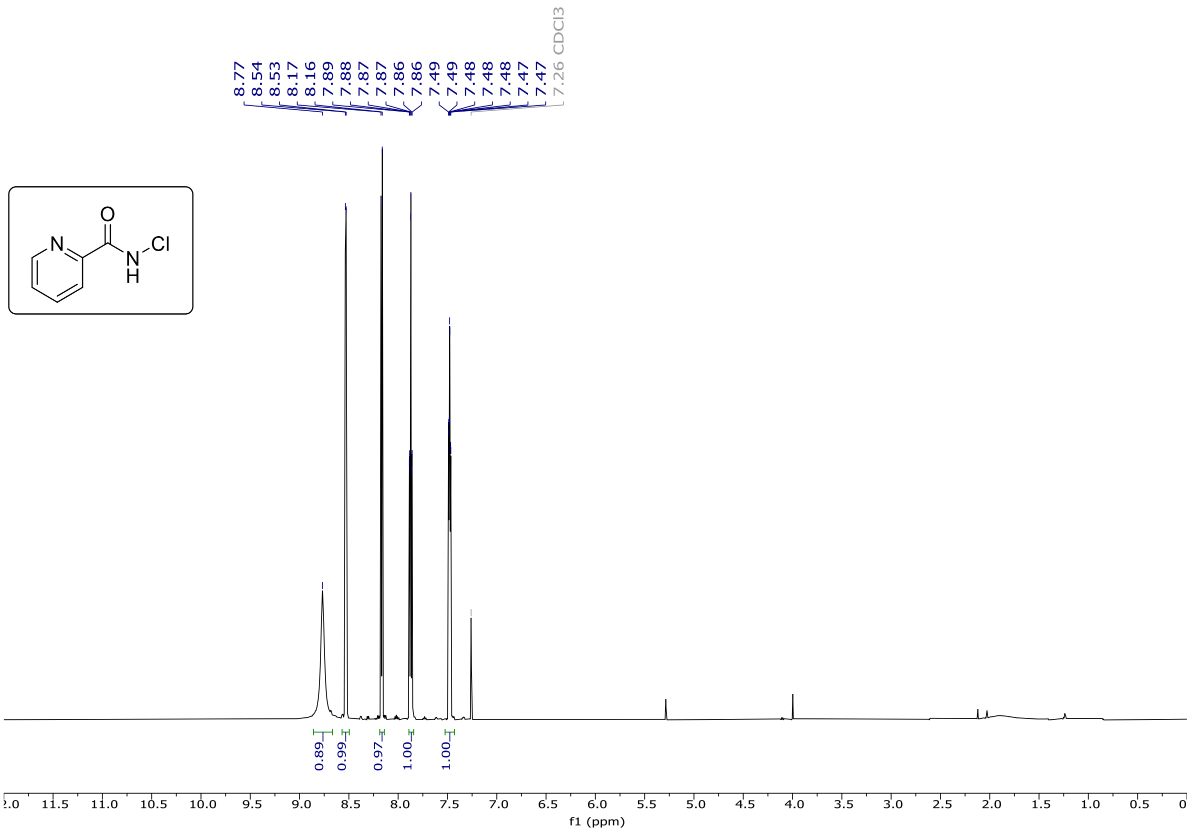

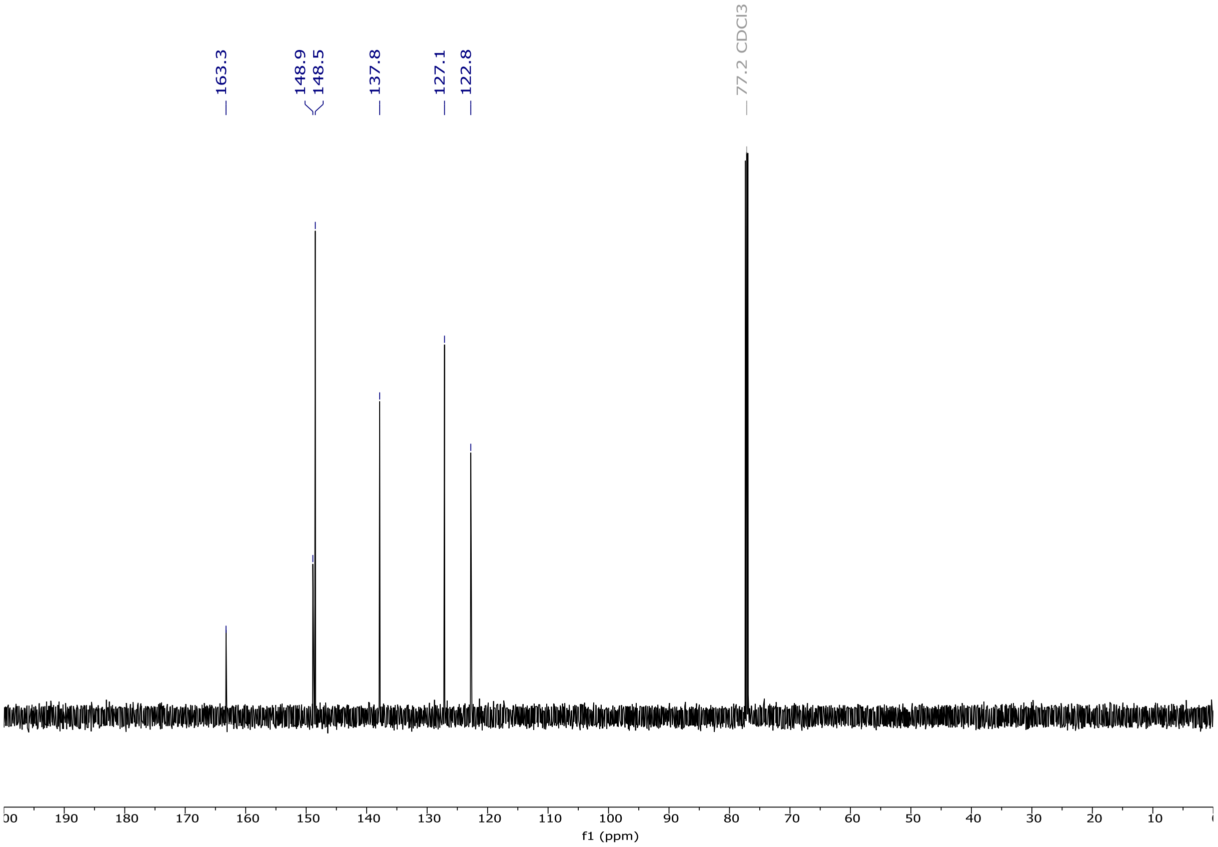

[(PzPh)$_2$Rh(1a)], Rh1a

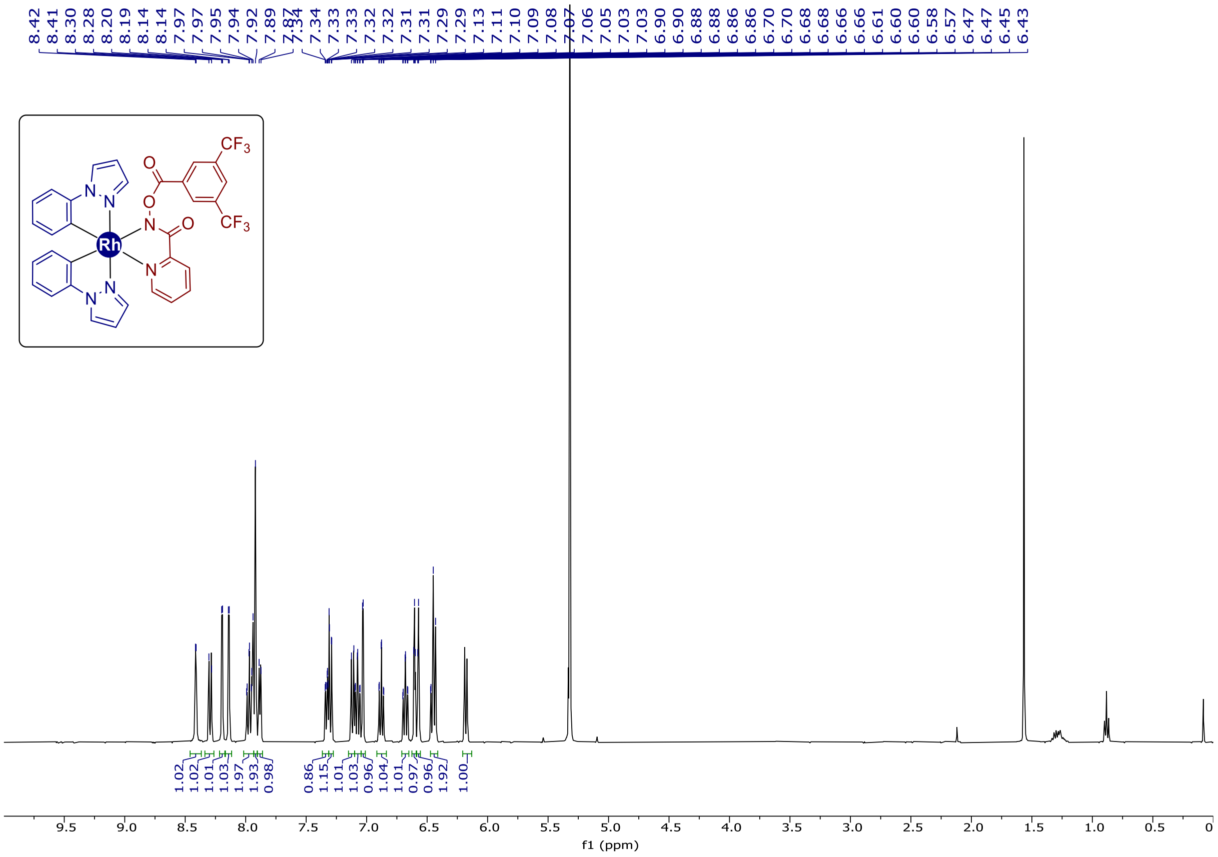

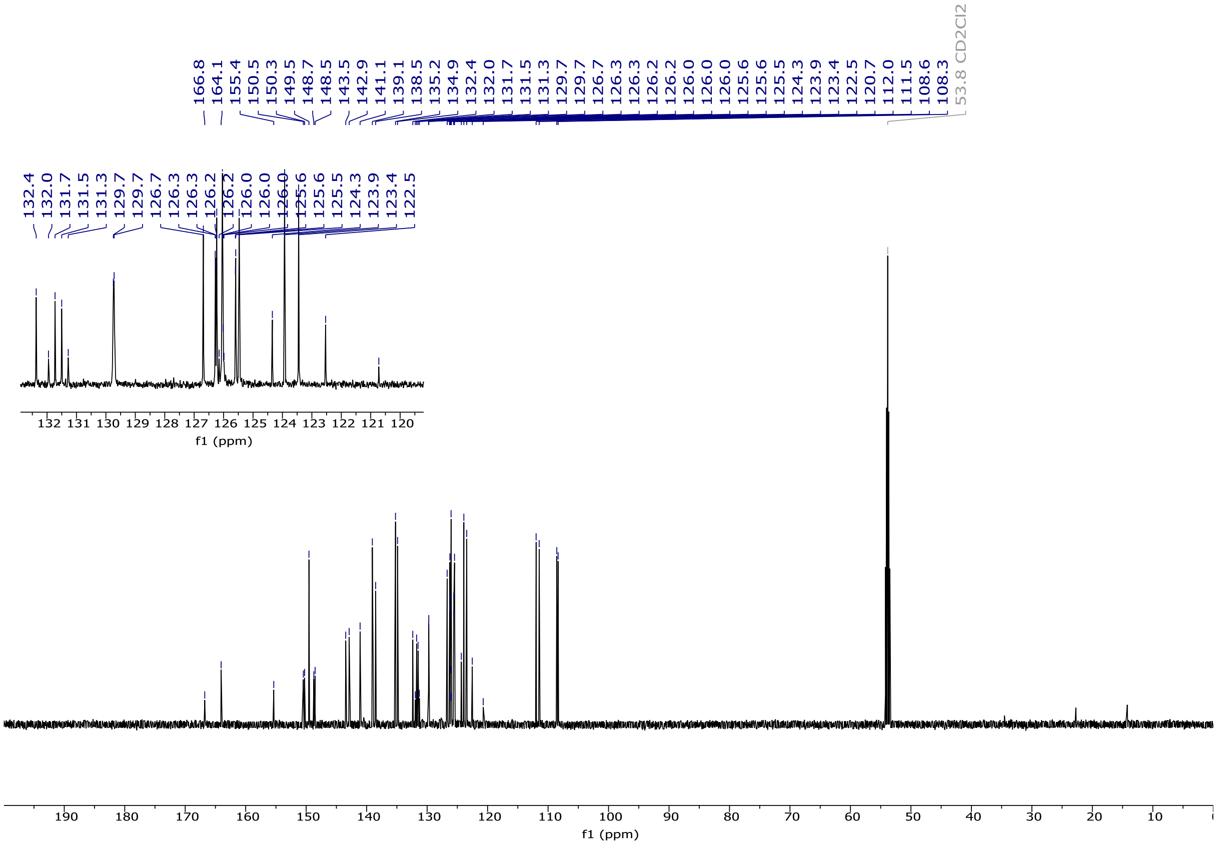

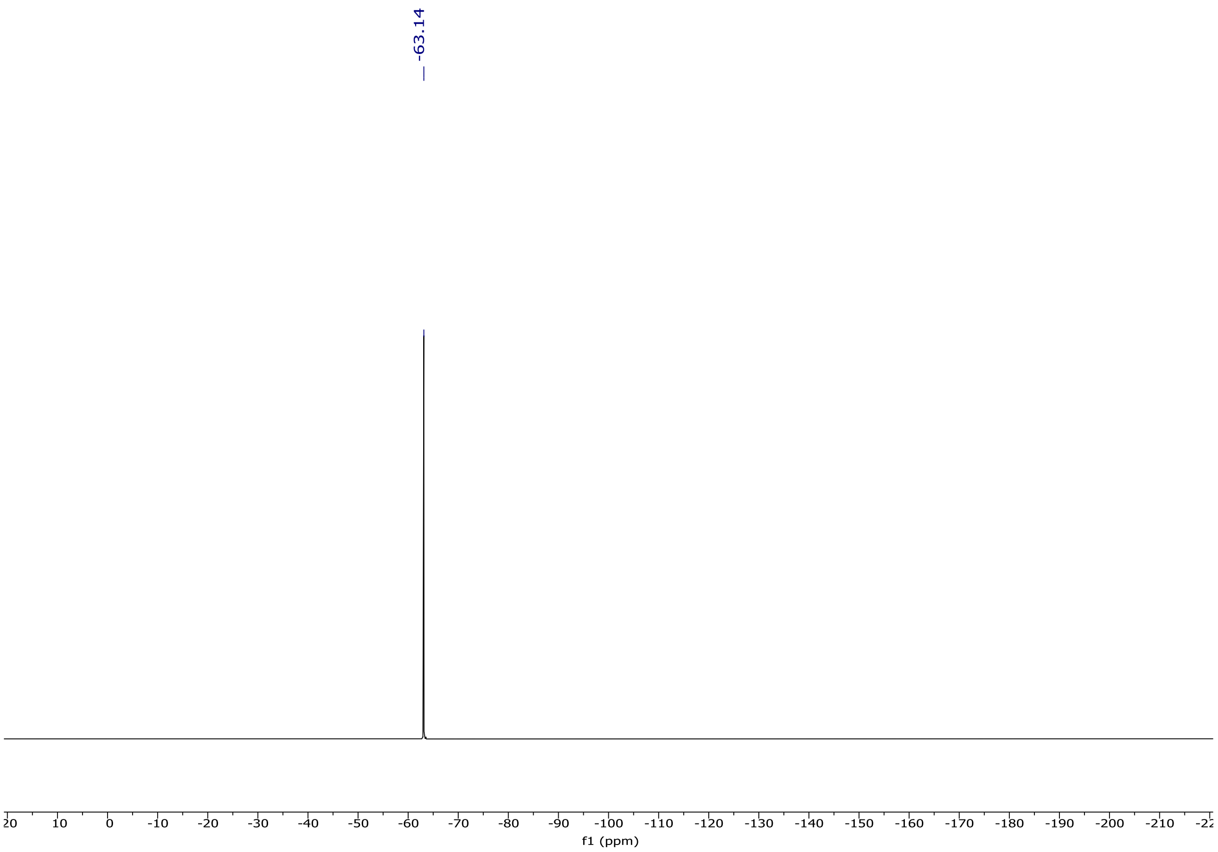
-63.14
20
10
0
-10
-20
-30
-40
-50
-60
-70
-80
-90
-100
-110
-120
-130
-140
-150
-160
-170
-180
-190
-200
-210
f1 (ppm)

[(PzPh)$_2$Rh(^{15}N-1a)], ^{15}N-Rh1a

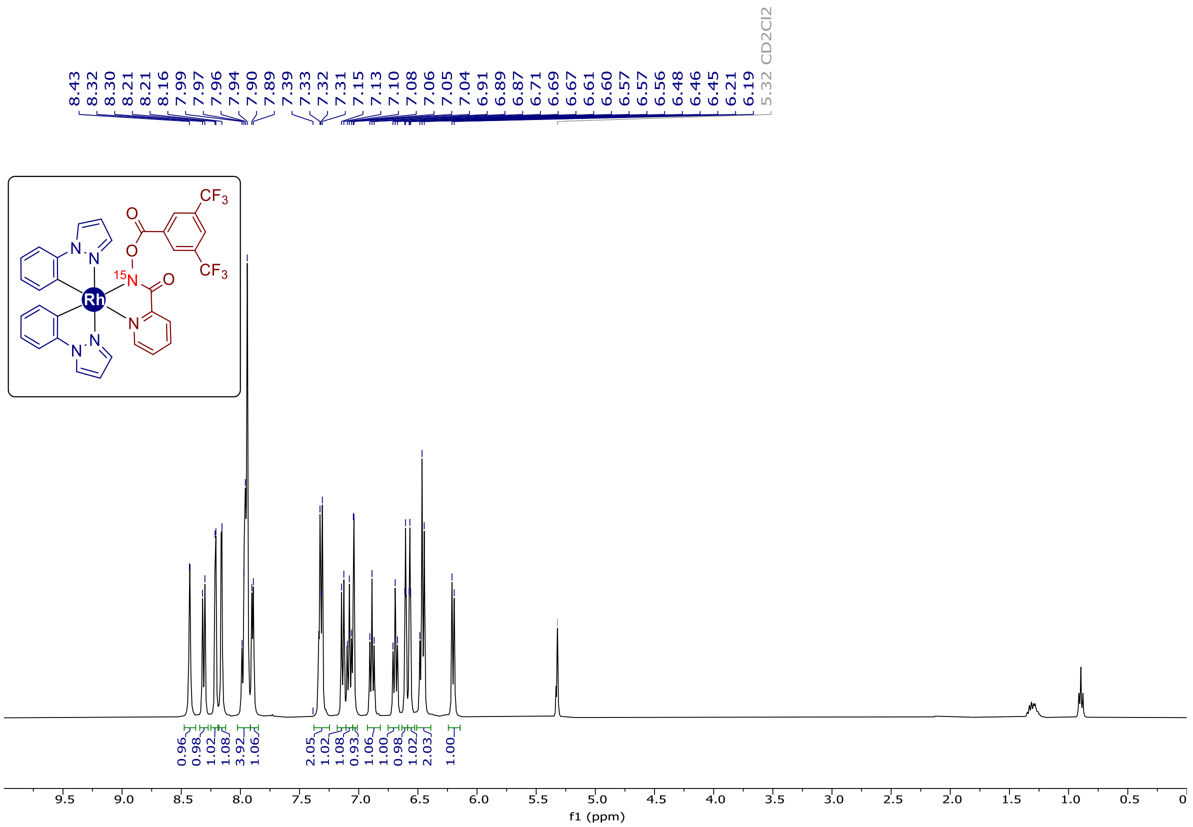

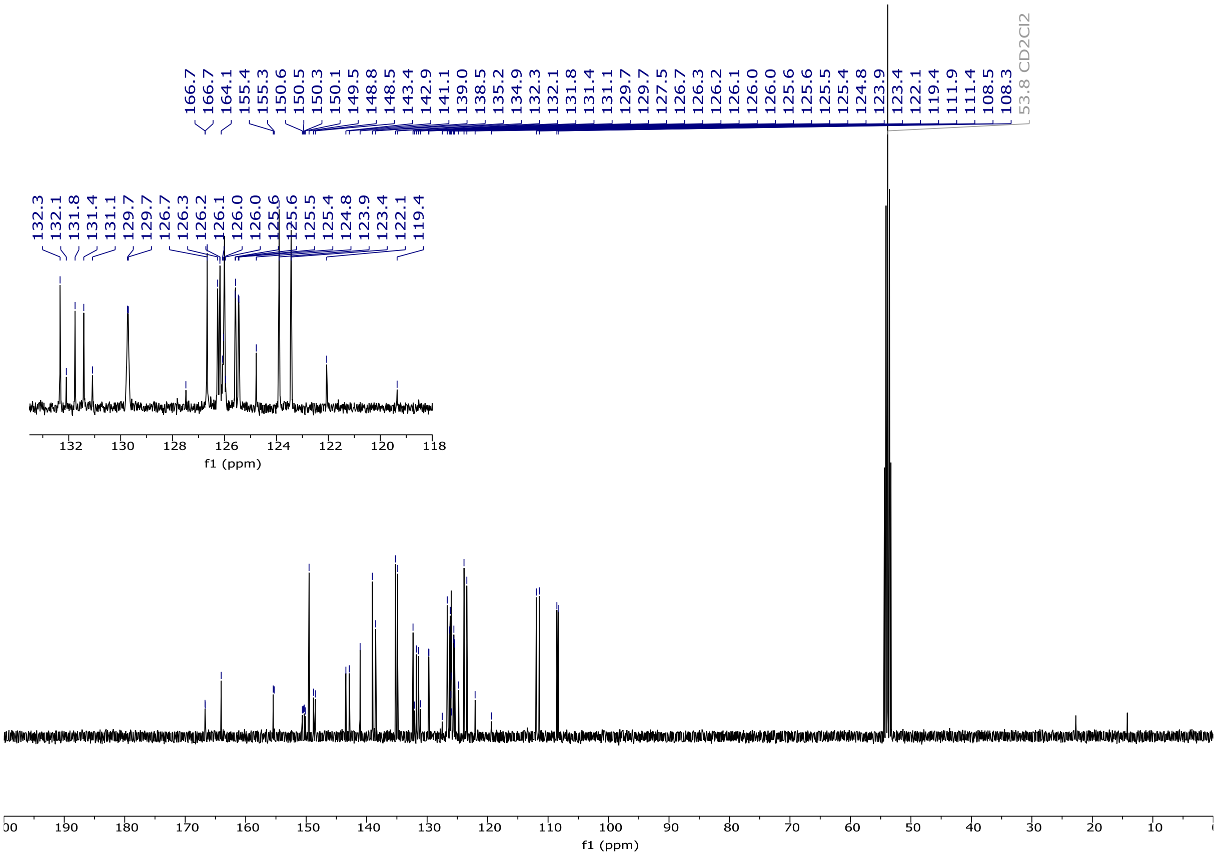

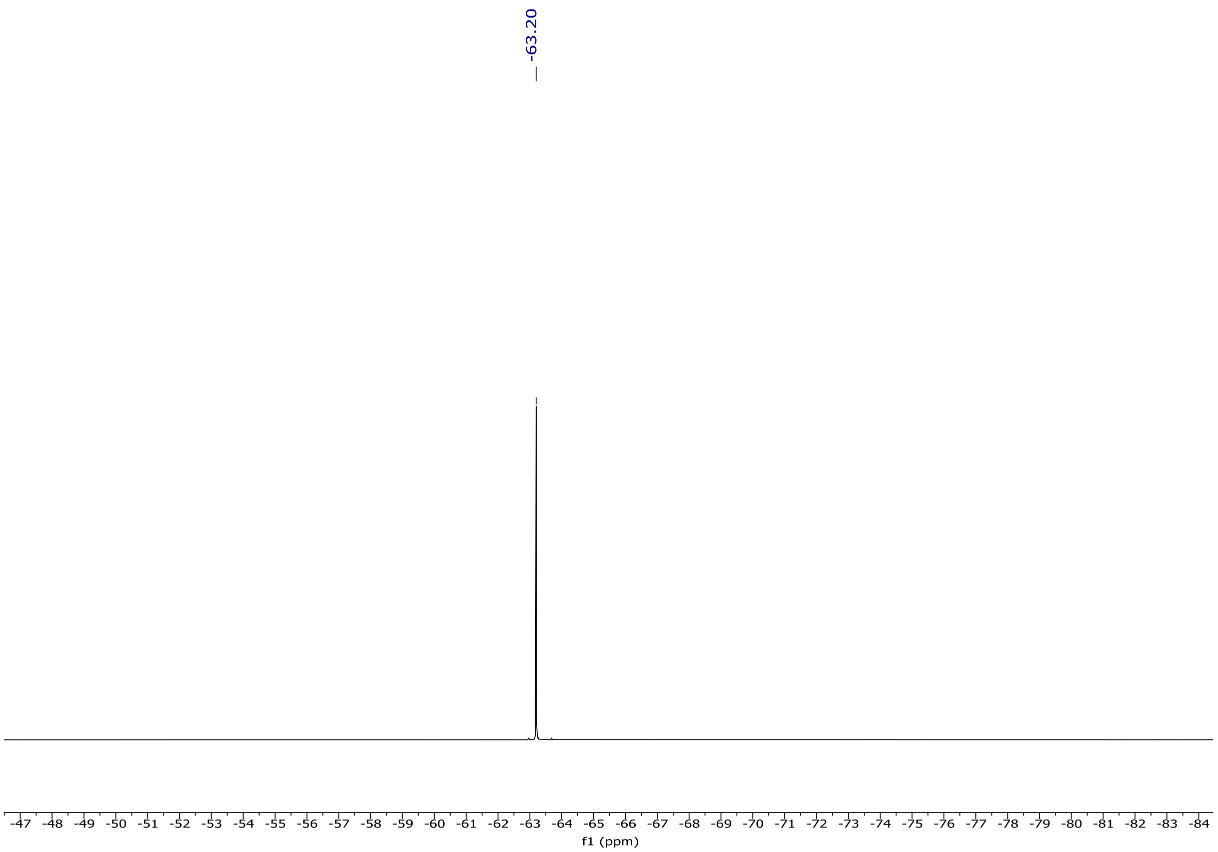
-63.20
-47 -48 -49 -50 -51 -52 -53 -54 -55 -56 -57 -58 -59 -60 -61 -62 -63 -64 -65 -66 -67 -68 -69 -70 -71 -72 -73 -74 -75 -76 -77 -78 -79 -80 -81 -82 -83 -84
f1 (ppm)

[(PzPh)$_2$Rh(1b)], Rh1b

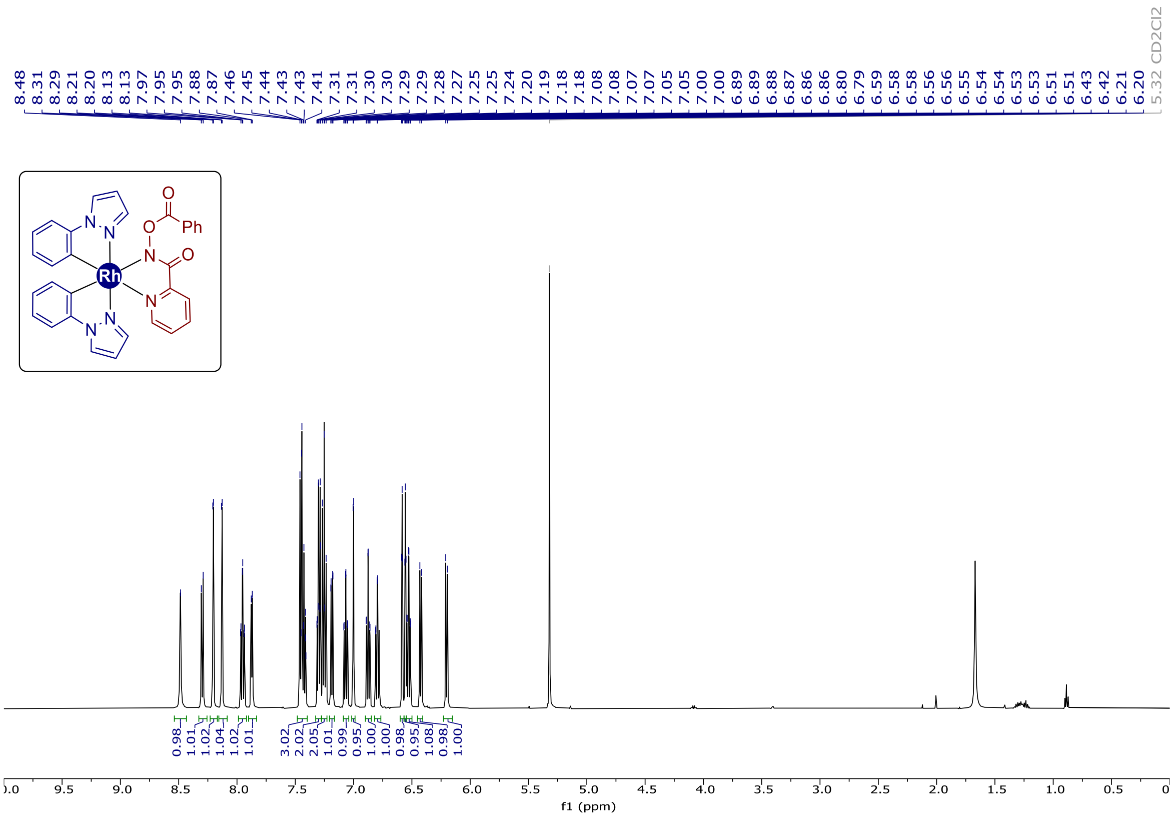

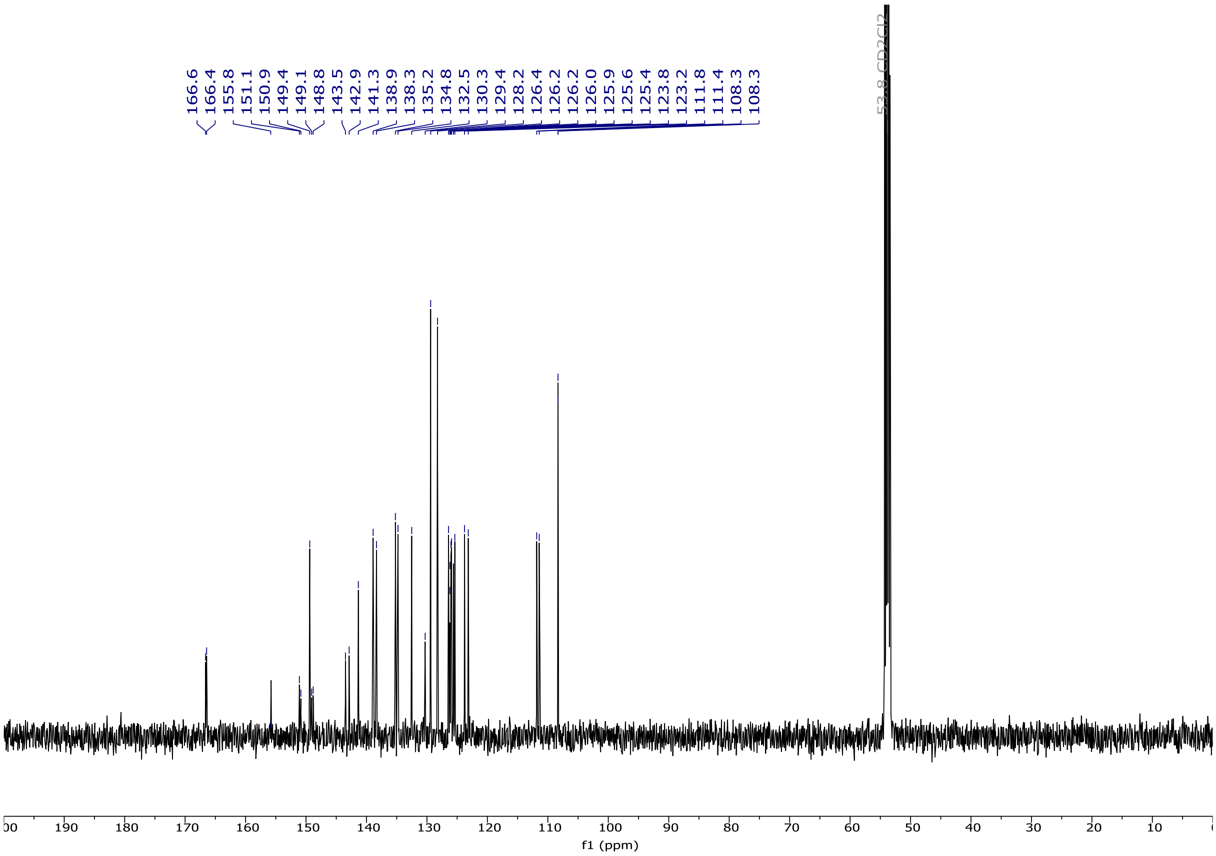

[(PzPh)$_2$Rh(1c)], Rh1c

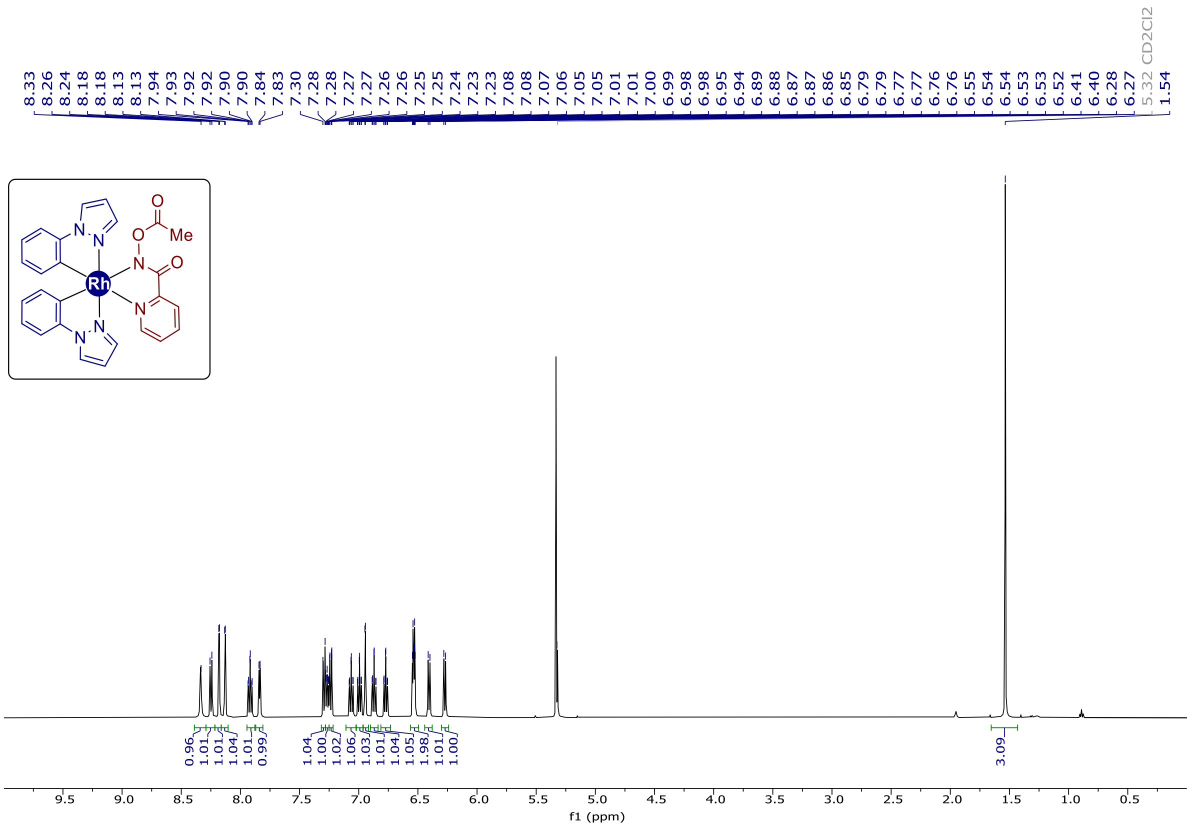

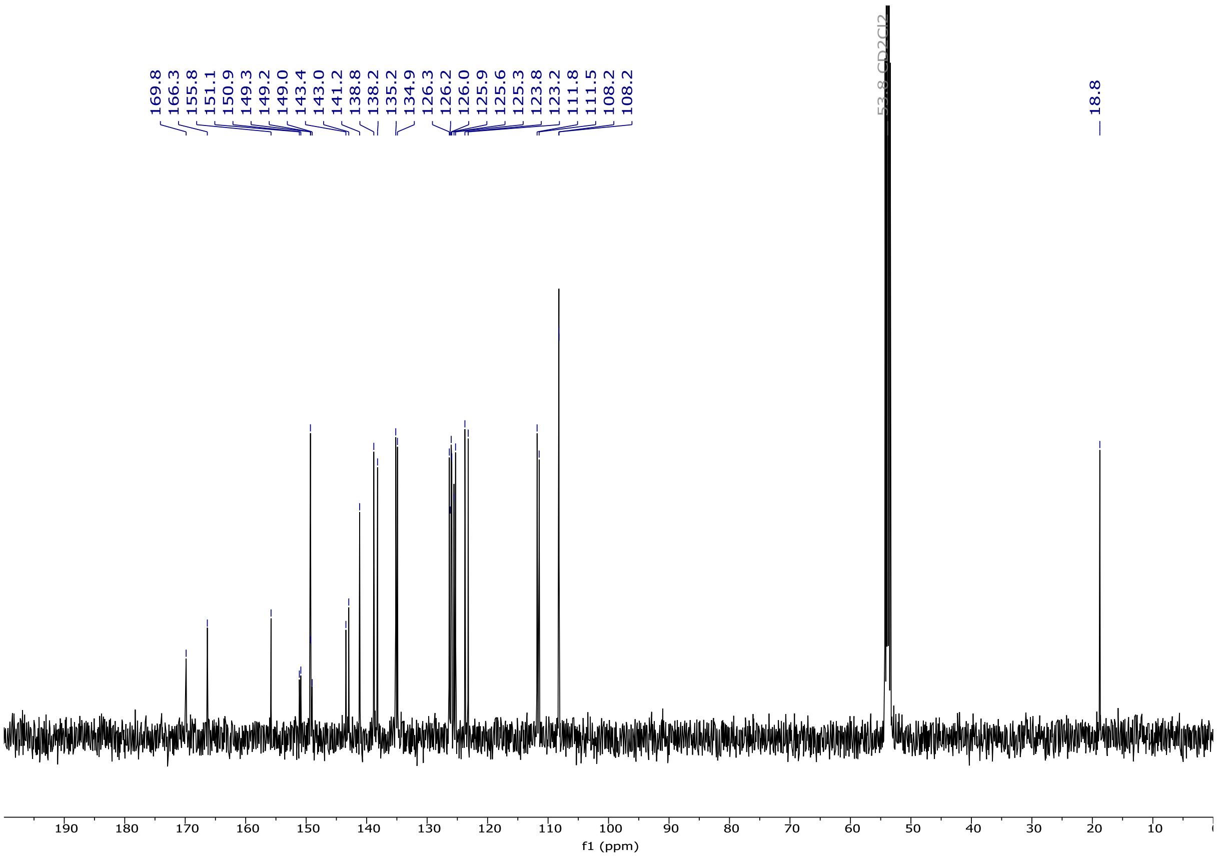

[(PzPh)$_2$Rh(1d)], Rh1d

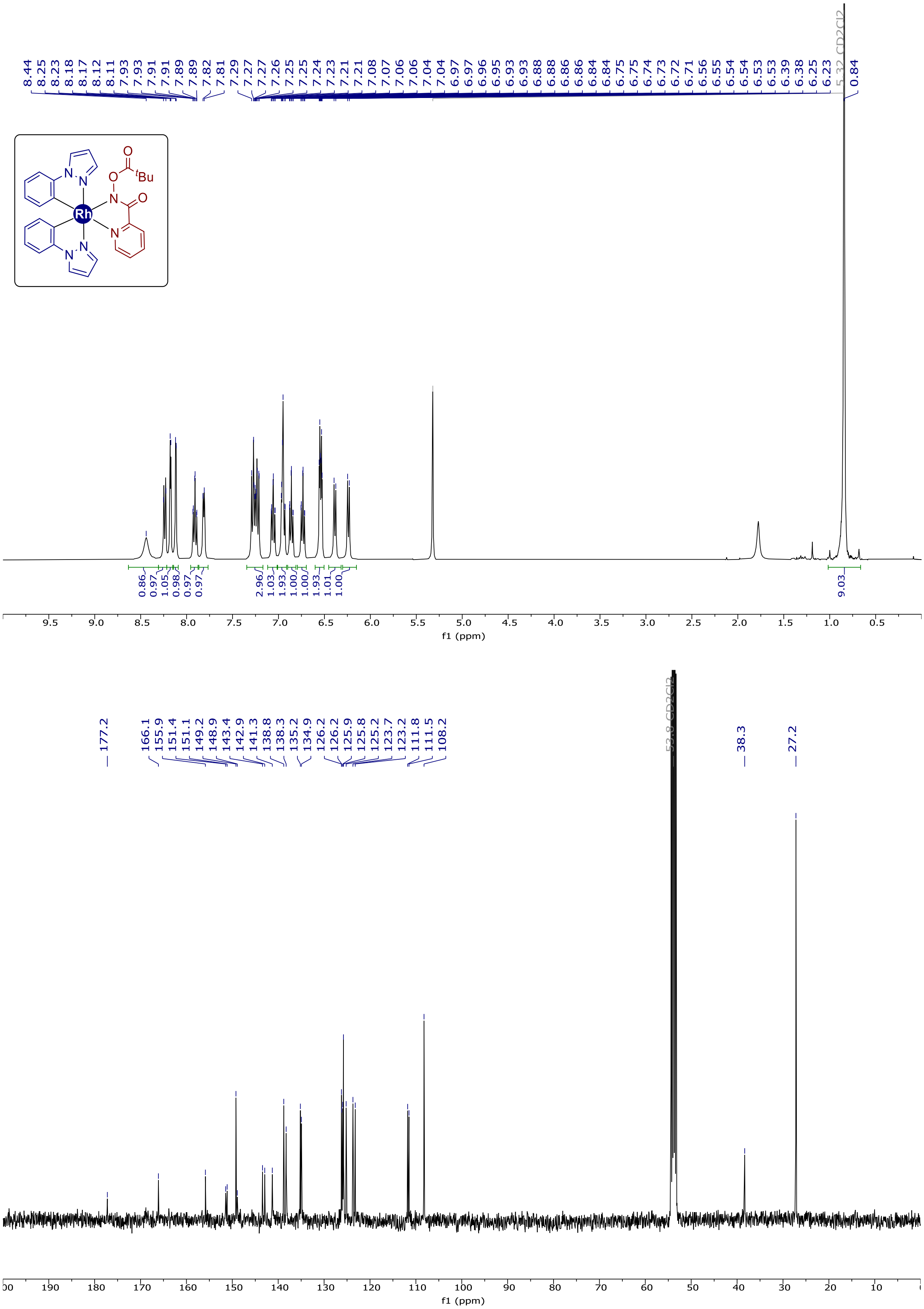

[(PzPh)$_2$Rh(1e)], Rh1e

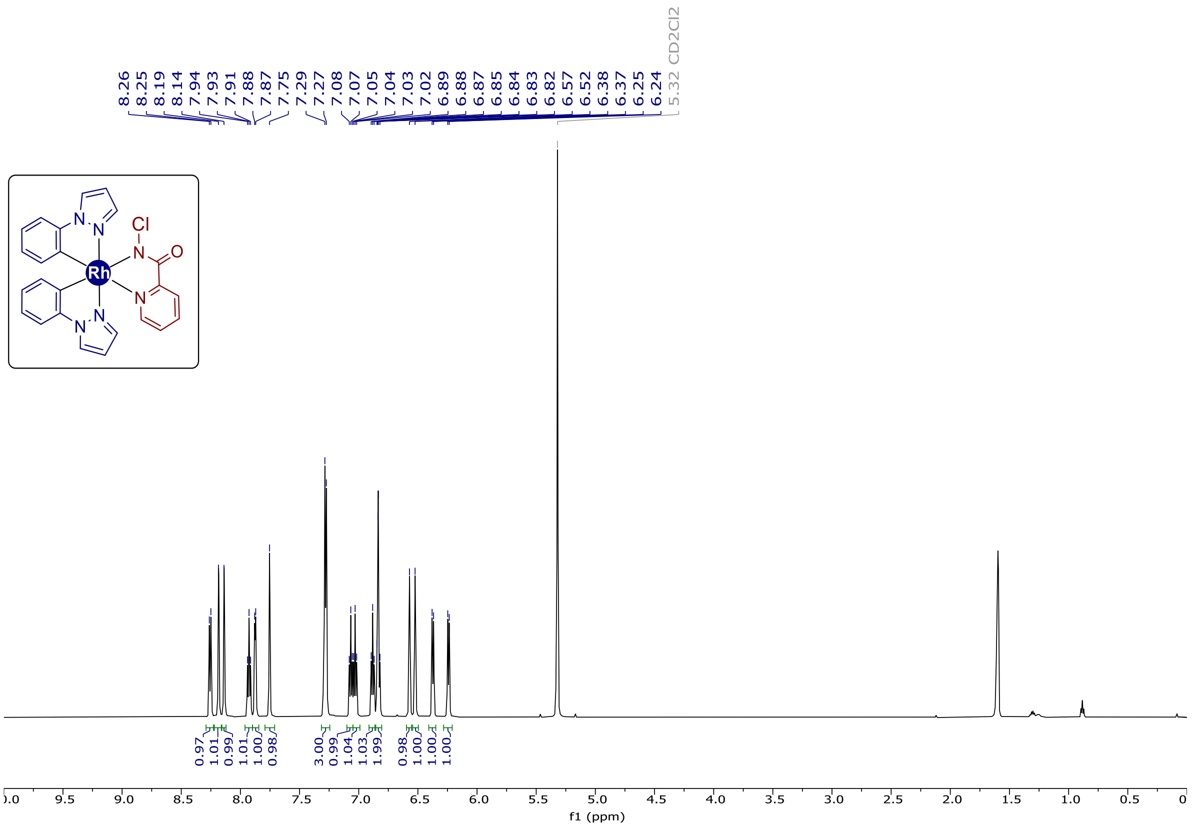

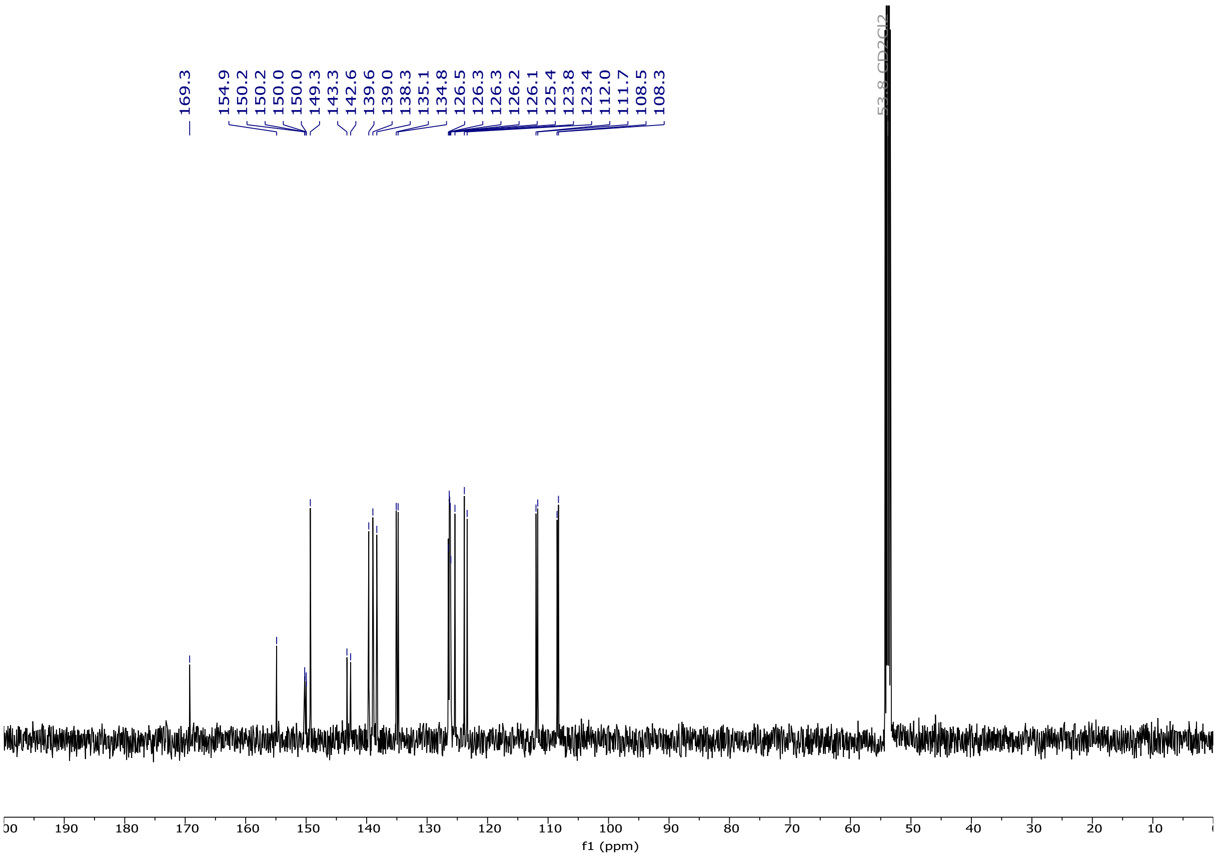

[$(CF_3PzPh)_2Rh(1f)$], Rh1f

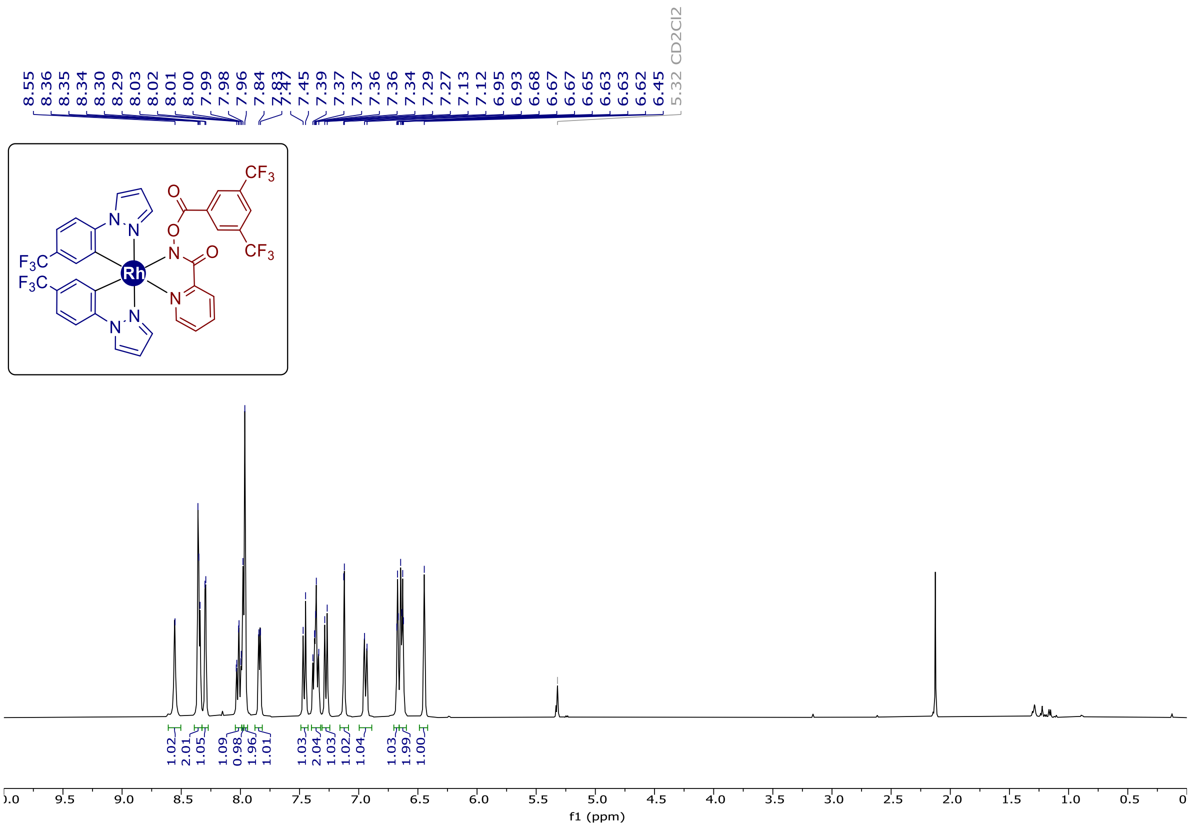

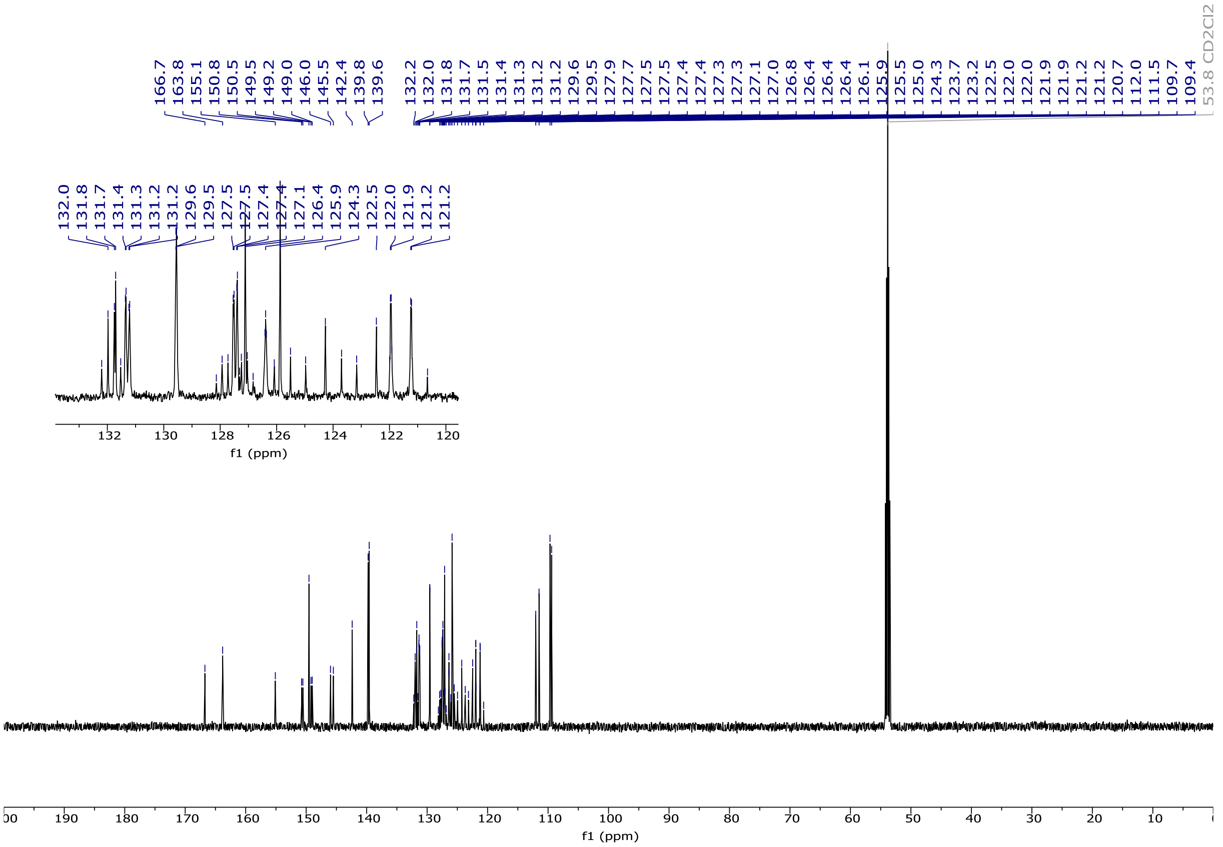

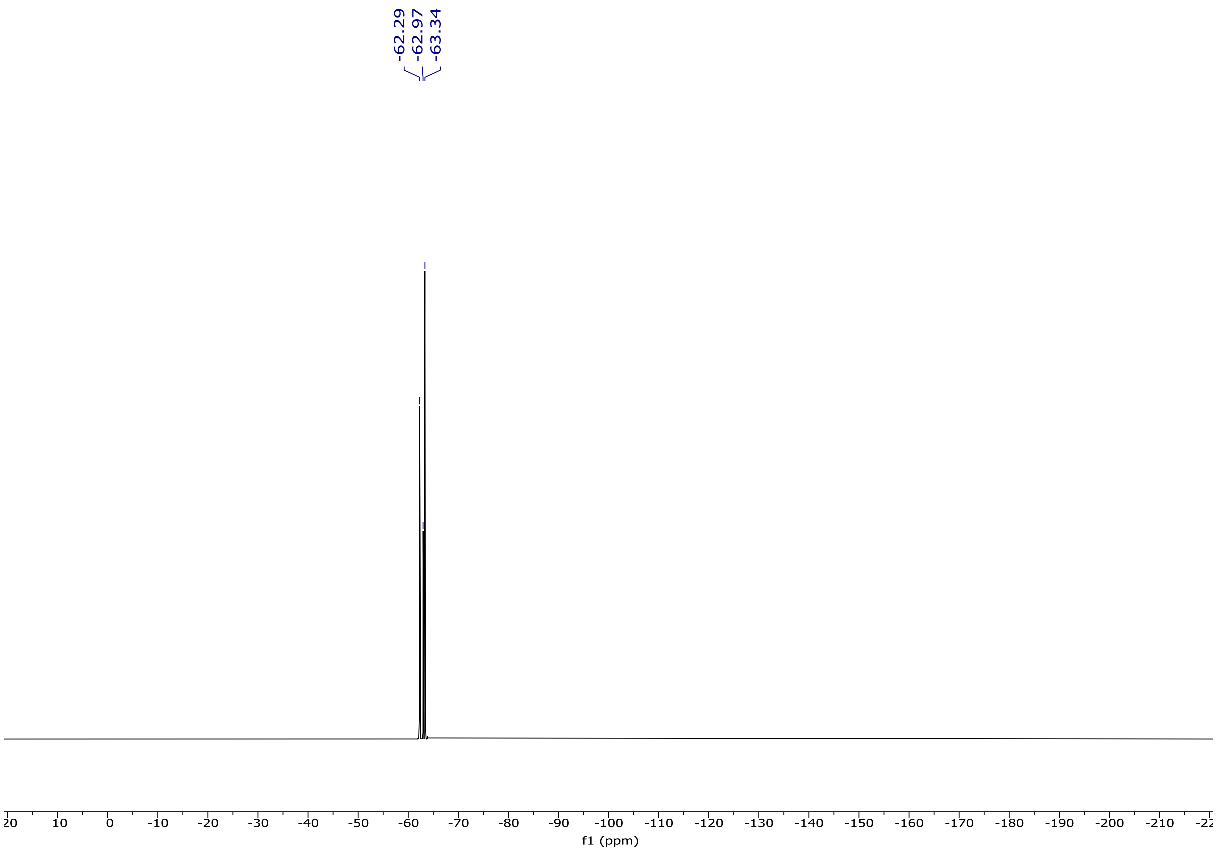

-62.29
-62.97
-63.34
20 10 0 -10 -20 -30 -40 -50 -60 -70 -80 -90 -100 -110 -120 -130 -140 -150 -160 -170 -180 -190 -200 -210
f1 (ppm)

[(PhPy)$_2$Rh(1a)], Rh3

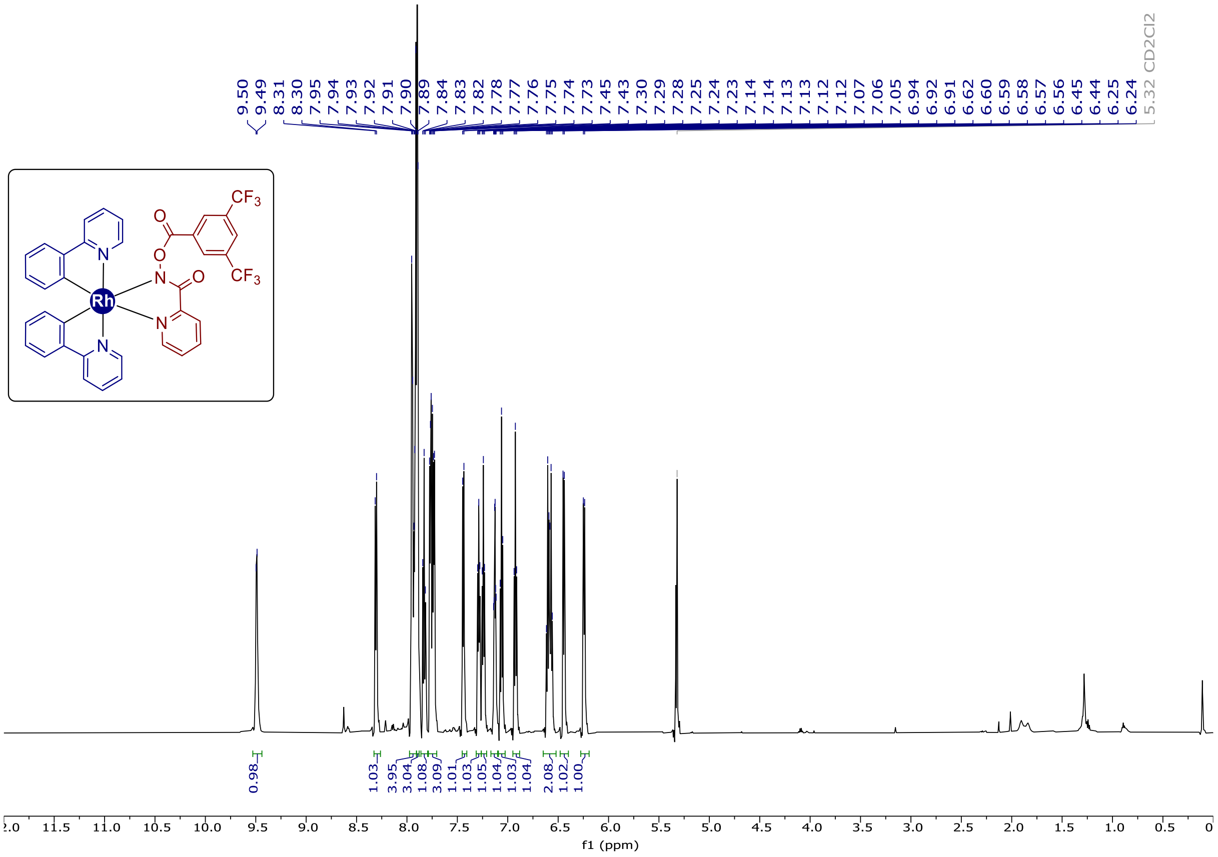

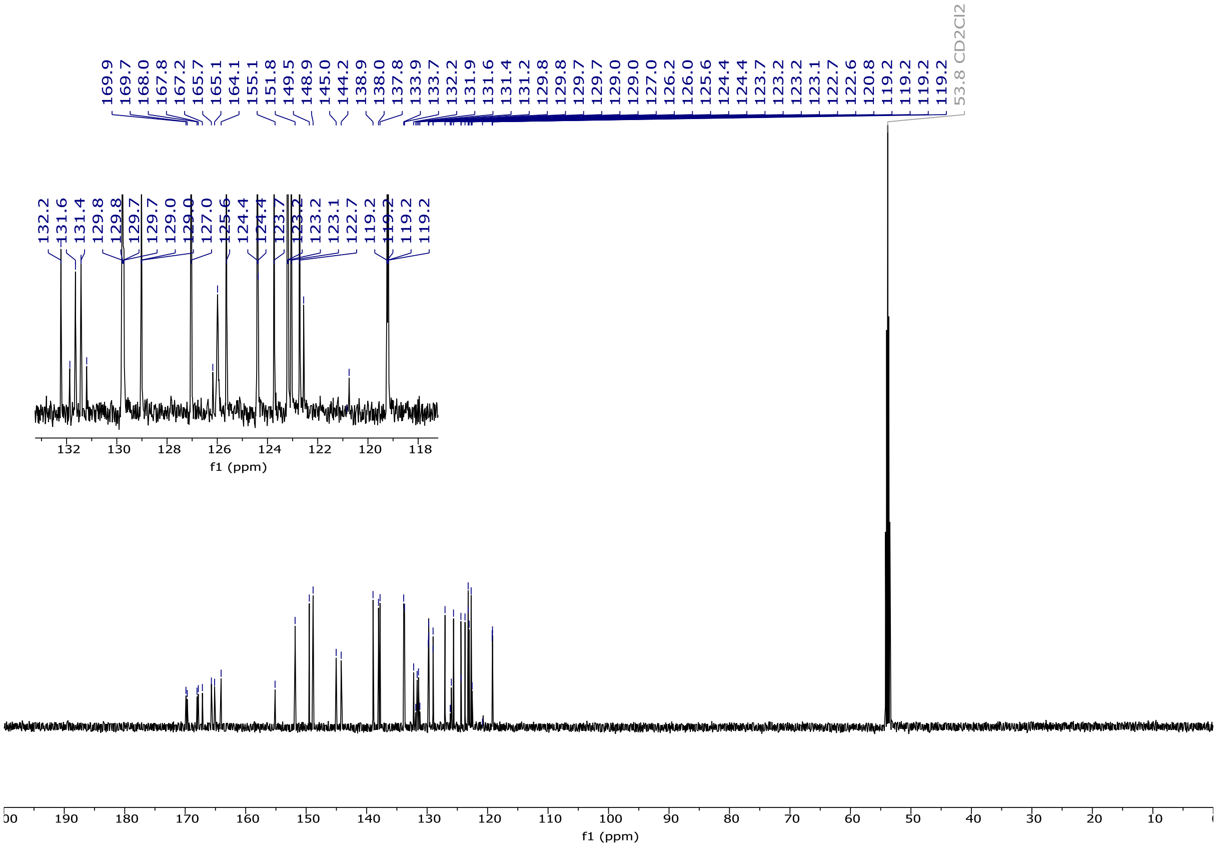

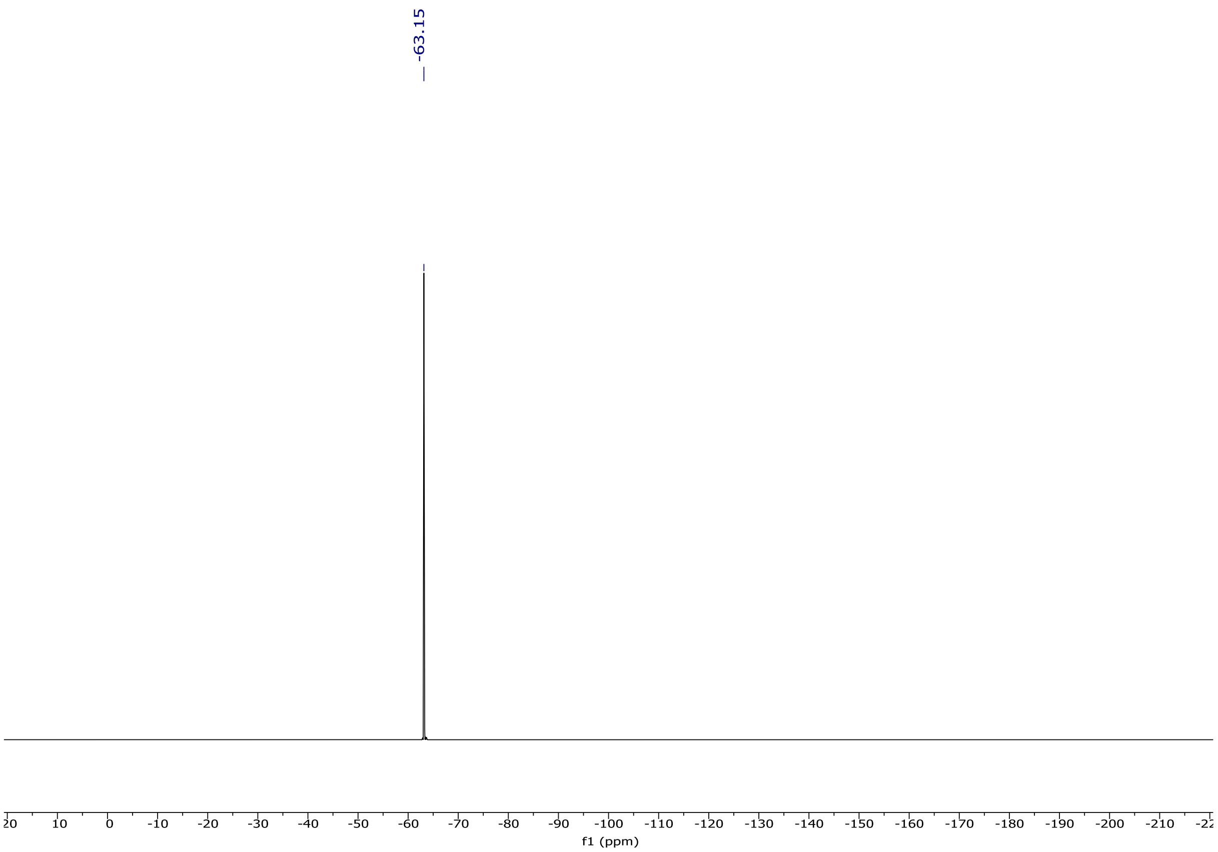
-63.15
20 10 0 -10 -20 -30 -40 -50 -60 -70 -80 -90 -100 -110 -120 -130 -140 -150 -160 -170 -180 -190 -200 -210 -22
f1 (ppm)

[(PhTh)$_2$Rh(1a)], Rh4

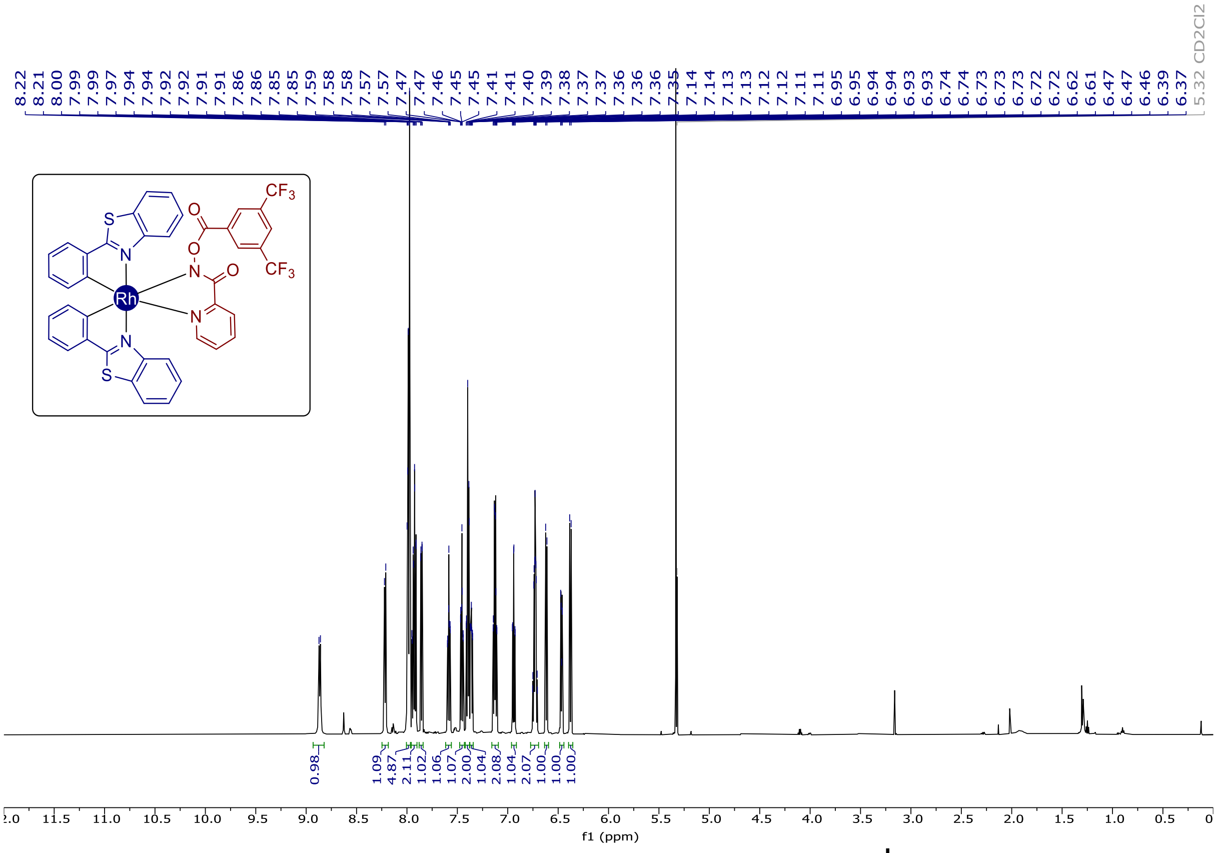

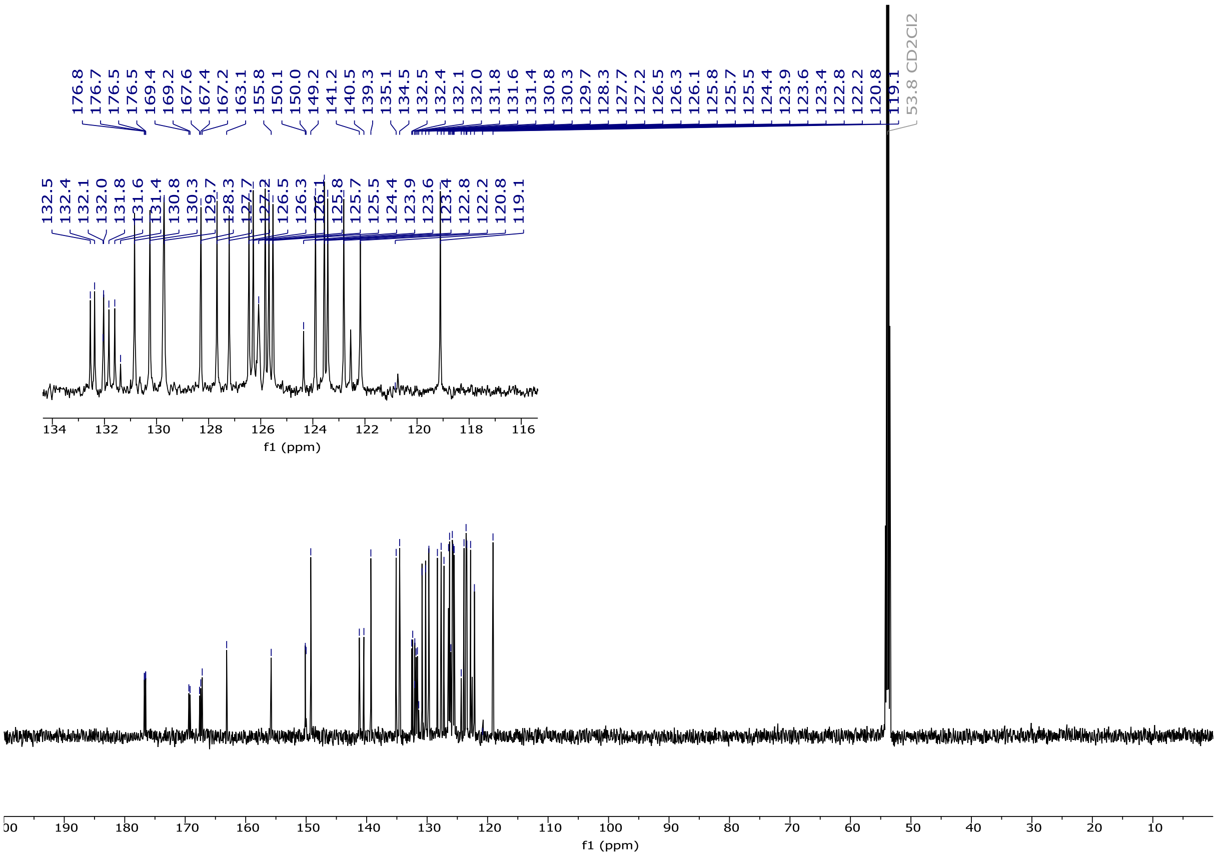

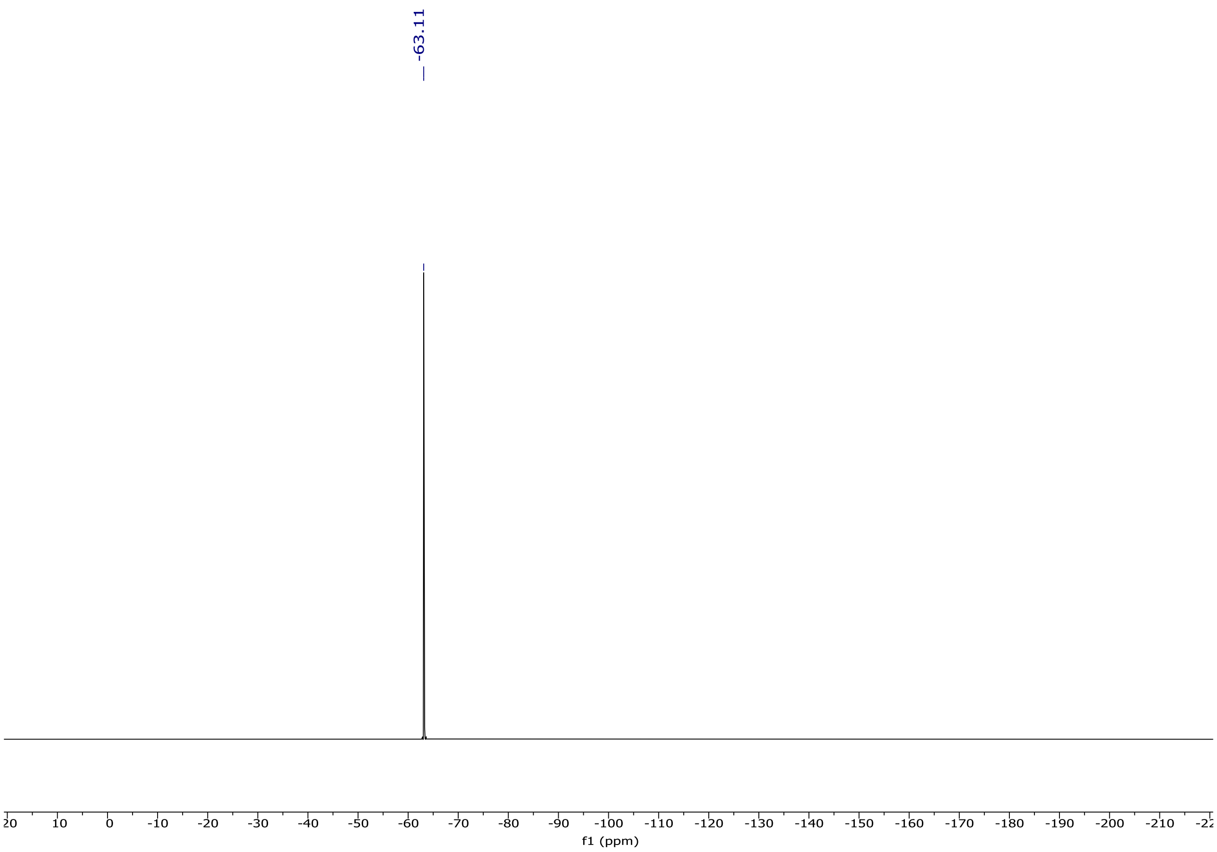

-63.11
20 10 0 -10 -20 -30 -40 -50 -60 -70 -80 -90 -100 -110 -120 -130 -140 -150 -160 -170 -180 -190 -200 -210
f1 (ppm)

[(PhInd)$_2$Rh(1a)], Rh5

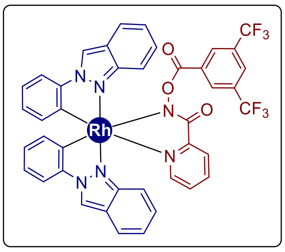

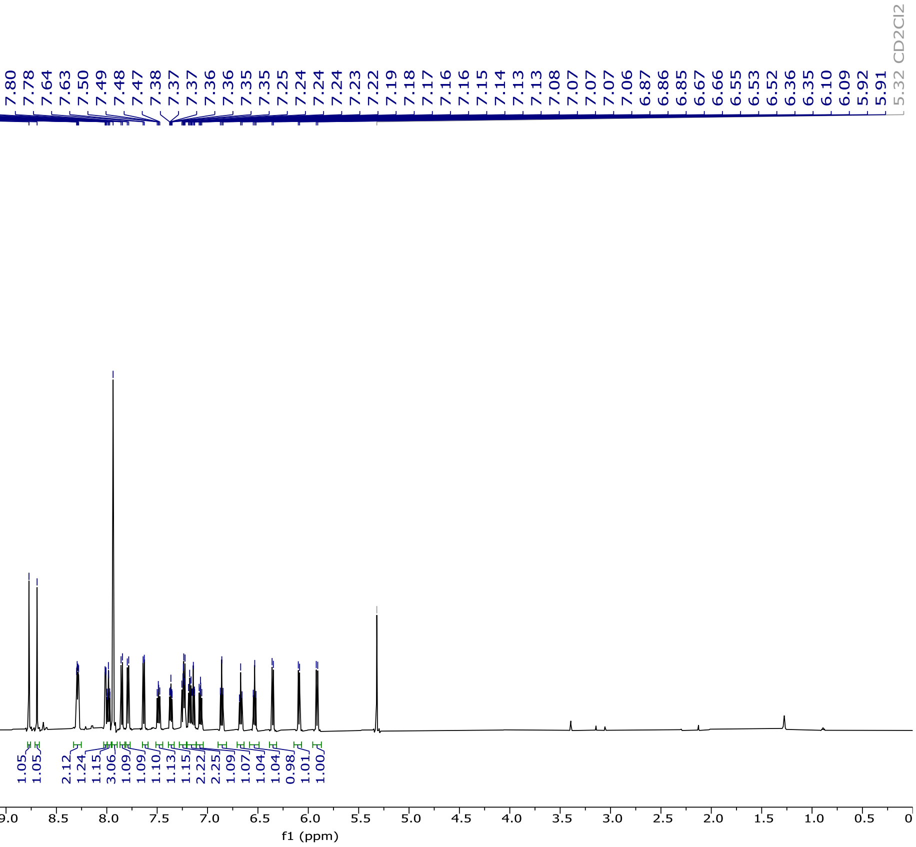

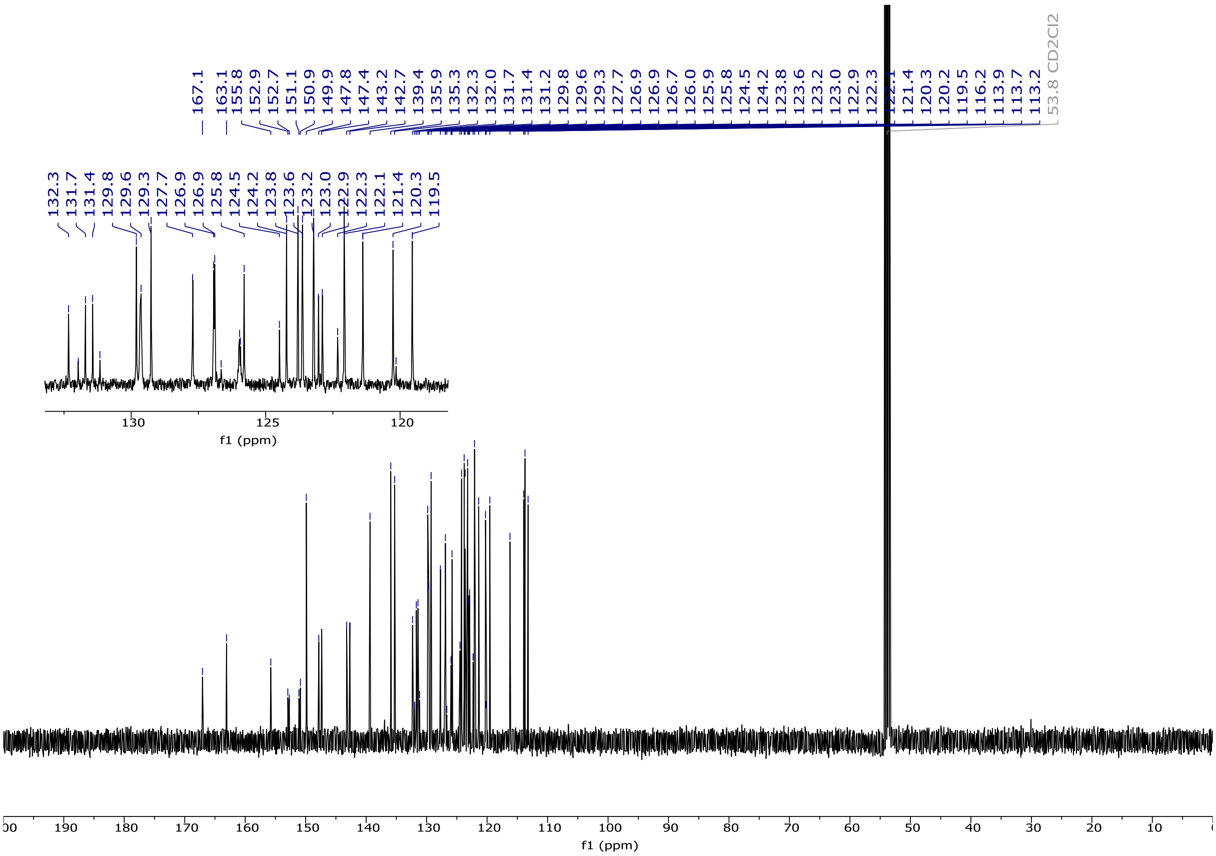

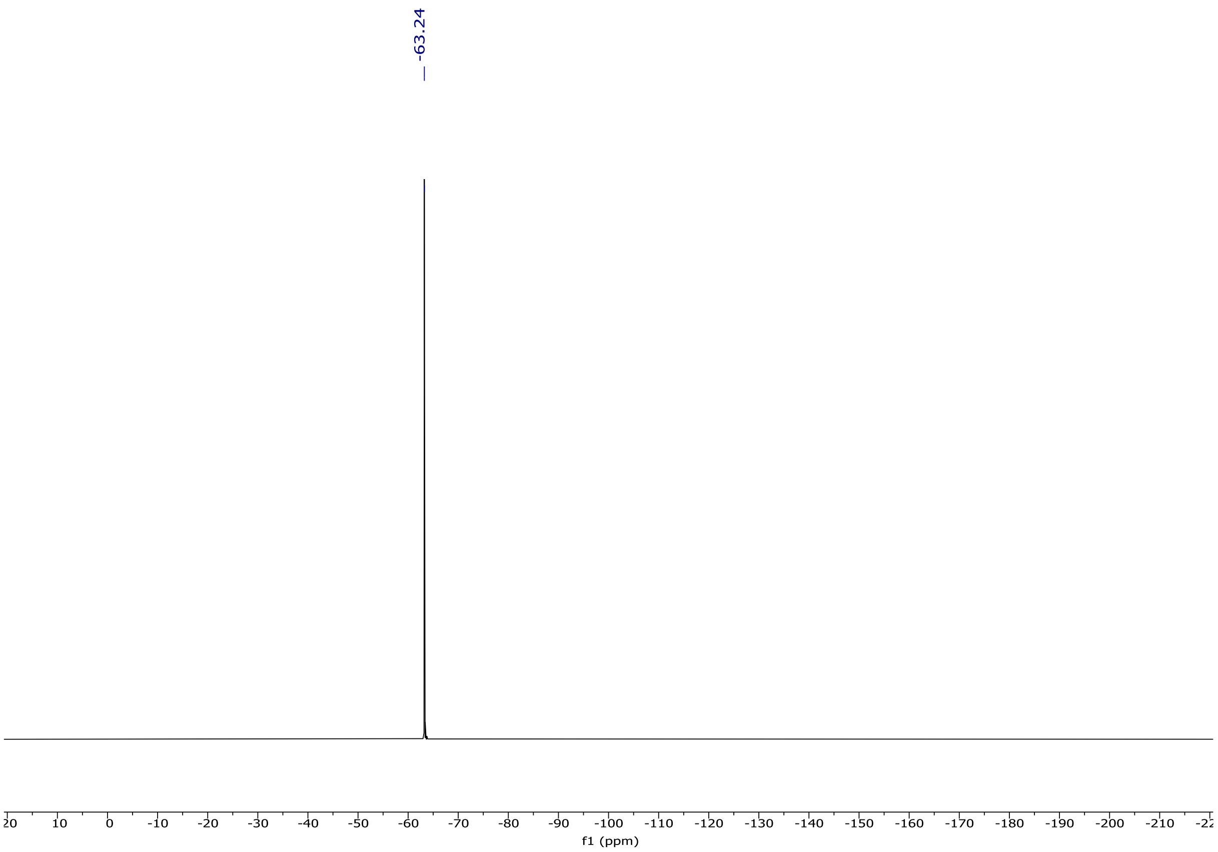
-63.24
20
10
0
-10
-20
-30
-40
-50
-60
-70
-80
-90
-100
-110
-120
-130
-140
-150
-160
-170
-180
-190
-200
-210
f1 (ppm)

[(PzPh)$_2$Rh(2)], Rh2

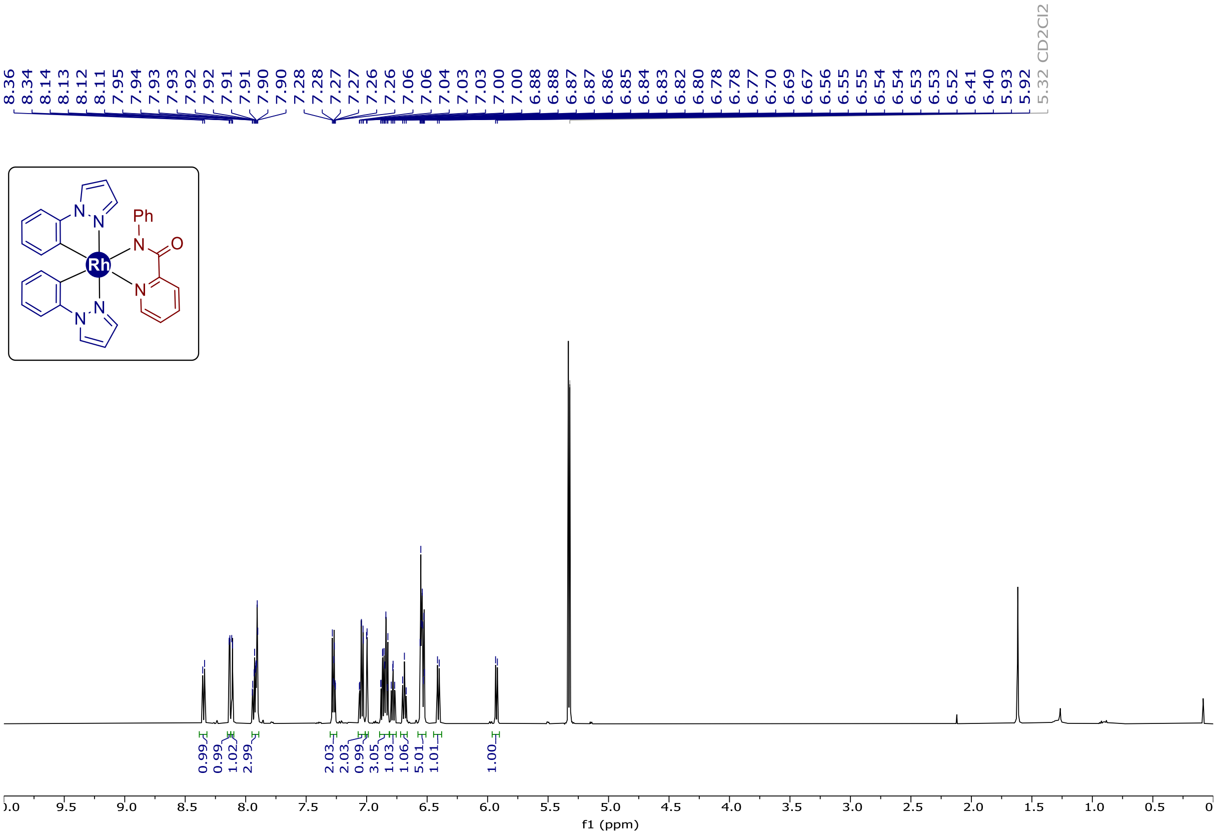

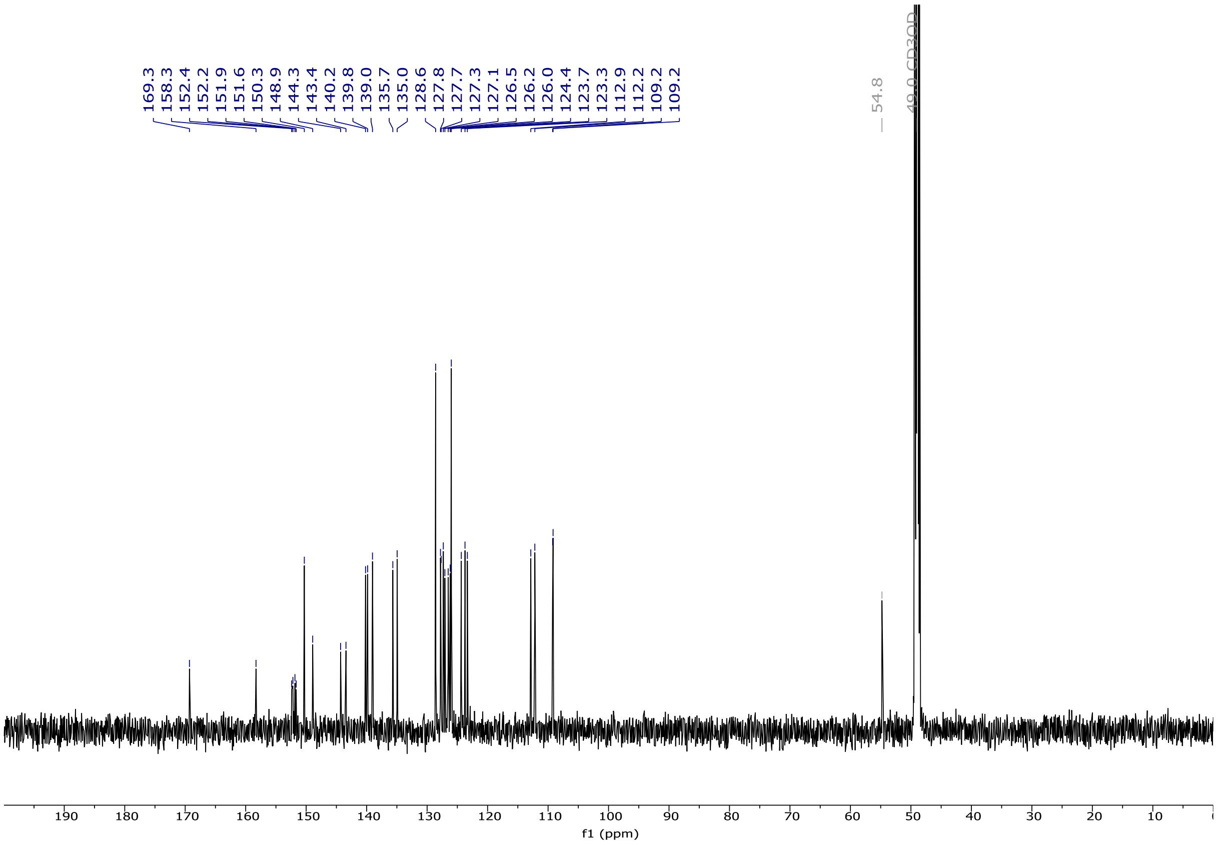

[(PzPh)$_2$Rh(1f)], Rh6

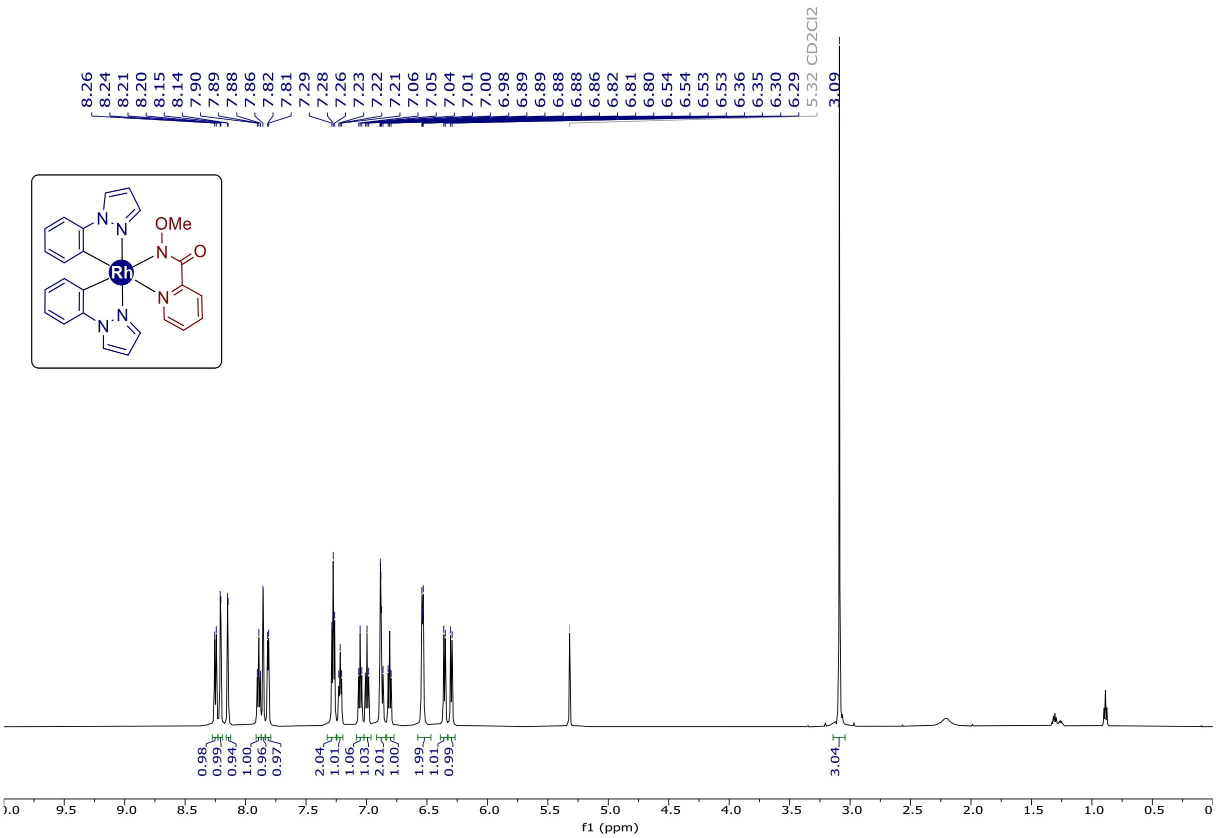

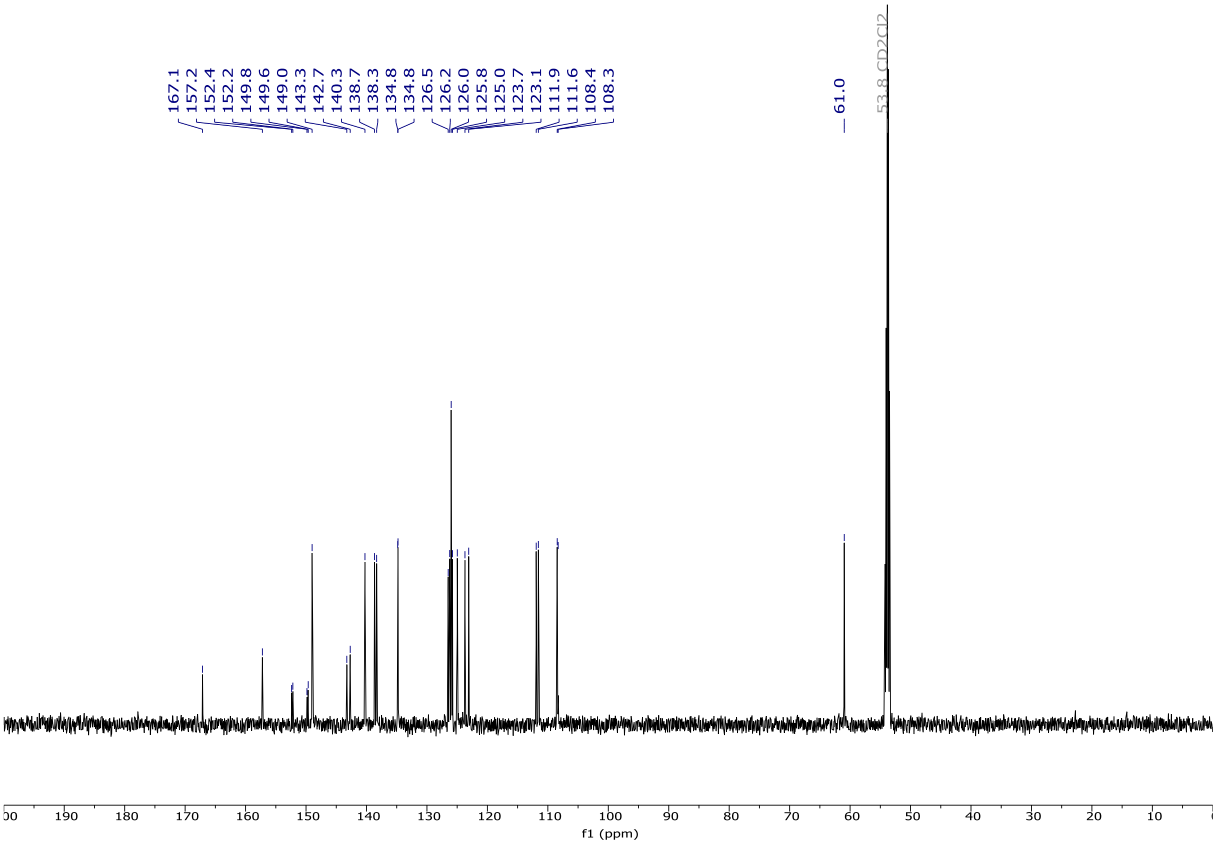

N-Phenylpicolinamide (2)

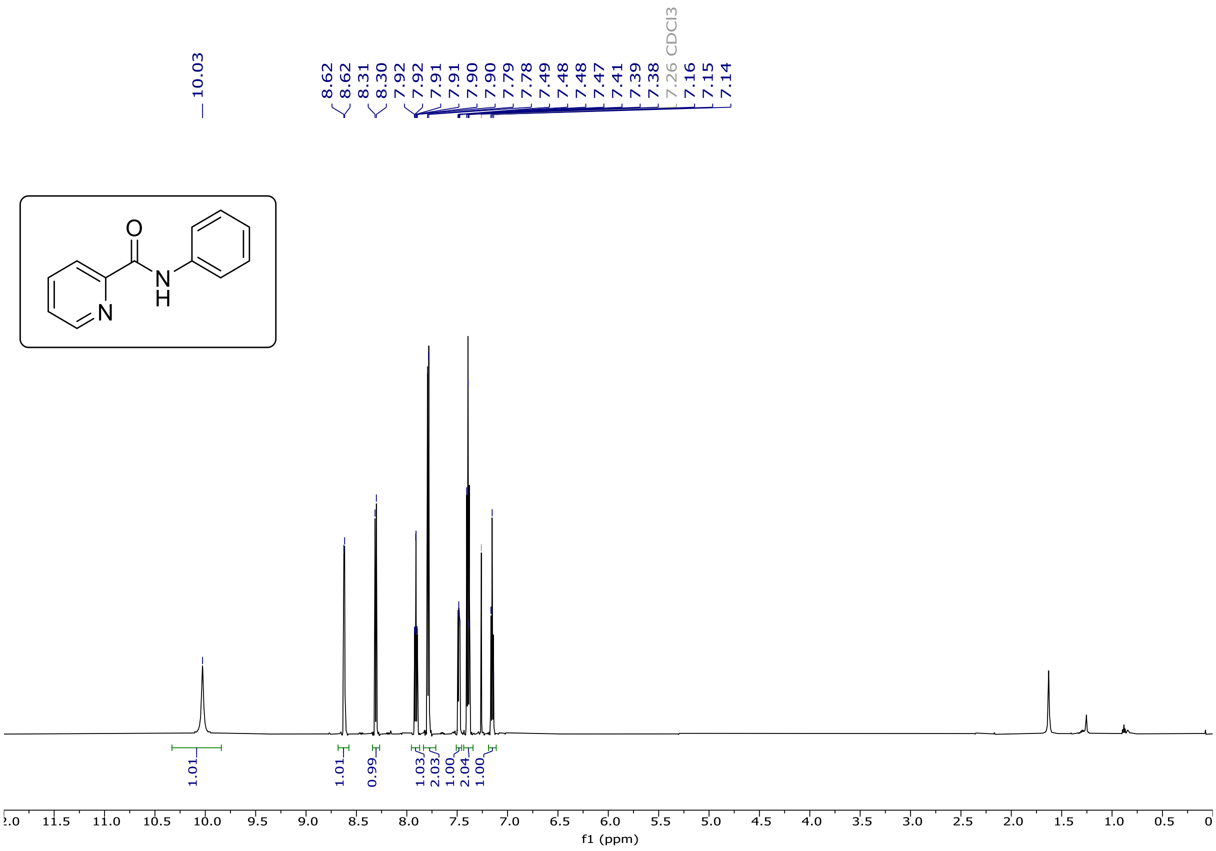

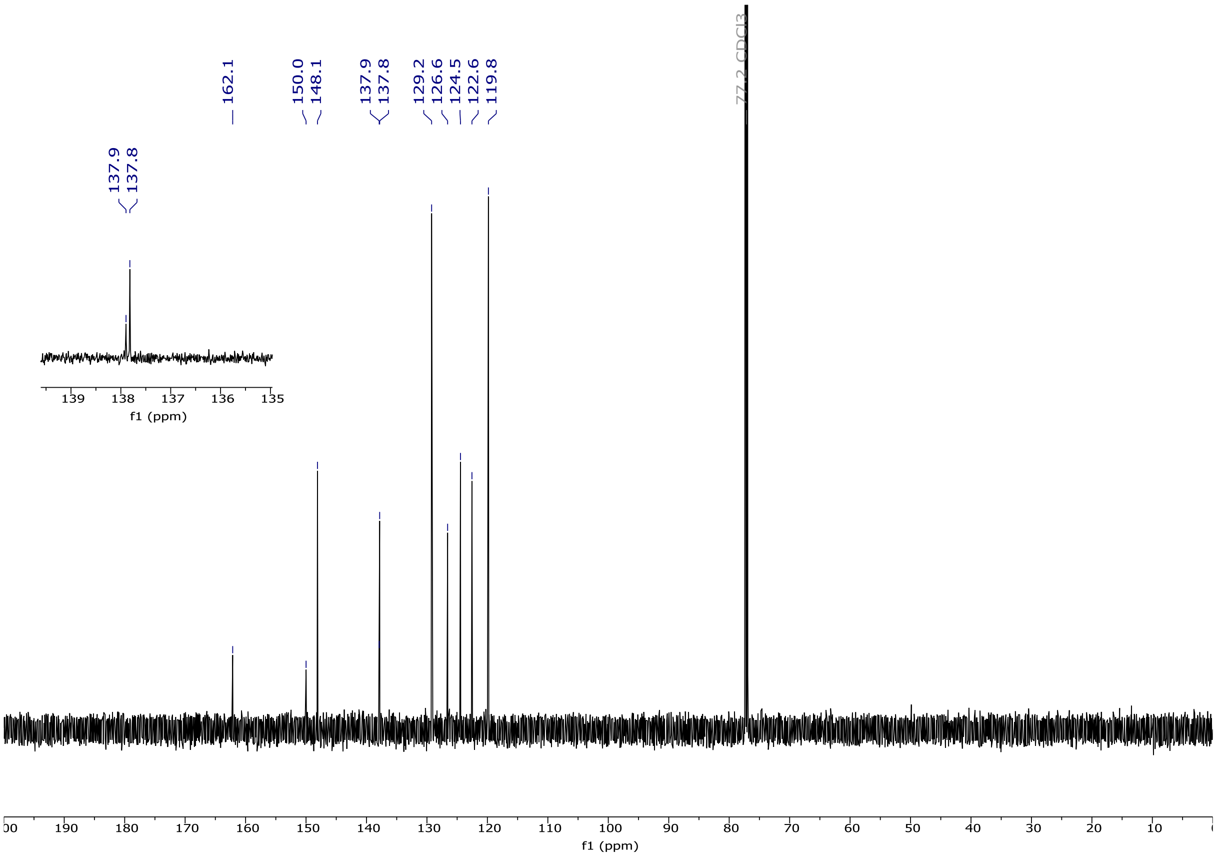

N-Cyclohexylpicolinamide (3)

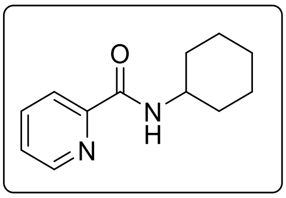

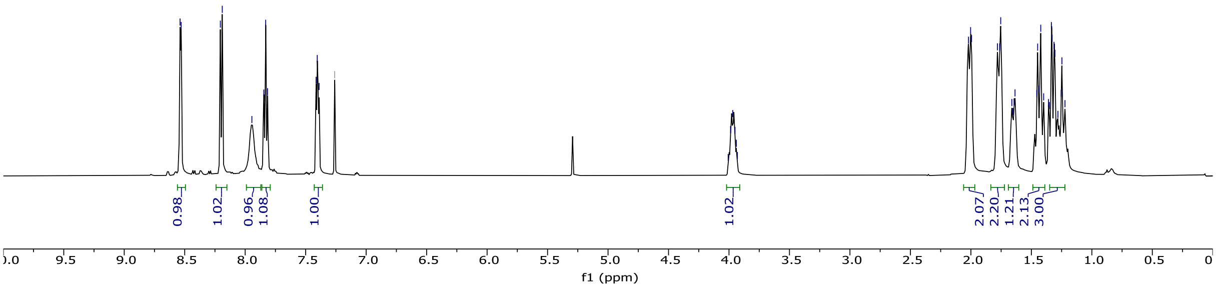

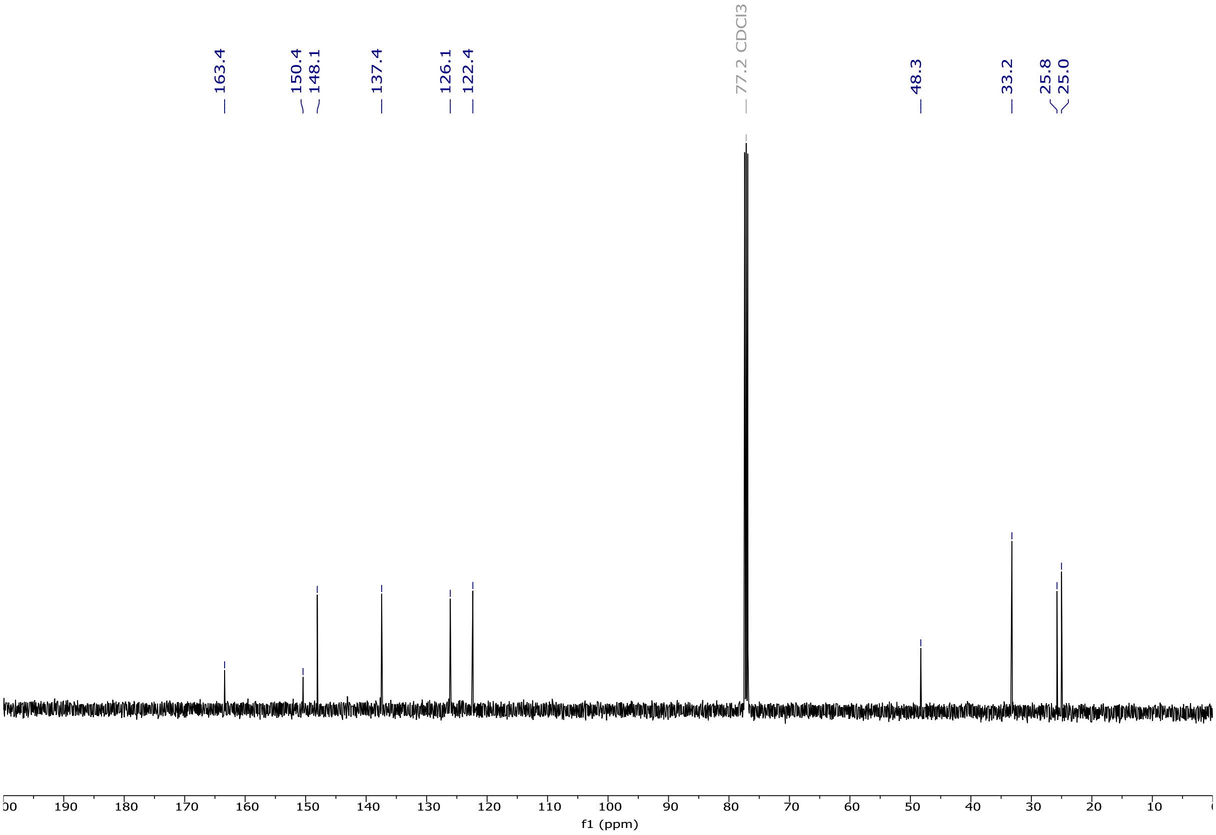

(*R*)-*N*-(1-Chloro-2-methylbutan-2-yl)picolinamide ((*R*)-4)

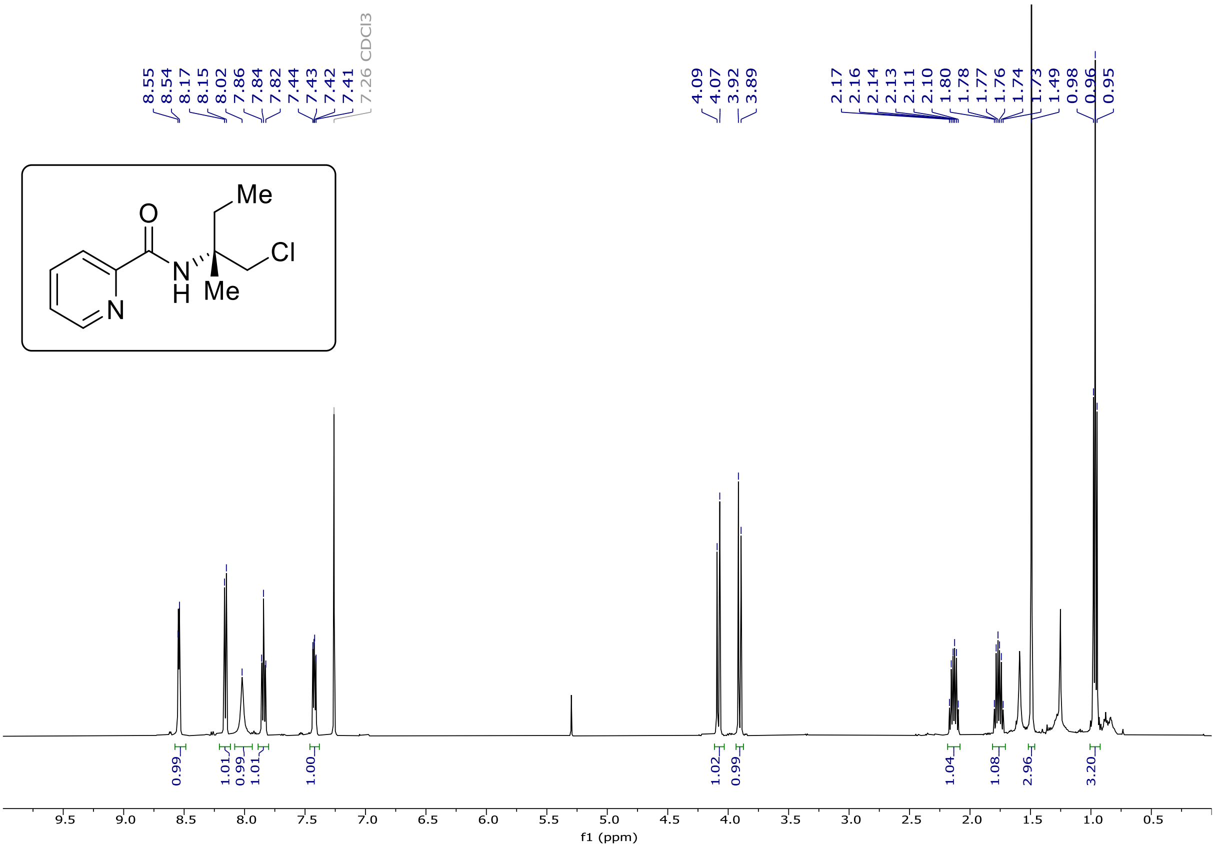

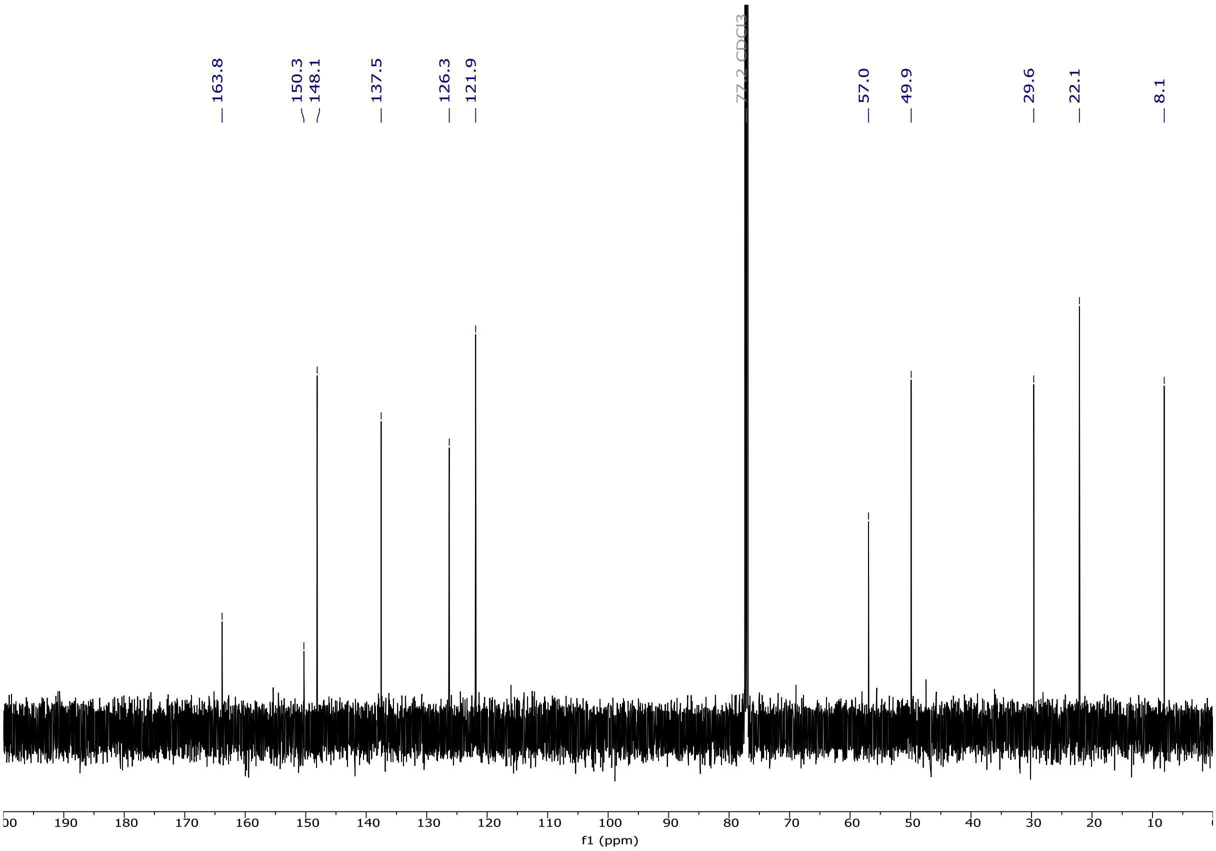

Mixture of *N*-(*o*-tolyl)picolinamide (5o), *N*-(*m*-tolyl)picolinamide (5m) and *N*-(*p*-tolyl)picolinamide (5p)

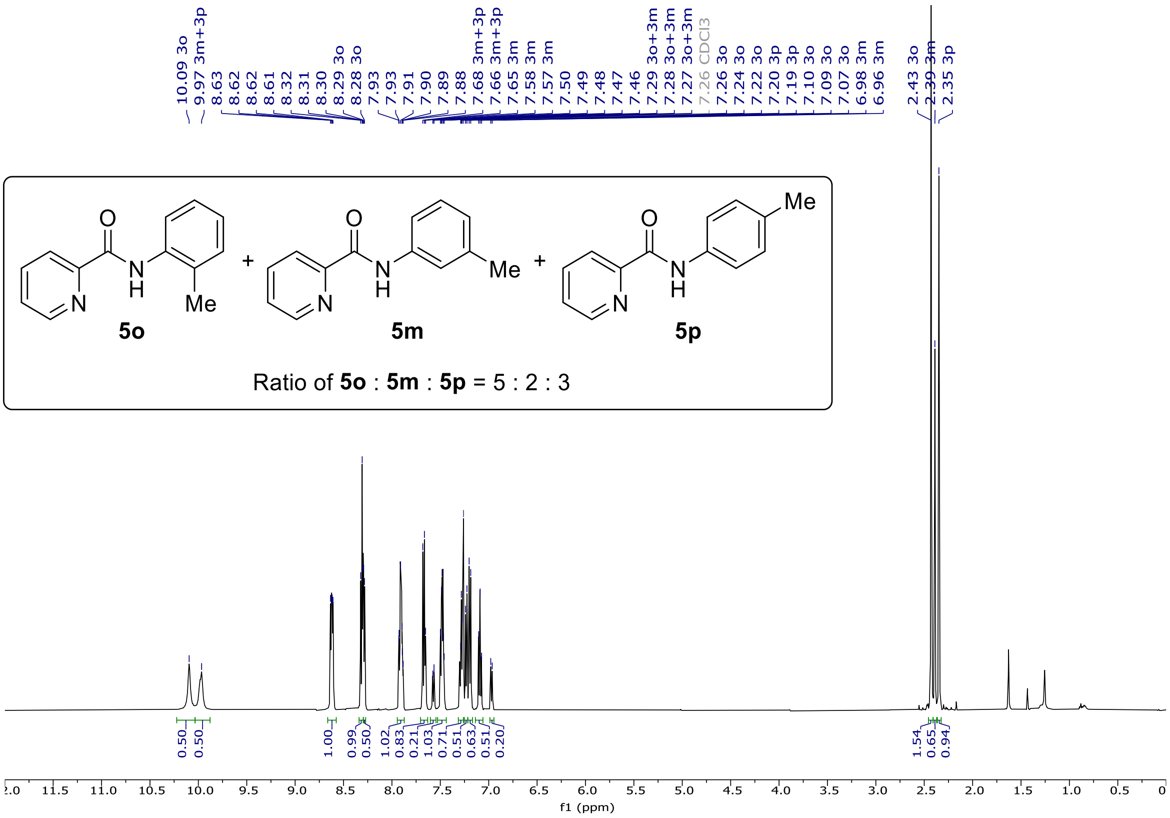

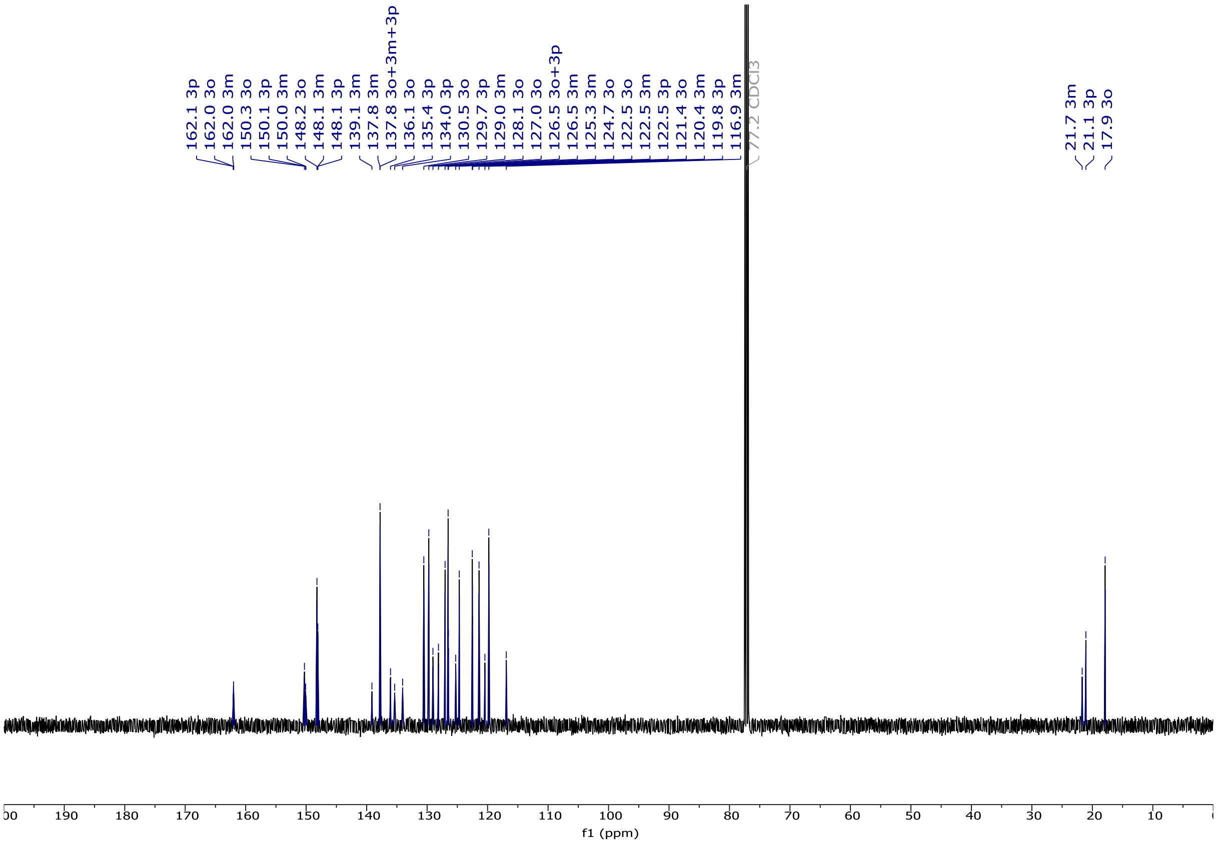

N-(2-Methoxyphenyl)picolinamide (6o)

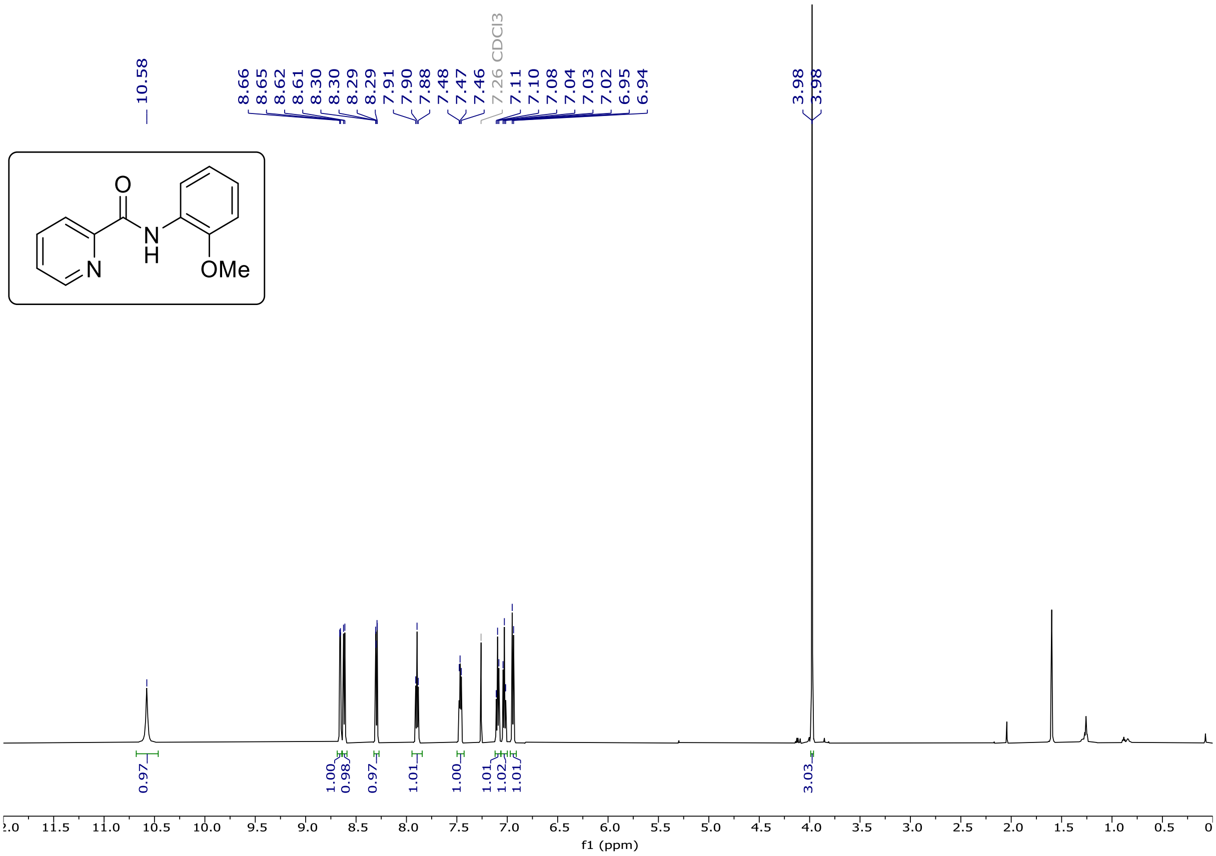

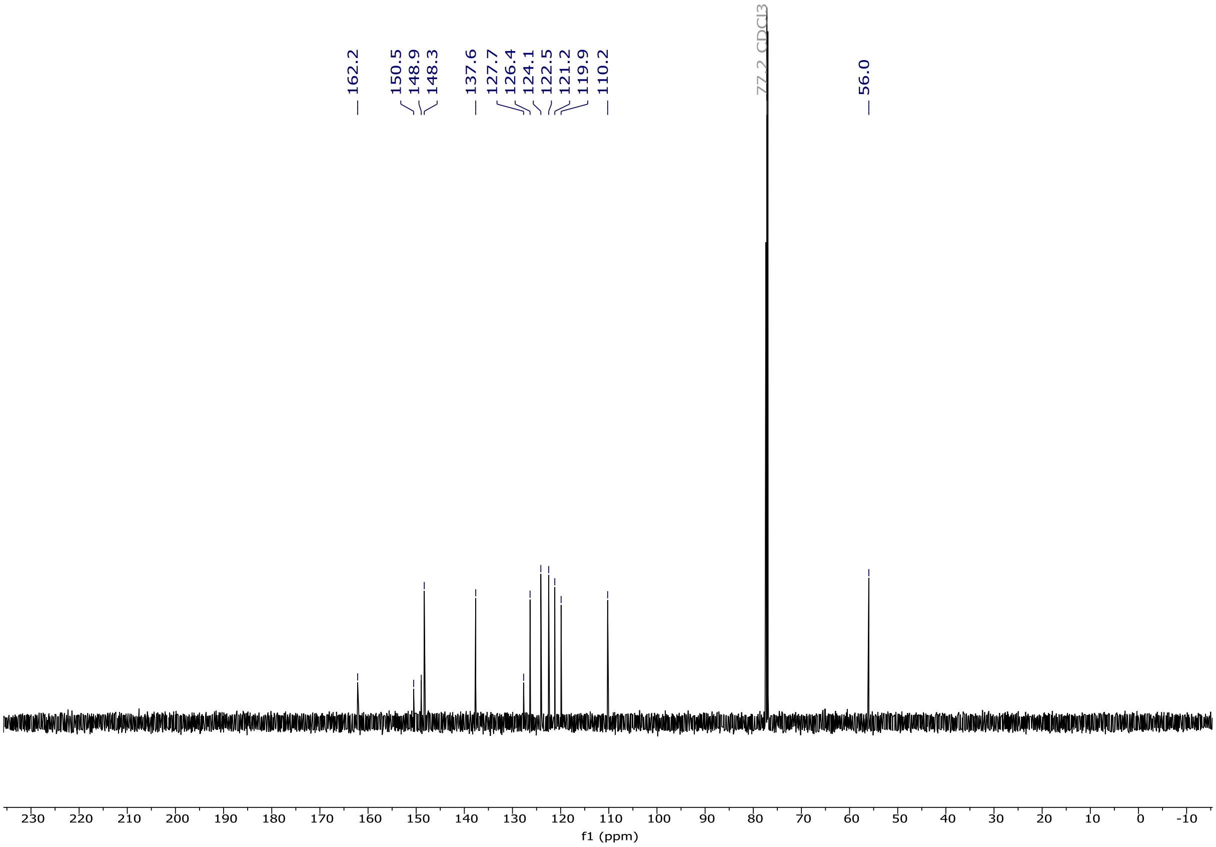

N-(4-Methoxyphenyl)picolinamide (6p)

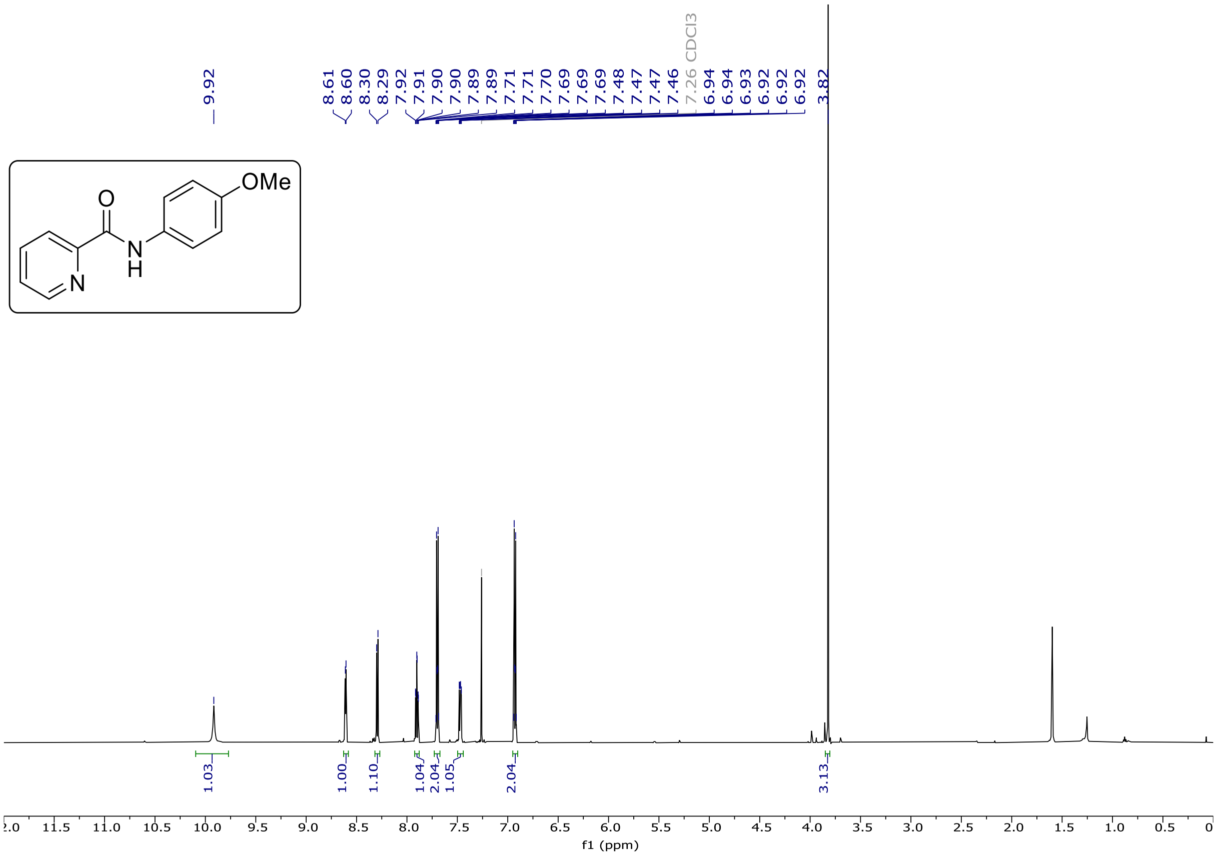

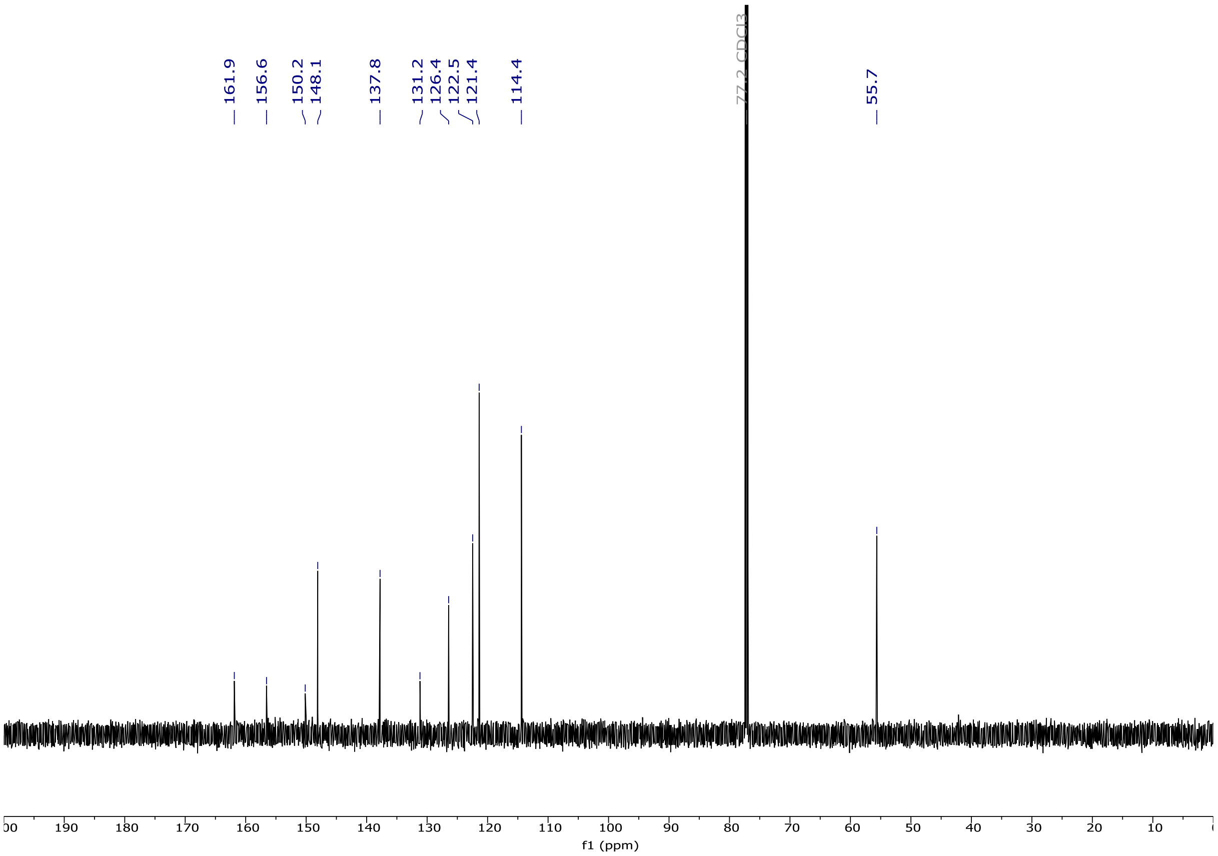

***N*-(2-Chloro)picolinamide (7o)**

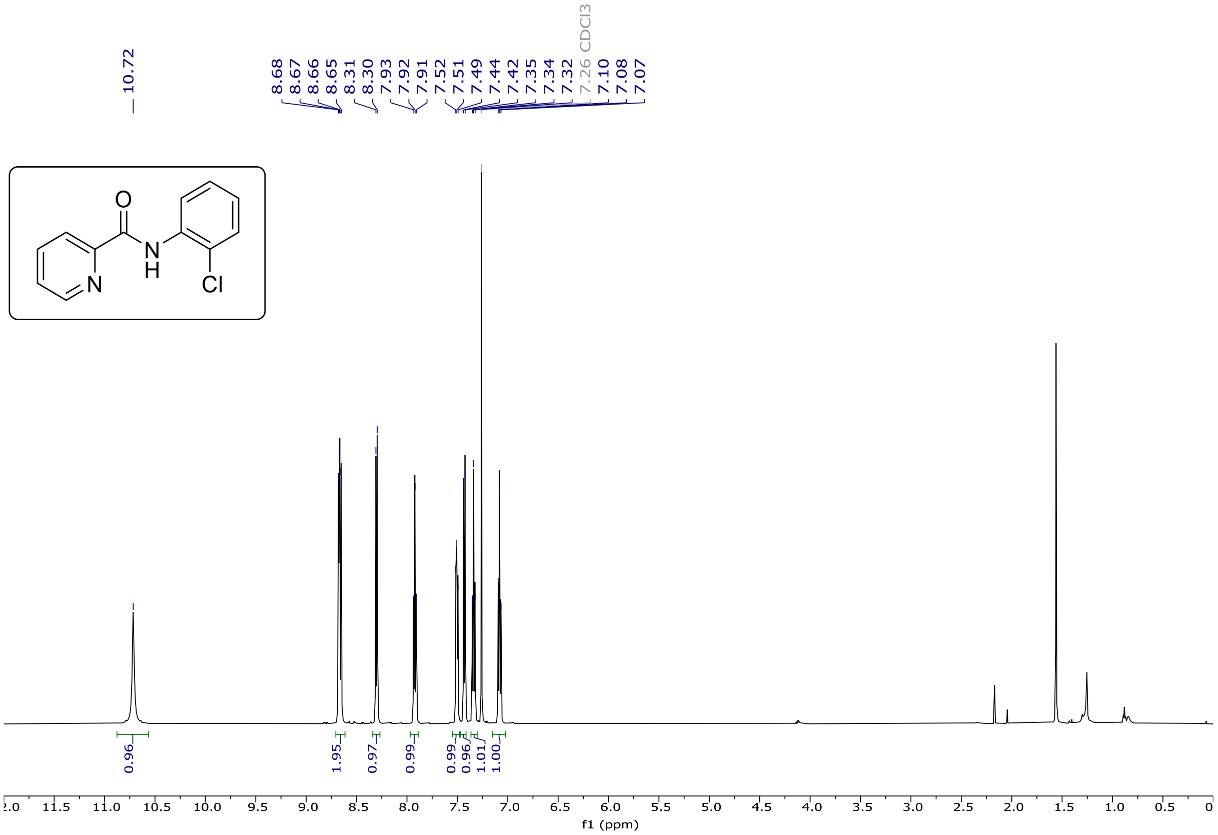

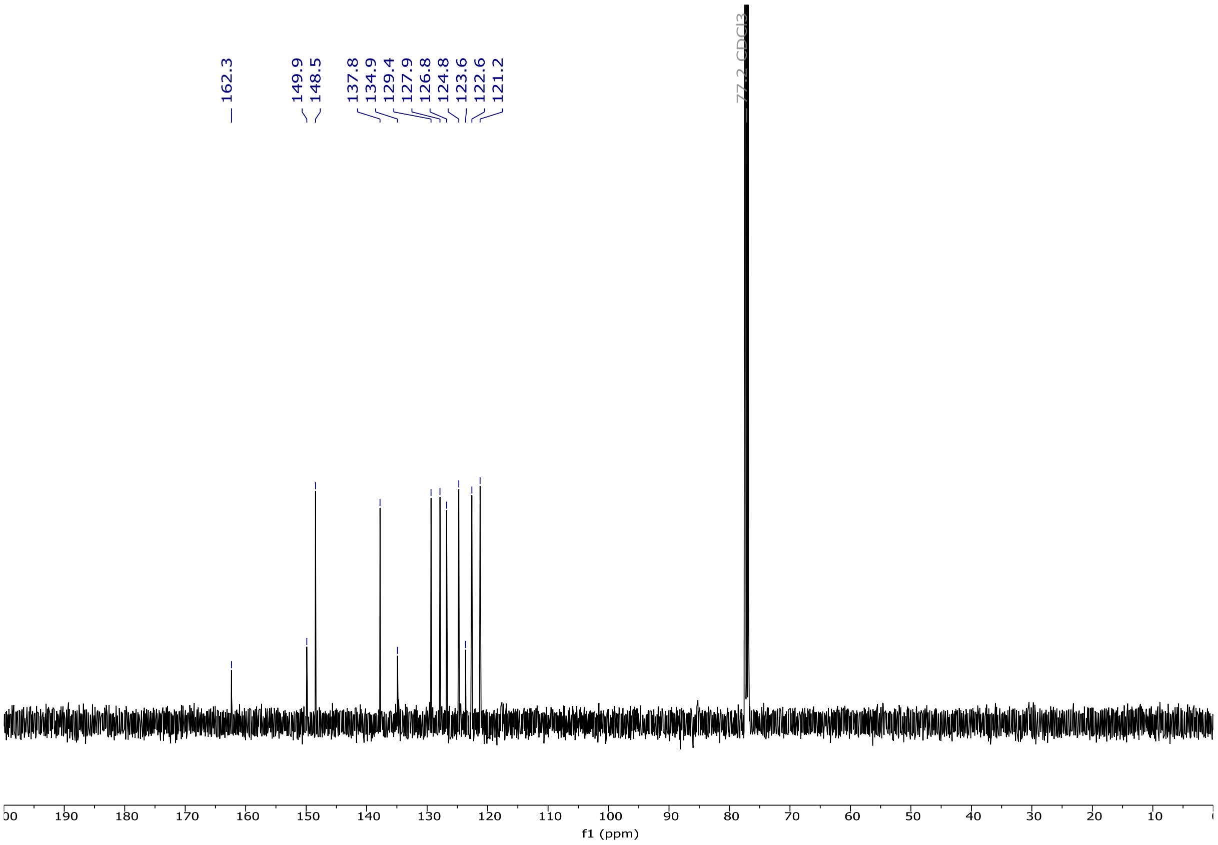

Mixture of *N*-(3-chloro)picolinamide (7m) and *N*-(4-chloro)picolinamide (7p)

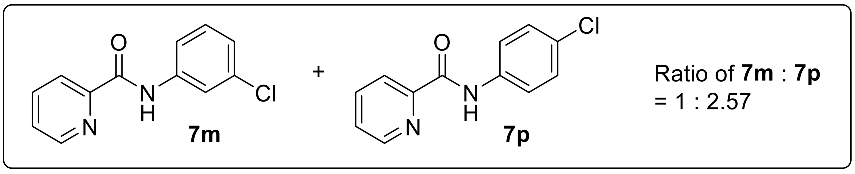

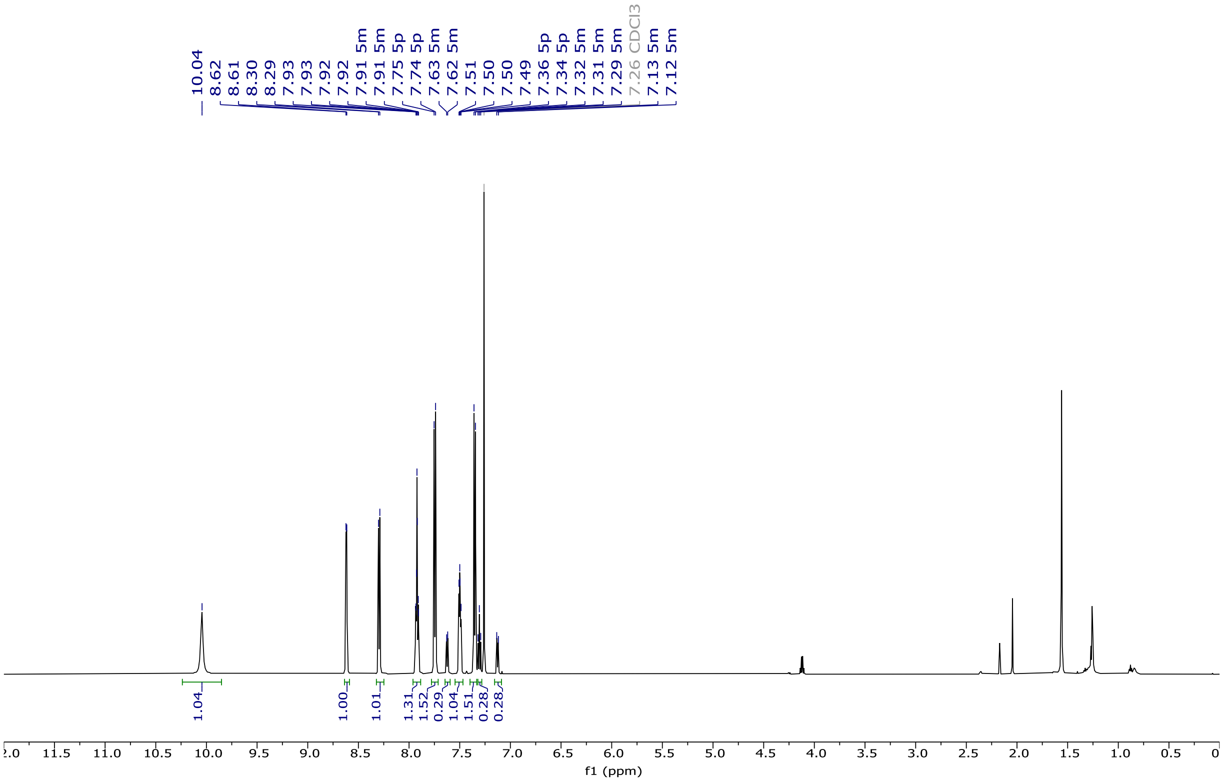

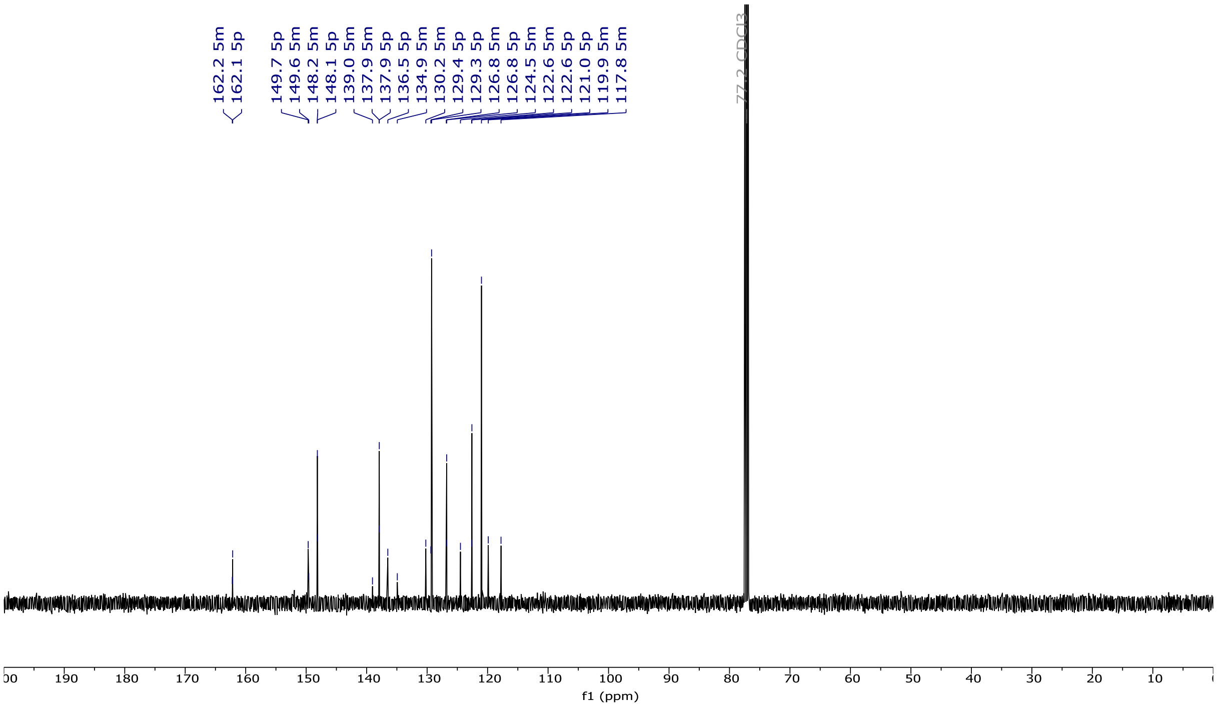

***N*-(2-Hydroxyphenyl)picolinamide (8o)**

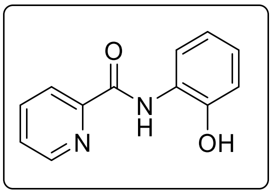

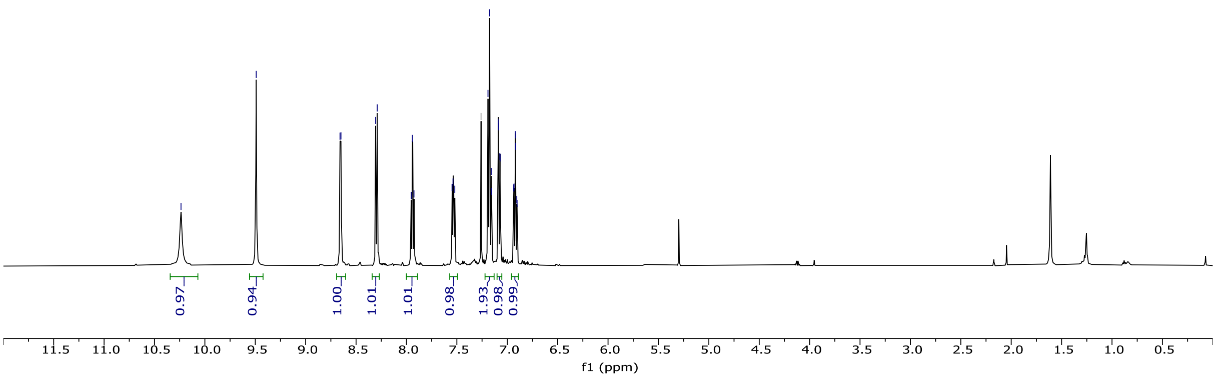

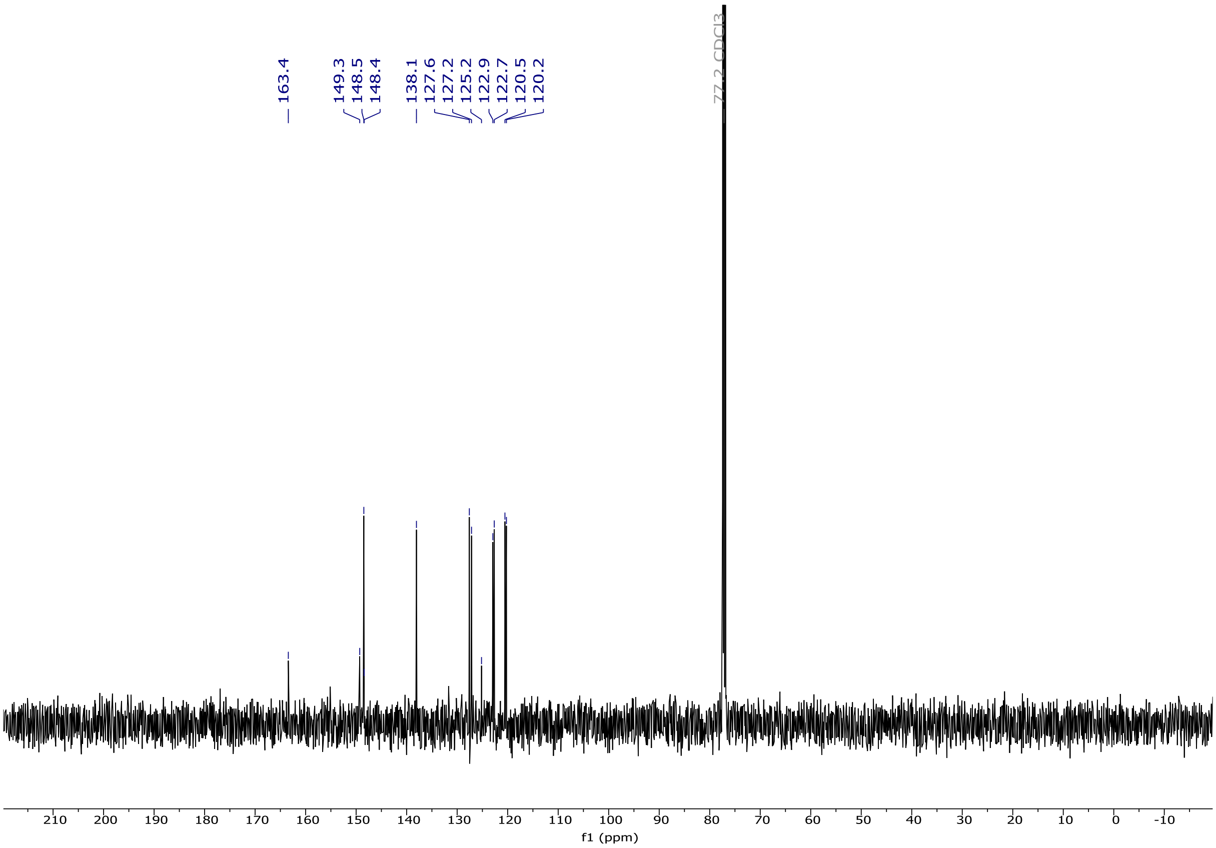

N-(4-Hydroxyphenyl)picolinamide (8p)

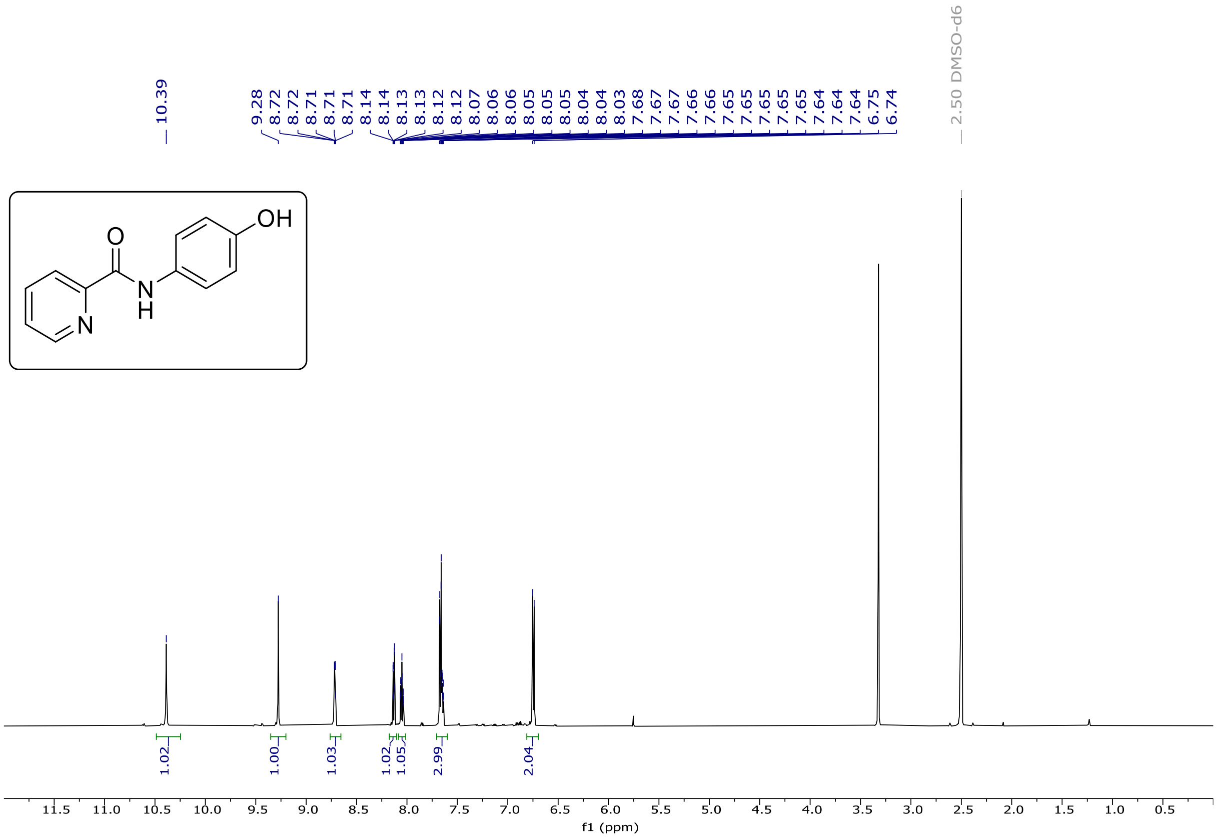

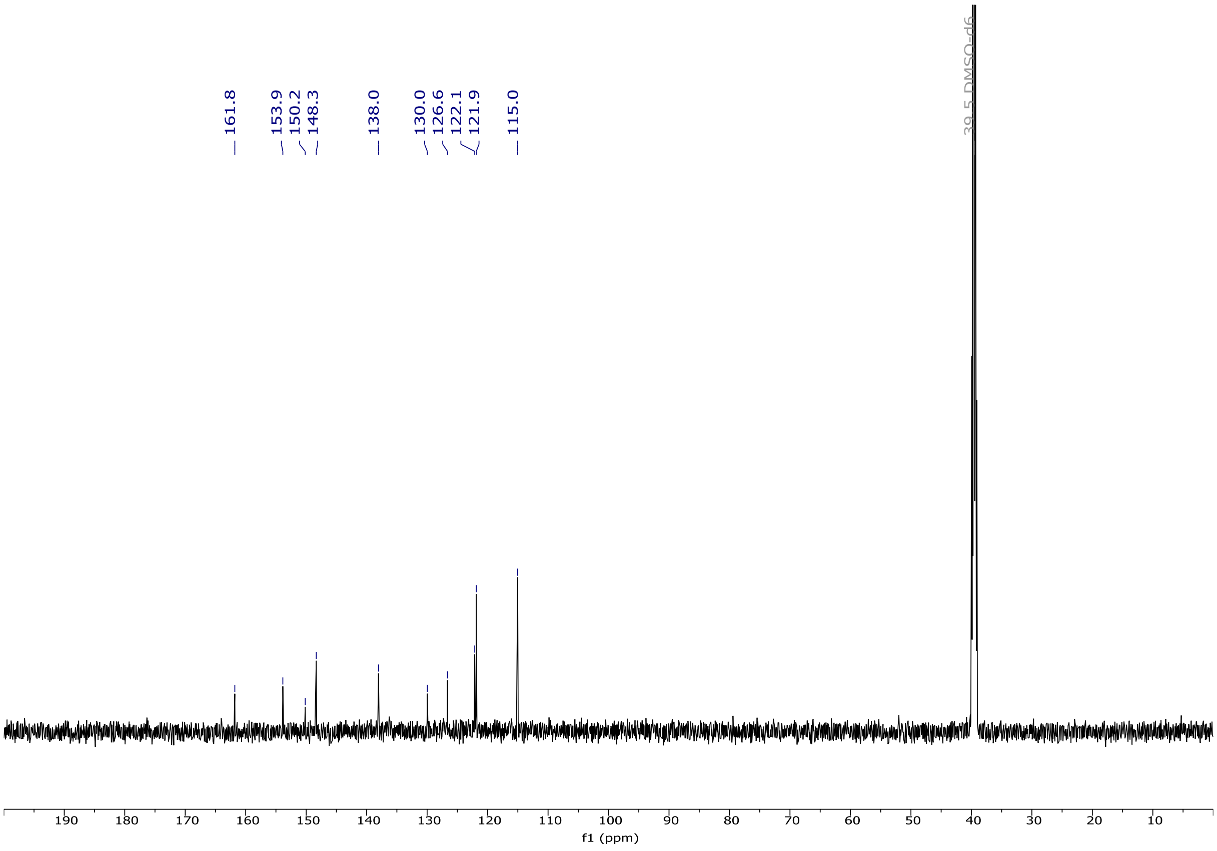

N-(2,5-Dimethylphenyl)picolinamide (9)

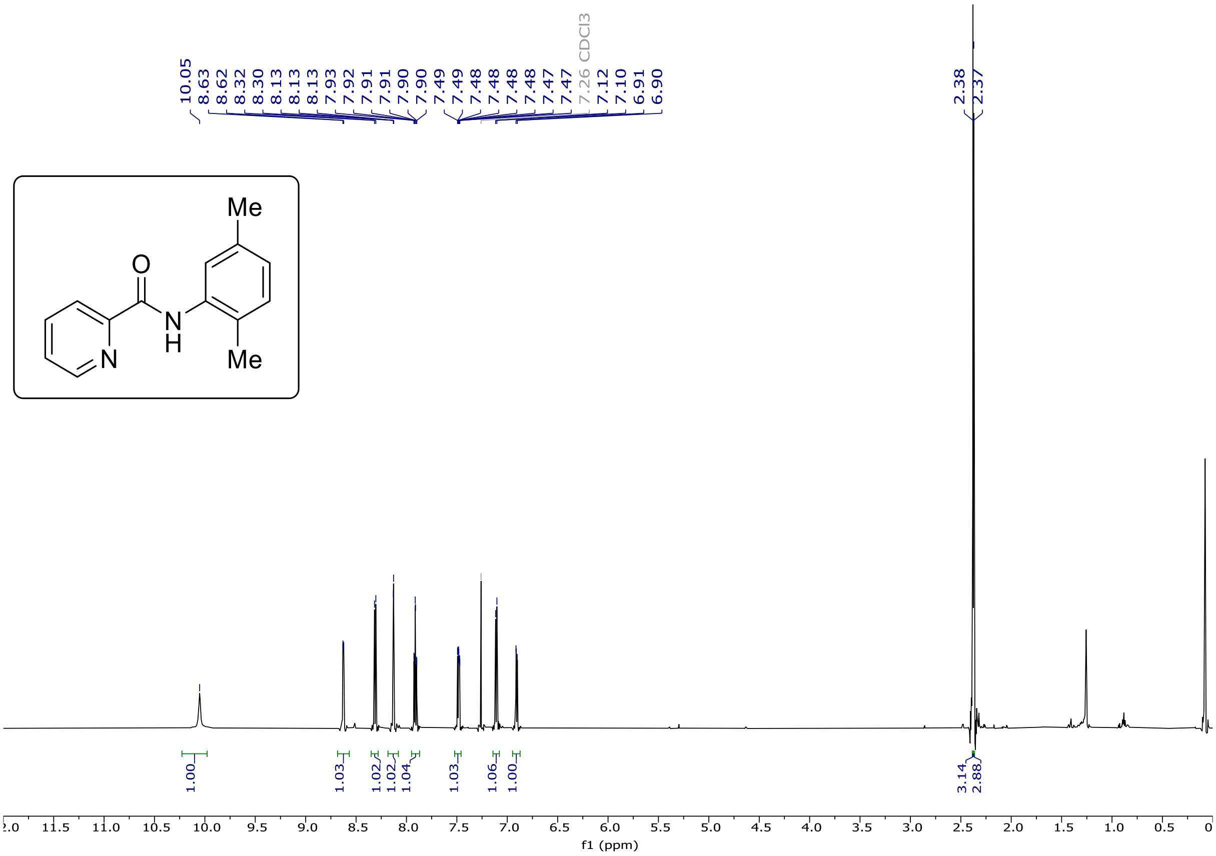

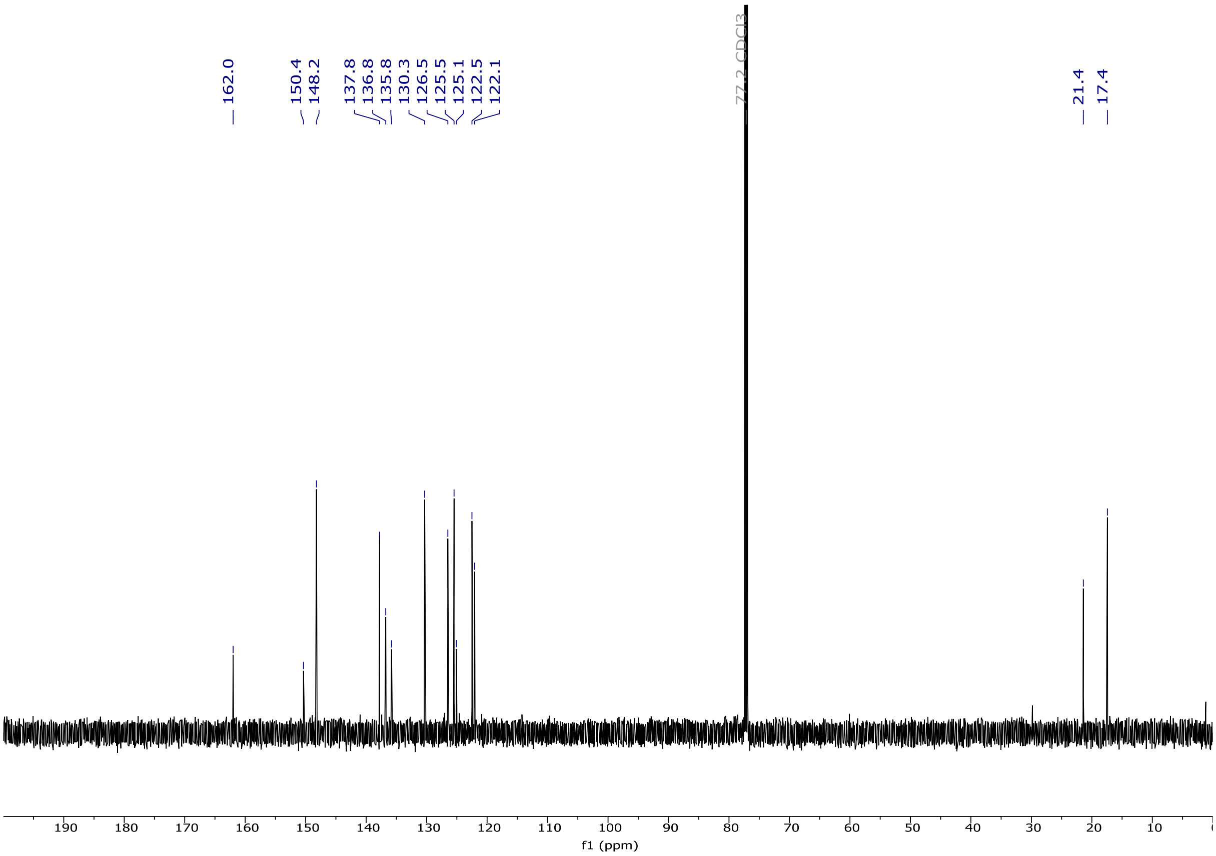

Mixture of *N*-(2,3-dimethylphenyl)picolinamide (10a) and *N*-(3,4-dimethylphenyl)picolinamide (10b)

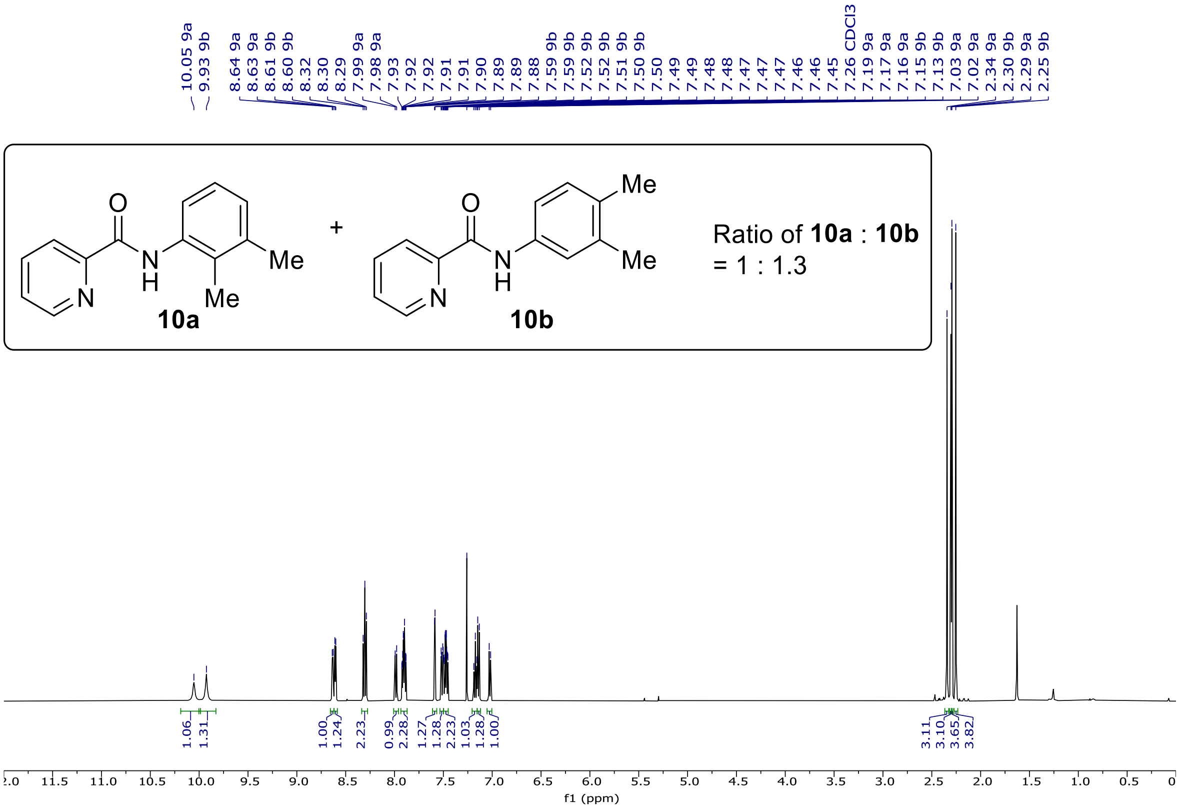

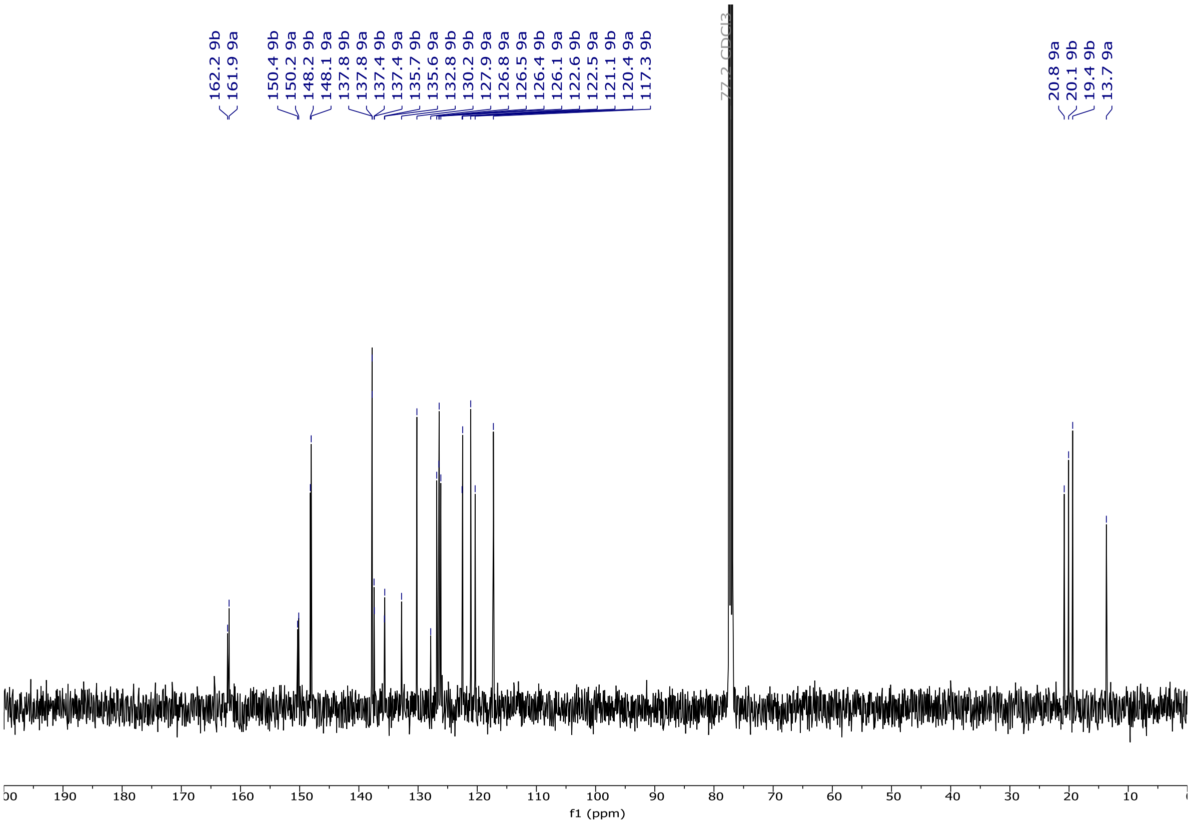

N-(5-Fluoro-2-methoxyphenyl)picolinamide (11a)

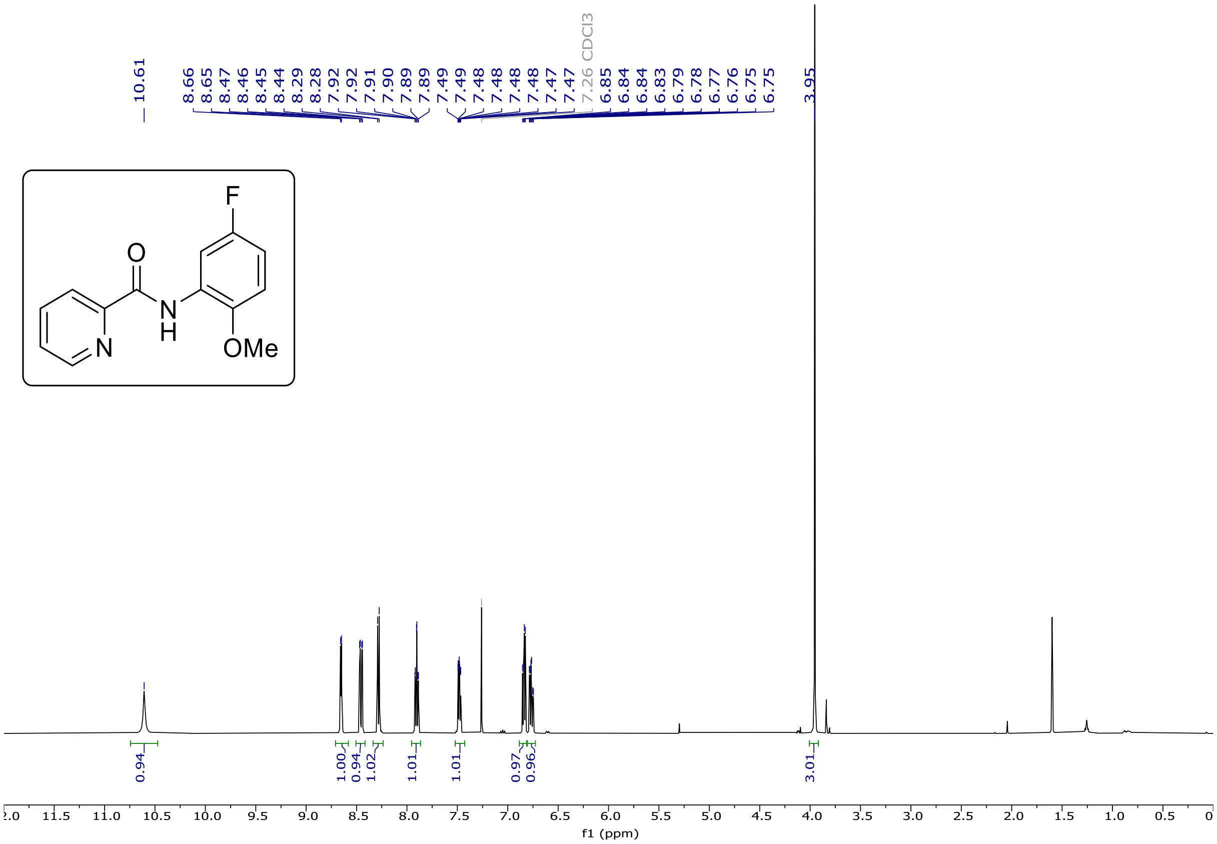

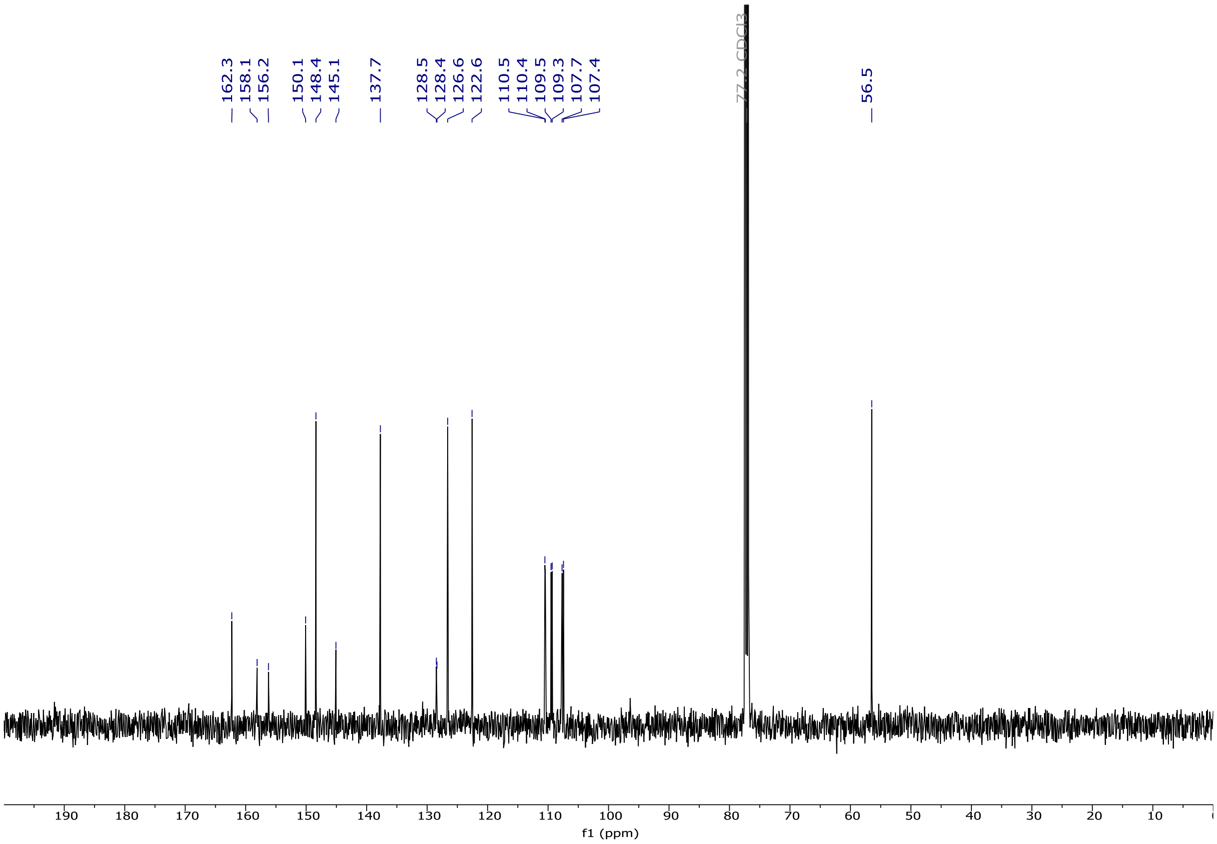

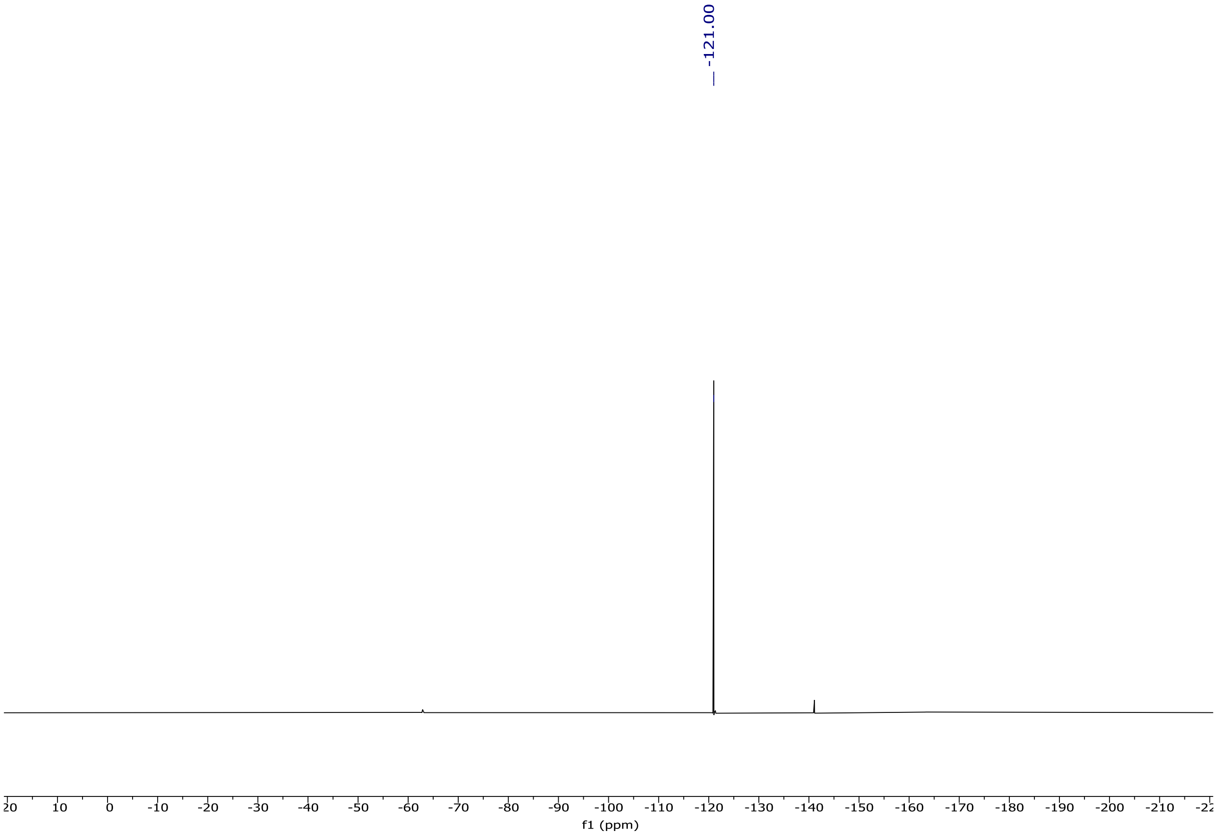

-121.00
20
10
0
-10
-20
-30
-40
-50
-60
-70
-80
-90
-100
-110
-120
-130
-140
-150
-160
-170
-180
-190
-200
-210
f1 (ppm)

_N_-(2-Fluoro-5-methoxyphenyl)picolinamide (11b)

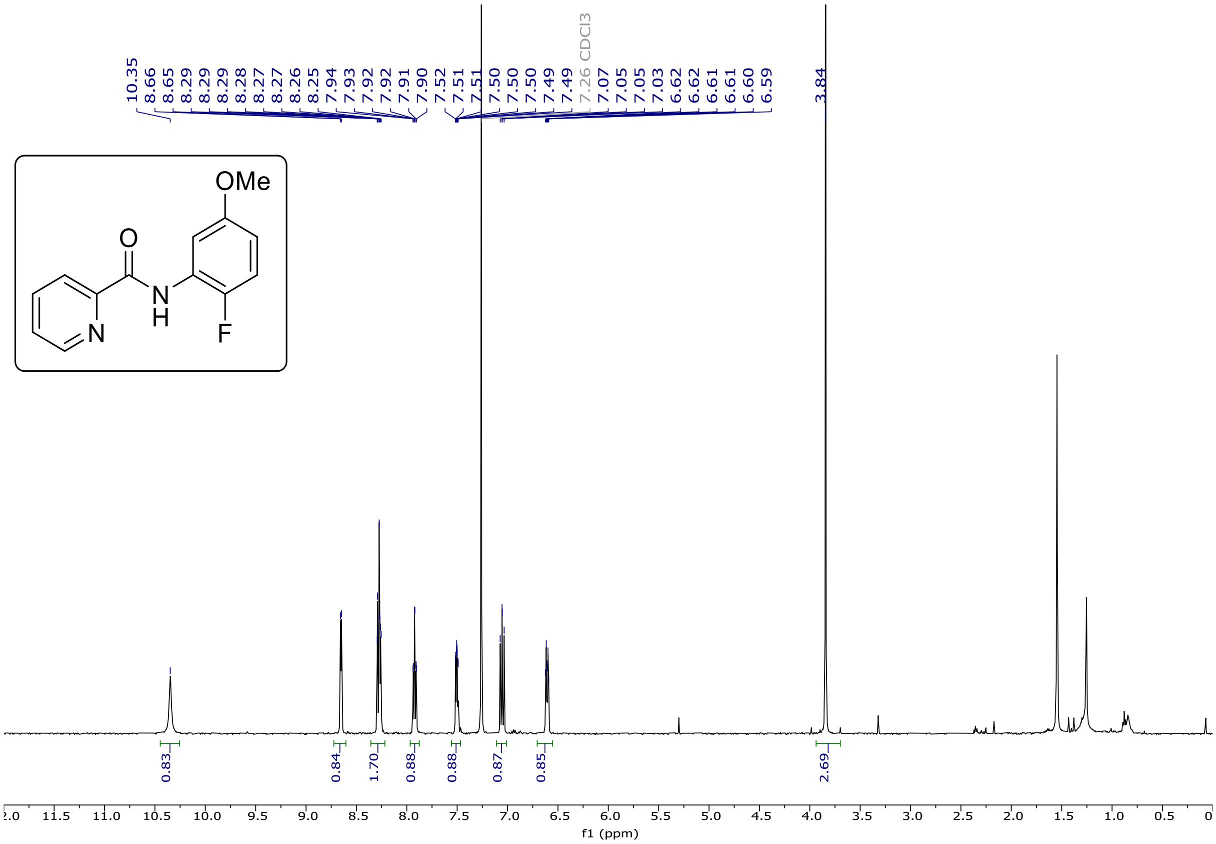

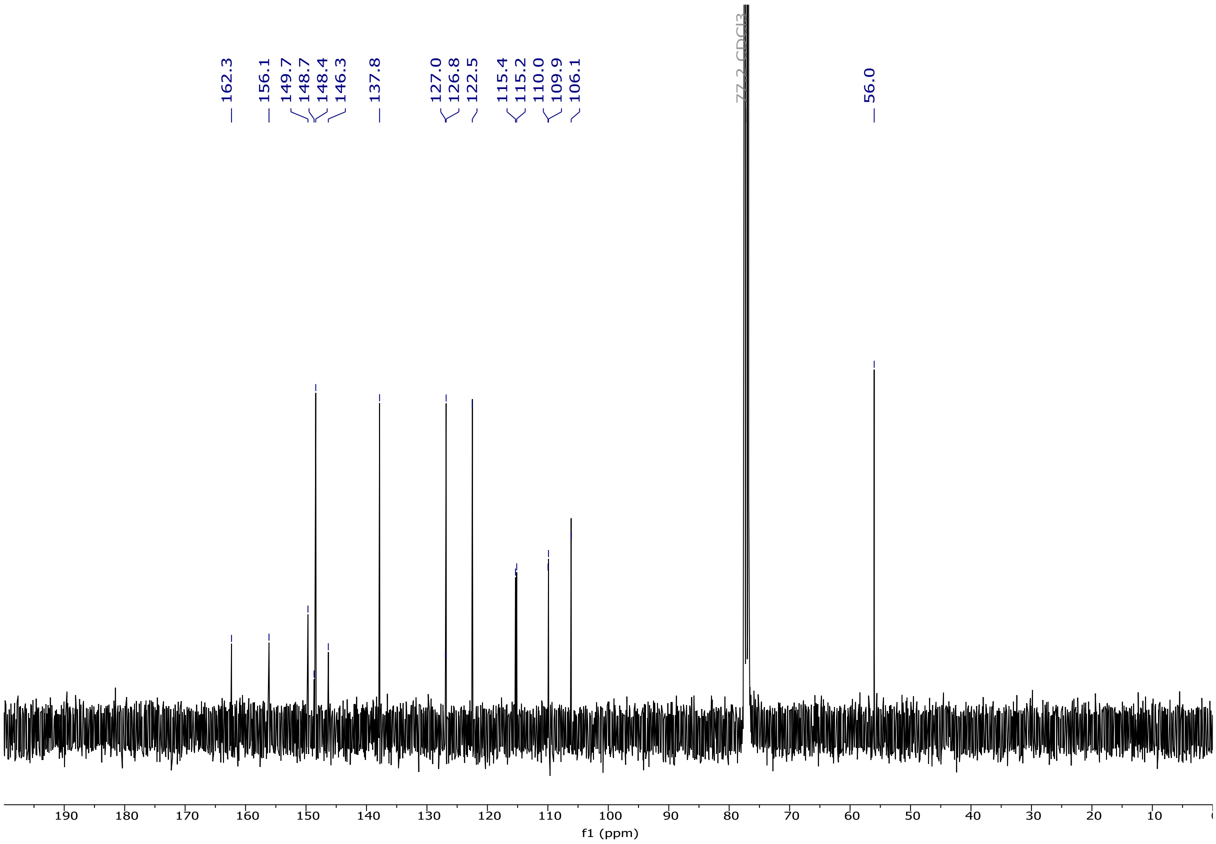

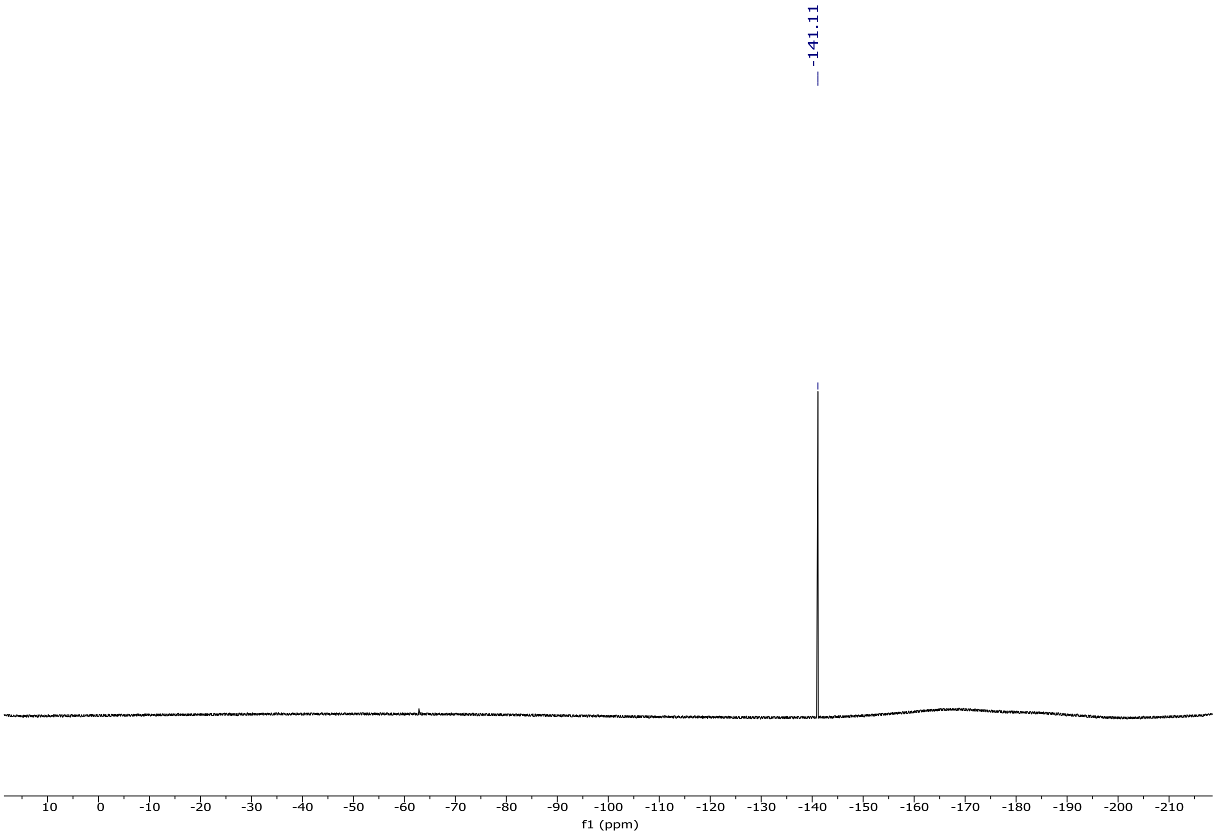
-141.11
10 0 -10 -20 -30 -40 -50 -60 -70 -80 -90 -100 -110 -120 -130 -140 -150 -160 -170 -180 -190 -200 -210
f1 (ppm)

***N*-(2,4,6-Trimethoxyphenyl)picolinamide (12)**

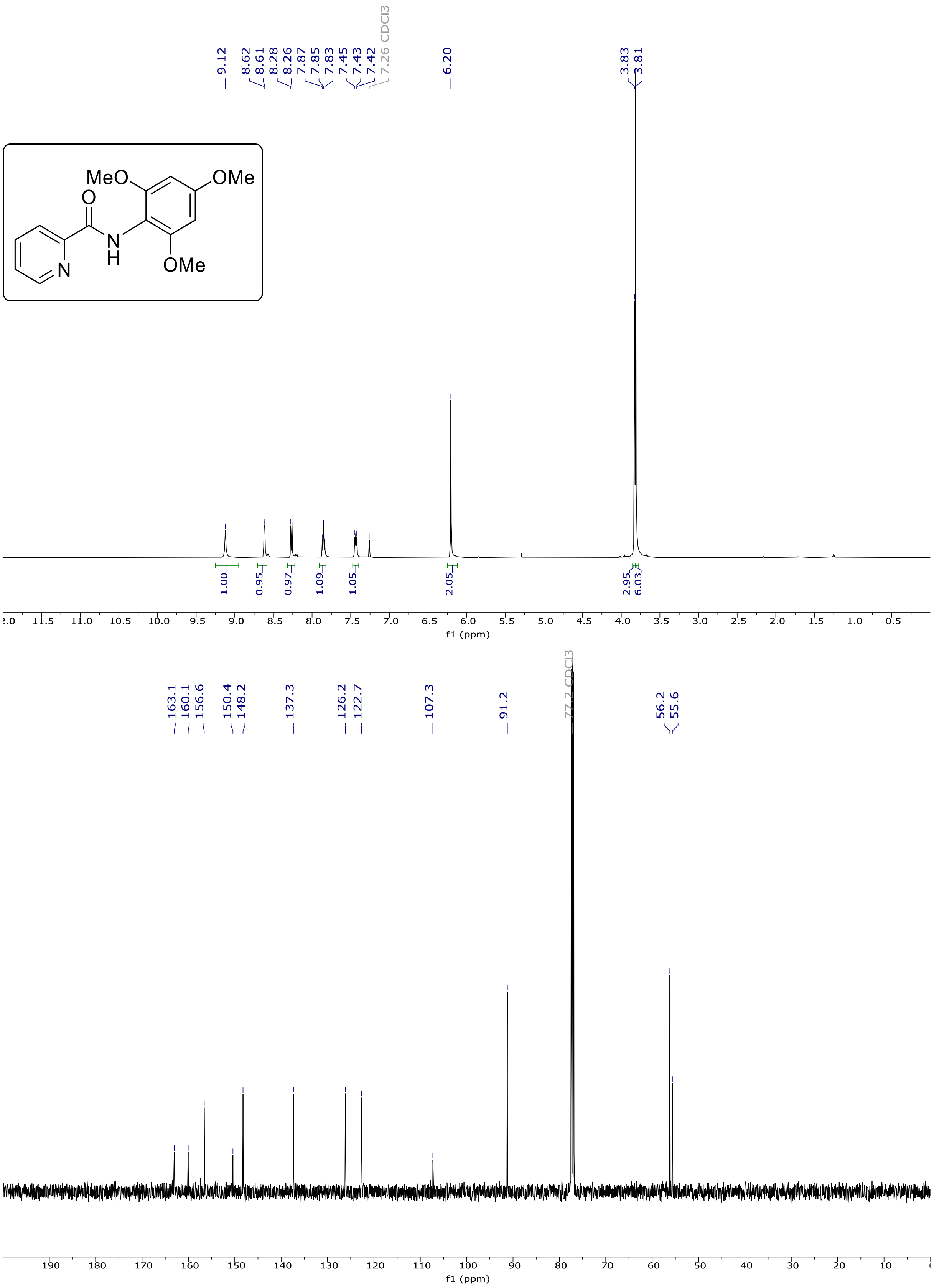

N-Mesitylpicolinamide (13)

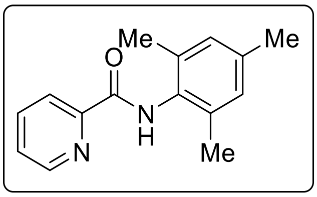

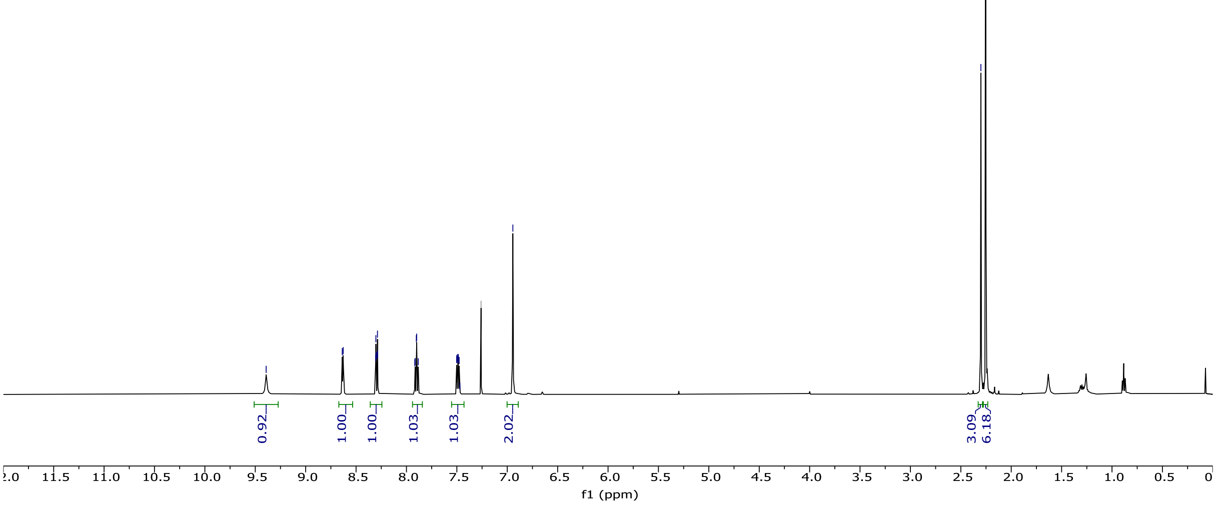

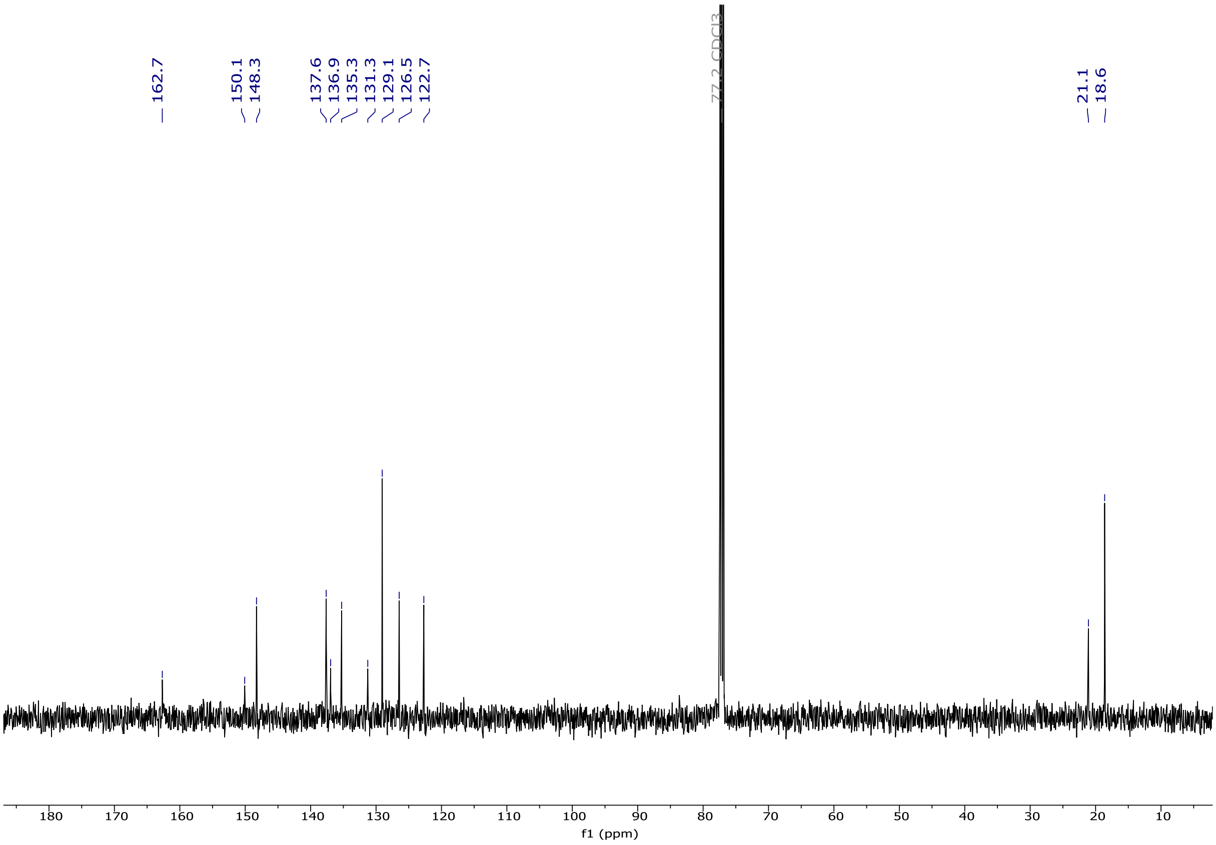

N-Cyclopentylpicolinamide (14)

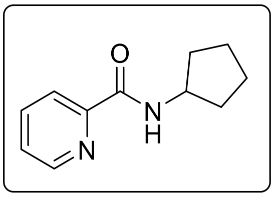

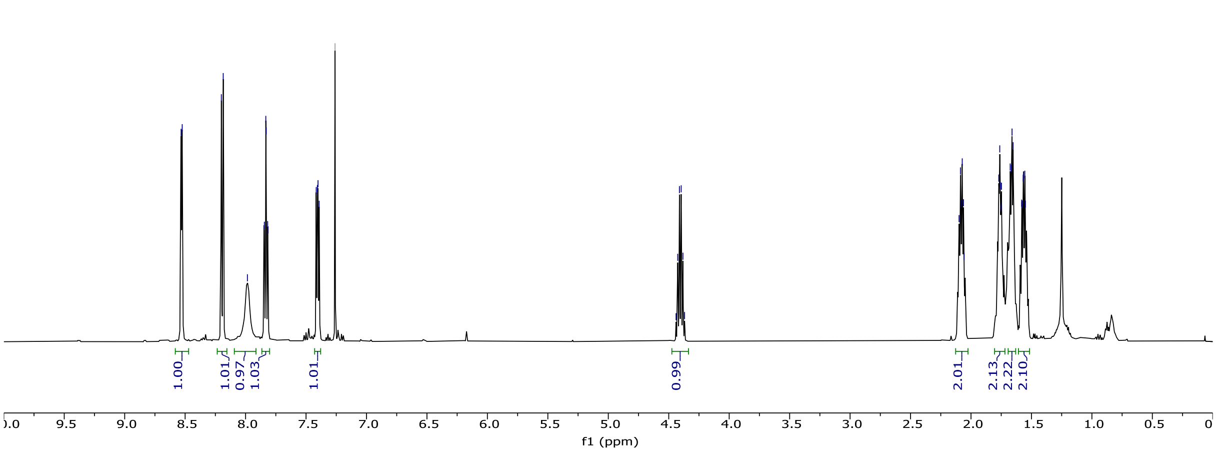

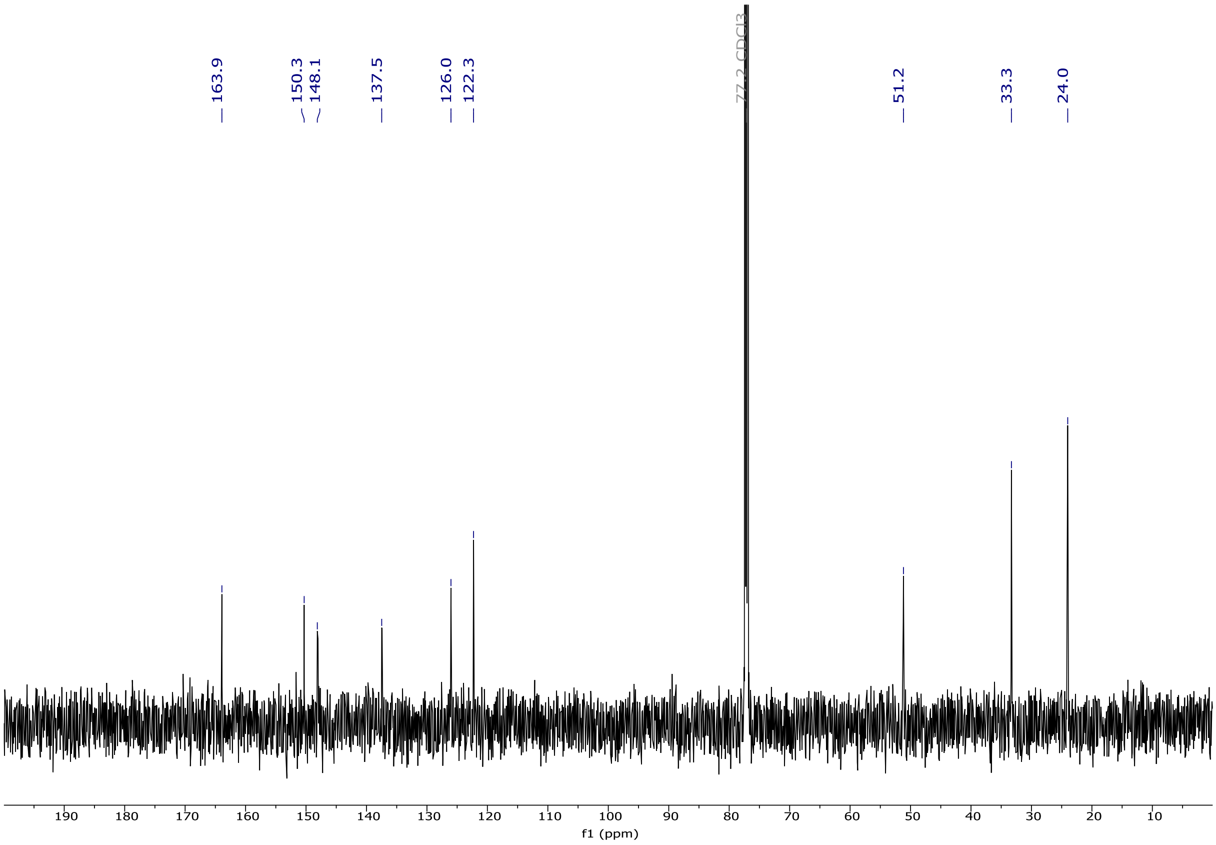

N-(Tetrahydrofuran-2-yl)picolinamide (15)

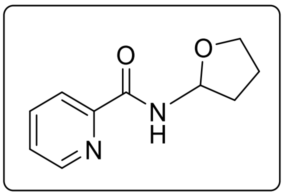

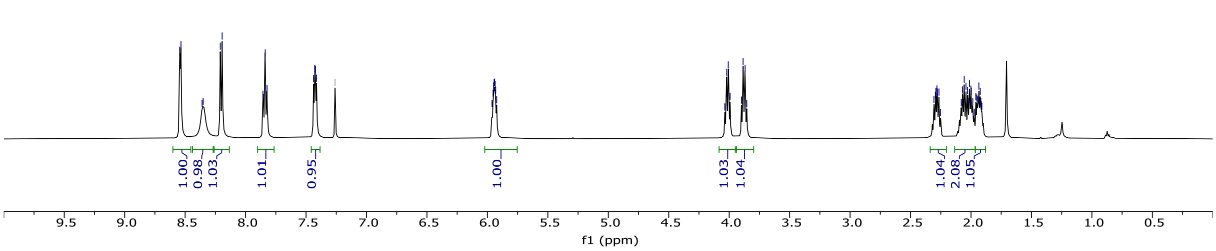

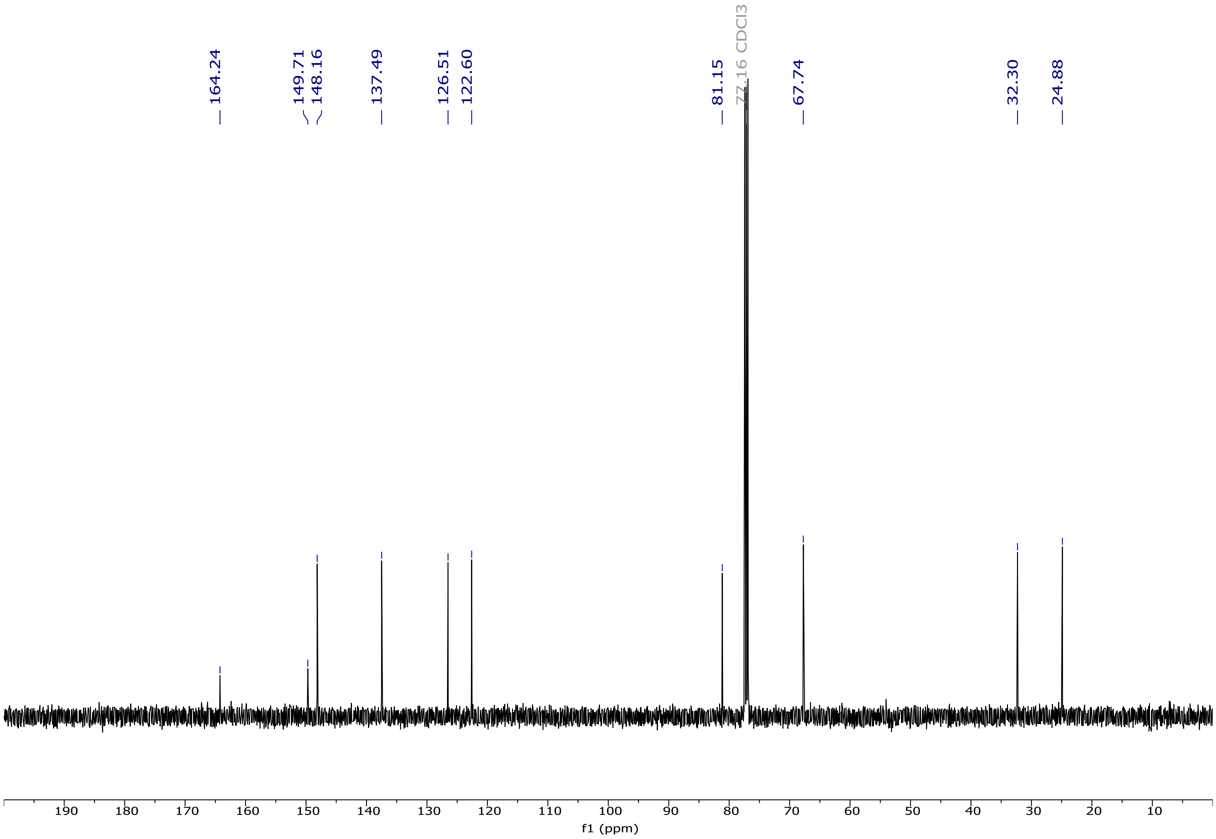

N-(1,4-Dioxan-2-yl)picolinamide (16)

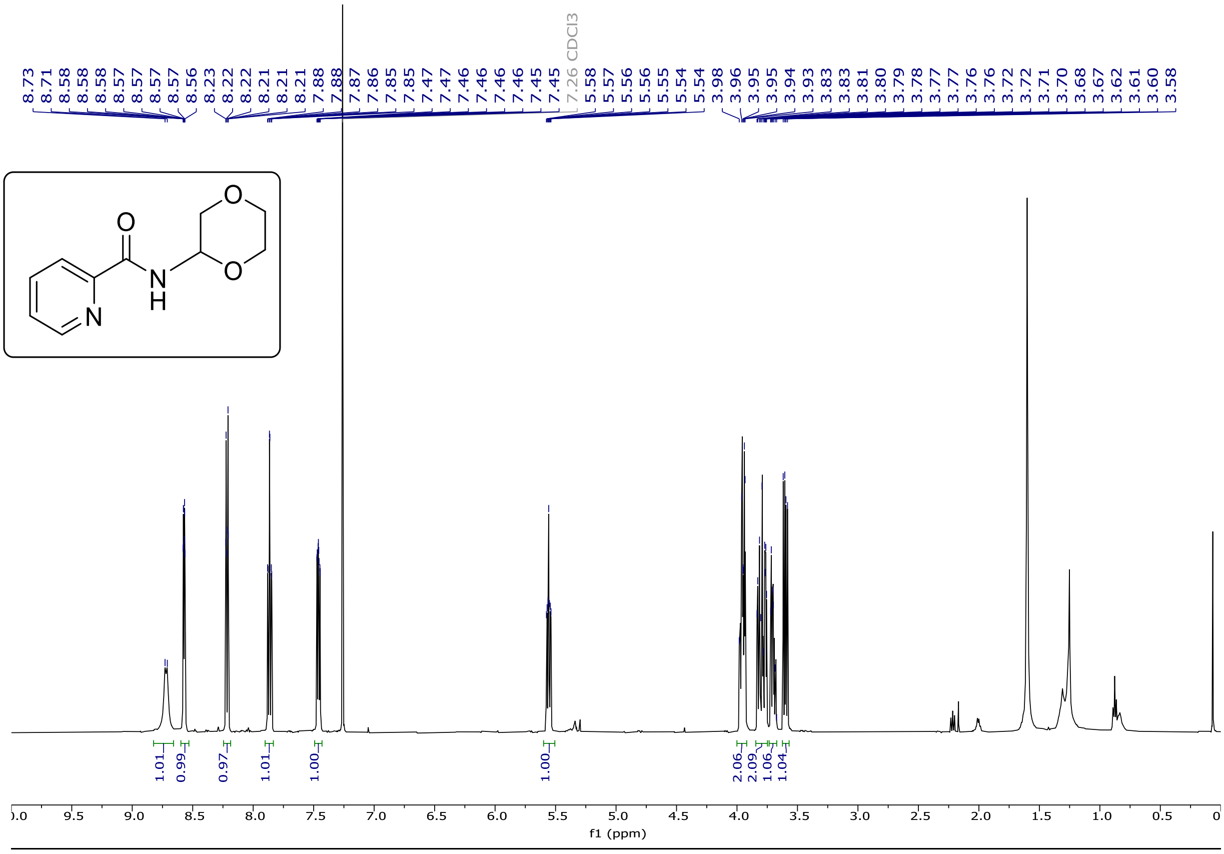

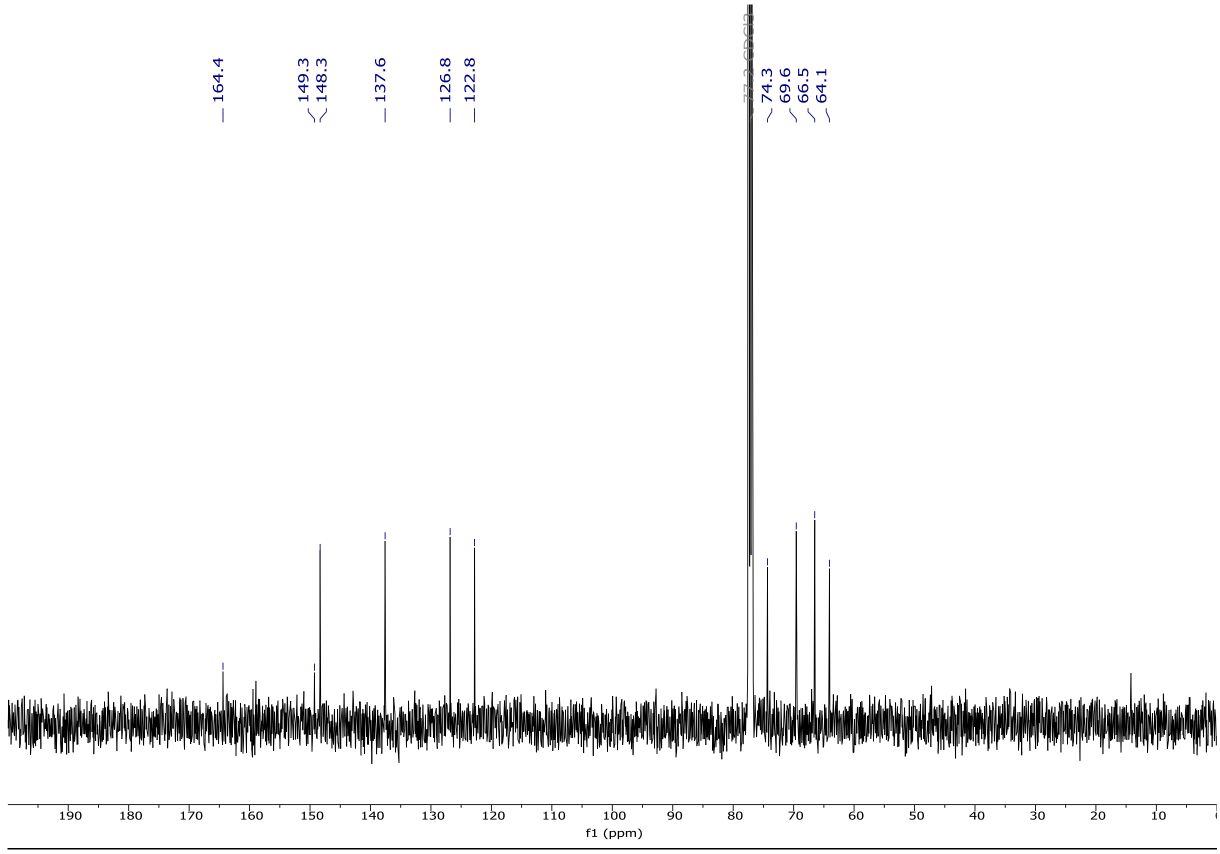

N-(1-Ethoxyethyl)picolinamide (17)

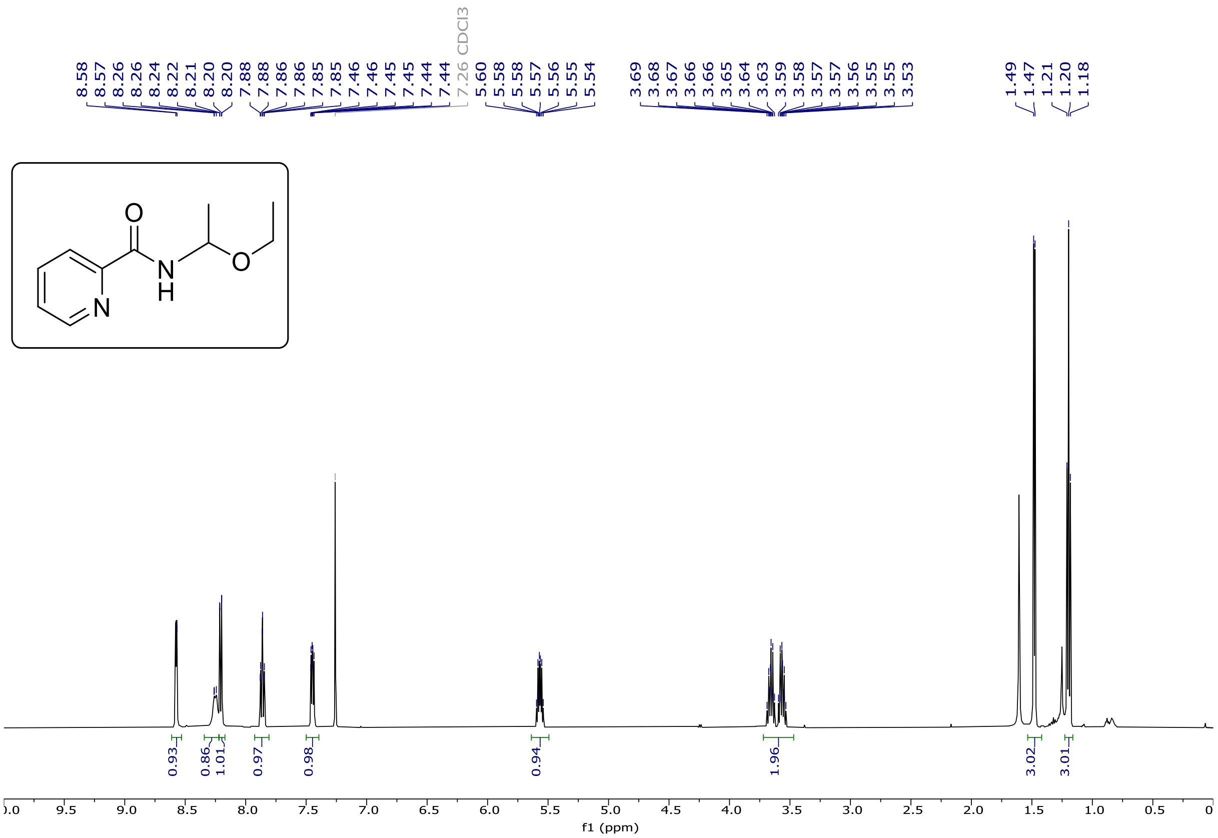

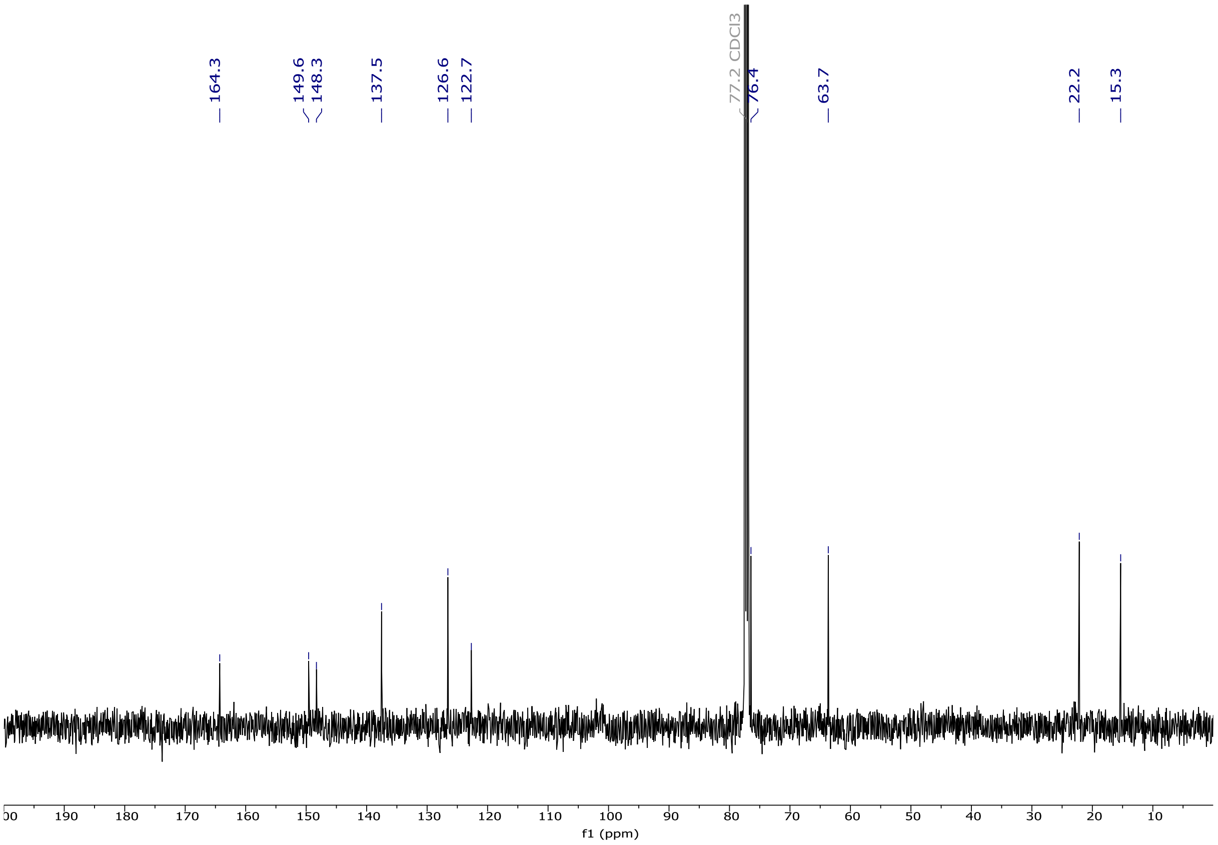

***N*-(*tert*-Butoxy)picolinamide (18)**

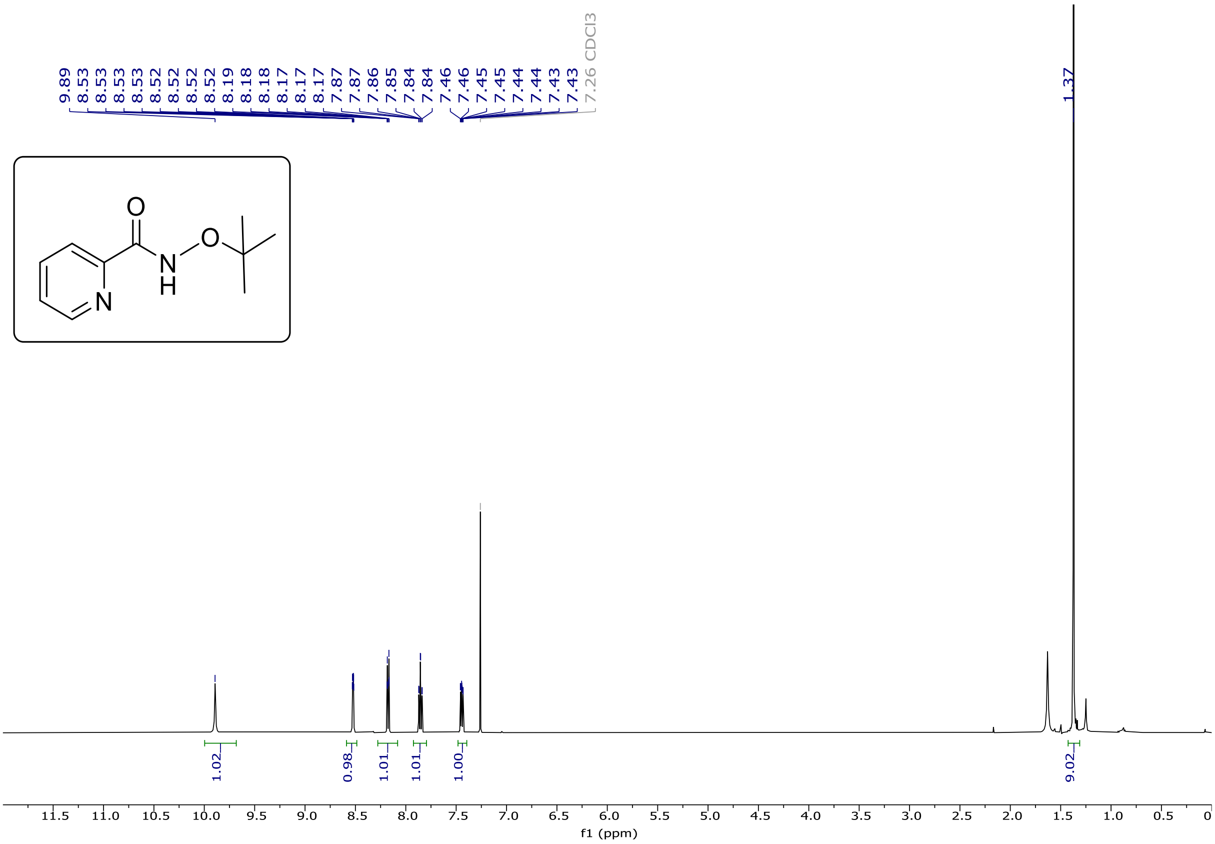

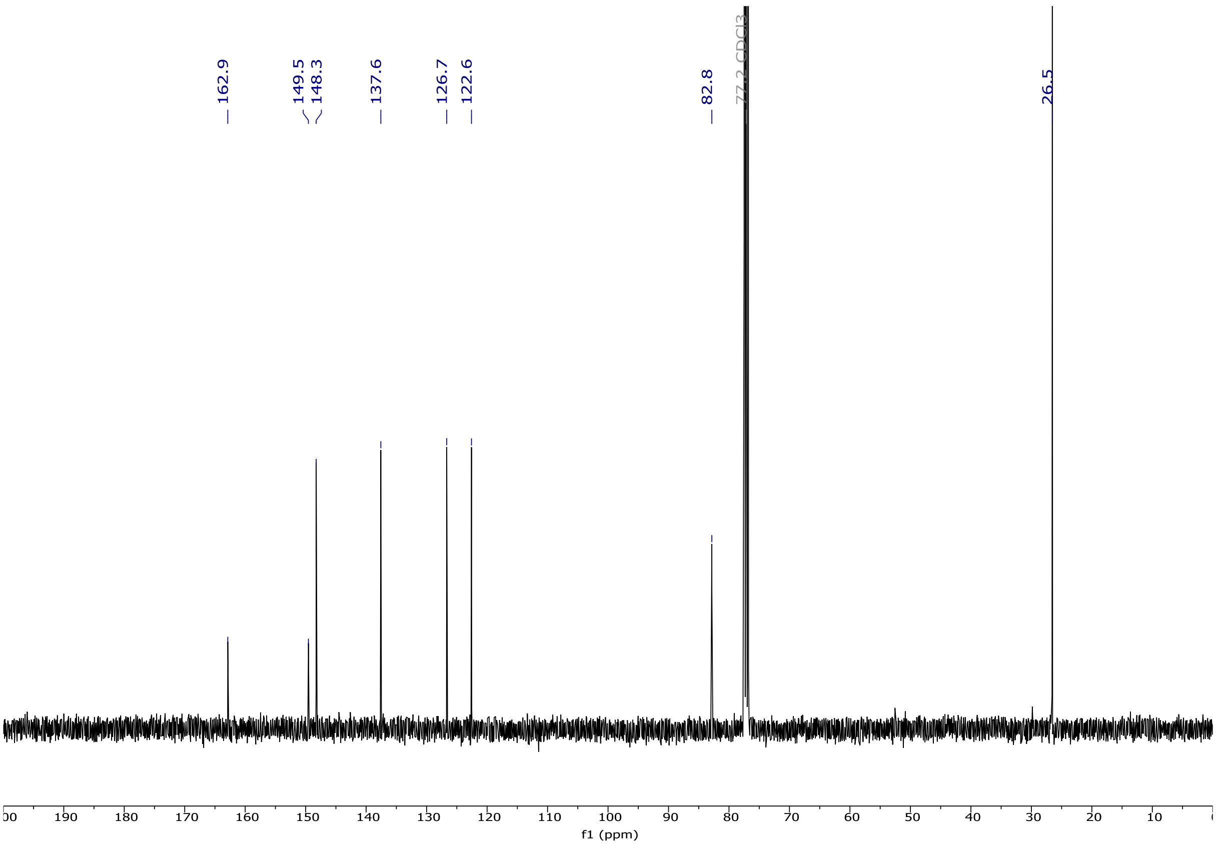

N'-Methyl-*N*'-phenylpicolinohydrazide (19)

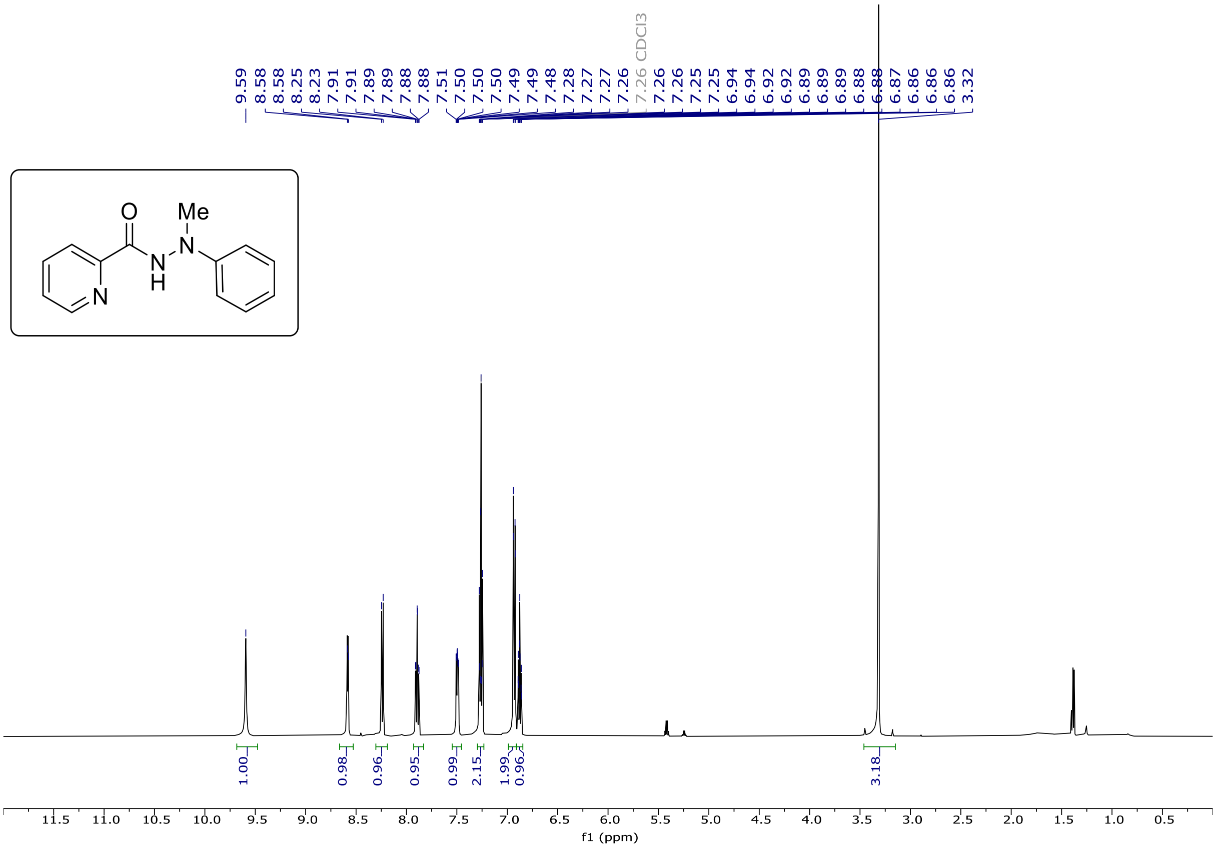

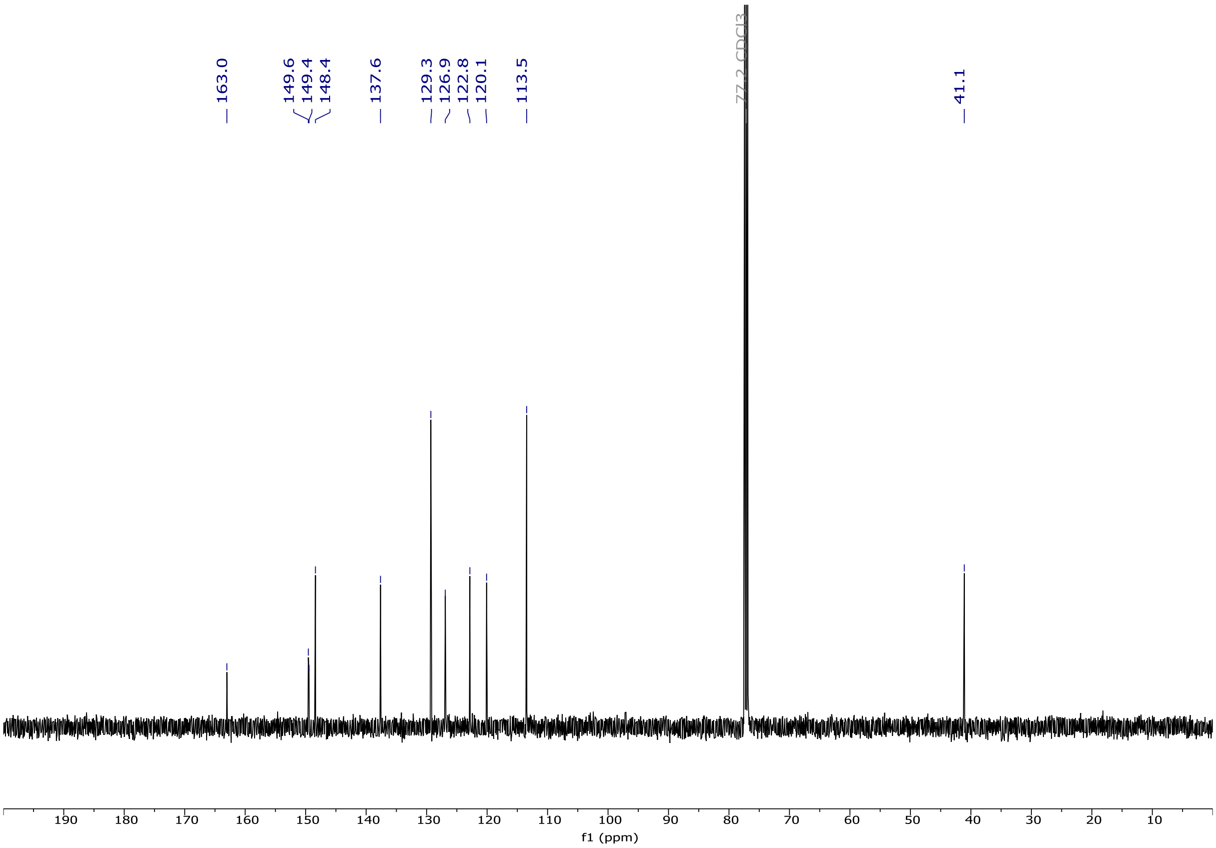

_N_-(3,4-Dihydroquinolin-1(2_H_)-yl)picolinamide (20)

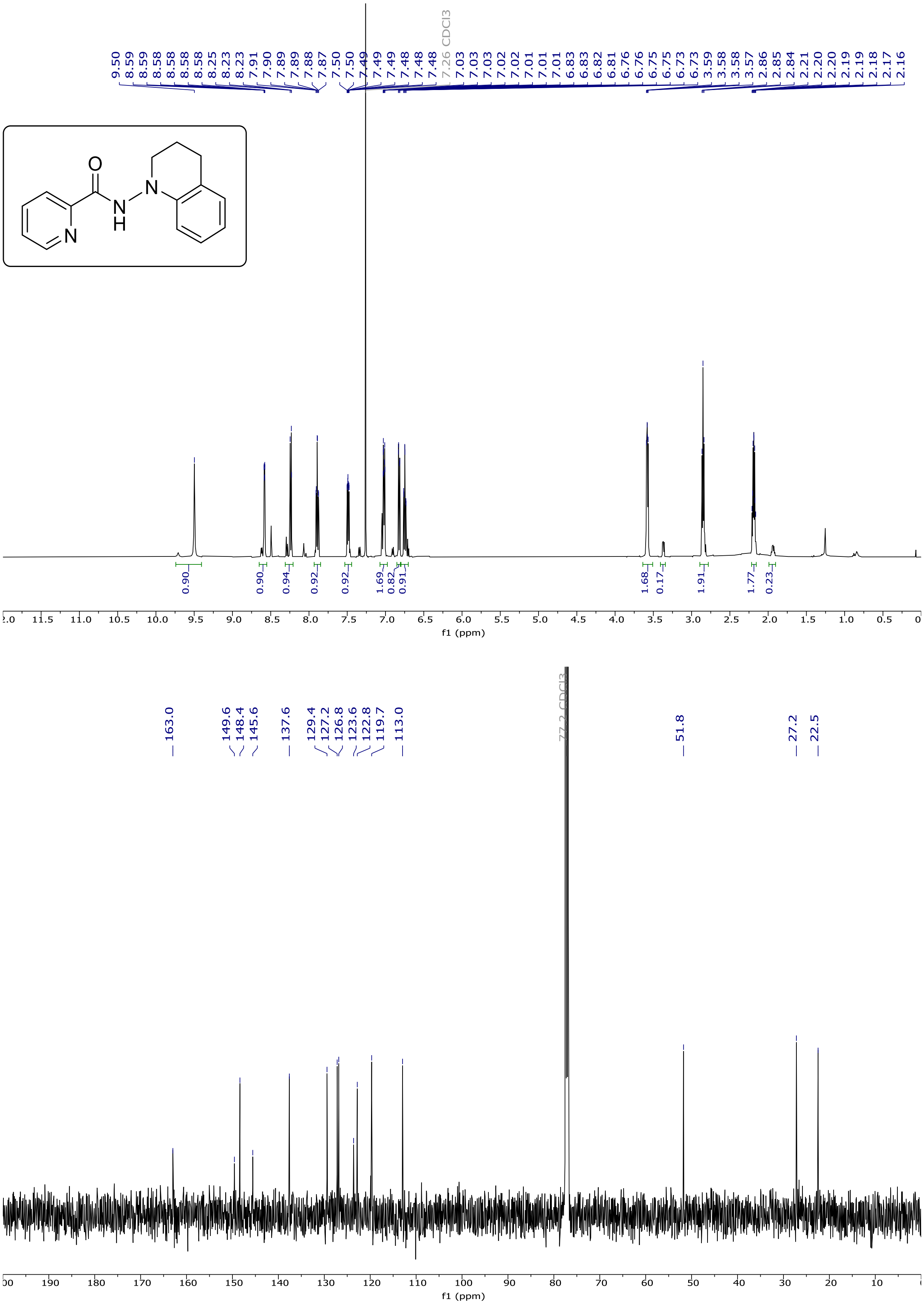

8. References

(1) Jung, H.; Kweon, J.; Suh, J.-M.; Lim, M. H.; Kim, D.; Chang, S. Mechanistic Snapshots of Rhodium-Catalyzed Acylnitrene Transfer Reactions. *Science* **2023**, *381*, 525–532.

(2) Steinlandt, P. S.; Zuo, W.; Harms, K.; Meggers, E. Bis-Cyclometalated Indazole Chiral-at-Rhodium Catalyst for Asymmetric Photoredox Cyanoalkylations. *Chem. -Eur. J.* **2019**, *25*, 15333–15340.

(3) Li, Q.; Zhang, S.-Y.; He, G.; Ai, Z.; Nack, W. A.; Chen, G. Copper-Catalyzed Carboxamide-Directed Ortho Amination of Anilines with Alkylamines at Room Temperature. *Org. Lett.* **2014**, *16*, 1764–1767.

(4) Dick, B. L.; Cohen, S. M. Metal-Binding Isosteres as New Scaffolds for Metalloenzyme Inhibitors. *Inorg. Chem.* **2018**, *57*, 9538–9543.

(5) Le, C. C.; Wismer, M. K.; Shi, Z.-C.; Zhang, R.; Conway, D. V.; Li, G.; Vachal, P.; Davies, I. W.; MacMillan, D. W. C. A General Small-Scale Reactor To Enable Standardization and Acceleration of Photocatalytic Reactions. *ACS Cent. Sci.* **2017**, *3*, 647–653.

(6) Nadres, E. T.; Santos, G. I. F.; Shabashov, D.; Daugulis, O. Scope and Limitations of Auxiliary-Assisted, Palladium-Catalyzed Arylation and Alkylation of Sp^2 and Sp^3 C–H Bonds. *J. Org. Chem.* **2013**, *78*, 9689–9714.

(7) Wang, W.-H.; Shao, W.-Y.; Sang, J.-Y.; Li, X.; Yu, X.; Yamamoto, Y.; Bao, M. N,N-Dialkylation of Acyl Hydrazides with Alcohols Catalyzed by Amidato Iridium Complexes via Borrowing Hydrogen. *Organometallics* **2023**, *42*, 2623–2631.

(8) Wang, Y.; Liu, H.; Zhang, X.; Zhang, Z.; Huang, D. Experimental and Mechanistic Insights into Copper(Ii)-Dioxygen Catalyzed Oxidative N-Dealkylation of N-(2-Pyridylmethyl)Phenylamine and Its Derivatives. *Org. Biomol. Chem.* **2017**, *15*, 9164–9168.

(9) Jaiswal, A.; Preeti; Singh, K. N. A Convenient Synthesis of N-(Hetero)Arylamides by the Oxidative Coupling of Methylheteroarenes with Amines. *Org. Biomol. Chem.* **2022**, *20*, 6915–6922.

(10) Cheng, T.-J.; Wang, X.; Xu, H.; Dai, H.-X. Copper-Mediated Ortho CH Primary Amination of Anilines. *Tetrahedron Lett.* **2021**, *73*, 153099.

(11) Martínez, Á. M.; Rodríguez, N.; Arrayás, R. G.; Carretero, J. C. Copper-Catalyzed Ortho-C-H Amination of Protected Anilines with Secondary Amines. *Chem. Commun.* **2014**, *50*, 2801–2803.

(12) Gao, L.; Wang, Z.-F.; Wang, L.-W.; Tang, H.-T.; Mo, Z.-Y.; He, M.-X. Electrochemical Selenium-Catalyzed Para-Amination of N-Aryloxyamides: Access to Polysubstituted Aminophenols. *Org. Biomol. Chem.* **2023**, *21*, 7895–7899.

(13) Ju, L.; Yao, J.; Wu, Z.; Liu, Z.; Zhang, Y. Palladium-Catalyzed Oxidative Acetoxylation of Benzylic C-H Bond Using Bidentate Auxiliary. *J. Org. Chem.* **2013**, *78*, 10821–10831.

(14) van Rijt, S. H.; Hebden, A. J.; Amaresekera, T.; Deeth, R. J.; Clarkson, G. J.; Parsons, S.; McGowan, P. C.; Sadler, P. J. Amide Linkage Isomerism as an Activity Switch for Organometallic Osmium and Ruthenium Anticancer Complexes. *J. Med. Chem.* **2009**, *52*, 7753–7764.

(15) Sankar, R.; Babu, S. A. Construction of Tertiary Amides: Ni^{II}-Catalyzed *N*-Arylation of Secondary Acyclic Amides (2-Picolinamides) with Aryl Halides. *Asian J. Org. Chem.* **2017**, *6*, 269–273.

(16) Guo, J.-Y.; Zhong, C.-H.; He, Z.-Y.; Tian, S.-K. Benzyne-Promoted Curtius-Type Rearrangement of Acyl Hydrazides in the Presence of Nucleophiles. *Asian J. Org. Chem.* **2018**, *7*, 119–122.

(17) M. J. Frisch, G. W. Trucks, H. B. Schlegel, G. E. Scuseria, M. A. Robb, J. R. Cheeseman, G. Scalmani, V. Barone, G. A. Petersson, H. Nakatsuji, X. Li, M. Caricato, A. V. Marenich, J. Bloino, B. G. Janesko, R. Gomperts, B. Mennucci, H. P. Hratchian, J. V. Ortiz, A. F. Izmaylov,

J. L. Sonnenberg, D. Williams-Young, F. Ding, F. Lipparini, F. Egidi, J. Goings, B. Peng, A. Petrone, T. Henderson, D. Ranasinghe, V. G. Zakrzewski, J. Gao, N. Rega, G. Zheng, W. Liang, M. Hada, M. Ehara, K. Toyota, R. Fukuda, J. Hasegawa, M. Ishida, T. Nakajima, Y. Honda, O. Kitao, H. Nakai, T. Vreven, K. Throssell, J. A. Montgomery, Jr., J. E. Peralta, F. Ogliaro, M. J. Bearpark, J. J. Heyd, E. N. Brothers, K. N. Kudin, V. N. Staroverov, T. A. Keith, R. Kobayashi, J. Normand, K. Raghavachari, A. P. Rendell, J. C. Burant, S. S. Iyengar, J. Tomasi, M. Cossi, J. M. Millam, M. Klene, C. Adamo, R. Cammi, J. W. Ochterski, R. L. Martin, K. Morokuma, O. Farkas, J. B. Foresman, and D. J. Fox. *Gaussian 16, Revision C. 01*; Gaussian, Inc.: Wallingford CT, 2019.

(18) Parr, R. G.; Weitao, Y. *Density-Functional Theory of Atoms and Molecules*; Oxford University Press, 1994.

(19) Lee, C.; Yang, W.; Parr, R. G. Development of the Colle-Salvetti Correlation-Energy Formula into a Functional of the Electron Density. *Phys. Rev. B Condens. Matter* **1988**, *37*, 785–789.

(20) Becke, A. D. A New Mixing of Hartree–Fock and Local Density-functional Theories. *J. Chem. Phys.* **1993**, *98*, 1372–1377.

(21) Grimme, S.; Antony, J.; Ehrlich, S.; Krieg, H. A Consistent and Accurate Ab Initio Parametrization of Density Functional Dispersion Correction (DFT-D) for the 94 Elements H-Pu. *J. Chem. Phys.* **2010**, *132*, 154104.

(22) Hay, P. J.; Wadt, W. R. *Ab Initio* Effective Core Potentials for Molecular Calculations. Potentials for the Transition Metal Atoms Sc to Hg. *J. Chem. Phys.* **1985**, *82*, 270–283.

(23) Wadt, W. R.; Hay, P. J. *Ab Initio* Effective Core Potentials for Molecular Calculations. Potentials for Main Group Elements Na to Bi. *J. Chem. Phys.* **1985**, *82*, 284–298.

(24) Hay, P. J.; Wadt, W. R. *Ab Initio* Effective Core Potentials for Molecular Calculations. Potentials for K to Au Including the Outermost Core Orbitals. *J. Chem. Phys.* **1985**, *82*, 299–310.

(25) Ditchfield, R.; Hehre, W. J.; Pople, J. A. Self-Consistent Molecular-Orbital Methods. IX. An Extended Gaussian-Type Basis for Molecular-Orbital Studies of Organic Molecules. *J. Chem. Phys.* **1971**, *54*, 724–728.

(26) Hariharan, P. C.; Pople, J. A. The Influence of Polarization Functions on Molecular Orbital Hydrogenation Energies. *Theor. Chim. Acta* **1973**, *28*, 213–222.

(27) Hehre, W. J.; Ditchfield, R.; Pople, J. A. Self—Consistent Molecular Orbital Methods. XII. Further Extensions of Gaussian—Type Basis Sets for Use in Molecular Orbital Studies of Organic Molecules. *J. Chem. Phys.* **1972**, *56*, 2257–2261.

(28) Francl, M. M.; Pietro, W. J.; Hehre, W. J.; Binkley, J. S. Self-consistent Molecular Orbital Methods. XXIII. A Polarization-type Basis Set for Second-row Elements. *J. Chem. Phys.* **1982**, *77*, 3654–3665.

(29) Gordon, M. S.; Binkley, J. S.; Pople, J. A.; Pietro, W. J.; Hehre, W. J. Self-Consistent Molecular-Orbital Methods. 22. Small Split-Valence Basis Sets for Second-Row Elements. *J. Am. Chem. Soc.* **1982**, *104*, 2797–2803.

(30) Gonzalez, C.; Schlegel, H. B. An Improved Algorithm for Reaction Path Following. *J. Chem. Phys.* **1989**, *90*, 2154–2161.

(31) Gonzalez, C.; Schlegel, H. B. Reaction Path Following in Mass-Weighted Internal Coordinates. *J. Phys. Chem.* **1990**, *94*, 5523–5527.

(32) Andrae, D.; Häußermann, U.; Dolg, M.; Stoll, H.; Preuß, H. Energy-Adjustedab Initio Pseudopotentials for the Second and Third Row Transition Elements. *Theor. Chim. Acta* **1990**, *77*, 123–141.

(33) Clark, T.; Chandrasekhar, J.; Spitznagel, G. W.; Schleyer, P. V. R. Efficient Diffuse Function-Augmented Basis Sets for Anion Calculations. III. The 3-21+G Basis Set for First-Row Elements, Li-F. *J. Comput. Chem.* **1983**, *4*, 294–301.

(34) Krishnan, R.; Binkley, J. S.; Seeger, R.; Pople, J. A. Self-Consistent Molecular Orbital Methods. XX. A Basis Set for Correlated Wave Functions. *J. Chem. Phys.* **1980**, *72*, 650–654.

(35) McLean, A. D.; Chandler, G. S. Contracted Gaussian Basis Sets for Molecular Calculations. I. Second Row Atoms, Z= 11--18. *J. Chem. Phys.* **1980**, *72*, 5639–5648.

(36) Spitznagel, G. W.; Clark, T.; von Ragué Schleyer, P.; Hehre, W. J. An Evaluation of the Performance of Diffuse Function-Augmented Basis Sets for Second Row Elements, Na-Cl. *J. Comput. Chem.* **1987**, *8*, 1109–1116.

(37) Marenich, A. V.; Cramer, C. J.; Truhlar, D. G. Universal Solvation Model Based on Solute Electron Density and on a Continuum Model of the Solvent Defined by the Bulk Dielectric Constant and Atomic Surface Tensions. *J. Phys. Chem. B* **2009**, *113,* 6378–6396.

(38) Runge, E.; Gross, E. K. U. Density-Functional Theory for Time-Dependent Systems. *Phys. Rev. Lett.* **1984**, *52*, 997–1000.

(39) Neese, F.; Wennmohs, F.; Becker, U.; Riplinger, C. The ORCA Quantum Chemistry Program Package. *J. Chem. Phys.* **2020**, *152*. 224108.
